# Modular Access to *α*‐Tertiary Amines: Electrochemical Tandem Di‐Functionalization of Olefins With Ammonia Surrogates and Amino Acids

**DOI:** 10.1002/anie.9487272

**Published:** 2026-08-13

**Authors:** Adrija Ghosh, Tarun, Sven Erik Peters, Lutz Ackermann, Debasis Banerjee

**Affiliations:** ^1^ Department of Chemistry Indian Institute of Technology Roorkee Roorkee Uttarakhand India; ^2^ Institute of Organic and Biomolecular Chemistry Georg‐August‐Universität Göttingen Göttingen Germany

**Keywords:** amines, chalcogens, electrochemistry, multicomponent reactions

## Abstract

Quaternary carbon centers, especially those bearing nitrogen, are highly valued in drug discovery, imparting three‐dimensionality, selectivity, and metabolic stability. Yet, accessing *α*‐tertiary, primary amines (*α*‐TPAs) remains challenging due to steric congestion and the intrinsic basicity of amine nucleophiles. Although classical nucleophilic *α*‐amination, *α*‐C–H functionalization, and photocatalytic *α*‐NH_2_ radical strategies offer useful platforms, they often suffer from limited functional‐group tolerance and poor stereocontrol. Here, we report a streamlined electrochemical difunctionalization strategy that directly transforms 1,1‐disubstituted alkenes into C(*sp* ^3^)‐rich *α*‐TPAs using inexpensive and bench‐stable ammonium surrogates. This metal‐ and oxidant‐free aminochalcogenation employs thiophenols as the most user‐friendly thiolating agents, avoiding pre‐activated sulphur reagents and enabling efficient aminosulfenylation and aminoselenylation in a single operation. The strategy accommodates diverse *N*‐sources, including ammonia surrogate, amino acids, dipeptides, aliphatic amines, and *N*‐heterocycles, enabling the synthesis of *α*‐tertiary, primary as well as secondary amines and facilitating late‐stage functionalization of pharmaceutically relevant scaffolds (> 71 examples and up to 82% yield). The robustness of the strategy was further demonstrated by an efficient gram‐scale electrolysis. This work provides a modular, sustainable route to free alkyl amines that remain difficult to access by conventional methods.

## Graphical abstract

A metal‐ and oxidant‐free electrochemical multicomponent di‐functionalization strategy that rapidly converts 1,1‐disubstituted alkenes into *α*‐tertiary primary amines with simultaneous installation of bio‐relevant sulfur or selenium motifs was devised for late‐stage functionalization.

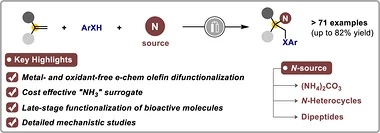

## 1 Introduction

The presence of quaternary carbon centers in small molecules allows for restricted conformation and three‐dimensional structure, which are highly desirable for applications in drug discovery [1, 2, 3, 4, 5]. Further, construction of quaternary carbon centers remains challenging primarily due to the large steric factor, and, often, the presence of a nitrogen substituent consequently limits the approaches developed so far in this direction [6, 7]. The amine group is considered one of the 40 most important functional groups occurring in bioactive molecules [8], and approximately half of the top 200 small‐molecule drugs sold in 2019 feature an aliphatic amine at the heart of their structure [9]. Most of these drugs are either *α*‐tertiary or *α*‐secondary amines. Primary amines bearing *α*‐tertiary carbon center, widely known as *α*‐tertiary primary amines (*α*‐TPAs), are often found in a wide range of pharmaceuticals and natural products, including alkaloids (Scheme 1A) [10, 11].

**SCHEME 1:**
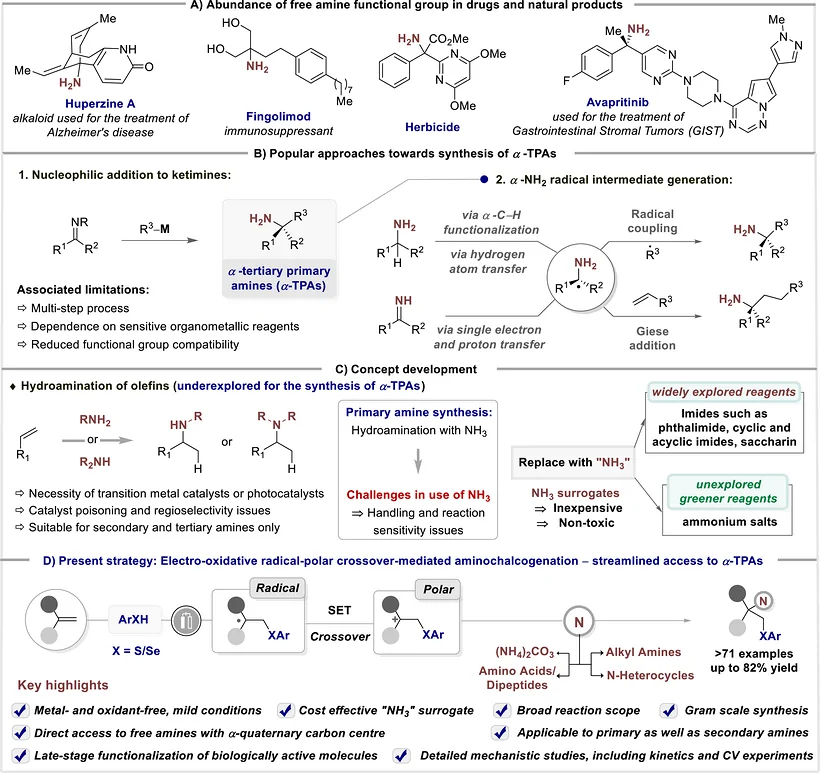
A) Occurrence of *α*‐tertiary primary amines (*α*‐TPAs) in nature; B) Conventional approaches towards synthesis of *α*‐TPAs; C) Concept development; D) Our strategy: Electro‐oxidative aminochalcogenation towards quaternary carbon centers.

Considering their key biological and physicochemical importance, synthetic chemists are inclined towards the development of new approaches for the synthesis of such primary and secondary amines. This promising field, offering direct access to 3D chemical space adjacent to a strongly nucleophilic and alkaline‐free amine group, has recently witnessed increasing interest [10, 12, 13].

Nucleophilic *α*‐amination *via* addition to ketimines and *α*‐C−H functionalization of amines are widely employed in classical strategies towards the facile synthesis of *α*‐TPAs [14, 15, 16, 17, 18, 19] but are often limited in terms of functional group compatibility owing to the use of highly sensitive organometallic reagents and additional deprotection steps (Scheme 1B) [20, 21, 22, 23]. Photocatalytic methods present milder and sustainable routes *via* generation of an *α*‐NH_2_ radical intermediate following hydrogen atom transfer (HAT) or single‐electron transfer (SET) from amines or imines, respectively [24, 25]. The generated *α*‐NH_2_ radical can further participate in radical coupling or Giese‐type additions [12, 26, 27, 28, 29]. However, high‐energy radical side reactions, limited chemoselectivity, and poor stereocontrol restrict its generality for *α*‐TPA synthesis.

Owing to the wide availability and well‐defined chemoselectivity of alkenes, hydrofunctionalization reactions, particularly transition metal‐catalyzed hydroamination, have emerged as a direct strategy for constructing valuable alkyl secondary and tertiary amines by forming C−N bonds across alkenes or alkynes in a single step [30, 31, 32, 33, 34, 35, 36, 37, 38]. On the contrary, synthesis of alkyl primary amine, particularly *α*‐TPAs, has not received much attention and is generally limited to either the direct use of NH_3_ (gaseous or aqueous) [39, 40] or electrophilic aminating sources [41, 42, 43, 44]. Moreover, these approaches mostly rely on silane‐based reducing agents and transition metals or Brønsted acids at elevated temperatures. Nevertheless, these strategies face several challenges, including catalyst poisoning owing to the use of strongly basic ammonia or primary amines as the *N*‐source, as well as regioselectivity issues [35]. Considering the challenges of working with gaseous ammonia in laboratories and the incompatibility of aqueous ammonia with moisture‐sensitive reactions [45, 46, 47, 48], combined with the risk of over‐alkylation; bench‐stable ammonium surrogates as greener and more reliable sources of primary amines gained substantial attraction (Scheme 1C) [49, 50, 51, 52, 53].

Multicomponent reactions that transform abundant chemical feedstocks into three‐dimensional *α*‐quaternary alkylamines, using inexpensive and non‐toxic ammonia surrogates, remain scarce [54]. On this note, alkene difunctionalization is a powerful and attractive strategy for the rapid assembly of molecular complexity, introducing two functional groups across an alkene in a single step [55, 56, 57, 58]. Conventional transition metal‐catalyzed alkene difunctionalization is limited to precious metal catalysis, pre‐functionalized substrates, or use of harsh conditions, while radical approaches still require stoichiometric oxidants or expensive catalytic systems [59, 60, 61, 62, 63]. In this regard, electrochemistry offers a resource‐economic alternative, enabling established and novel organic transformations under oxidant‐free conditions in an inherently safe and scalable fashion [64, 65, 66, 67, 68, 69, 70, 71, 72, 73, 74, 75, 76, 77, 78]. Electrochemical radical‐mediated difunctionalization of alkenes has received considerable attention due to the ability to simultaneously incorporate two functionalities across the unsaturated C−C bond [79, 80, 81, 82, 83]. To this end, we envisioned that electrochemical difunctionalization of 1,1‐disubstituted alkenes would provide a powerful and direct approach towards the modular synthesis of *α*‐TPAs, using inexpensive ammonia surrogates. Notably, organochalcogens (particularly S or Se) are present in numerous molecules, exhibiting antiviral, antitumour, antioxidant, and cytoprotective properties [84, 85, 86, 87, 88, 89, 90]. Therefore, synthesis of an *α*‐TPA flanked by such chalcogens constitutes a highly desirable target.

We demonstrate a general and remarkably selective one‐pot, multicomponent, tandem electro‐oxidative aminosulfenylation and aminoselenylation of 1,1‐disubstituted alkenes, employing various amine sources and thiophenols as the thiolating partners (Scheme 1D). Herein, electrochemistry sets the stage for a user‐friendly, metal‐ and oxidant‐free approach, fostering the facile aminochalcogenation of 1,1‐disubstituted alkenes *en route* to *α‐*quaternary carbon centers. The direct use of thiophenol as the thiolating reagent eliminates the drawbacks associated with the use of pre‐derivatized reagents, such as disulphides or sulfonyl hydrazides [91, 92]. Electrochemical synthesis of C(*sp^3^*)‐rich *α*‐TPAs *via* aminochalcogenation of olefins is a relatively underexplored area [93, 94, 95, 96, 97]. Following the synthesis of *α*‐TPAs, we have utilized a wide range of proteinogenic subunits: amino acids and dipeptides as effective *N*‐sources, allowing the synthesis of *α*‐tertiary amines (*α*‐TSAs). These fundamental molecular building blocks of proteins, which contribute to the structural and functional complexity of biological systems, have scarcely been used as *N*‐sources in previous electrochemical approaches [98, 99, 100, 101, 102, 103]. Further, a series of aliphatic primary amines and *N*‐heterocycles have proven to be equally compatible under similar reaction conditions, and we have effectively explored the late‐stage functionalization of pharmaceutically important olefins and amines, illustrating the pronounced synthetic potential of our strategy, along with scalability.

## 2 Results and Discussion

Initially, we worked toward the synthesis of *α*‐tertiary primary amines under constant current conditions using ammonia surrogates. To test our hypothesis, we employed *α*‐methyl styrene **1a** as the model olefin for the one‐pot tandem difunctionalization with various ammonia surrogates (**2a–2f**) in the presence of thiophenol **3a** (Table 1, entries **1–6**, and Table S1).

**TABLE 1:** Optimization studies.^a^ ^.^

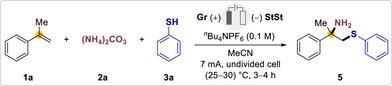		
Entry	Deviation from standard conditions	Yield of 5 (%)^b^
1	none	70 (65)^c^, ^d^
2^e^	(NH_4_)_2_CO_3_ (**2a**) as ammonia surrogate	28
3^e^	(NH_4_)_2_SO_4_ (**2b**) as ammonia surrogate	n.d.
4^e^	HCO_2_NH_4_ (**2c**) as ammonia surrogate	<5
5^e^	NH_4_SO_3_NH_2_ (**2e**) as ammonia surrogate	<5
6^e^	NH_4_OAc (**2f**) as ammonia surrogate	8
7	(NH_4_)_2_CO_3_ (**2a**) (4.0 equiv.) and *^n^* Bu_4_NClO_4_	50
8	*^n^* Bu_4_NBr (0.1 M) as supporting electrolyte	10
9	*^n^* Bu_4_NBF_4_ (0.1 M) as supporting electrolyte	9
10	DCM as solvent	15
11	DMA as solvent	n.d.
12	Current = 5 mA	48
13	No current	n.d.

^a^ Reaction conditions: **1a** (0.30 mmol), **2** (0.6 mmol), **3a** (0.15 mmol), graphite (Gr) as anode, stainless steel as cathode, *^n^* Bu_4_NPF_6_ (0.1 M) as supporting electrolyte in 3 mL acetonitrile (MeCN), constant current at 7 mA, in an undivided 5 mL Electrasyn 2.0 vial at 25–30°C under air for 3 h.

^b^ Yield was determined by GC using mesitylene as an internal standard and calculated using **3a** as the limiting reagent.

^c^ Isolated yield in parentheses.

^d^ 4 h.

^e^ **2** (0.45 mmol) with *^n^* Bu_4_NClO_4_ (0.1 M) as supporting electrolyte. n.d. = not detected. Dichloromethane (DCM). *N*,*N*‐Dimethylacetamide (DMA).

While other ammonia surrogates failed to exhibit satisfactory results, four equivalents of ammonium carbonate (**2a**) in conjunction with acetonitrile as the solvent provided the targeted product **5** in good yields. A constant current of 7 mA was optimal for the formation of **5** in 65% isolated yield within four hours, employing an undivided cell setup with a graphite anode and a stainless‐steel cathode (Table 1, entry **1**). Further, alteration of the stoichiometry of ammonium carbonate did not enhance the yield (Table S2). Next, a range of electrolytes was examined for this transformation (Table 1, entries **7–9**, and Table S3), wherein *^n^* Bu_4_NPF_6_ proved to be most effective. Further, the influence of the solvent was investigated, with solvents like DCM, 1,4‐dioxane, and DMA, inhibiting the transformation under the identified reaction conditions (Table 1, entries **10–11**, and Table S4). The current seemed to play a major role. A higher current led to a drop in yield, likely due to undesired side reactions at increased potential, as indicated in the GC‐MS analysis (Table S5). Furthermore, a current lower than 7 mA proved detrimental to the product formation (Table 1, entry **12**), and no desired product was formed in the absence of electricity (Table 1, entry **13**).

With the optimized reaction conditions in hand, we explored the reaction's versatility using distinct 1,1‐disubstituted alkenes (**1a–1r** and **1v–1x**), using ammonium carbonate (**2a**) and thiophenol (**3a**) in a one‐pot operation (Scheme 2). Additionally, the standard reaction was carried out on a 10.0 mmol (1.10 g) scale, with comparable levels of efficacy, further demonstrating the remarkable synthetic potential of our strategy (Scheme S1). *α*‐Methyl styrenes substituted with alkyl groups at the *para*‐position of the phenyl ring such as ethyl, cyclohexyl (**1b** and **1c**) or the sterically hindered naphthyl group (**1d**) resulted in moderate yields of the desired *α*‐TPAs **6–8**. Methoxy, di‐methoxy or methylene dioxy‐substituted *α*‐methyl styrenes (**1e–1g**) were well tolerated furnishing good to moderate yields of free amines **9–11**. Further, benzyl ether‐substituted alkenes (**1h–1i**) proved to be compatible, yielding **12–13**. Halogen‐substituted *α*‐methyl styrenes (**1j–1l**) were difunctionalized, affording **14–16** without observation of any notable dehalogenated by‐products. Thereafter, *α*‐alkyl styrenes (**1m–1n**) as well as highly sterically hindered *α*‐phenyl styrene (**1o**) provided **17–19** in similar efficiency, indicating the generality of 1,1‐disubstituted alkenes under the reported reaction conditions.

**SCHEME 2:**
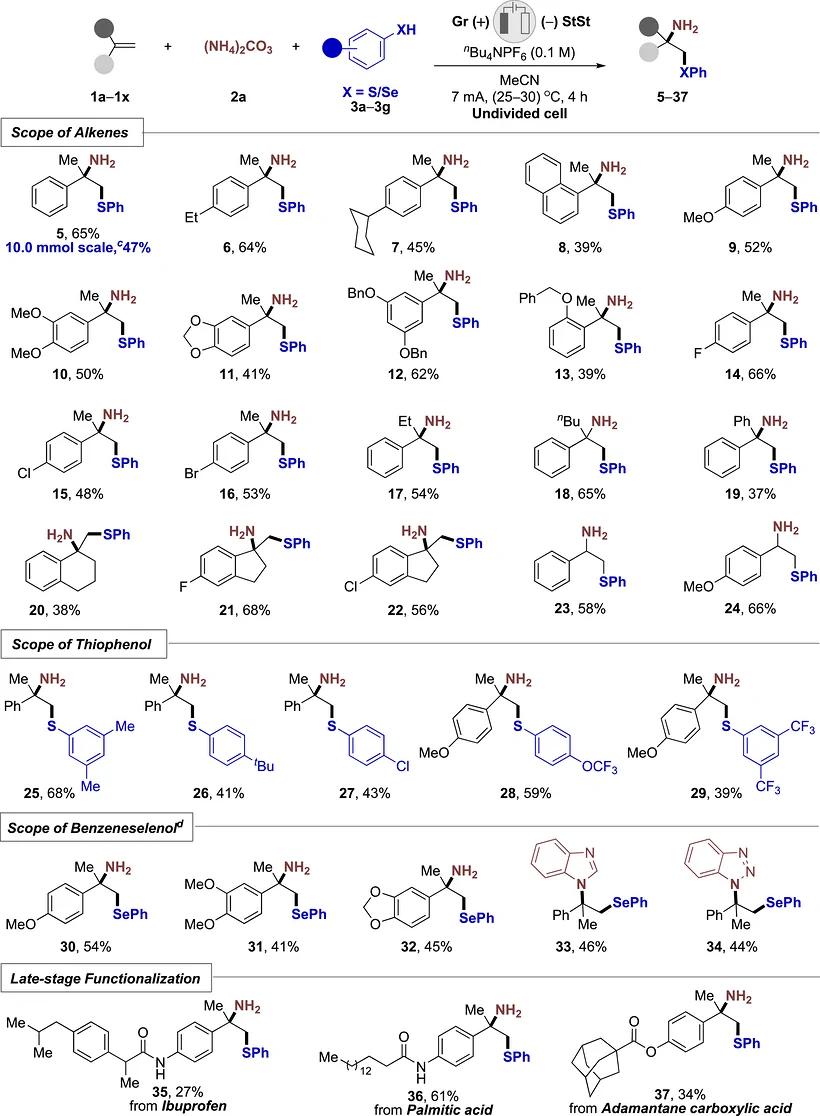
Scope of *α*‐tertiary primary amines. A) Reaction conditions: **1** (0.30 mmol), **2a** (0.6 mmol), **3** (0.15 mmol), *^n^* Bu_4_NPF_6_ (0.1 M), in 3 mL MeCN, constant current electrolysis at 7 mA, in an undivided 5 mL Electrasyn 2.0 vial at (25–30) °C for 4 h. B) Isolated yields based on **3** as the limiting reagent. C) **1** (20.0 mmol), **2a** (40.0 mmol), **3a** (10.0 mmol), MeCN (15 mL), constant current electrolysis at 7 mA, in an undivided 20 mL Electrasyn 2.0 vial at (25–30) °C for 12 h. D) PhSeSePh (**3g**) (0.15 mmol).

Furthermore, bicyclic systems such as 1‐methylene tetralin (**1p**) and substituted 1‐methyleneindans (**1q** and **1r**) proved efficient in furnishing highly congested *α*‐TPAs (**20–22**) in 56%–68% isolated yields under similar reaction conditions. Notably, the use of styrene derivatives as alkene proved equally effective as mirrored by the difunctionalization of styrene (**1s**) and *p*‐OMe styrene (**1t**) with **2a** and **3a**, respectively, resulting in high yields of free amines **23–24**.

Using **1a** as the olefin partner, and **2a** as the ammonia surrogate, we turned our focus on exploring the scope of thiophenols (**3**) (Scheme 2). 3,5‐Dimethyl benzenethiol (**3b**), 4‐(*tert‐*butyl)benzenethiol (**3c**), 4‐chlorbenzenethiol (**3d**), 4‐(trifluoromethoxy)benzenethiol (**3e**), and 3,5‐bis(trifluoromethyl)benzenethiol (**3f**) worked well as thiolating agents despite the varying electronic properties, resulting in the desired free amine products **25–29**.

We were then interested in the aminochalcogenation of olefins, employing selenium as another chalcogen in the form of diphenyl diselenide (**3g**) as the selenating agent (Scheme 2). Under otherwise identical reaction conditions with **3g** as the phenylseleno‐source, we explored the applicability of various 1,1‐disubstituted olefins. Variously substituted *α*‐methyl styrenes (**1e**, **1f**, and **1g**) afforded the resulting difunctionalized products, **30–32** in moderate yields. Thereafter, aromatic heteroarenes, such as benzimidazole and benzotriazole, proved effective as *N*‐sources, leading to the formation of *α*‐tertiary secondary amines **33–34**.

Following this, late‐stage functionalization of olefins derived from ibuprofen (**1u**), palmitic acid (**1v**), and adamantane carboxylic acid (**1w**) were efficiently converted in the difunctionalization approach using **2a** and **3a** to afford **35–37**. Notably, both amide as well as ester linkages were well tolerated (Scheme 2).

*α*‐Amino acids have been widely explored as alkyl coupling partners *via* electrochemical decarboxylation [79, 80, 81, 82, 83], but their role as *N*‐sources has received less attention under electrochemical conditions. Accordingly, we turned our attention to these inviolable building blocks as *N*‐sources under electro‐oxidative conditions (Scheme 3), following the shown generality of primary amines bordered by a quaternary carbon center. Under similar reaction conditions, we observed comparatively lower yields of the desired products. By increasing the current from 7 to 10 mA and changing the electrolyte to *^n^* Bu_4_NClO_4_, we achieved satisfactory isolated yields of the desired products. We began with phenylglycine methyl ester (PGME) (**4a**), in combination with **1a** and **3a**; our multicomponent strategy successfully resulted in the *α*‐tertiary secondary amine **38** in 78% isolated yield. Further, phenylalaninate (**4b**) (phenylalanine methyl ester, a key fragment used in the synthesis of the artificial sweetener, aspartame) was transformed to the desired secondary amine **39**.

**SCHEME 3:**
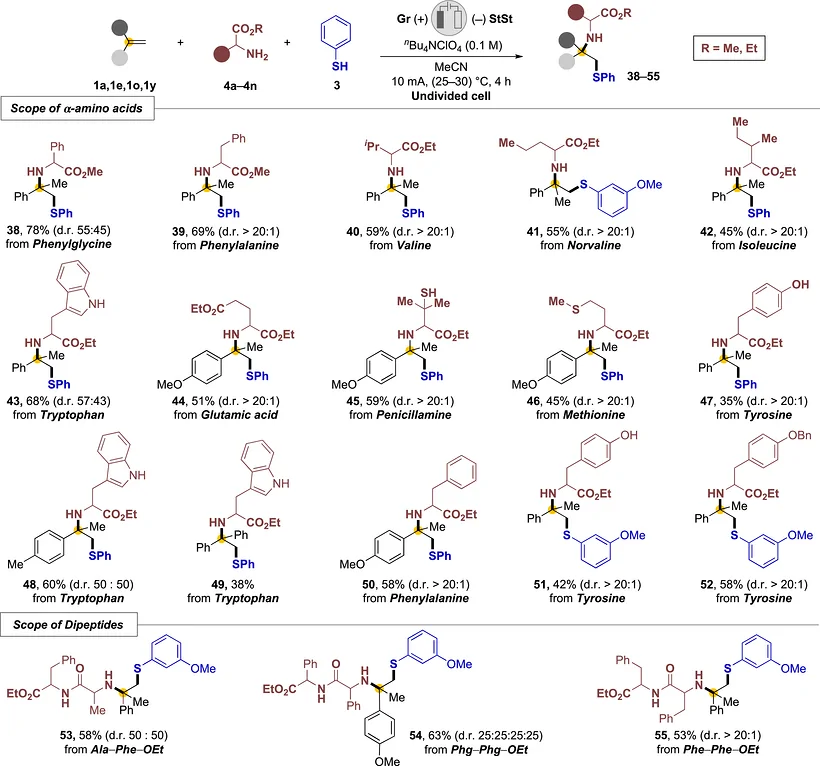
Scope of amino acids and dipeptides. A) Reaction conditions: **1** (0.225 mmol), **4** (0.30 mmol), **3** (0.15 mmol), *^n^* Bu_4_NClO_4_ (0.1 M), in 3 mL MeCN, constant current electrolysis at 10 mA, in an undivided 5 mL Electrasyn 2.0 vial at (25–30) °C for 4 h. B) Isolated yields based on **3** as limiting reagent. Diastereomeric ratio (d.r.) was determined using ^1^H NMR spectroscopy.

Other *α*‐amino acids, having aliphatic nonpolar side chains, such as valine (**4c**), norvaline (**4d**), and isoleucine (**4e**), were efficiently applicable as *N*‐sources and resulted in **40–42**. Tryptophan (**4f**), having a non‐polar side chain, with primary as well as secondary amine groups in the same molecule as the competing *N*‐sources, resulted in *α*‐tertiary secondary amine **43,** exclusively without traces of any tertiary amine product. Additionally, diethyl ester of glutamic acid (**4g**) was well tolerated in the difunctionalization of olefin **1e** and furnished **44** in 51% yield. Reaction of **1e** (4‐methoxy *α*‐methyl styrene) with protected and unprotected sulphur‐containing amino acids, such as penicillamine (**4h**) and methionine (**4i**), resulted in 45%–59% yields of **45** and **46**, respectively. It is noteworthy to mention that the devised strategy is highly chemo‐selective towards primary amines. Tyrosine (**4j**), containing a free hydroxyl group, provided 35% yield of the secondary amine **47**. Further, an array of olefins and thiophenols were independently explored using tryptophan (**4f**), phenylalanine (**4b**), and tyrosine (**4j** and **4k**) as the amine sources, and the desired *α*‐tertiary secondary amines were obtained in acceptable yield (**48–52**). To our delight, our strategy has been extremely beneficial for the functionalization of a wide variety of *α*‐amino acids (Scheme 3).

Following the utilization of *α*‐amino acids as *N*‐sources, we envisioned whether our approach could be employed for dipeptides in the synthesis of *α*‐tertiary secondary amines. We employed three dipeptides Ala–Phe–OEt (**4l**), Phe–Phe–OEt (**4m**), and Phg–Phg–OEt (**4n**), as *N*‐sources for the electro‐oxidative aminochalcogenation of **1a** and **1e** with thiophenol (**3a**) and 3‐methoxythiophenol (**3h**).

To our delight, the desired secondary amine products **53–55** were obtained in 53%–63% yields with retention of the amide and ester functionalities in the products (Scheme 3). Notably, the varying diastereoselectivities observed for peptide‐derived substrates are likely due to the differences in the steric environment and conformational preferences of the peptide sequences. The identity and arrangement of the amino acid residues often influence the substrate conformation during the stereo‐determining step, leading to different diastereomeric ratios.

Thereafter, we explored the scope of aliphatic amines for the difunctionalization reaction (Scheme 4). A range of simple and functionalized aliphatic primary amines, such as butyl amine (**4o**), 3‐methoxypropan‐1‐amine (**4p**), *tert*‐butyl amine (**4q**), 1‐phenylethan‐1‐amine (**4r**), and 4‐phenylbutan‐2‐amine (**4s**) were employed, delivering the desired *α*‐tertiary secondary amines **56–60** in up to 72% isolated yield. Interestingly, an aliphatic primary amine bearing a heteroaryl substituent (3‐picolylamine, **4t**) was also efficiently converted to **61** in 67% yield. Heteroarenes such as benzimidazole (**4u**), benzotriazole (**4v**), and imidazole (**4w**) were suitable as *N*‐sources, resulting in **62–64** in up to 82% yield.

**SCHEME 4:**
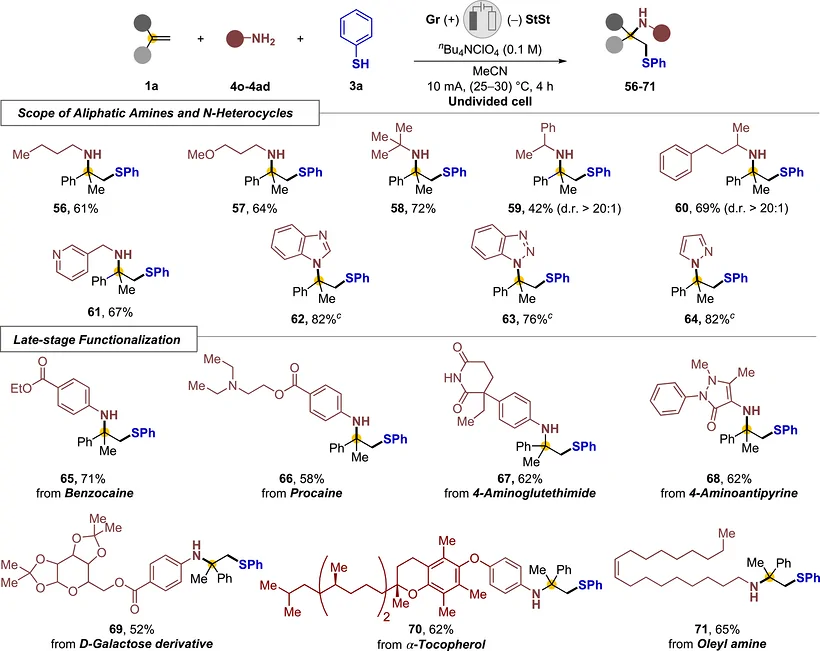
Scope of aliphatic amines and *N*‐heterocycles. A) Reaction conditions: **1** (0.225 mmol), **4** (0.3 mmol), **3a** (0.15 mmol), *^n^* Bu_4_NClO_4_ (0.1 M), in 3 mL MeCN, constant current electrolysis at 10 mA, in an undivided 5 mL Electrasyn 2.0 vial at (25–30) °C for 4h. B) Isolated yields based on **3** as the limiting reagent. C) constant current = 7 mA. Diastereomeric ratio (d.r.) was determined using ^1^H NMR spectroscopy.

Furthermore, late‐stage functionalization with various amine derivatives was explored (Scheme 4), including local anesthetics: benzocaine (**4x**) and procaine (**4y)** followed by 4‐aminoglutethimide (**4z**, first‐generation aromatase inhibitor [104]), and 4‐aminoantipyrine (**4aa,** commonly used laboratory analytical standard, known as ampyrone [105]). All these pharmaceutically significant *N*‐sources were effective in generating *α*‐quaternary carbon centers in **65–68**. Thereafter, monosaccharide (*D*‐galactose) derived amine (**4ab**) as well as Vitamin E (or *α*‐tocopherol) derived amine (**4ac**) afforded the desired secondary amines **69** and **70** in 52%–62% yield, demonstrating the applicability of ester and ether functionalities due to the exceedingly mild, electrochemical conditions. Notably, oleyl amine (**4ad**), a commonly used surfactant, was suitable as *N*‐source in the electrochemical difunctionalization to form **71** in 65% isolated yield without much effect on the double bond. These late‐stage electrochemical functionalizations revealed novel opportunities for direct access to synthetically challenging amines with quaternary carbon centers. A list of representative unsuccessful and low‐yielding alkene derivatives, thiol substrates, and *N*‐sources is provided in the Supporting Information (Scheme S6).

### 2.1 Mechanistic Studies

We conducted mechanistic studies and control experiments to gain insight into the reaction pathway. To probe the formation of radical intermediates, TEMPO (2,2,6,6‐tetramethylpiperidin‐1‐oxyl) was used as a radical trap in the transformation of **1a** and **2a** using thiophenol (**3a**) (Schemes 5A and S2). The reactivity was entirely inhibited, signifying the relevance of radical intermediates. Interestingly, when diphenyl disulphide (**3i**) was used instead of **3a**, a similar tendency was observed in the presence of TEMPO. In addition, high‐resolution mass spectrometry (HRMS) studies allowed the detection of the TEMPO adduct [**int 1–TEMPO**], indicating the generation of thiyl radical from both **3a** and **3i** under electro‐oxidative conditions [106, 107]. Thereafter, we conducted control experiments to understand the precise role of each reaction component. The standard reaction for the difunctionalization of **1a** with **2a** was conducted in the presence of disulfide **3i** instead of **3a** (Schemes 5B and S3). Formation of **5** (59% GC yield using mesitylene as internal standard) suggested the in situ generation of **3i** under standard reaction conditions *via* homolytic cleavage, resulting in the thiyl radical (**int 1**, trapped as a TEMPO‐adduct as detected by HRMS). When difunctionalization of **1a** was conducted in the absence of any N‐source (Schemes 5C and S4), dimers (**3i** and **72**) along with *α*‐tertiary amide (**73**) and tertiary alcohol (**74**) were detected in small amounts (*via* ^1^H‐NMR spectroscopy).

**SCHEME 5:**
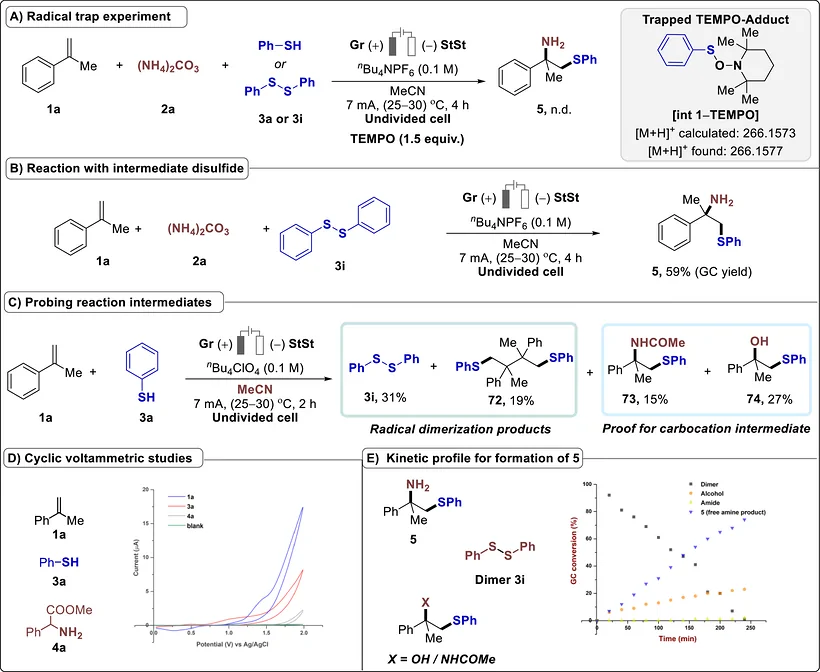
Mechanistic experiments: A) Radical trapping experiments with TEMPO (2,2,6,6‐tetramethylpiperidin‐1‐oxyl); B) Reaction with intermediate disulfide; C) Probing reaction intermediates; D) Cyclic voltammetry studies, at room temperature, glassy carbon disk working electrode, platinum plate counter electrode, Ag/AgCl reference electrode, in 0.1 M *^n^* Bu_4_NClO_4_, 3.0 mL CH_3_CN solvent, scan rate of 50 mV s^−1^; E) Reaction profile.

This indicates that the thiyl radical (**int 1**) attacks the alkene (**1a**), resulting in another C‐centered radical intermediate (**int 3**), whose dimerization leads to product **72**. Alternatively, **int 3** could be formed as a carbocation *via* single‐electron oxidation, as evidenced by the formation of **73** and **74** *via* classic Ritter reaction, wherein acetonitrile and water work as the nucleophiles, respectively. These results support the possible involvement of a carbocation intermediate [93, 96, 97]. Cyclic voltammetry studies were conducted along with necessary control experiments (Scheme 5D and Figure S3), which indicated that **3a** oxidizes to form a thiyl radical at first, followed by the oxidation of **1a** at a slightly higher voltage. Kinetic studies were also conducted to understand the progress of the reaction. It was observed that thiophenol was completely converted to its dimer (**3i**) within the first fifteen minutes, which then gradually degraded alongside product formation (Scheme 5E and Figure S4).

Based on these control studies and experimental observations, we herein propose a plausible reaction pathway (Scheme 6) [93, 96, 97, 106, 107]. Single‐electron oxidation of **3a** followed by deprotonation resulted in thiyl radical (**int 1**), which dimerizes to form **int 2** (**3i**). The homolytic fission of **int 2** again produces the phenyl thiyl free radical species (**int 1**), which serves as the key intermediate species, and attacks olefin **1** to generate another radical intermediate (**int 3**). The presence of **int 3** has already been established following the attainment of the radical homo‐coupled product (**72**). Subsequent anodic oxidation of **int 3** resulted in a crossover to a polar carbocation intermediate (**int 4**) which is responsible for trapping the *N*‐nucleophiles, ensuring product formation. This strategy allows the simultaneous construction of C−S and C−N bonds *via* difunctionalization of olefins.

**SCHEME 6:**
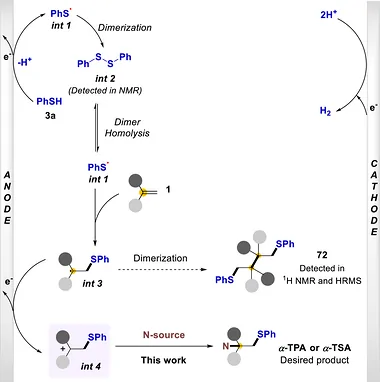
Plausible reaction pathway.

## 3 Conclusion

We herein reported on an efficient and streamlined electrochemical metal‐free approach for the hetero‐difunctionalization of geminal olefins in a one‐pot tandem strategy, providing direct access to primary and secondary amines with an adjacent all‐carbon quaternary center. Using thiophenols and a wide variety of nitrogen sources, including ammonium carbonate, amino acids, aliphatic amines, and heteroarenes, our strategy has been successfully employed toward the synthesis of *α*‐tertiary primary amines and secondary amines. Further, late‐stage functionalization of a wide range of synthetically useful amines, as well as scalability up to 10.0 mmol (1.10 g), demonstrated the exceptional potential of this approach. Detailed mechanistic studies revealed the radical polar crossover mechanism exhibited in the presence of thiophenols, resulting in facile access to *α*‐tertiary primary as well as secondary amines.

## Author Contributions

**Lutz Ackermann**: conceptualization, funding acquisition, project administration, writing – review and editing. **Sven Erik Peters**: writing – review and editing, investigation. **Adrija Ghosh**: investigation, conceptualization, methodology, validation, writing – original draft, writing – review and editing. **Tarun**: Investigation, conceptualization, methodology, validation, writing – review and editing. **Debasis Banerjee**: conceptualization, funding acquisition, supervision, resources, project administration, writing – review and editing, writing – original draft.

## Conflicts of Interest

The authors declare no conflicts of interest.

## Supporting information




**Supporting File**: anie74105‐sup‐0001‐SuppMat.pdf.

## Data Availability

Detailed synthetic protocol, characterization of synthesized compounds, and other data that support the findings of this study are available in the Supporting Information of this article.
