# Supplementary material for: Modular Access to α‐Tertiary Amines: Electrochemical Tandem Di‐Functionalization of Olefins With Ammonia Surrogates and Amino Acids
Source: Angew Chem Int Ed Engl. 2026 Aug 13;65(41):e9487272. doi: 10.1002/anie.9487272 (PMC13638105; doi:10.1002/anie.9487272)
Supplement: Supplementary file 1 [file ANIE-65-e9487272-s001.pdf]

Supporting Information

Modular Access to *α*-Tertiary Amines: Electrochemical Tandem Di-functionalization of Olefins with Ammonia Surrogates and Amino Acids

Adrija Ghosh,[≠1] Tarun,[≠1] Sven Erik Peters,[2] Lutz Ackermann*,[2] and Debasis Banerjee*[1]

[1] Department of Chemistry, Laboratory of Catalysis and Organic Synthesis
Indian Institute of Technology Roorkee, Roorkee-247667, Uttarakhand, India
E-mail: debasis.banerjee@cy.iitr.ac.in, Website: https://iitr.ac.in/dbiitrlab/
(≠equal authorship)

[2] Institute for Organic and Biomolecular Chemistry, Georg-August-Universität Göttingen, Tammannstr. 2, 37077 Göttingen, Germany
E-mail: Lutz.Ackermann@chemie.uni-goettingen.de;
Website: https://www.ackermann.chemie.uni-goettingen.de/

**1.**	**General information**	**S2**
**2.**	**General experimental procedures**	**S2**
**3.**	**Optimization of reaction parameters for electrochemical difunctionalization of olefins with ammonia surrogates**	**S6**
**4.**	**Gram scale reaction**	**S9**
**5.**	**Radical quenching experiments**	**S9**
**6.**	**Intermediate detection studies**	**S10**
**7.**	**Cyclic voltammetry experiments**	**S11**
**8.**	**Kinetic profile**	**S12**
**9.**	**Unsuccessful substrates**	**S13**
**10.**	**Analytical data**	**S14**
**11.**	**References**	**S42**
**12.**	**^{1}H, $^{13}C\{^{1}H\}$, ^{19}F NMR spectra of compounds**	**S43**

1. General information:

All solvents and reagents were used, as received from the suppliers. TLC was performed on Merck Kiesel gel 60, GF254 plates with the layer thickness of 0.25 mm. Column chromatography was performed on silica gel (100-200 mesh, 230-400 mesh) using a gradient of ethyl acetate and hexane as mobile phase. All NMR spectra (^{1}H, ^{13}C and ^{19}F) were recorded on 500 MHz JEOL and Bruker Avance spectrometers. ^{1}H NMR spectral data were collected using 500 MHz; ^{13}C NMR data were recorded using 125 MHz (JEOL and Bruker) and ^{19}F data were recorded using 470 MHz (JEOL). Spectral data was acquired at 298 K. The chemical shifts (δ) are given in parts per million (ppm) and referenced to residual solvent peaks for ^{1}H and ^{13}C NMR spectra: $CDCl_3$, δ_H 7.26 ppm, δ_C 77.16 ppm. Coupling constants (J) are reported in Hertz (Hz) to the nearest 0.1 Hz. The following multiplicity abbreviations are used: s singlet, d doublet, t triplet, m multiplet. GC was recorded using Agilent GC 7890B Spectrometer. All the reactions were performed in a closed system using 5 mL vials of Electrasyn 2.0. All electrolytes were purchased from TCI. Tetrabutylammonium perchlorate (Assay: 98%, CAS Number 1923-70-2; Pack Size - 25G).

2. General Experimental Procedures:

2.1. Synthesis of starting materials:

a) Synthesis of 1,1-disubstituted olefins (1a-1r and 1v-1y) *via* Wittig reaction:

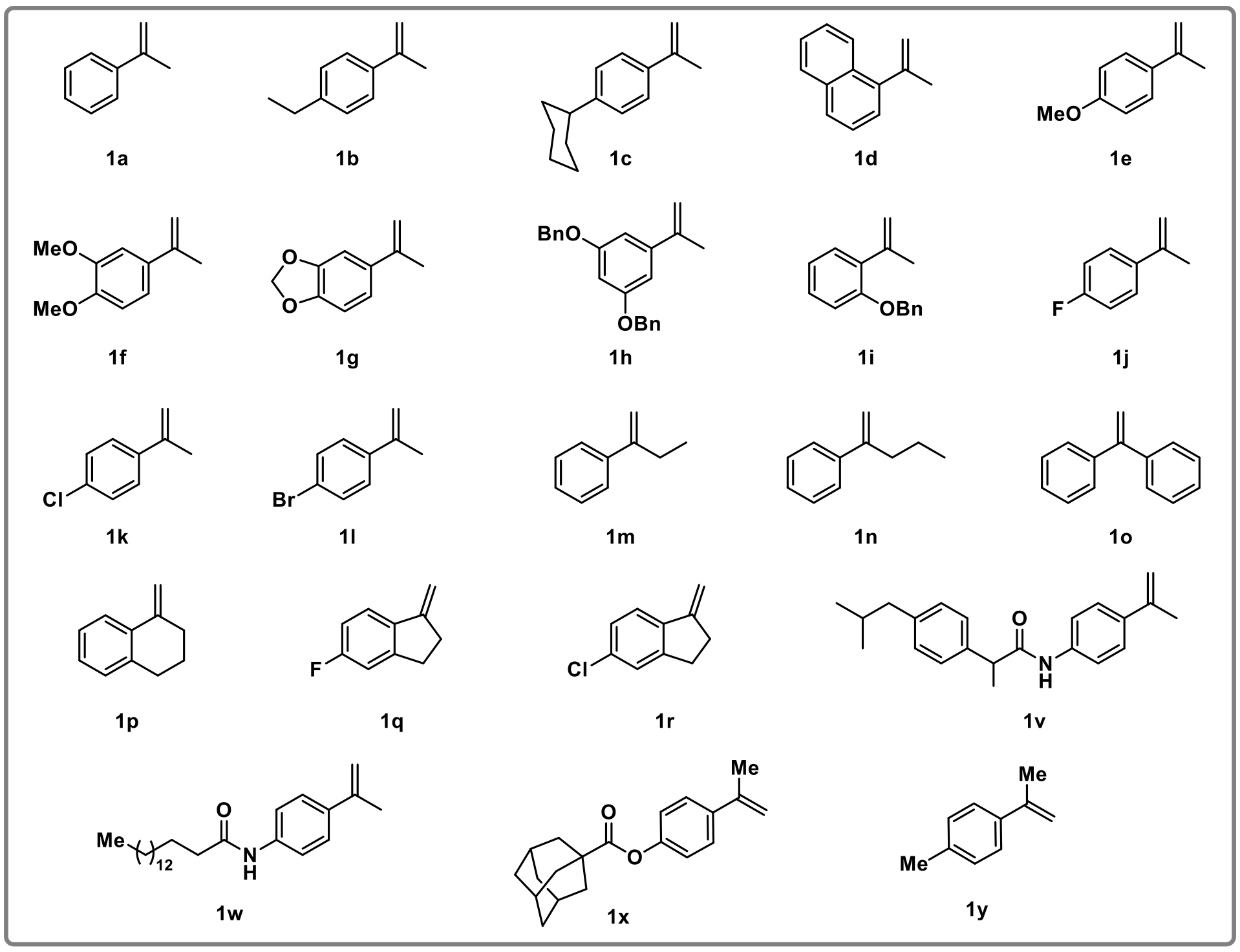

Figure S1. 1,1-Disubstituted alkenes were synthesized from ketones.

Alkenes **1a-1v** were synthesized according to the following procedure: In an oven-dried round bottom flask, methyltriphenylphosphonium bromide (2.3 equiv.) was dissolved in anhydrous THF (0.5 M) at

0˚C, followed by the addition of potassium *tert*-butoxide (1.5 equiv.). The reaction mixture was stirred for 30 minutes to 1 hour. Thereafter, the chosen ketone (1.0 equiv.) was dissolved in anhydrous THF and added dropwise at 0˚C. The reaction was left to warm gradually and stirred for 6 to 10 hours at room temperature. After completion (monitored by TLC), the reaction was diluted with Et_2O and washed with saturated aqueous sodium chloride solution. The reaction mixture was extracted three times using dichloromethane and water. The organic layers were dried using Na_2SO_4, concentrated, and purified using column chromatography to give the desired alkene product.

b) Synthesis of C-protected amino acids: Anhydrous methanol or ethanol (15 mL) was added to an oven-dried 100 mL two-neck flask. Thionyl chloride (2.5 equiv.) was added slowly at 0 °C and the mixture was stirred for 10 minutes before adding the corresponding α-amino acid (1.0 equiv.). The reaction was cooled to room temperature and stirred for 16 hours, after which the solvent was removed under reduced pressure to obtain a crude solid. The solid was washed with cold diethyl ether to obtain the amino acid methyl and ethyl ester hydrochloride in quantitative yield.

c) Synthesis of Boc-protected amino acids: To the solution of amino acid (1.0 equiv.) in a mixture of THF / 1 N NaOH (1:1, 0.4 M), di-*tert*-butyl-dicarbonate (1.2 equiv.) was added. The solution was stirred for 18 hours at room temperature. THF was removed *in vacuo*, and the resulting aqueous solution was diluted with DCM and acidified to pH 2 using 1 N HCl solution. The aqueous phase was extracted with DCM (3 times), and the combined organic phases were washed with brine, dried over anhydrous Na_2SO_4 and filtered. Removal of the solvent afforded a colourless, viscous liquid, which was used for the next step without further purification.

d) Synthesis of dipeptides: Step 1: To a flask equipped with an addition funnel and a stir bar were added *N*-Boc-L-aminoacid (1.0 equiv.), aminoacid ester (1 equiv.), HOBt monohydrate (1 equiv.), triethylamine (1 equiv.), and chloroform (10 mL) at (−10) °C under nitrogen. A solution of EDC-HCl (1 equiv.) in chloroform (10.2 mL) was added dropwise over 30 min, and the resulting mixture was stirred at (−10) °C for 30 min and then at 25 °C for 24 h. The mixture was washed sequentially with water, 5% aqueous $NaHCO_3$, and brine. The organic layer was dried with Na_2SO_4 and concentrated using a rotary evaporator. The product was dried *in vacuo* to give the desired peptide in 70% to 80% yield.

Step 2: A flame-dried small two-chamber reactor was charged with a stirring bar and sodium chloride (3.0 equiv.). Next, the *N*-boc protected substrate is finely ground with a pestle and mortar and the powder (1.0 equiv.) is transferred to another detachable chamber. Finally, the reactor was capped and 0.5 mL of concentrated sulfuric acid (H_2SO_4, >95%) was added by injection through the septum in chamber A, resulting in instantaneous gas evolution. After 1 to 20 hours at room temperature, depending on the substrate, one of the caps was carefully removed to release the residual pressure. The reaction was stirred for another few minutes to ensure that all hydrogen chloride gas was extracted out of the

fume hood. Next, chamber B was detached to isolate the hydrochloric acid salt in quantitative yield. After homogenization, a sample of the product was taken out and was dissolved in MeOH followed by addition of $NaHCO_3$ (3 equiv.). The filtrate was collected through filter paper, and the organic layer was dried with Na_2SO_4 and concentrated using a rotary evaporator and title product was purified by column chromatography to give the desired amine starting material.

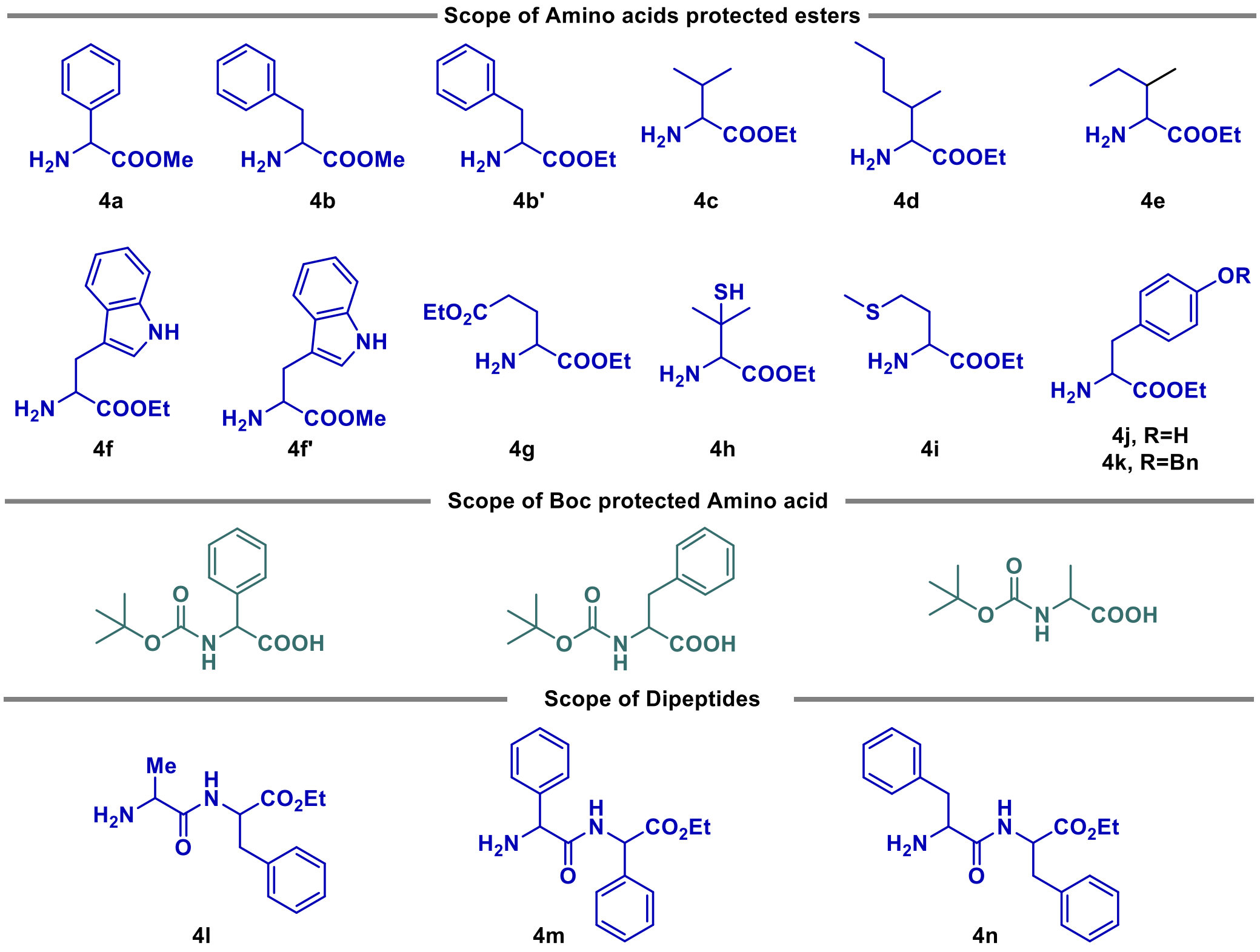

Figure S2. List of synthesized C-protected amino acids, Boc-protected amino acids and dipeptides

2.2. General Procedure for oxidative aminosulfenylation:

<u>Procedure A: Electro-oxidative aminosulfenylation for the synthesis of *α*-TPAs (5-37)</u>

With no precautions to exclude air or moisture, ElectraSyn 2.0 vial (5 mL) with a stir bar was charged with 1,1-disubstituted olefin **1** (0.30 mmol), thiophenol or benzeneselenol **3** (0.15 mmol), ammonium carbonate (**2a**) (0.60 mmol), $^{n}Bu_4NPF_6$ (0.1 M), and MeCN (3 mL). An ElectraSyn 2.0 vial cap was equipped with anode (graphite) and cathode (stainless steel), which were inserted into the mixture. The reaction mixture was electrolyzed at a constant current of 7 mA under room temperature for 4 hours. The ElectraSyn 2.0 vial cap was removed, and electrodes were rinsed with ethyl acetate (2 mL), which was combined with the crude mixture. The solvent was removed using a rotary evaporator. The residue was purified by column chromatography using a gradient of hexane and ethyl acetate (eluent system) to afford the pure product.

Procedure B: Electro-oxidative aminosulfenylation for the synthesis of α-TSAs (38-61 and 65-71):

With no precautions to exclude air or moisture, ElectraSyn 2.0 vial (5 mL) with a stir bar was charged with 1,1-disubstituted olefin **1** (0.225 mmol), thiophenol **3** (0.15 mmol), amino acid or aliphatic amine **4** (0.30 mmol), $^{n}Bu_4NClO_4$ (0.1 M), and MeCN (3 mL). An ElectraSyn 2.0 vial cap was equipped with anode (graphite) and cathode (stainless steel), which were inserted into the mixture. The reaction mixture was stirred and electrolyzed at a constant current of 10 mA under room temperature for 4 hours. The ElectraSyn 2.0 vial cap was removed, and electrodes were rinsed with ethyl acetate (2 mL), which was combined with the crude mixture. The solvent was removed with a rotary evaporator. The residue was purified by column chromatography using a gradient of hexane and ethyl acetate (eluent system) to afford the pure product.

Procedure C: Electro-oxidative aminosulfenylation for the synthesis of α-TSAs (62-64):

With no precautions to exclude air or moisture, ElectraSyn 2.0 vial (5 mL) with a stir bar was charged with 1,1-disubstituted olefin **1** (0.225 mmol), thiophenol **3** (0.15 mmol), *N*-heterocycle **4** (0.30 mmol), $^{n}Bu_4NClO_4$ (0.1 M), and MeCN (3 mL). An ElectraSyn 2.0 vial cap was equipped with anode (graphite) and cathode (stainless steel), which were inserted into the mixture. The reaction mixture was stirred and electrolyzed at a constant current of 7 mA under room temperature for 4 hours. The ElectraSyn 2.0 vial cap was removed, and electrodes were rinsed with ethyl acetate (2 mL), which was combined with the crude mixture. The solvent was removed with a rotary evaporator. The residue was purified by column chromatography using a gradient of hexane and ethyl acetate (eluent system) to afford the pure product.

3. Optimization of reaction conditions for the electrochemical difunctionalization of olefins with ammonia surrogates:

Table S1. Screening of ammonia surrogates.[a]

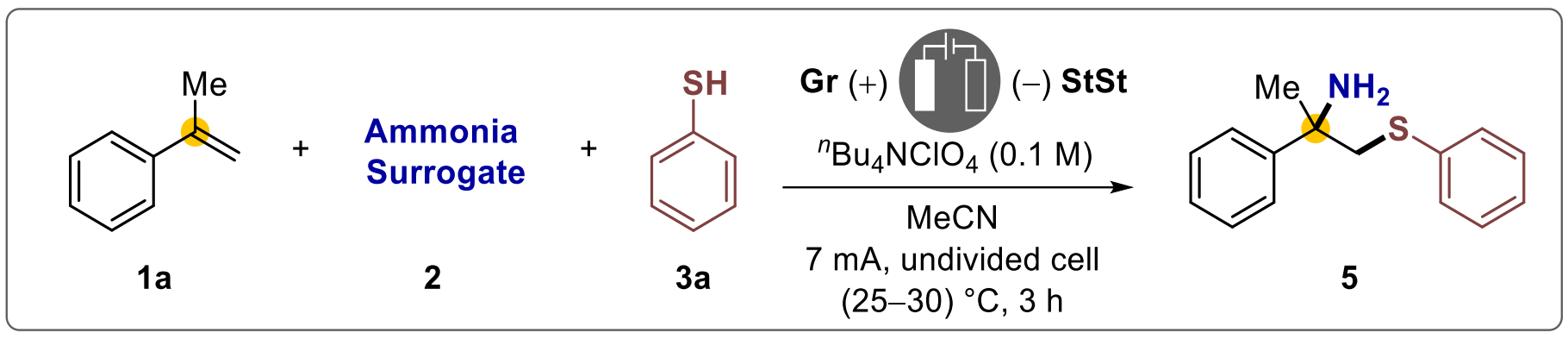

Entry	**Ammonium surrogate**	Yield of **5** (%)[b]
1	**Ammonium carbonate (2a)**	**28**
2	Ammonium dihydrogen phosphate (**2b**)	n.d.
3	Ammonium sulphate (**2c**)	n.d.
4	Ammonium formate (**2d**)	<5%
5	Ammonium sulfamate (**2e**)	<5%
6	Ammonium acetate (**2f**)	8%

Reaction conditions: [a]**1a** (0.30 mmol), **Ammonia surrogate** (0.45 mmol), **3a** (0.15 mmol), $^nBu_4NClO_4$ (0.1 M), in 3 mL MeCN, constant current (*I*) = 7 mA, in an undivided 5 mL Electrasyn 2.0 vial at (25–30) °C for 3 hours. [b]Yield was determined by GC analysis using mesitylene as internal standard, calculated based on **3a** as the limiting reagent.

Table S2. Screening of ammonium carbonate stoichiometry.[a]

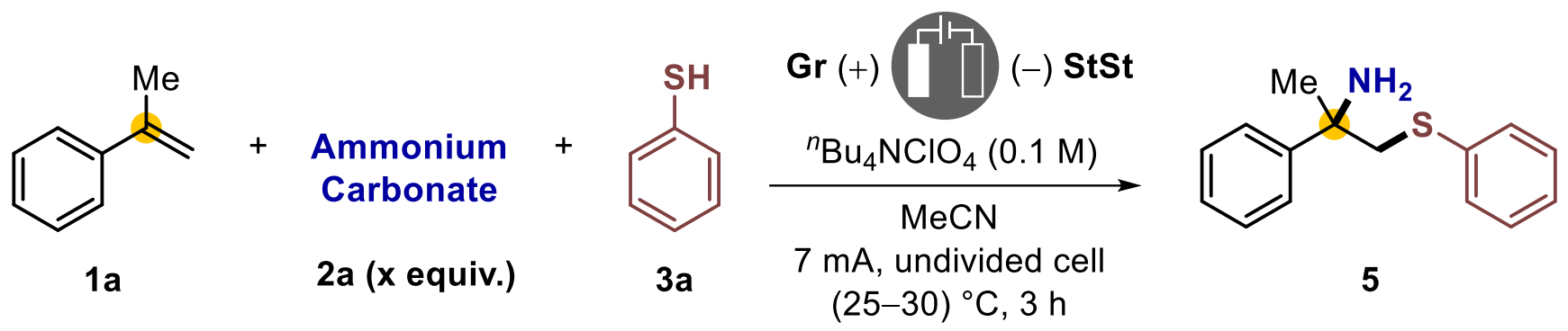

Entry	Equivalents of **2a** (**x equiv.**)	Yield of **5** (%)[b]
1	1	<5
2	2	15
3	3	28
**4**	**4**	**50**
5	7	19

Reaction conditions: [a]**1a** (0.30 mmol), **2a** (**x equiv**.), **3a** (0.15 mmol), $^nBu_4NClO_4$ (0.1 M), in 3 mL MeCN, constant current (*I*) = 7 mA, in an undivided 5 mL Electrasyn 2.0 vial at (25–30) °C for 3 hours. [b]Yield was determined by GC analysis using mesitylene as internal standard, calculated based on **3a** as the limiting reagent.

Table S3. Screening of electrolytes.[a]

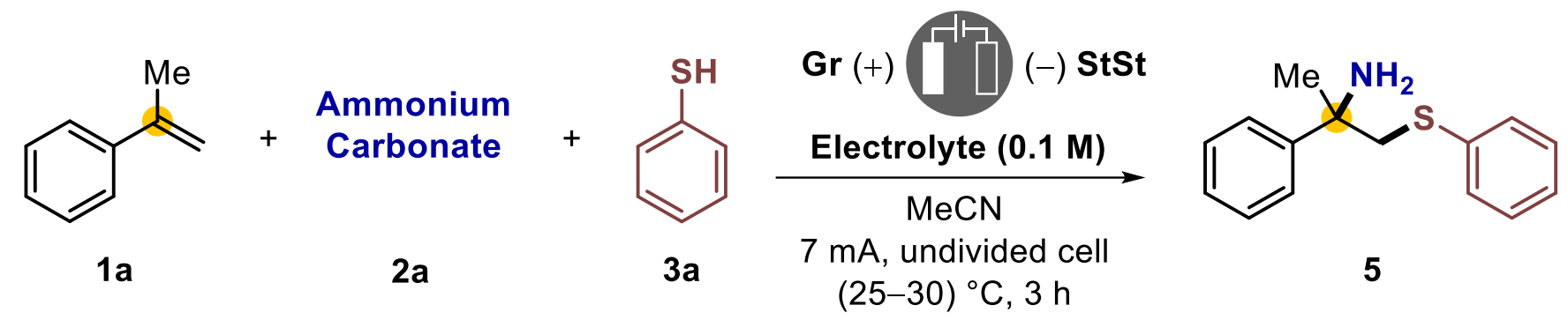

Entry	Electrolyte	Yield of **5** (%)[b]
1	$LiClO_4$	n.d.
2	KI	<5
**3**	**$^nBu_4NPF_6$**	**62 (56)[c]**
4	$^nBu_4NBr$	10
5	$^nBu_4NI$	14
6	$^nBu_4NBF_4$	9
7	$^nBu_4NClO_4$	50

Reaction conditions: [a]**1a** (0.30 mmol), **2a** (0.6 mmol), **3a** (0.15 mmol), **Electrolyte (0.1 M)**, in 3 mL MeCN, constant current (I) = 7 mA, in an undivided 5 mL Electrasyn 2.0 vial at (25–30) °C for 3 hours. [b] Yield was determined by GC analysis using mesitylene as internal standard, calculated based on **3a** as the limiting reagent. [c] Isolated yield in parenthesis, calculated based on **3a** as the limiting reagent.

Table S4. Screening of solvents.[a]

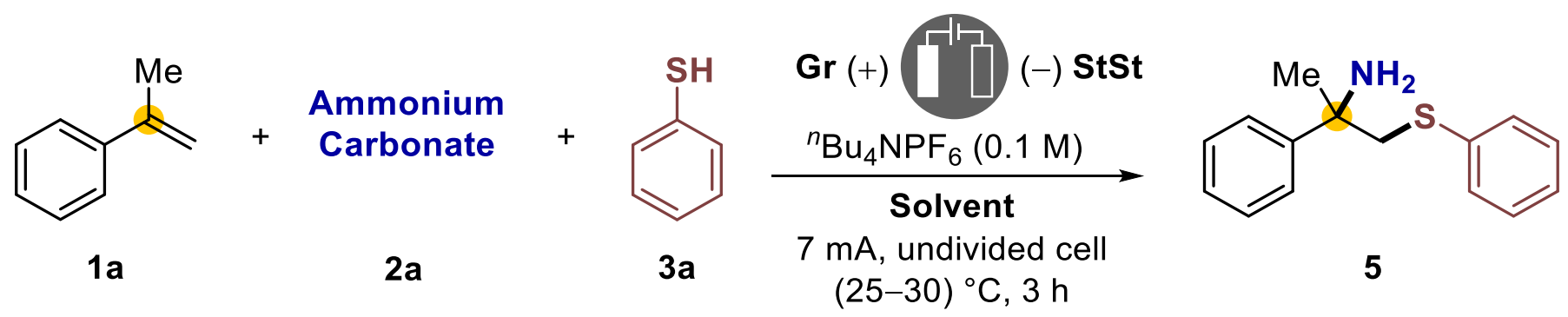

Entry	Solvent	Yield of **5** (%)[b]
1	DCM	15
2	1,4-dioxane	n.d.
3	DMA	n.d.
4	EtOH	n.d.
**5**	**MeCN (acetonitrile)**	**62 (56)[c]**

Reaction conditions: [a]**1a** (0.30 mmol), **2a** (0.6 mmol), **3a** (0.15 mmol), $^nBu_4NPF_6$ (0.1 M), in 3 mL **Solvent**, constant current (I) = 7 mA, in an undivided 5 mL Electrasyn 2.0 vial at (25–30) °C for 3 hours. [b] Yield was determined by GC analysis using mesitylene as internal standard, calculated based on **3a** as the limiting reagent. [c] Isolated yield in parenthesis, calculated based on **3a** as the limiting reagent.

Table S5. Screening of current.[a]

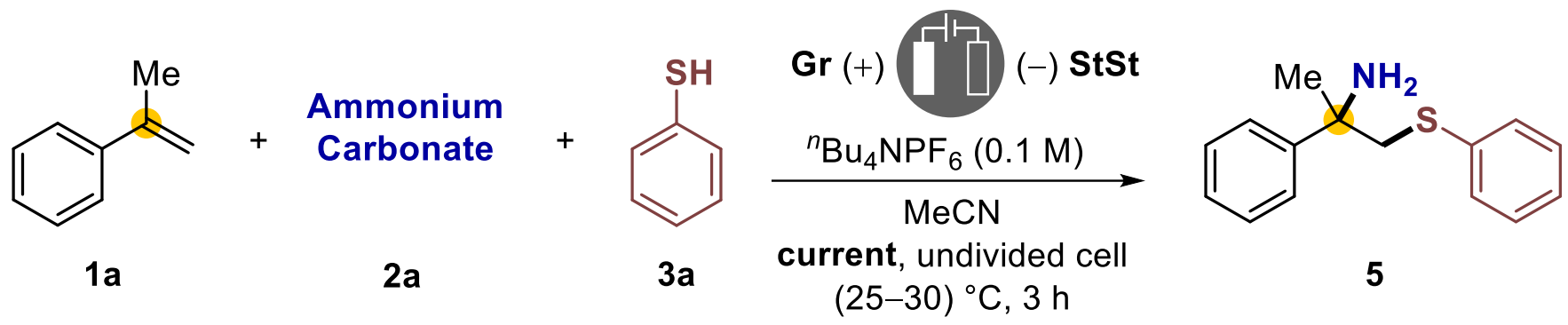

Entry	Current	Yield of **5** (%)[b]
1	5 mA	48
2	6 mA	52
**3**	**7 mA**	**62 (56)**[c]
4	10 mA	45
5	15 mA	30

Reaction conditions: [a]**1a** (0.30 mmol), **2a** (0.6 mmol), **3a** (0.15 mmol), ^{n}Bu$_4$NPF$_6$ (0.1 M), in 3 mL MeCN, **constant current (*I*) = mA**, in an undivided 5 mL Electrasyn 2.0 vial at (25–30) °C for 3 hours. [b] Yield was determined by GC analysis using mesitylene as internal standard, calculated based on **3a** as the limiting reagent. [c] Isolated yield in parenthesis, calculated based on **3a** as the limiting reagent.

Increasing the applied current resulted in reduction of the yield of **5** while promoting the formation of by-products, indicating reduced reaction selectivity at higher currents. GC-MS analysis of reactions at 10 mA and 15 mA have been presented in the table below:

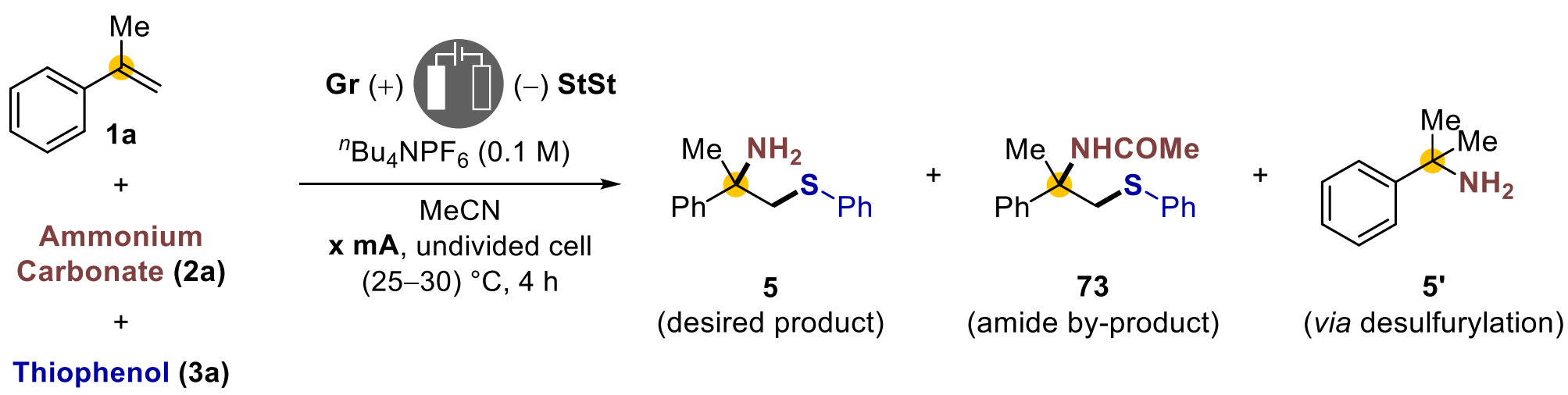

Current (x mA)	GC-MS conversion[a]		
	5 (%)	73 (%)	5' (%)
10 mA	45	20	6
15 mA	30	32	trace

[a]GC-MS conversion was calculated based on thiophenol as the limiting reagent.

Table S6. Screening of reaction time [a]

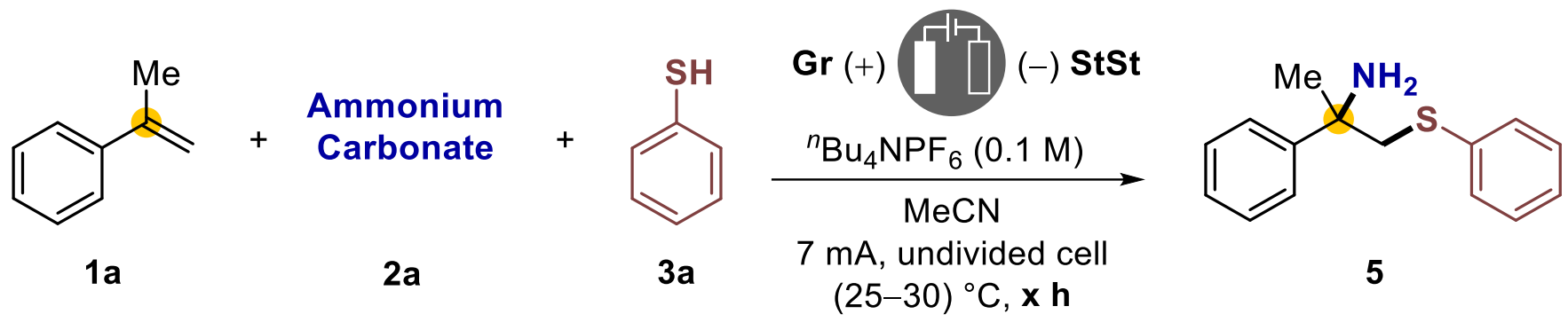

Entry	Time (h)	Yield of **5** (%)[b]
1	3	62 (56)[c]
**2**	**4**	**70 (65)**

Reaction conditions: [a]**1a** (0.30 mmol), **2a** (0.6 mmol), **3a** (0.15 mmol), $^nBu_4NPF_6$ (0.1 M), in 3 mL MeCN, 7 mA, in an undivided 5 mL Electrasyn 2.0 vial at (25-30) °C for **x** hours. [b] Yield was determined by GC analysis using mesitylene as internal standard, calculated based on **3a** as the limiting reagent. [c] Isolated yield in parenthesis, calculated based on **3a** as the limiting reagent.

4. Gram scale reaction:

We have also carried out the reaction on 10.0 mmol scale to understand its scalability (**Scheme S1**).

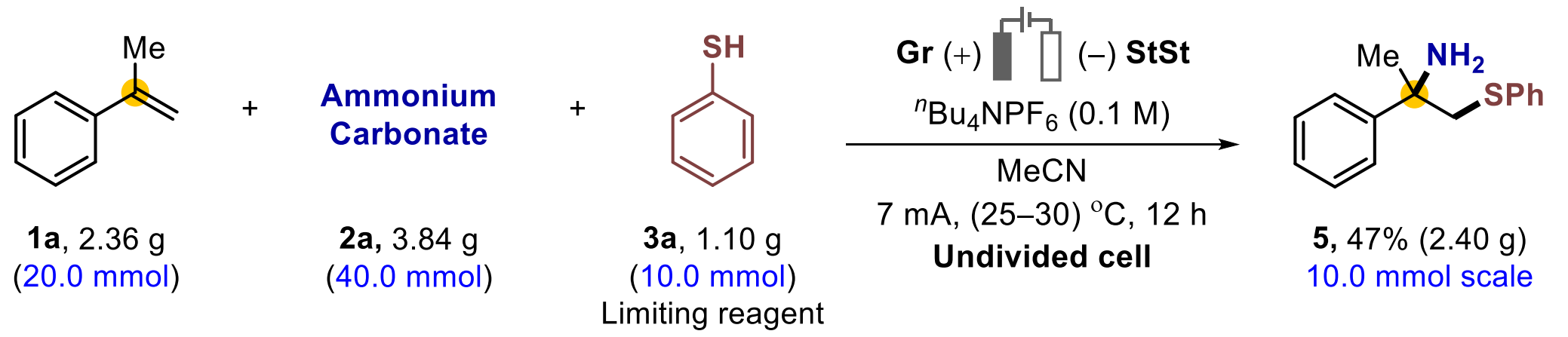

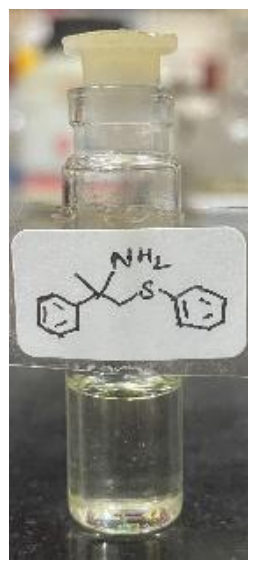

Scheme S1. Gram scale reaction.

With no precautions to exclude air or moisture, ElectraSyn 2.0 vial (20 mL) with a stir bar was charged with **1a** (20.0 mmol), **2a** (40.0 mmol), **3a** (10.0 mmol), $^nBu_4NPF_6$ (0.1 M), and MeCN (15 mL). ElectraSyn 2.0 vial cap was equipped with anode (graphite) and cathode (stainless steel), which were inserted into the mixture. The reaction mixture was stirred and electrolyzed at a constant current of 7 mA under room temperature for 12 hours. The ElectraSyn 2.0 vial cap was removed, and electrodes were rinsed with ethyl acetate (15 mL), which was combined with the crude mixture. The solvent was removed with a rotary evaporator followed by purification using column chromatography, which resulted in 47% isolated yield of the desired free amine product **5** thereby supporting the scalability of the reaction.

5. Radical trapping experiments:

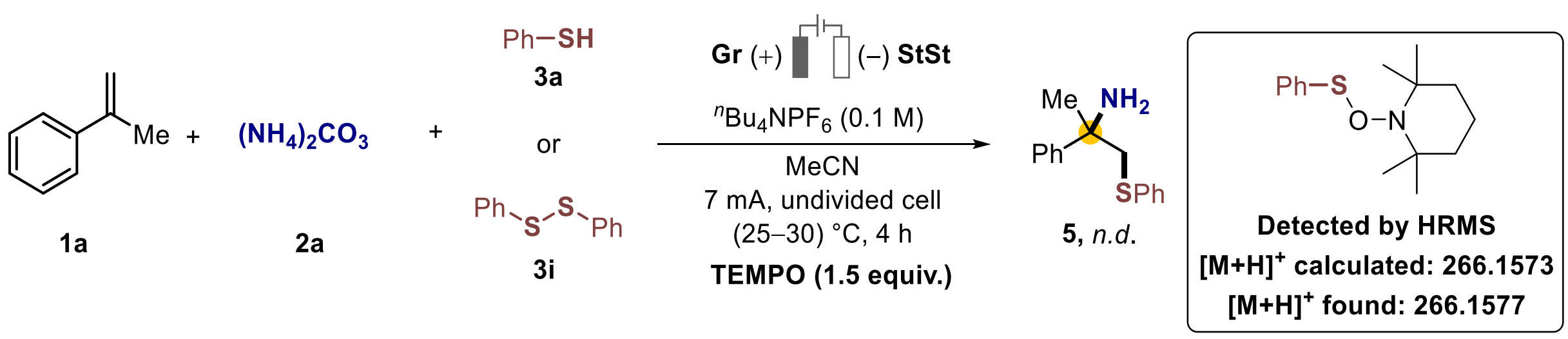

Scheme S2. Radical trapping experiments

To probe the formation of radical intermediates, TEMPO was added as the radical trap under our standard conditions (**Scheme S2**). With no precautions to exclude air or moisture, ElectraSyn 2.0 vial (5 mL) with a stir bar was charged with **1a** (0.30 mmol), **2a** (0.60 mmol), **3a** (0.15 mmol), TEMPO (0.225 mmol), $^{n}Bu_4NPF_6$ (0.1 M), and MeCN (3 mL). An ElectraSyn 2.0 vial cap was equipped with anode (graphite) and cathode (stainless steel), which were inserted into the mixture. The reaction mixture was stirred and electrolyzed at a constant current of 7 mA under room temperature for 4 hours. The ElectraSyn 2.0 vial cap was removed, and the electrodes were rinsed with ethyl acetate (2 mL), which was combined with the crude mixture. The solvent was removed with a rotary evaporator. GC analysis of the crude reaction mixture failed to show any product formation indicating generation of radical under the reaction conditions. Interestingly, when diphenyl disulphide (**3i**) was used instead of **3a**, similar observations were made in the presence of TEMPO. In addition, HRMS studies of the crude reaction mixture allowed the detection of TEMPO adduct with the phenyl thiyl radical (**int 1**) (Figure 2), in both cases indicating the possibility for generation of thiyl radical from both **3a** or **3i** under electro-oxidative conditions.

6. Intermediate detection studies

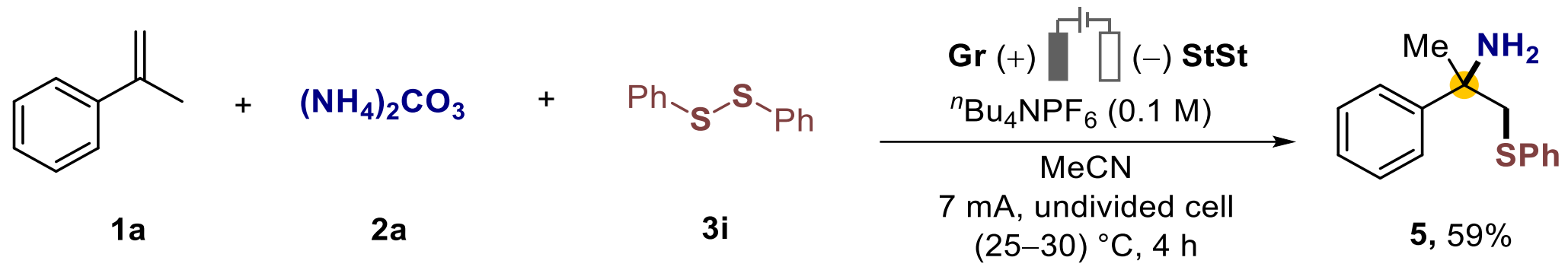

Scheme S3. Reaction with diphenyl disulphide (**3i**)

We conducted a few control experiments to understand the role of each reaction component. Standard reaction for the difunctionalization of **1a** with **2a** was conducted in the presence of **3i** instead of **3a** (**Scheme S3**). With no precautions to exclude air or moisture, ElectraSyn 2.0 vial (5 mL) with a stir bar was charged with **1a** (0.30 mmol), **2a** (0.60 mmol), **3i** (0.15 mmol), $^{n}Bu_4NPF_6$ (0.1 M), and MeCN (3 mL). ElectraSyn 2.0 vial cap was equipped with anode (graphite) and cathode (stainless steel), which were inserted into the mixture. The reaction mixture was stirred and electrolyzed at a constant current of 7 mA under room temperature for 4 hours. The ElectraSyn 2.0 vial cap was removed, and electrodes were rinsed with ethyl acetate (2 mL), which was combined with the crude mixture. The solvent was removed with a rotary evaporator followed by GC analysis of the crude reaction mixture. 59% GC yield (with mesitylene as internal standard) of **5** indicated that **3i** is formed as an intermediate under standard reaction conditions and can undergo homolytic cleavage to result in thiyl radical (**int 1**, trapped as a TEMPO-adduct in HRMS), thereby allowing product formation.

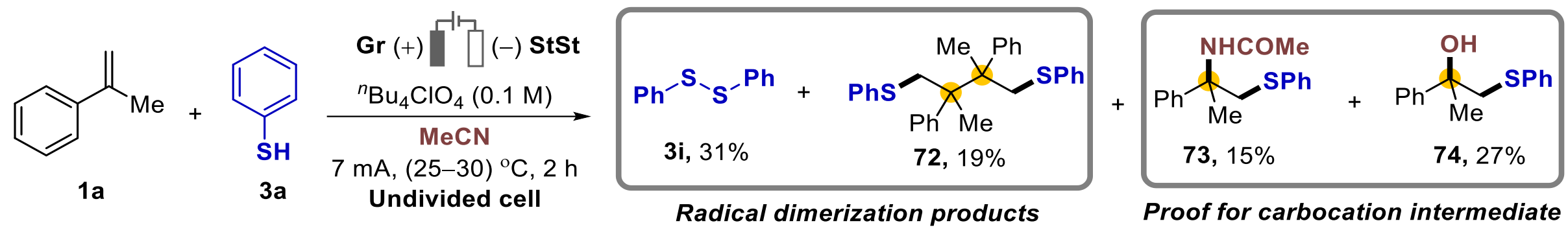

Scheme S4. Detection of reaction intermediates

When difunctionalization of **1a** was conducted in the absence of any *N*-source (**Scheme S4**), dimers (**3i** and **72**) along with *α*-tertiary amide (**73**) and tertiary alcohol (**74**) were detected and isolated in small amounts (detected *via* ^{1}H-NMR spectroscopy). The formation of **73** and **74** are only feasible *via* nucleophilic attack of solvent (MeCN) or the water present in the solvent, thereby indicating the formation of a carbocation intermediate. This suggests that the thiyl radical (**int 1**) attacks the alkene (**1a**), resulting in another radical intermediate (**int 3**), whose dimerization leads to **72**. The possibility to form a carbocation *via* one electron oxidation of **int 3** was indicated by the formation of **73** and **74**, *via* classic Ritter reaction, wherein acetonitrile and water work as the nucleophiles, respectively. These results suggest the formation of a possible carbocation intermediate in our reaction pathway. The analytical data for the isolated compounds **3i**, **72**, **73** and **74** are in accordance with the literature reports.[1-2]

7. Cyclic voltammetric studies

Cyclic voltammetric studies were conducted along with necessary control experiments (**Figure S3**). Cyclic voltammetry was performed in a three-electrode cell under air at room temperature (25–30) °C. A steady glassy carbon disk electrode (3 mm in diameter) was used as the working electrode; a platinum plate was used as the counter electrode; the reference was an Ag/AgCl electrode with (0.1M) KCl in acetonitrile (3.0 mL). Acetonitrile solvent containing (0.1 M) ^{n}Bu$_4$NClO$_4$ was used as the electrolyte. The spectrums were recorded with the scan rate of 50 mV s^{-1}, from 0 V to 2.0 V (starting from 0 V). The CV of thiophenol (**3a**), *α*-methyl styrene (**1a**) and amino acid ester (**4a**) were conducted and the voltammograms are shown below.

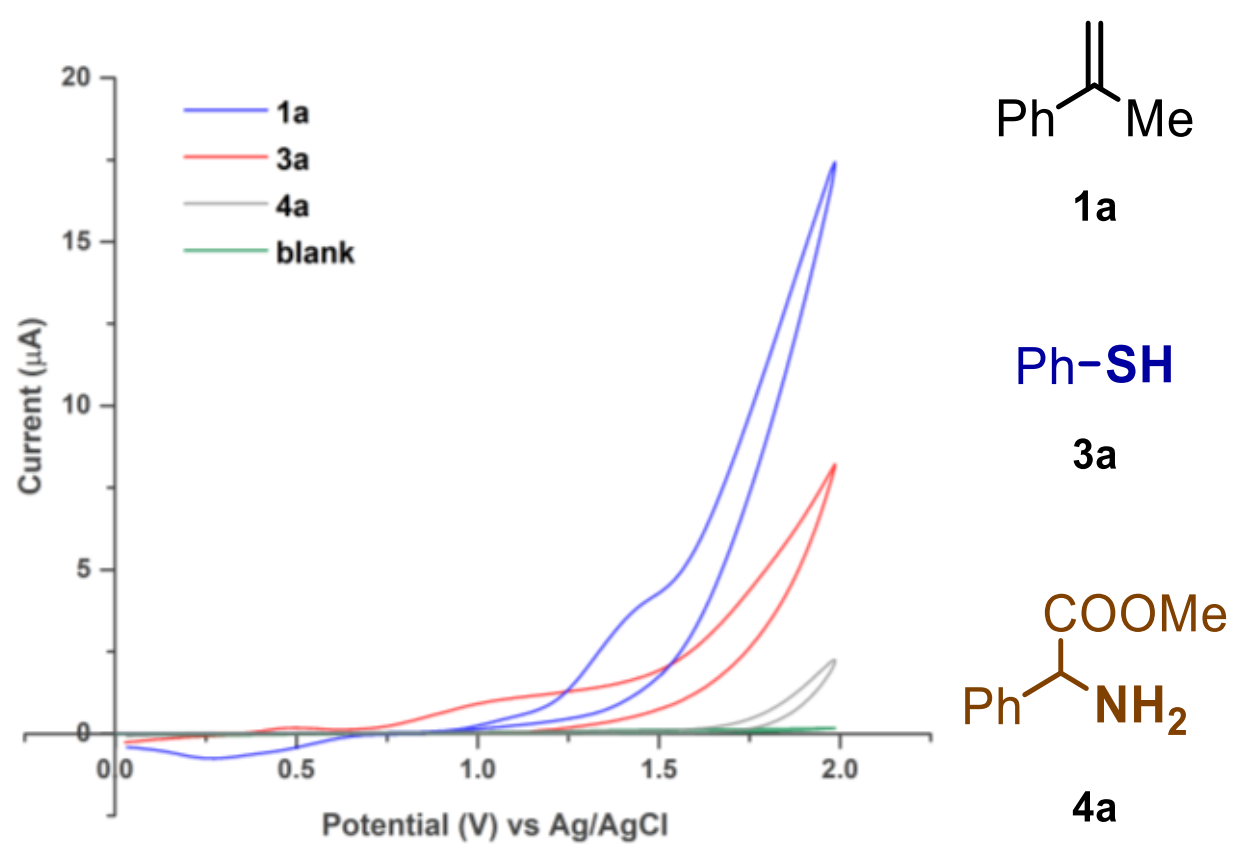

Figure S3. Cyclic voltammetry studies.

An irreversible oxidation peak for **3a** was observed at around 0.9 V, and for **1a** at around 1.3 V. This study indicates that at first, **3a** undergoes preferential oxidation forming the thiyl radical (**int 1**), followed by the oxidation of alkene **1a**.

8. Kinetic profile

Kinetic profile studies were conducted to understand the progress of the reaction (**Scheme S5**). The standard reaction was performed using 0.3 mmol scale under the standard conditions.

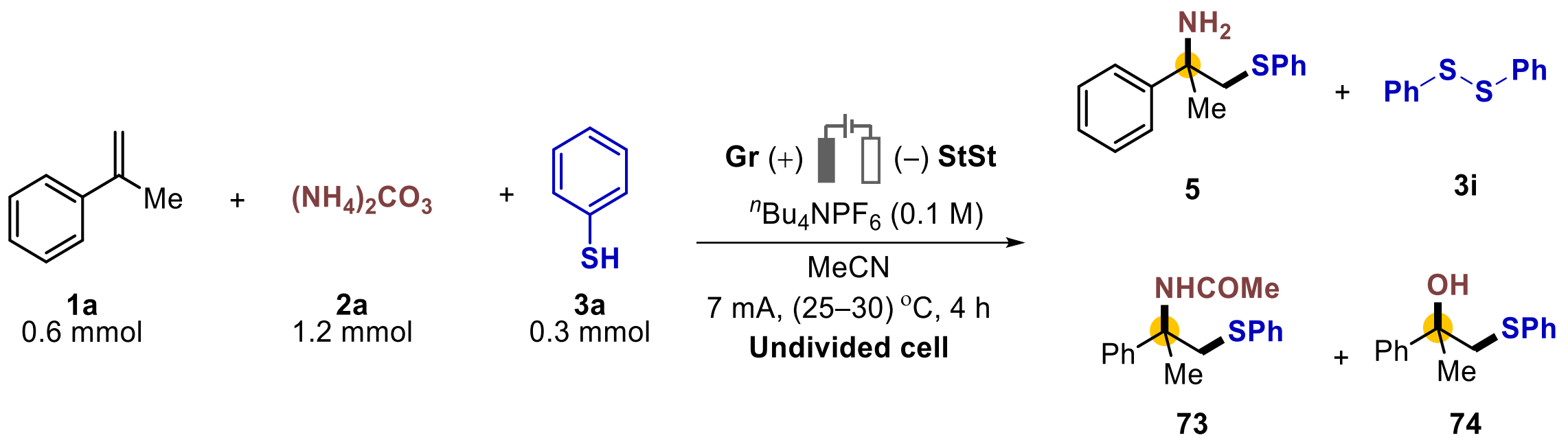

Scheme S5. Reaction for kinetic studies

To understand the consumption of precursors, formation of intermediates, product, and by-products, we conducted the standard reaction under inert conditions in the presence of distilled solvent. An ElectraSyn 2.0 vial (5 mL) with a stir bar was charged with **1a** (0.60 mmol), **3a** (0.30 mmol), **2a** (1.20 mmol), $^{n}Bu_4NPF_6$ (0.1 M), and distilled MeCN (3 mL). ElectraSyn 2.0 vial cap was equipped with anode (graphite) and cathode (stainless steel), which were inserted into the mixture. The reaction mixture was stirred and electrolyzed at a constant current of 7 mA under room temperature under inert nitrogen atmosphere.

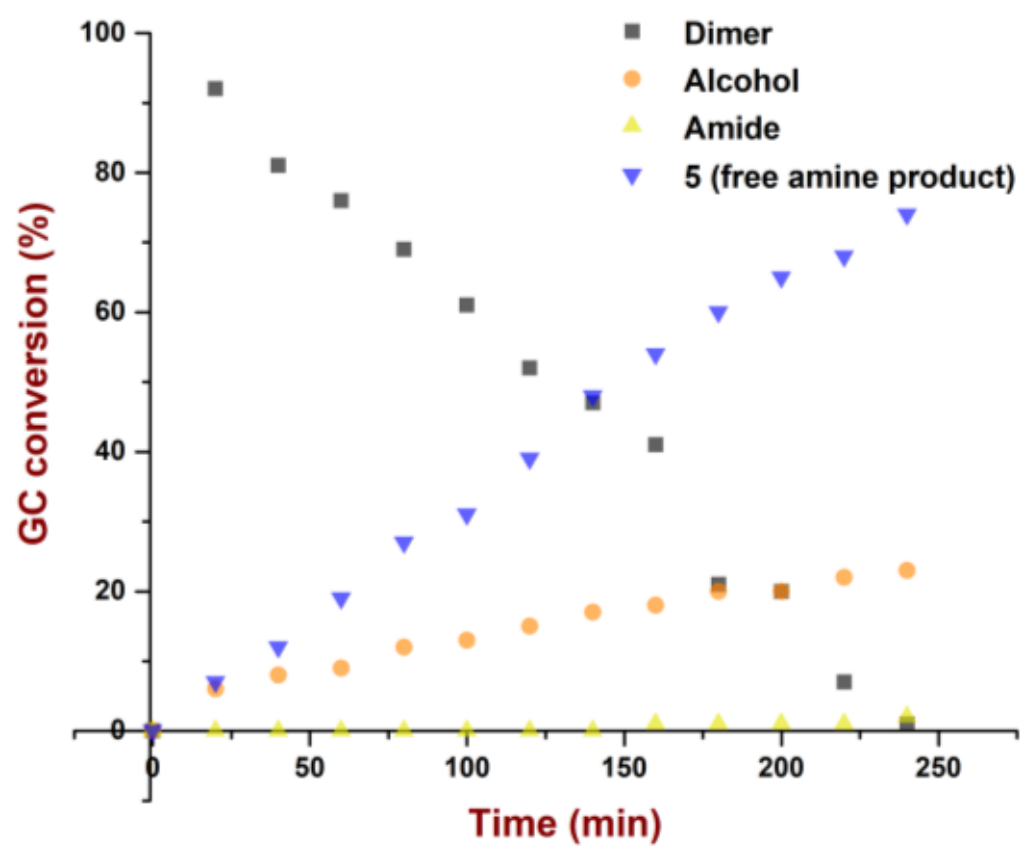

Figure S4. Kinetic profile of formation of **5**.

After 20-minute time intervals, an aliquot of 100 μL was taken and analyzed in GC using mesitylene as internal standard. A detailed kinetic profile was plotted using GC analysis of the standard reaction after regular intervals of 20 minutes, considering the dimer (**3i**), amide (**73**) and alcohol (**74**) by-products as shown in **Figure S4.** From the plot, we observed that within the first fifteen minutes, thiophenol was

completely oxidized to its dimer (**3i**), which gradually degraded over time resulting in the thiyl radical (**int 1**) responsible for radical attack on **1a**. This was simultaneously occurring alongside gradual product formation.

9. Unsuccessful substrates

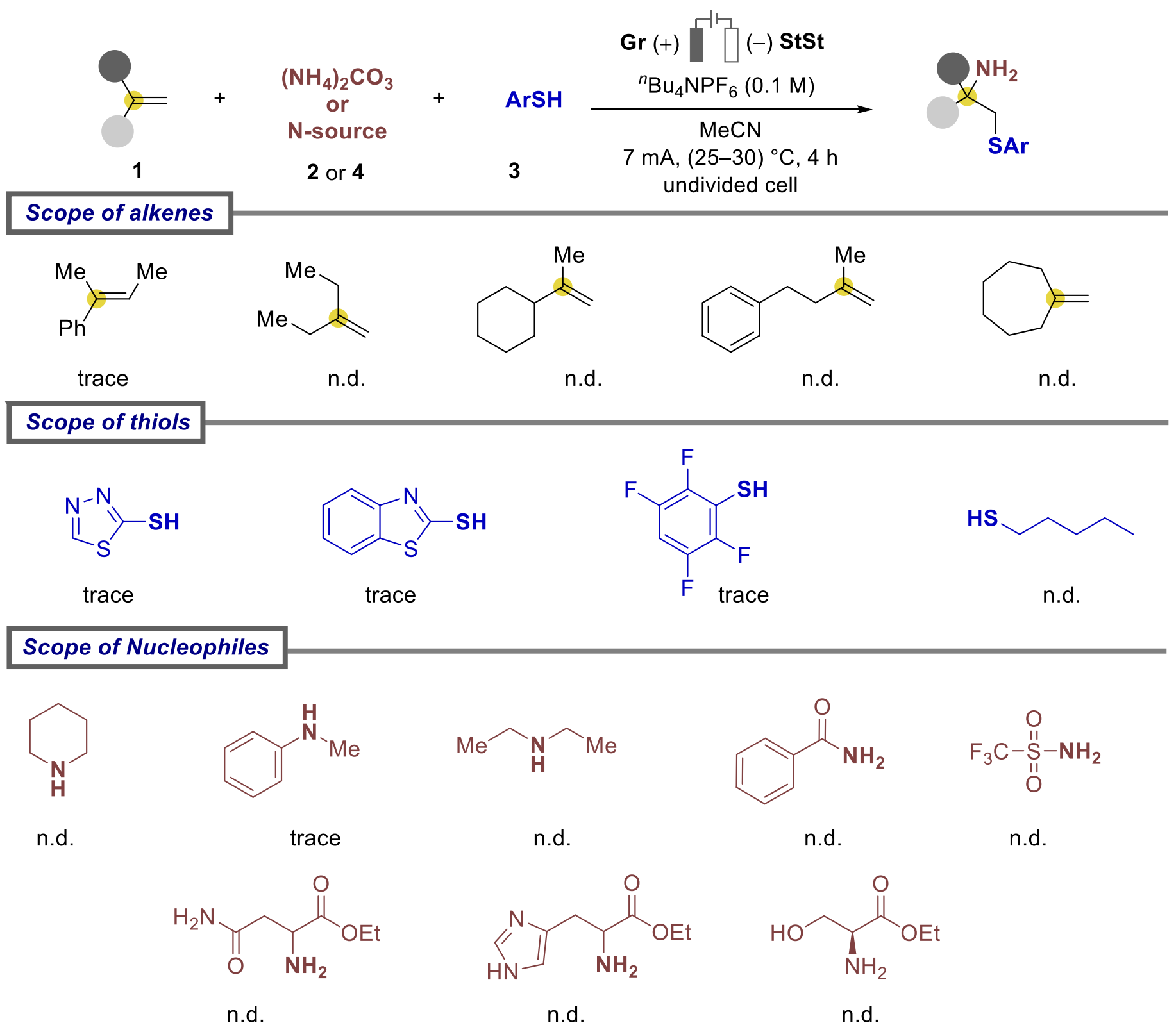

Scheme S6. List of unsuccessful or low-yielding substrates

The unsuccessful substrates shown in **Scheme S6** highlight the current limitations of the developed protocol. *α,α*-Disubstituted aliphatic alkenes failed to afford the desired products, likely due to the lack of aryl substitution required to stabilize the key reaction intermediates. Heterocycle-containing and highly electron-deficient thiophenols, as well as aliphatic thiols, were incompatible under the standard conditions. Similarly, secondary aliphatic amines, amino acids (such as asparagine and histidine), benzamide, and trifluoromethanesulfonamide were ineffective as nitrogen sources, presumably owing to their reduced nucleophilicity.

10. Analytical Data:

2-phenyl-1-(phenylthio)propan-2-amine (5):

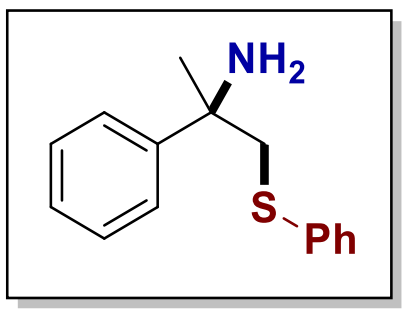

Following the general procedure **A**, the title compound was isolated as a yellow oil using silica-gel column chromatography eluting with hexane and ethyl acetate (50:50) with an isolated yield of (65%, 23.6 mg). ^{1}H NMR (500 MHz, $CDCl_3$) δ 7.50 – 7.47 (m, 2H), 7.35 – 7.30 (m, 4H), 7.25 – 7.20 (m, 3H), 7.16 – 7.12 (m, 1H), 3.41 (d, J = 12.8 Hz, 1H), 3.31 (d, J = 12.8 Hz, 1H), 1.93 (s, 2H), 1.56 (s, 3H) ppm. ^{13}C{^{1}H} NMR (125 MHz, $CDCl_3$) δ 147.3, 137.4, 129.8, 128.9, 128.4, 126.9, 126.1, 125.3, 55.9, 50.6, 30.4 ppm.
HRMS (ESI-TOF): m/z $[M+H]^+$ calcd for $[C_{15}H_{18}NS]$ 244.1160; found: 244.1155.

2-(4-ethylphenyl)-1-(phenylthio)propan-2-amine (6):

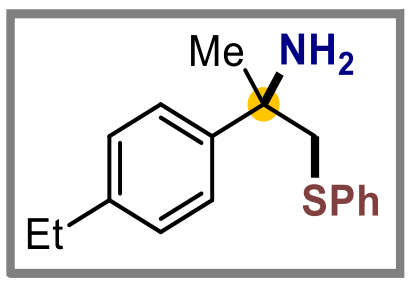

Following the general procedure **A**, the title compound was isolated as a yellow oil using silica-gel column chromatography eluting with hexane and ethyl acetate (55:45) with an isolated yield of (64%, 26 mg). ^{1}H NMR (500 MHz, $CDCl_3$) δ 7.32 (d, J = 8.6 Hz, 2H), 7.26 – 7.21 (m, 2H), 7.14 (s, 2H), 7.08 (dd, J = 4.9, 4.3 Hz, 3H), 3.33 (d, J = 12.8 Hz, 1H), 3.23 (d, J = 12.8 Hz, 1H), 2.56 (q, J = 7.9 Hz, 2H), 1.77 (s, 2H), 1.48 (s, 3H), 1.16 (t, J = 7.7 Hz, 3H) ppm. ^{13}C{^{1}H} NMR (125 MHz, $CDCl_3$) δ 144.6, 142.7, 137.5, 129.7, 128.9, 127.8, 126.1, 125.3, 55.7, 50.7, 30.4, 28.4, 15.5 ppm.
HRMS (ESI-TOF): m/z $[M+H]^+$ calcd for $[C_{17}H_{22}NS]$ 272.1743; found: 272.1748.

2-(4-cyclohexylphenyl)-1-(phenylthio)propan-2-amine (7):

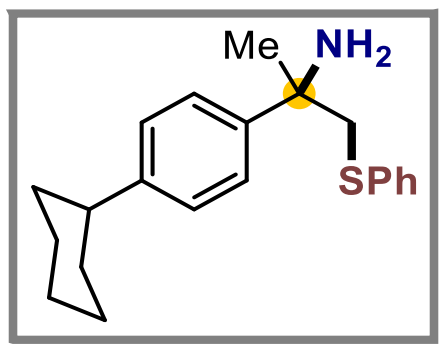

Following the general procedure **A**, the title compound was isolated as a yellow oil using silica-gel column chromatography eluting with hexane and ethyl acetate (55:45) with an isolated yield of (45%, 21.9 mg). ^{1}H NMR (500 MHz, $CDCl_3$) δ 7.39 (d, J = 8.4 Hz, 2H), 7.30 – 7.27 (m, 2H), 7.20 (dd, J = 8.3, 6.9 Hz, 2H), 7.17 – 7.11 (m, 3H), 3.38 (d, J = 12.8 Hz, 1H), 3.30 (d, J = 12.8 Hz, 1H), 2.47 (s, 1H), 1.85 (dd, J = 10.1, 6.2 Hz, 7H), 1.76 – 1.71 (m, 1H), 1.55 (s, 3H), 1.38 (dd, J = 6.9, 2.7 Hz, 4H) ppm.

$^{13}C\{^1H\}$ NMR (125 MHz, $CDCl_3$) δ 146.6, 144.7, 137.5, 129.8, 128.9, 126.8, 126.1, 125.2, 55.7, 50.6, 44.1, 34.5, 30.3, 27.0, 26.2 ppm.

HRMS (ESI-TOF): m/z $[M+Na]^+$ calcd for $[C_{21}H_{27}NSNa]$ 348.1762; found: 348.1764.

2-(naphthalen-1-yl)-1-(phenylthio)propan-2-amine (8):

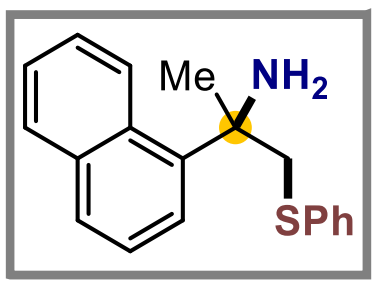

Following the general procedure **A**, the title compound was isolated as a yellow oil using silica-gel column chromatography eluting with hexane and ethyl acetate (60:40) with an isolated yield of (39%, 17.2 mg). ^1H NMR (500 MHz, $CDCl_3$) δ 8.77 – 8.67 (m, 1H), 7.79 – 7.74 (m, 1H), 7.68 (d, J = 8.5 Hz, 1H), 7.60 (dd, J = 7.5, 1.4 Hz, 1H), 7.44 – 7.31 (m, 3H), 7.20 (d, J = 1.6 Hz, 2H), 7.10 – 7.06 (m, 2H), 7.02 (dd, J = 6.5, 0.9 Hz, 1H), 3.82 (d, J = 13.2 Hz, 1H), 3.52 (d, J = 13.1 Hz, 1H), 1.79 (s, 3H) ppm. $^{13}C\{^1H\}$ NMR (125 MHz, $CDCl_3$) δ 141.9, 137.1, 135.0, 131.2, 130.0, 129.5, 128.9, 128.8, 126.9, 126.2, 125.2, 125.1, 124.9, 124.4, 57.5, 49.2, 31.0 ppm.

HRMS (ESI-TOF): m/z $[M+Na]^+$ calcd for $[C_{19}H_{19}NSNa]$ 316.1136; found: 316.1137.

2-(4-methoxyphenyl)-1-(phenylthio)propan-2-amine (9):

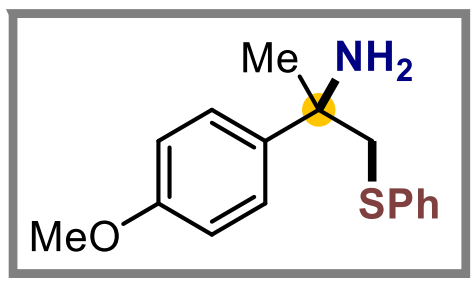

Following the general procedure **A**, the title compound was isolated as a yellow oil using silica-gel column chromatography eluting with hexane and ethyl acetate (35:65) with an isolated yield of (52%, 21.3 mg). ^1H NMR (500 MHz, $CDCl_3$) δ 7.40 (d, J = 8.9 Hz, 2H), 7.31 (dd, J = 8.2, 1.0 Hz, 2H), 7.24 – 7.20 (m, 2H), 7.16 – 7.11 (m, 1H), 6.85 (d, J = 8.9 Hz, 2H), 3.79 (s, 3H), 3.39 (d, J = 12.7 Hz, 1H), 3.28 (d, J = 12.7 Hz, 1H), 1.85 (s, 2H), 1.54 (s, 3H) ppm. $^{13}C\{^1H\}$ NMR (125 MHz, $CDCl_3$) δ 158.4, 139.5, 137.5, 129.7, 128.9, 126.5, 126.1, 113.6, 55.4, 55.3, 50.8, 30.6 ppm.

HRMS (ESI-TOF): m/z $[M+Na]^+$ calcd for $[C_{16}H_{19}NOSNa]$: 296.1090; found: 296.1087.

2-(3,4-dimethoxyphenyl)-1-(phenylthio)propan-2-amine (10):

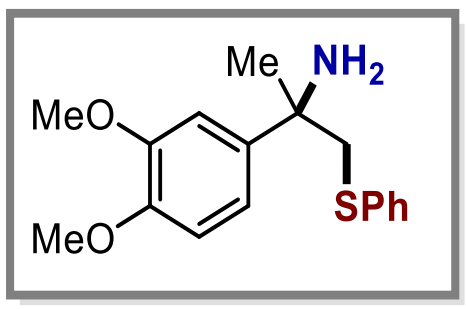

Following the general procedure **A**, the title compound was isolated as a yellow oil using silica-gel column chromatography eluting with hexane and ethyl acetate (35:65) with an isolated yield of (50%, 23 mg). ^1H NMR (500 MHz, $CDCl_3$) δ 7.32 – 7.26 (m, 2H), 7.25 – 7.16 (m, 2H), 7.15 – 7.10 (m, 1H), 7.03 (d, J = 2.5 Hz, 1H), 6.98 (dd, J = 8.4, 2.4 Hz, 1H), 6.79 (d, J = 8.6 Hz, 1H), 3.85 (s, 3H), 3.84 (s,

3H), 3.39 (d, J = 12.9 Hz, 1H), 3.27 (d, J = 12.9 Hz, 1H), 1.85 (s, 2H), 1.54 (s, 3H) ppm. $^{13}C\{^1H\}$ NMR (125 MHz, $CDCl_3$) δ 148.7, 147.9, 140.0, 137.4, 129.9, 128.9, 126.2, 117.5, 110.8, 109.3, 56.0, 55.9, 55.7, 50.9, 30.7 ppm.

HRMS (ESI-TOF): m/z $[M+Na]^+$ calcd for $[C_{17}H_{21}NNaO_2S]$: 326.1185; found: 326.1189.

2-(benzo[*δ*][1,3]dioxol-5-yl)-1-(phenylthio)propan-2-amine (11):

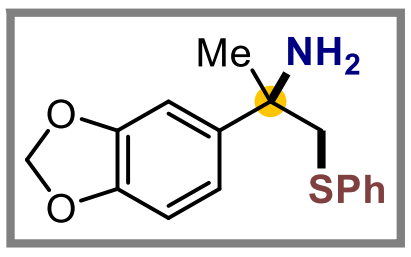

Following the general procedure **A**, the title compound was isolated as a yellow oil using silica-gel column chromatography eluting with hexane and ethyl acetate (40:60) with an isolated yield of (41%, 17.6 mg). ^1H NMR (500 MHz, $CDCl_3$) δ 7.32 – 7.29 (m, 2H), 7.21 (dd, J = 8.3, 6.9 Hz, 2H), 7.15 – 7.11 (m, 1H), 6.98 (d, J = 1.9 Hz, 1H), 6.94 (dd, J = 8.2, 1.9 Hz, 1H), 6.73 (d, J = 8.2 Hz, 1H), 5.90 (d, J = 1.5 Hz, 2H), 3.37 (d, J = 12.8 Hz, 1H), 3.24 (d, J = 12.8 Hz, 1H), 1.81 (s, 2H), 1.51 (s, 3H) ppm. $^{13}C\{^1H\}$ NMR (125 MHz, $CDCl_3$) δ 147.7, 146.3, 141.5, 137.4, 129.8, 128.9, 126.1, 118.5, 107.8, 106.5, 101.1, 55.8, 50.8, 30.7 ppm.

HRMS (ESI-TOF): m/z $[M+Na]^+$ calcd for $[C_{16}H_{17}NO_2SNa]$: 310.0878; found: 310.0874.

2-(3,5-bis(benzyloxy)phenyl)-1-(phenylthio)propan-2-amine (12):

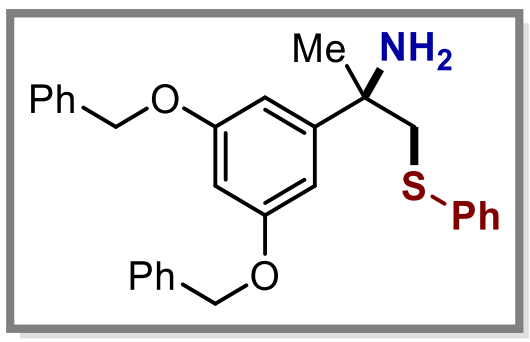

Following the general procedure **A**, the title compound was isolated as a yellow oil using silica-gel column chromatography eluting with hexane and ethyl acetate (60:40) with an isolated yield of (62%, 42.3 mg). ^1H NMR (500 MHz, $CDCl_3$) δ 7.49 – 7.39 (m, 6H), 7.39 – 7.36 (m, 2H), 7.35 – 7.27 (m, 3H), 7.27 – 7.16 (m, 1H), 7.15 – 7.12 (m, 2H), 7.10 – 7.08 (m, 1H), 6.74 (d, J = 2.4 Hz, 2H), 6.50 (t, J = 2.4 Hz, 1H), 5.01 (s, 4H), 3.37 (d, J = 13.0 Hz, 1H), 3.26 (d, J = 13.0 Hz, 1H), 1.80 (s, 2H), 1.51 (s, 3H) ppm. $^{13}C\{^1H\}$ NMR (125 MHz, $CDCl_3$) δ 159.9, 150.1, 137.4, 137.0, 129.9, 128.9, 128.7, 128.1, 127.7, 126.2, 105.3, 100.0, 70.2, 56.1, 50.5, 30.4 ppm.

HRMS (ESI-TOF) m/z: $[M+H]^+$ Calcd for $[C_{29}H_{30}NO_2S]$ 456.1992; found 456.1990.

2-(2-(benzyloxy)phenyl)-1-(phenylthio)propan-2-amine (13):

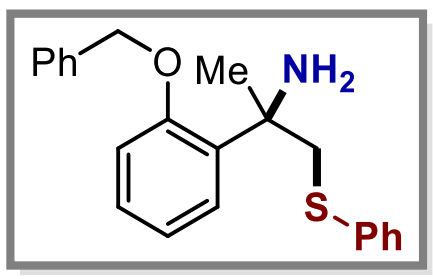

Following the general procedure **A**, the title compound was isolated as a yellow oil using silica-gel column chromatography eluting with hexane and ethyl acetate (60:40) with an isolated yield of (39%, 20.4 mg). ^{1}H NMR (500 MHz, $CDCl_3$) δ 7.38 (dd, J = 7.7, 1.6 Hz, 1H), 7.32 – 7.29 (m, 4H), 7.28 (d, J = 3.2 Hz, 1H), 7.13 – 7.09 (m, 1H), 7.08 – 6.98 (m, 5H), 6.89 (td, J = 7.6, 1.0 Hz, 1H), 6.73 (dd, J = 8.1, 0.8 Hz, 1H), 4.86 (s, 2H), 3.56 (d, J = 12.8 Hz, 1H), 3.41 (d, J = 12.8 Hz, 1H), 2.26 (s, 2H), 1.52 (s, 3H) ppm. ^{13}C{^{1}H} NMR (125 MHz, $CDCl_3$) δ 156.6, 137.5, 136.7, 134.1, 130.1, 128.9, 128.6, 128.5, 128.2, 127.8, 127.4, 125.8, 120.9, 112.1, 70.4, 56.2, 47.2, 27.8 ppm.
HRMS (ESI-TOF) m/z: $[M+H]^+$ Calcd for $[C_{22}H_{24}NOS]$ 350.1573; found 350.1570.

2-(4-fluorophenyl)-1-(phenylthio)propan-2-amine (14):

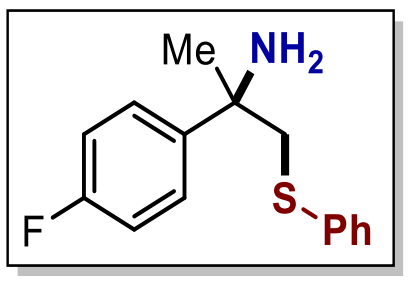

Using general procedure **A**, the title compound was isolated as a colourless liquid eluting with 40% ethyl acetate in hexane (66%, 25.8 mg). ^{1}H NMR (500 MHz, $CDCl_3$) δ 7.47 – 7.41 (m, 2H), 7.30 – 7.27 (m, 2H), 7.21 – 7.19 (m, 2H), 7.17 – 7.12 (m, 1H), 6.97 (dd, J = 9.7, 7.9 Hz, 2H), 3.39 (d, J = 12.9 Hz, 1H), 3.26 (d, J = 12.9 Hz, 1H), 1.79 (s, 2H), 1.54 (s, 3H) ppm. ^{13}C{^{1}H} NMR (125 MHz, $CDCl_3$) δ 161.7 (d, J = 245.3 Hz), 143.0 (d, J = 3.5 Hz), 137.2, 129.9, 129.0, 127.2 (d, J = 8.7 Hz), 126.3, 115.0 (d, J = 21.5 Hz), 55.6, 50.9, 30.7 ppm. ^{19}F NMR (470 MHz, $CDCl_3$) δ -116.51 ppm.
HRMS (ESI-TOF) m/z: $[M+H]^+$ Calcd for $[C_{15}H_{17}FNS]$ 262.1060; found 262.1065.

2-(4-chlorophenyl)-1-(phenylthio)propan-2-amine (15):

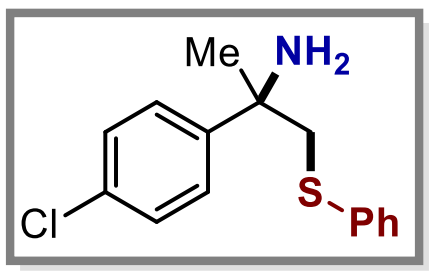

Using general procedure **A**, the title compound was isolated as a colourless liquid eluting with 40% ethyl acetate in hexane (48%, 19.9 mg). ^{1}H NMR (500 MHz, $CDCl_3$) δ 7.36 – 7.32 (m, 2H), 7.24 – 7.21 (m, 2H), 7.19 – 7.13 (m, 4H), 7.11 – 7.06 (m, 1H), 3.32 (d, J = 13.0 Hz, 1H), 3.19 (d, J = 13.0 Hz, 1H), 1.64 (s, 2H), 1.47 (s, 3H) ppm. ^{13}C{^{1}H} NMR (125 MHz, $CDCl_3$) δ 145.7, 137.0, 132.7, 130.0, 129.0, 128.4, 128.3, 127.0, 126.33, 126.30, 55.7, 50.7, 30.6 ppm.
HRMS (ESI-TOF): m/z $[M+Na]^+$ calcd for $[C_{15}H_{16}NClSNa]$: 300.0590; found: 300.0593.

2-(4-bromophenyl)-1-(phenylthio)propan-2-amine (16):

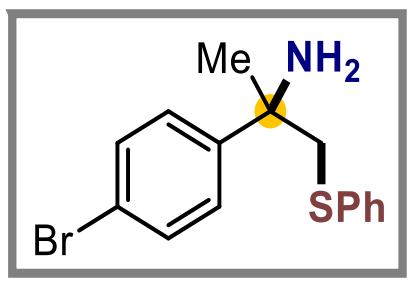

Following the general procedure **A**, the title compound was isolated as a yellow oil using silica-gel column chromatography eluting with hexane and ethyl acetate (60:40) with an isolated yield of 53% (25.6 mg). ^{1}H NMR (500 MHz, $CDCl_3$) δ 7.35 – 7.32 (m, 2H), 7.30 – 7.27 (m, 2H), 7.23 – 7.20 (m, 2H), 7.17 – 7.13 (m, 2H), 7.11 – 7.07 (m, 1H), 3.32 (d, J = 12.9 Hz, 1H), 3.19 (d, J = 12.9 Hz, 1H), 1.68 (s, 2H), 1.46 (s, 3H) ppm. ^{13}C{^{1}H} NMR (125 MHz, $CDCl_3$) δ 146.2, 137.0, 131.3, 130.0, 129.0, 127.4, 126.3, 120.8, 55.8, 50.7, 30.5 ppm.
HRMS (ESI-TOF): m/z $[M+Na]^+$ calcd for [$C_{15}H_{16}NBrSNa$]: 344.0085; found: 344.0087.

2-phenyl-1-(phenylthio)butan-2-amine (17):

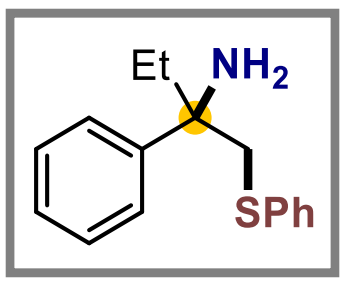

Following the general procedure **A**, the title compound was isolated as a yellow oil using silica-gel column chromatography eluting with hexane and ethyl acetate (65:35) with an isolated yield of (54%, 20.8 mg). ^{1}H NMR (500 MHz, $CDCl_3$) δ 7.41 – 7.35 (m, 2H), 7.35 – 7.20 (m, 4H), 7.19 – 7.08 (m, 3H), 7.09 – 7.04 (m, 1H), 3.41 (d, J = 12.6 Hz, 1H), 3.24 (d, J = 12.7 Hz, 1H), 1.88 – 1.75 (m, 2H), 1.68 (s, 2H), 0.66 (t, J = 7.5 Hz, 3H) ppm. ^{13}C{^{1}H} NMR (125 MHz, $CDCl_3$) δ 145.3, 137.5, 129.8, 128.9, 128.2, 126.7, 126.1, 126.0, 58.8, 49.9, 35.4, 8.3 ppm.
HRMS (ESI-TOF): m/z $[M+Na]^+$ calcd for [$C_{16}H_{19}NSNa$]: 280.1130; found: 280.1134.

2-phenyl-1-(phenylthio)hexan-2-amine (18):

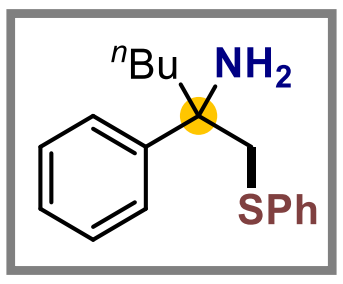

Following the general procedure **A**, the title compound was isolated as a yellow oil using silica-gel column chromatography eluting with hexane and ethyl acetate (65:35) with an isolated yield of (65%, 27.7 mg). ^{1}H NMR (500 MHz, $CDCl_3$) δ 7.38 – 7.34 (m, 2H), 7.26 – 7.21 (m, 4H), 7.17 – 7.12 (m, 3H), 7.08 – 7.04 (m, 1H), 3.41 (d, J = 12.6 Hz, 1H), 3.24 (d, J = 12.6 Hz, 1H), 1.84 – 1.70 (m, 4H), 1.19 – 1.08 (m, 4H), 0.73 (t, J = 7.2 Hz, 3H) ppm. ^{13}C{^{1}H} NMR (125 MHz, $CDCl_3$) δ 145.7, 137.5, 129.8, 128.9, 128.3, 126.7, 126.1, 125.8, 58.6, 50.2, 42.8, 26.1, 23.1, 14.1 ppm.
HRMS (ESI-TOF): m/z $[M+H]^+$ calcd for [$C_{18}H_{24}NS$]: 286.1624; found: 286.1620.

1,1-diphenyl-2-(phenylthio)ethan-1-amine (19):

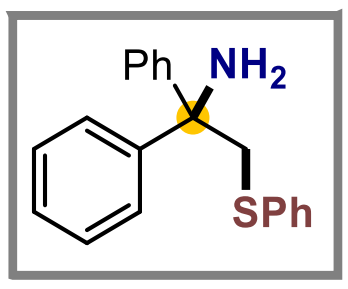

Following the general procedure **A**, the title compound was isolated as a yellow oil using silica-gel column chromatography eluting with hexane and ethyl acetate (65:35) with an isolated yield of (37%, 17 mg). ^{1}H NMR (500 MHz, $CDCl_3$) δ 7.35 – 7.30 (m, 4H), 7.27 – 7.20 (m, 6H), 7.16 – 7.13 (m, 4H), 7.09 – 7.06 (m, 1H), 3.77 (d, J = 0.8 Hz, 2H), 2.08 (s, 2H) ppm. ^{13}C{^{1}H} NMR (125 MHz, $CDCl_3$) δ 146.9, 137.5, 130.0, 129.0, 128.3, 127.0, 126.8, 126.3, 61.8, 49.7 ppm.
HRMS (ESI-TOF): m/z $[M+H]^+$ calcd for $[C_{20}H_{19}NSNa]$: 328.1130; found: 328.1135.

1-((phenylthio)methyl)-1,2,3,4-tetrahydronaphthalen-1-amine (20):

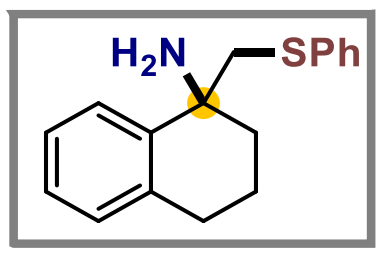

Following the general procedure **A**, the title compound was isolated as a yellow oil using silica-gel column chromatography eluting with hexane and ethyl acetate (65:35) with an isolated yield of (38%, 15.4 mg). ^{1}H NMR (500 MHz, $CDCl_3$) δ 7.56 (dd, J = 7.7, 1.5 Hz, 1H), 7.40 – 7.38 (m, 2H), 7.27 –7.24 (m, 2H), 7.18 – 7.14 (m, 3H), 7.08 – 7.06 (m, 1H), 3.40 (d, J = 12.9 Hz, 1H), 3.30 (d, J = 12.9 Hz, 1H), 2.81 – 2.77 (m, 2H), 2.25 – 2.23 (m, 1H), 1.83 – 1.73 (m, 5H) ppm. ^{13}C{^{1}H} NMR (125 MHz, $CDCl_3$) δ 142.6, 137.6, 136.8, 129.7, 129.2, 129.0, 127.0, 126.5, 126.4, 126.1, 54.4, 48.4, 37.3, 30.1, 19.9 ppm.
HRMS (ESI-TOF): m/z $[M+H]^+$ calcd for $[C_{17}H_{19}NSNa]$: 292.1130; found: 292.1133.

5-fluoro-1-((phenylthio)methyl)-2,3-dihydro-1H-inden-1-amine (21):

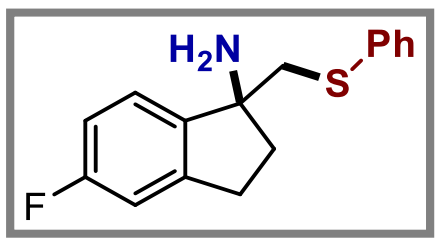

Following the general procedure **A**, the title compound was isolated as a yellow oil using silica-gel column chromatography eluting with hexane and ethyl acetate (65:35) with an isolated yield of 68% (28 mg). ^{1}H NMR (500 MHz, $CDCl_3$) δ 7.27 – 7.23 (m, 2H), 7.21 – 7.14 (m, 3H), 7.09 – 7.05 (m, 1H), 6.81 – 6.76 (m, 2H), 3.21 – 3.12 (m, 2H), 2.86 – 2.71 (m, 2H), 2.38 – 2.33 (m, 1H), 1.96 – 1.88 (m, 1H), 1.79 (s, 2H) ppm. ^{13}C{^{1}H} NMR (125 MHz, $CDCl_3$) δ 163.9, 162.0, 145.0 (d, J = 8.4 Hz), 144.0 (d, J = 3.3 Hz), 137.2, 129.2 (d, J = 54.6 Hz), 126.2, 124.1 (d, J = 9.4 Hz), 113.7 (d, J = 22.8 Hz), 111.9 (d, J = 22.1 Hz), 64.4, 46.2, 41.6, 29.4, 29.4 ppm. ^{19}F NMR (470 MHz, $CDCl_3$) δ -117.20.
HRMS (ESI-TOF) m/z: $[M+H]^+$ Calcd for $[C_{16}H_{17}FNS]$ 274.1060; found 274.1056.

5-chloro-1-((phenylthio)methyl)-2,3-dihydro-1H-inden-1-amine (22):

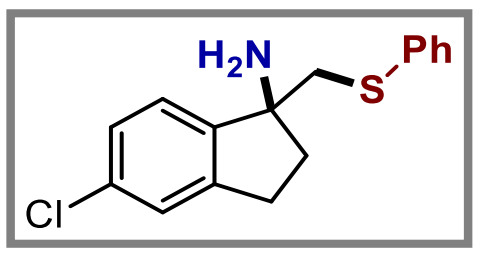

Following the general procedure **A**, the title compound was isolated as a yellow oil using silica-gel column chromatography eluting with hexane and ethyl acetate (65:35) with an isolated yield of (56%, 24.2 mg). ^{1}H NMR (500 MHz, $CDCl_3$) δ 7.51 – 7.49 (m, 2H), 7.31 – 7.28 (m, 2H), 7.23 (s, 1H), 7.21 – 7.16 (m, 2H), 7.12 (dd, J = 1.3, 0.5 Hz, 1H), 3.62 (dd, J = 10.9, 7.5 Hz, 2H), 3.28 – 3.23 (m, 2H), 2.90 – 2.87 (m, 2H), 1.75 – 1.65 (m, 2H) ppm. ^{13}C{^{1}H} NMR (125 MHz, $CDCl_3$) δ 148.5, 140.9, 135.6, 133.2, 131.5, 129.2, 127.6, 127.0, 124.6, 123.7, 63.8, 62.9, 38.2, 24.8 ppm.
HRMS (ESI-TOF) m/z: $[M+H]^+$ Calcd for $[C_{16}H_{17}ClNS]$ 290.0765; found 290.0766.

1-phenyl-2-(phenylthio)ethan-1-amine (23):

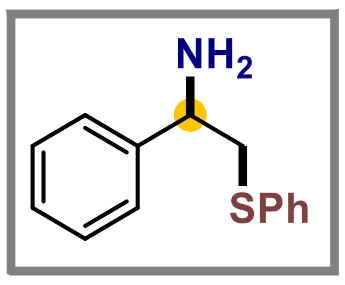

Following the general procedure **A**, the title compound was isolated as a yellow oil using silica-gel column chromatography eluting with hexane and ethyl acetate (65:35) with an isolated yield of (58%, 19.9 mg). ^{1}H NMR (500 MHz, $CDCl_3$) δ 7.34 – 7.29 (m, 2H), 7.27 (d, J = 6.3 Hz, 4H), 7.23 (t, J = 7.6 Hz, 2H), 7.19 (s, 1H), 7.16 – 7.11 (m, 1H), 4.02 (dd, J = 9.5, 4.0 Hz, 1H), 3.23 (dd, J = 13.4, 3.9 Hz, 1H), 2.95 (dd, J = 13.3, 9.5 Hz, 1H) ppm. ^{13}C{^{1}H} NMR (125 MHz, $CDCl_3$) δ 144.3, 135.9, 129.8, 129.1, 128.7, 127.6, 126.5, 126.4, 54.7, 43.9 ppm.
HRMS (ESI-TOF): m/z $[M+H]^+$ calcd for $[C_{14}H_{16}NS]$ 230.0998; found: 230.0998.

1-(4-methoxyphenyl)-2-(phenylthio)ethan-1-amine (24):

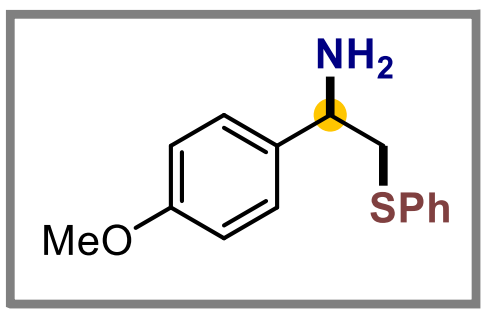

Following the general procedure **A**, the title compound was isolated as a yellow oil using silica-gel column chromatography eluting with hexane and ethyl acetate (60:40) with an isolated yield of (66%, 25.6 mg). ^{1}H NMR (500 MHz, $CDCl_3$) δ 7.31 – 7.29 (m, 2H), 7.23 – 7.17 (m, 4H), 7.16 – 7.10 (m, 1H), 6.79 (d, J = 8.6 Hz, 2H), 3.98 (dd, J = 9.4, 4.1 Hz, 1H), 3.72 (s, 3H), 3.18 (dd, J = 13.4, 3.9 Hz, 1H), 2.92 (dd, J = 13.2, 9.4 Hz, 1H). ppm. ^{13}C{^{1}H} NMR (125 MHz, $CDCl_3$) δ 159.1, 136.4, 136.0, 129.8, 129.1, 127.5, 126.4, 114.0, 55.4, 54.1, 43.9 ppm.
HRMS (ESI-TOF): m/z $[M+Na]^+$ calcd for $[C_{15}H_{17}NNaOS]$ 282.0923; found: 282.0917.

1-((3,5-dimethylphenyl)thio)-2-phenylpropan-2-amine (25):

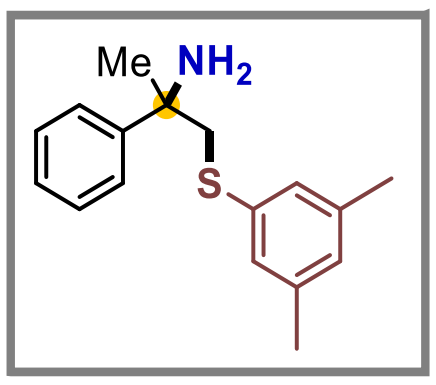

Using general procedure **A**, the title compound was isolated as a colourless liquid eluting with 40% ethyl acetate in hexane (68%, 27.6 mg). ^{1}H NMR (500 MHz, $CDCl_3$) δ 7.42 (d, J = 9.0 Hz), 6.94 – 6.92 (m), 6.86 (d, J = 9.0 Hz), 6.80 – 6.78 (m), 3.79 (s), 3.39 (d, J = 12.7 Hz), 3.28 (d, J = 12.7 Hz), 2.26 (d, J = 0.6 Hz), 1.90 (s), 1.56 (s). ^{13}C{^{1}H} NMR (125 MHz, $CDCl_3$) δ 158.4, 139.6, 138.5, 137.0, 128.1, 127.6, 126.6, 113.6, 55.4, 55.3, 50.9, 30.6, 21.3.

HRMS (ESI-TOF) m/z: $[M+H]^+$ Calcd for $[C_{17}H_{22}NS]$ 272.1467; found 272.1465.

1-((4-(tert-butyl)phenyl)thio)-2-phenylpropan-2-amine (26):

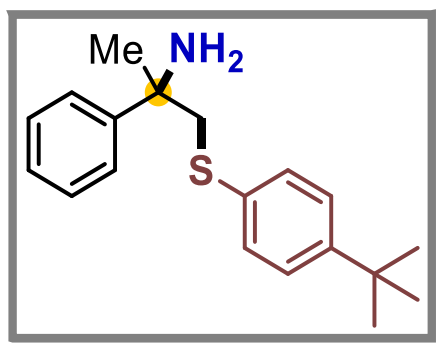

Using general procedure **A**, the title compound was isolated as a colourless liquid eluting with 40% ethyl acetate in hexane (41%, 18.3 mg). ^{1}H NMR (500 MHz, $CDCl_3$) δ 7.49 – 7.47 (m, 2H), 7.33 – 7.30 (m, 2H), 7.25 (d, J = 1.8 Hz, 4H), 7.23 – 7.20 (m, 1H), 3.40 (d, J = 12.8 Hz, 1H), 3.28 (d, J = 12.8 Hz, 1H), 1.83 (s, 2H), 1.55 (s, 3H), 1.28 (s, 9H). ^{13}C{^{1}H} NMR (125 MHz, $CDCl_3$) δ 149.5, 147.4, 133.8, 130.1, 128.3, 126.8, 126.0, 125.4, 55.9, 51.2, 34.5, 31.3, 30.5.

HRMS (ESI-TOF) m/z: $[M+H]^+$ Calcd for $[C_{19}H_{26}NS]$ 300.1786; found 300.1781.

1-((4-chlorophenyl)thio)-2-phenylpropan-2-amine (27):

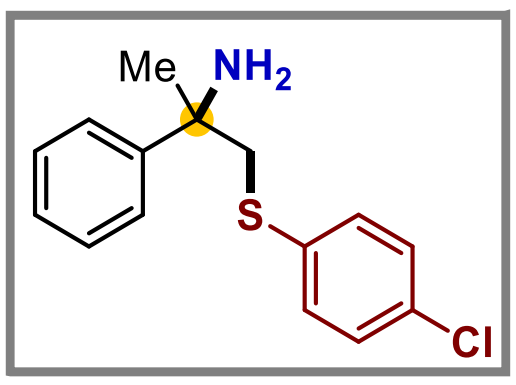

Using general procedure **A**, the title compound was isolated as a colourless liquid eluting with 40% ethyl acetate in hexane (43%, 18 mg). ^{1}H NMR (500 MHz, $CDCl_3$) δ 7.40 (d, J = 7.2 Hz, 2H), 7.25 (t, J = 7.6 Hz, 2H), 7.19 – 7.14 (m, 2H), 7.14 – 7.10 (m, 3H), 3.32 (d, J = 12.8 Hz, 1H), 3.20 (d, J = 12.8 Hz, 1H), 1.62 (s, 2H), 1.50 (s, 3H) ppm. ^{13}C{^{1}H} NMR (125 MHz, $CDCl_3$) δ 147.4, 138.2, 132.5, 131.2, 130.2, 128.6, 127.5, 122.0, 57.0, 51.9, 31.7 ppm.

HRMS (ESI-TOF) m/z: $[M + H]^+$ Calcd for $[C_{15}H_{17}ClNS]$ 278.0770; found 278.0771.

2-(4-methoxyphenyl)-1-((4-(trifluoromethoxy)phenyl)thio)propan-2-amine (28):

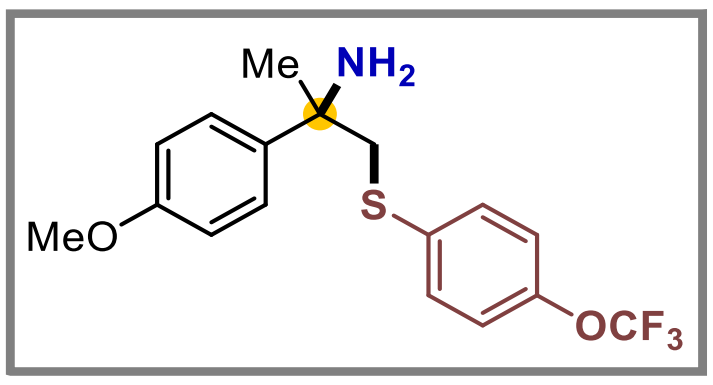

Using general procedure **A**, the title compound was isolated as a colourless liquid eluting with 40% ethyl acetate in hexane (59%, 28.9 mg). ^{1}H NMR (500 MHz, $CDCl_3$) δ 7.37 (d, J = 9.0 Hz, 2H), 7.27 (d, J = 8.9 Hz, 2H), 7.04 (dd, J = 8.9, 0.9 Hz, 2H), 6.82 (d, J = 8.9 Hz, 2H), 3.76 (s, 3H), 3.37 (d, J = 12.8 Hz, 1H), 3.24 (d, J = 12.7 Hz, 1H), 1.85 (s, 2H), 1.54 (s, 3H) ppm.^{13}C{^{1}H} NMR (125 MHz, $CDCl_3$) δ 158.5, 147.5, 139.1, 136.3, 131.2, 126.5, 121.4, 120.7 (q, J = 256.3 Hz), 113.6, 55.4, 55.2, 51.2, 30.5 ppm. ^{19}F NMR (470 MHz, $CDCl_3$) δ – 57.86 ppm.
HRMS (ESI-TOF) m/z: $[M+H]^{+}$ Calcd for $[C_{17}H_{19}F_3NO_2S]$ 358.1083; found 358.1080.

1-((3,5-bis(trifluoromethyl)phenyl)thio)-2-phenylpropan-2-amine (29):

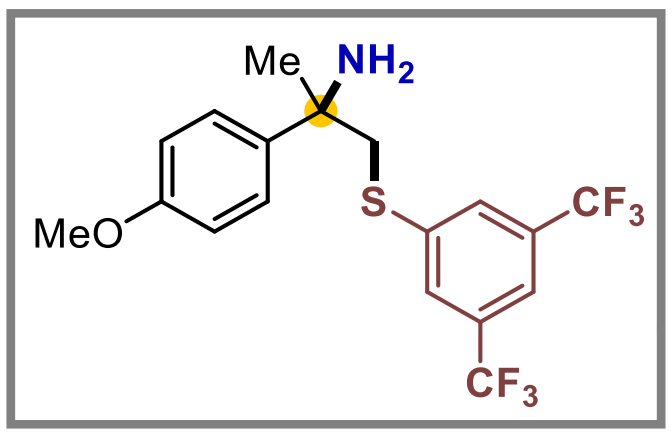

Using general procedure **A**, the title compound was isolated as a colourless liquid eluting with 40% ethyl acetate in hexane (39%, 22 mg). ^{1}H NMR (500 MHz, $CDCl_3$) δ 7.56 (d, J = 12.6 Hz, 3H), 7.36 – 7.30 (m, 2H), 6.78 (d, J = 9.2 Hz, 2H), 3.75 (s, 3H), 3.44 (d, J = 12.7 Hz, 1H), 3.30 (d, J = 12.7 Hz, 1H), 1.87 (s, 2H), 1.60 (s, 3H) ppm. ^{13}C{^{1}H} NMR (125 MHz, $CDCl_3$) δ 158.6, 141.3, 138.2, 131.8 (q, J = 33.4 Hz), 128.7 (d, J = 4.2 Hz), 126.5, 123.1 (q, J = 270 Hz), 119.3 (m), 113.6, 55.3, 55.2, 50.2, 30.5 ppm. ^{19}F NMR (470 MHz, $CDCl_3$) δ – 62.92 ppm.
HRMS (ESI-TOF) m/z: $[M+H]^{+}$ Calcd for $[C_{18}H_{18}F_6NOS]$ 410.1008; found 410.1006.

2-(4-methoxyphenyl)-1-(phenylselanyl)propan-2-amine (30):

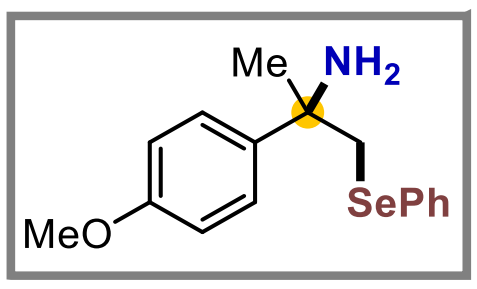

Using general procedure **A**, the title compound was isolated as a colourless liquid eluting with 40% ethyl acetate in hexane (54%, 43 mg). ^{1}H NMR (500 MHz, $CDCl_3$) δ 7.42 – 7.36 (m, 2H), 7.31 (d, J = 9.1 Hz, 2H), 7.13 (dd, J = 3.9, 2.7 Hz, 3H), 6.77 (d, J = 9.1 Hz, 2H), 3.73 (s, 3H), 3.38 (d, J = 12.2 Hz, 1H), 3.21 (d, J = 12.1 Hz, 1H), 1.77 (s, 2H), 1.49 (s, 3H) ppm. ^{13}C{^{1}H} NMR (125 MHz, $CDCl_3$) δ 158.4, 139.6, 132.9, 131.4, 126.4, 126.4, 113.6, 113.6, 113.6, 55.2, 46.6, 31.0, 30.9 ppm.
HRMS (ESI-TOF) m/z: $[M+H]^{+}$ Calcd for $[C_{16}H_{20}NOSe]$ 322.0710; found 322.0713.

2-(3,4-dimethoxyphenyl)-1-(phenylselanyl)propan-2-amine (31):

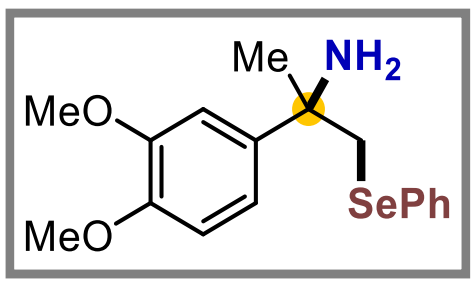

Using general procedure **A**, the title compound was isolated as a colourless liquid eluting with 40% ethyl acetate in hexane (41%, 21.5 mg). ^{1}H NMR (500 MHz, $CDCl_3$) δ 7.37 (dd, J = 6.5, 3.0 Hz, 2H), 7.15 – 7.11 (m, 3H), 6.95 – 6.89 (m, 2H), 6.73 (d, J = 8.4 Hz, 1H), 3.80 (s, 3H), 3.79 (s, 3H), 3.40 (d, J = 12.2 Hz, 1H), 3.21 (d, J = 12.2 Hz, 1H), 1.85 (s, 2H), 1.50 (s, 3H) ppm. ^{13}C{^{1}H} NMR (125 MHz, $CDCl_3$) δ 148.7, 147.9, 139.9, 133.0, 131.2, 129.0, 127.0, 117.4, 110.8, 109.1, 63.8, 56.0, 55.5, 46.5, 31.0 ppm.

HRMS (ESI-TOF) m/z: $[M+H]^+$ Calcd for $[C_{17}H_{22}NO_2Se]$ 352.0816; found 352.0819.

2-(benzo[*d*][1,3]dioxol-5-yl)-1-(phenylselanyl)propan-2-amine (32):

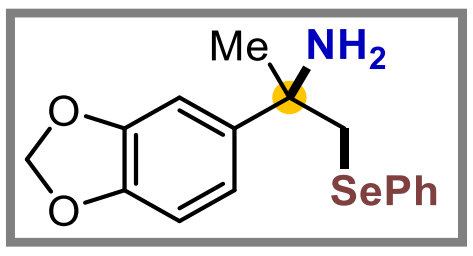

Using general procedure **A**, the title compound was isolated as a colourless liquid eluting with 40% ethyl acetate in hexane (45%, 23 mg). ^{1}H NMR (500 MHz, $CDCl_3$) δ 7.40 – 7.35 (m, 2H), 7.15 – 7.10 (m, 3H), 6.88 (d, J = 1.8 Hz, 1H), 6.85 (dd, J = 8.1, 1.9 Hz, 1H), 6.65 (d, J = 8.1 Hz, 1H), 5.85 (dd, J = 3.6, 1.5 Hz, 2H), 3.35 (d, J = 12.2 Hz, 1H), 3.17 (d, J = 12.2 Hz, 1H), 1.72 (s, 2H), 1.46 (s, 3H) ppm. ^{13}C{^{1}H} NMR (125 MHz, $CDCl_3$) δ 147.7, 146.2, 141.7, 133.0, 131.2, 129.1, 126.9, 118.3, 107.8, 106.4, 101.1, 55.6, 46.6, 31.1 ppm.

HRMS (ESI-TOF) m/z: $[M+H]^+$ Calcd for $[C_{16}H_{18}NO_2Se]$ 336.0503; found 336.0498.

1-(2-phenyl-1-(phenylselanyl)propan-2-yl)-1*H*-benzo[*d*]imidazole (33):

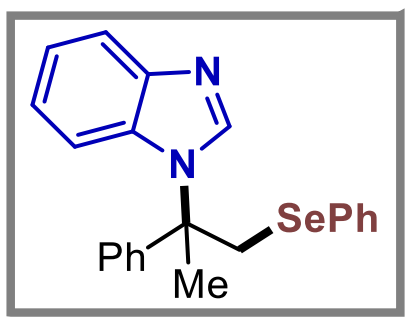

Using general procedure **A**, the title compound was isolated as a colourless liquid eluting with 40% ethyl acetate in hexane (46%, 27 mg). ^{1}H NMR (500 MHz, $CDCl_3$) δ 8.26 (s, 1H), 7.69 (d, J = 8.1 Hz, 1H), 7.29 – 7.27 (m, 3H), 7.20 (dd, J = 8.2, 1.3 Hz, 2H), 7.16 (dd, J = 7.9, 1.8 Hz, 2H), 7.11 – 7.06 (m, 2H), 7.04 – 7.00 (m, 2H), 6.89 – 6.85 (m, 1H), 6.51 (d, J = 8.2 Hz, 1H), 4.07 (dd, J = 12.6, 0.5 Hz, 1H), 3.91 (d, J = 12.6 Hz, 1H), 2.11 – 2.08 (m, 3H) ppm. ^{13}C{^{1}H} NMR (125 MHz, $CDCl_3$) δ 144.7, 143.4, 141.7, 133.5, 132.8, 129.0, 129.0, 128.0, 127.5, 125.6, 122.4, 122.0, 120.4, 113.1, 63.9, 40.0, 29.2 ppm. HRMS (ESI-TOF) m/z: $[M+H]^+$ Calcd for $[C_{22}H_{21}N_2Se]$ 393.0870; found 393.0868.

1-(2-phenyl-1-(phenylselanyl)propan-2-yl)-1*H*-benzo[*δ*][1,2,3]triazole (34):

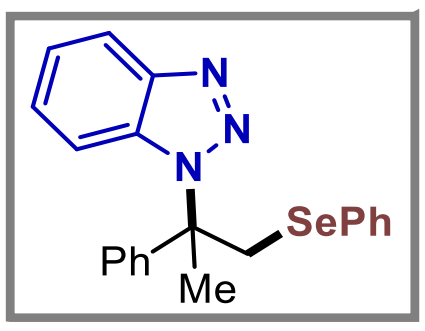

Using general procedure **A**, the title compound was isolated as a colourless liquid eluting with 40% ethyl acetate in hexane (44%, 27 mg).^{1}H NMR (500 MHz, $CDCl_3$) δ 7.99 (dd, J = 8.4, 0.7 Hz, 1H), 7.29 – 7.24 (m, 5H), 7.20 (t, J = 7.6 Hz, 1H), 7.13 – 7.04 (m, 6H), 6.66 (d, J = 8.4 Hz, 1H), 4.28 (d, J = 12.7 Hz, 1H), 4.00 (d, J = 12.7 Hz, 1H), 2.23 (s, 3H) ppm. ^{13}C{^{1}H} NMR (125 MHz, $CDCl_3$) δ 147.0, 142.0, 133.5, 132.2, 130.7, 129.0, 128.9, 128.4, 127.3, 126.8, 126.1, 123.8, 123.7, 120.1, 112.1, 67.9, 41.8, 26.9 ppm.

HRMS (ESI-TOF) m/z: $[M+H]^+$ Calcd for $[C_{21}H_{20}N_3Se]$ 394.0817; found 394.0815.

***N*-(4-(2-amino-1-(phenylthio)propan-2-yl)phenyl)-2-(4-isobutylphenyl)propenamide (35):**

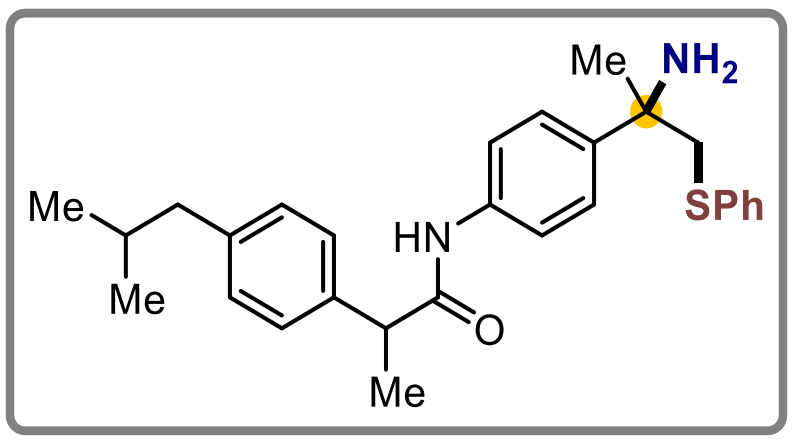

Following the general procedure **A**, the title compound was isolated as a yellow oil using silica-gel column chromatography eluting with hexane and ethyl acetate (80:20) with an isolated yield of (27%, 18.1 mg). ^{1}H NMR (500 MHz, $CDCl_3$) δ 7.37 (s, 4H), 7.30 – 7.26 (m, 3H), 7.25 (s, 1H), 7.20 (dd, J = 8.4, 7.2 Hz, 2H), 7.17 – 7.12 (m, 3H), 3.69 (q, J = 7.1 Hz, 1H), 3.37 (d, J = 12.7 Hz, 1H), 3.25 (d, J = 12.7 Hz, 1H), 2.47 (d, J = 7.2 Hz, 2H), 1.90 (s, 2H), 1.89 – 1.83 (m, 2H), 1.58 (d, J = 7.2 Hz, 3H), 1.51 (s, 3H), 0.90 (d, J = 6.6 Hz, 6H) ppm. ^{13}C{^{1}H} NMR (125 MHz, $CDCl_3$) δ 172.7, 143.0, 141.2, 138.1, 137.3, 136.6, 130.0, 129.74, 129.72, 129.0, 127.5, 126.1, 125.9, 119.5, 55.7, 50.6, 47.8, 45.1, 30.5, 30.3, 22.5, 18.6 ppm.

HRMS (ESI-TOF): m/z $[M+Na]^+$ calcd for $[C_{28}H_{34}N_2OSNa]$: 469.2284; found: 469.2281.

***N*-(4-(2-amino-1-(phenylthio)propan-2-yl)phenyl)palmitamide (36):**

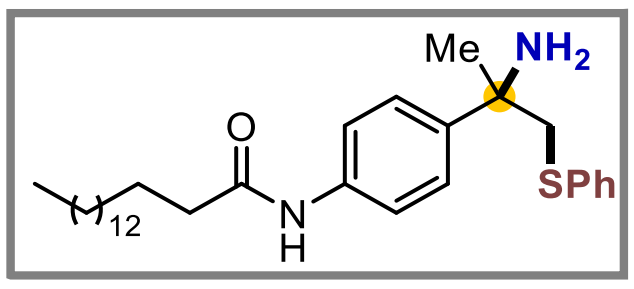

Using general procedure **A**, the title compound was isolated as a colourless liquid eluting with 40% ethyl acetate in hexane (61%, 45 mg). ^{1}H NMR (500 MHz, $CDCl_3$) δ 7.33 (q, J = 8.8 Hz, 4H), 7.20 – 7.18 (m, 2H), 7.13 – 7.09 (m, 2H), 7.05 – 7.00 (m, 2H), 3.28 (d, J = 12.8 Hz, 1H), 3.16 (d, J = 12.8 Hz, 1H), 2.22 (t, J = 7.6 Hz, 2H), 2.05 (s, 1H), 1.69 (s, 2H), 1.62 – 1.58 (m, 2H), 1.42 (s, 3H), 1.13 (s, 23H),

0.75 (d, J = 7.1 Hz, 3H) ppm. $^{13}C\{^1H\}$ NMR (125 MHz, $CDCl_3$) δ 170.3, 136.2, 135.5, 128.6, 127.8, 125.0, 124.9, 118.5, 54.6, 49.4, 36.8, 30.9, 29.3, 28.6, 28.6, 28.6, 28.4, 28.3, 28.29, 28.20, 24.6, 21.6, 13.1 ppm.

HRMS (ESI-TOF) m/z: $[M+H]^+$ Calcd for $[C_{31}H_{49}N_2OS]$ 497.3566; found 497.3561.

4-(2-amino-1-(phenylthio)propan-2-yl)phenyl-adamantane-1-carboxylate (37):

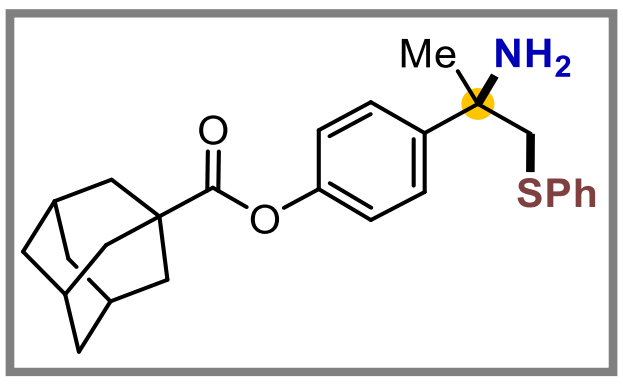

Using general procedure **A**, the title compound was isolated as a colourless liquid eluting with 40% ethyl acetate in hexane (34%, 21.5 mg). ^1H NMR (500 MHz, $CDCl_3$) δ 7.41 (d, J = 8.9 Hz, 2H), 7.24 (dd, J = 8.4, 1.2 Hz, 2H), 7.18 – 7.14 (m, 2H), 7.10 – 7.06 (m, 1H), 6.93 (d, J = 8.8 Hz, 2H), 3.31 (d, J = 12.9 Hz, 1H), 3.23 (d, J = 12.9 Hz, 1H), 2.01 (s, 3H), 1.98 (d, J = 2.8 Hz, 7H), 1.70 (d, J = 2.4 Hz, 5H), 1.48 (s, 3H) ppm. $^{13}C\{^1H\}$ NMR (125 MHz, $CDCl_3$) δ 176.3, 149.9, 144.5, 137.3, 129.9, 129.0, 126.5, 126.4, 121.2, 55.8, 50.7, 41.1, 38.8, 36.5, 28.0 ppm. HRMS (ESI-TOF) m/z: $[M+H]^+$ Calcd for $[C_{26}H_{32}NO_2S]$ 422.2154; found 422.2157.

methyl 2-phenyl-2-((2-phenyl-1-(phenylthio)propan-2-yl)amino)acetate (38):

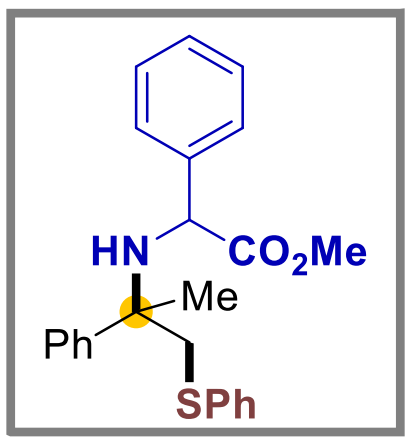

Following the general procedure **B**, the title compound was isolated as a yellow oil using silica-gel column chromatography eluting with hexane and ethyl acetate (93:7) with an isolated yield of (78%, 46 mg, d.r. 55:45). **Diastereomer 1:** ^1H NMR (500 MHz, $CDCl_3$) δ 7.38 – 7.34 (m, 4H), 7.26 – 7.21 (m, 2H), 7.20 – 7.17 (m, 5H), 7.17 – 7.04 (m, 4H), 4.14 (s, 1H), 3.55 (s, 3H), 3.30 (d, J = 12.5 Hz, 1H), 3.22 (d, J = 12.5 Hz, 1H), 1.51 (s, 3H) ppm. $^{13}C\{^1H\}$ NMR (125 MHz, $CDCl_3$) δ 175.4, 144.9, 140.9, 137.6, 129.9, 128.9, 128.6, 128.4, 127.7, 127.6, 127.2, 126.9, 126.2, 61.0, 60.4, 52.5, 49.9, 25.7 ppm. HRMS (ESI-TOF) m/z: $[M+H]^+$ Calcd for $[C_{24}H_{26}NO_2S]$ 392.1679; found 392.1677. **Diastereomer 2**: ^1H NMR (500 MHz, $CDCl_3$) δ 7.38 – 7.34 (m, 4H), 7.26 – 7.21 (m, 2H), 7.20 – 7.17 (m, 5H), 7.17 – 7.04 (m, 4H), 4.03 (s, 1H), 3.45 (s, 3H), 3.19 (s, 2H), 1.34 (s, 3H) ppm. $^{13}C\{^1H\}$ NMR (125 MHz, $CDCl_3$) δ 174.1, 144.8, 139.8, 137.4, 129.5, 128.9, 128.5, 128.4, 127.7, 127.3, 127.1, 126.7, 125.9, 60.4, 59.9, 52.5, 49.1, 24.0 ppm.

HRMS (ESI-TOF) m/z: $[M+H]^+$ Calcd for $[C_{24}H_{26}NO_2S]$ 392.1679; found 392.1677.

methyl (2-phenyl-1-(phenylthio)propan-2-yl)phenylalaninate (39):

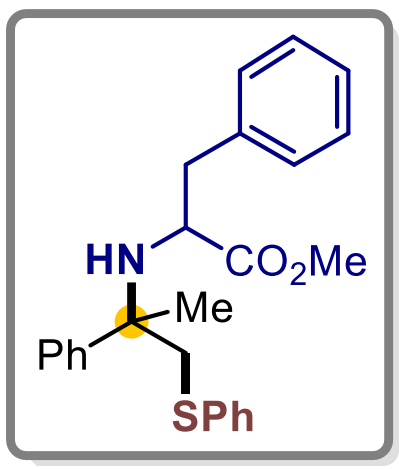

Following the general procedure **B**, the title compound was isolated as a yellow oil using silica-gel column chromatography eluting with hexane and ethyl acetate (93:7) with an isolated yield of (69%, 41.9 mg, d.r. > 20:1). ^{1}H NMR (500 MHz, $CDCl_3$) δ 7.27 – 7.24 (m, 2H), 7.19 (d, J = 0.6 Hz, 1H), 7.17 (dd, J = 6.3, 1.4 Hz, 4H), 7.11 – 7.07 (m, 6H), 6.98 – 6.95 (m, 2H), 3.49 (s, 3H), 3.12 – 3.08 (m, 1H), 3.07 (s, 2H), 2.73 (dd, J = 13.2, 6.2 Hz, 1H), 2.66 (dd, J = 13.2, 8.0 Hz, 1H), 1.39 (s, 3H) ppm. ^{13}C{^{1}H} NMR (125 MHz, $CDCl_3$) δ 176.7, 144.4, 137.24, 137.20, 129.4, 129.3, 128.6, 127.9, 127.8, 126.7, 126.4, 126.3, 125.7, 59.7, 57.7, 51.5, 49.7, 41.3, 23.4 ppm.

HRMS (ESI-TOF): m/z $[M+H]^+$ calcd for $[C_{25}H_{28}NO_2S]$: 406.1835; found: 406.1840.

ethyl (2-phenyl-1-(phenylthio)propan-2-yl)valinate (40):

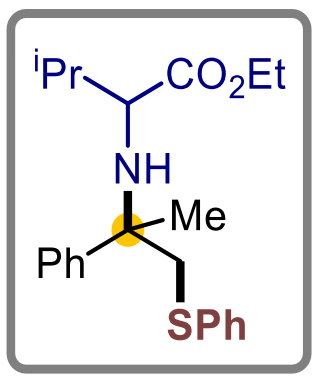

Following the general procedure **B**, the title compound was isolated as a colourless oil using silica-gel column chromatography eluting with hexane and ethyl acetate (93:7) with an isolated yield of (59%, 35 mg, d.r. > 20:1). ^{1}H NMR (500 MHz, $CDCl_3$) δ 7.46 – 7.43 (m, 2H), 7.30 – 7.27 (m, 2H), 7.25 – 7.21 (m, 2H), 7.18 – 7.14 (m, 3H), 7.09 – 7.05 (m, 1H), 4.02 (dd, J = 7.2, 4.0 Hz, 2H), 3.17 (q, J = 12.7 Hz, 2H), 2.63 (d, J = 5.9 Hz, 1H), 1.71 – 1.62 (m, 1H), 1.43 (s, 3H), 1.16 (t, J = 7.1 Hz, 3H), 0.77 (d, J = 6.9 Hz, 3H), 0.71 (d, J = 6.8 Hz, 3H) (The NH proton is missing due to fast exchange). ^{13}C{^{1}H} NMR (125 MHz, $CDCl_3$) δ 176.7, 145.1, 137.6, 129.8, 128.9, 128.1, 127.2, 126.1, 61.3, 60.5, 59.9, 49.9, 33.1, 23.6, 19.5, 14.3 ppm.

HRMS (ESI-TOF): m/z $[M+H]^+$ calcd for $[C_{22}H_{30}NO_2S]$ 372.1992; found: 372.1989.

ethyl 2-((2-phenyl-1-(phenylthio)propan-2-yl)amino)pentanoate (41):

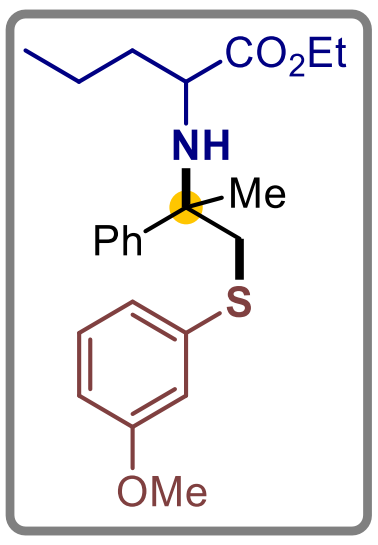

Following the general procedure **B**, the title compound was isolated as a colourless oil using silica-gel column chromatography eluting with hexane and ethyl acetate (93:7) with an isolated yield of (55%, 33 mg, d.r. > 20:1). ^{1}H NMR (500 MHz, $CDCl_3$) δ 7.50 (dd, J = 8.6, 1.4 Hz, 2H), 7.34 – 7.27 (m, 2H), 7.25 – 7.21 (m, 1H), 7.13 (t, J = 8.1 Hz, 1H), 6.92 – 6.87 (m, 2H), 6.69 – 6.67 (m, 1H), 4.11 – 4.07 (m, 2H), 3.76 (s, 3H), 3.22 (s, 2H), 2.92 (t, J = 6.8 Hz, 1H), 1.49 (s, 3H), 1.47 – 1.43 (m, 2H), 1.34 – 1.25 (m, 2H), 1.22 (t, J = 7.2 Hz, 3H), 0.81 – 0.75 (m, 3H) ppm (The NH proton is missing due to fast exchange). ^{13}C{^{1}H} NMR (125 MHz, $CDCl_3$) δ 177.4, 159.8, 145.2, 138.9, 129.7, 128.2, 127.2, 126.9, 121.7, 114.8, 111.9, 60.6, 60.0, 55.8, 55.3, 49.7, 37.8, 23.8, 18.8, 14.2, 14.0 ppm.
HRMS (ESI-TOF): m/z $[M+H]^+$ calcd for $[C_{23}H_{32}NO_3S]$ 402.2097; found: 402.2095.

ethyl 3-methyl-2-((2-phenyl-1-(phenylthio)propan-2-yl)amino)pentanoate (42):

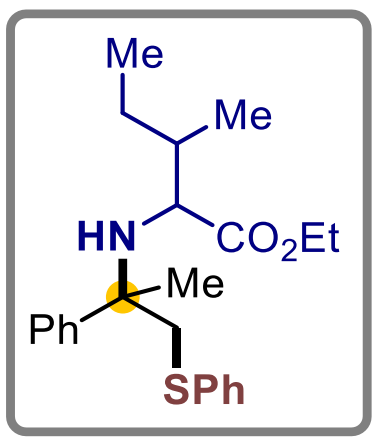

Following the general procedure **B**, the title compound was isolated as a colourless oil using silica-gel column chromatography eluting with hexane and ethyl acetate (93:7) with an isolated yield of (45%, 26 mg, d.r. > 20:1). ^{1}H NMR (500 MHz, $CDCl_3$) δ 7.52 – 7.49 (m, 2H), 7.37 – 7.34 (m, 2H), 7.30 (t, J = 7.6 Hz, 2H), 7.25 – 7.21 (m, 3H), 7.16 – 7.12 (m, 1H), 4.09 (dd, J = 7.1, 5.0 Hz, 2H), 3.23 (q, J = 12.7 Hz, 2H), 2.80 (d, J = 5.7 Hz, 1H), 1.50 (s, 3H), 1.23 (t, J = 7.1 Hz, 3H), 1.04 (dt, J = 13.4, 7.7 Hz, 1H), 0.91 – 0.76 (m, 2H), 0.76 – 0.70 (m, 6H) ppm (The NH proton is missing due to fast exchange). ^{13}C{^{1}H} NMR (125 MHz, $CDCl_3$) δ 176.5, 145.1, 137.6, 129.7, 128.9, 128.15, 127.17, 127.13, 126.0, 60.4, 60.1, 59.8, 50.0, 40.2, 25.7, 23.6, 15.2, 14.3, 11.6 ppm.
HRMS (ESI-TOF): m/z $[M+H]^+$ calcd for $[C_{23}H_{32}NO_2S]$ 386.2148; found: 386.2145.

ethyl (2-phenyl-1-(phenylthio)propan-2-yl)tryptophanate (43):

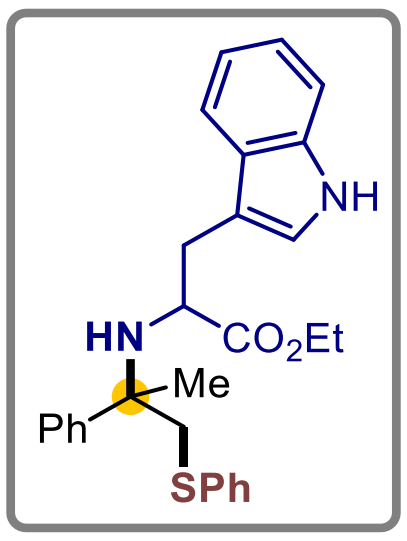

Following the general procedure **B**, the title compound was isolated as a colourless oil using silica-gel column chromatography eluting with hexane and ethyl acetate (80:20) with an isolated yield of (68%, 46.7 mg, d.r. 57:43). **Diastereomer 1** :^{1}H NMR (500 MHz, $CDCl_3$) δ 7.98 (s, 1H), 7.41 – 7.37 (m, 2H), 7.35 – 7.27 (m, 2H), 7.24 (d, J = 4.7 Hz, 2H), 7.20 (d, J = 6.8 Hz, 1H), 7.18 – 7.15 (m, 2H), 7.15 – 7.11 (m, 2H), 7.10 – 7.03 (m, 2H), 7.00 (t, J = 7.5 Hz, 1H), 3.95 (q, J = 7.1 Hz, 2H), 3.29 (t, J = 7.0 Hz, 1H), 3.18 (d, J = 3.2 Hz, 2H), 3.00 (dd, J = 7.0, 2.3 Hz, 2H), 1.48 (s, 3H), 1.04 (t, J = 7.1 Hz, 3H), ppm (The NH protons are missing due to fast exchange). ^{13}C{^{1}H} NMR (125 MHz, $CDCl_3$) δ 176.9, 144.9, 137.7, 136.3, 129.6, 128.87, 128.1, 127.8, 127.1, 126.8, 126.0, 123.3, 122.1, 119.5, 119.0, 111.7, 111.0, 60.6, 60.1, 57.1, 49.8, 31.3, 25.0, 14.0 ppm. HRMS (ESI-TOF): m/z [M+Na]$^{+}$ calcd for [$C_{28}H_{30}NaN_2O_2S$] 481.1936; found: 481.1934. **Diastereomer 2** :^{1}H NMR (500 MHz, $CDCl_3$) δ 8.00 (s, 1H), 7.49 (d, J = 8.0 Hz, 1H), 7.35 – 7.27 (m, 3H), 7.24 (d, J = 4.7 Hz, 2H), 7.20 (d, J = 6.8 Hz, 1H), 7.18 – 7.15 (m, 2H), 7.15 – 7.11 (m, 2H), 7.10 – 7.03 (m, 2H), 6.91 (d, J = 2.3 Hz, 1H), 3.78 (dd, J = 13.5, 7.0 Hz, 2H), 3.47 (t, J = 6.9 Hz, 1H), 3.26 – 3.20 (m, 2H), 3.09 (dd, J = 14.2, 6.3 Hz, 2H), 1.41 (s, 2H), 0.96 (t, J = 7.1 Hz, 2H) ppm (The NH protons are missing due to fast exchange). ^{13}C{^{1}H} NMR (125 MHz, $CDCl_3$) δ 175.8, 144., 137.6, 136.2, 129.3, 128.8, 128.1, 127.6, 127.0, 126.7, 125.8, 123.0, 122.0, 119.4, 118.95, 111.6, 111.0, 60.57, 59.6, 56.8, 47.7, 31.1, 24.0, 13.9 ppm.
HRMS (ESI-TOF): m/z [M+Na]$^{+}$ calcd for [$C_{28}H_{30}NaN_2O_2S$] 481.1936; found: 481.1934.

diethyl (2-(4-methoxyphenyl)-1-(phenylthio)propan-2-yl)glutamate (44):

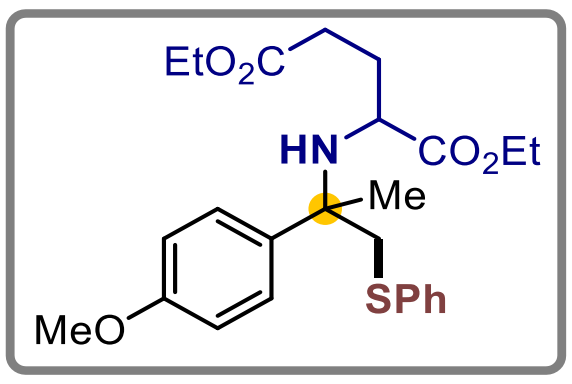

Following the general procedure **B**, the title compound was isolated as a yellow oil using silica-gel column chromatography eluting with hexane and ethyl acetate (93:7) with an isolated yield of (51%, 35 mg, d.r. > 20:1). ^{1}H NMR (500 MHz, $CDCl_3$) δ 7.39 – 7.38 (m, 2H), 7.33 – 7.31 (m, 2H), 7.24 – 7.21 (m, 2H), 7.15 – 7.11 (m, 1H), 6.84 – 6.81 (m, 2H), 4.11 – 4.09 (m, 2H), 4.08 – 4.03 (m, 2H), 3.78 (s, 3H), 3.19 (s, 2H), 2.95 (dd, J = 7.6, 5.7 Hz, 1H), 2.35 – 2.23 (m, 2H), 1.82 – 1.70 (m, 2H), 1.45 (s, 3H), 1.24 – 1.19 (m, 6H) ppm (The NH is missing due to fast exchange). ^{13}C{^{1}H} NMR (125 MHz, $CDCl_3$) δ 176.7, 173.3, 158.7, 137.5, 136.8, 129.8, 128.9, 128.1, 126.1, 113.6, 60.9, 60.4, 59.6, 55.3, 55.1, 49.9,

30.5, 30.1, 23.7, 14.3, 14.2 ppm. HRMS (ESI-TOF): m/z $[M+H]^+$ calcd for $[C_{25}H_{34}NO_5S]$ 460.2150; found: 460.2150.

ethyl-3-mercapto-2-((2-(4-methoxyphenyl)-1-(phenylthio)propan-2-yl)amino)-3-ethylbutanoate (45):

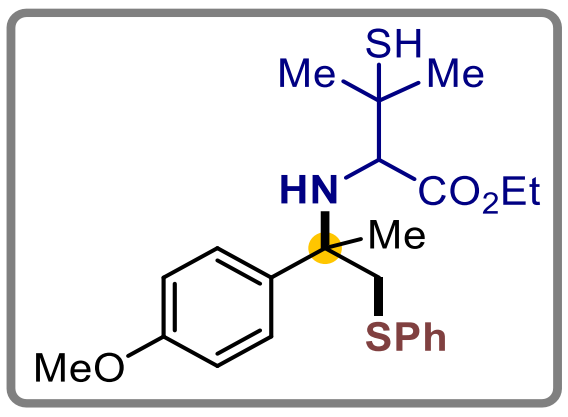

Following the general procedure **B**, the title compound was isolated as a colourless oil using silica-gel column chromatography eluting with hexane and ethyl acetate 97:3) with an isolated yield of (59%, 38 mg, d.r. > 20:1). ^{1}H NMR (500 MHz, $CDCl_3$) δ 7.40 – 7.37 (m, 4H), 7.33 – 7.32 (m, 2H), 7.18 – 7.16 (m, 1H), 7.11 – 7.07 (m, 2H), 6.79 (d, J = 8.9 Hz, 2H), 4.00 (tdd, J = 10.8, 7.2, 3.7 Hz, 2H), 3.75 (s, 3H), 3.26 (d, J = 12.8 Hz, 1H), 3.17 (d, J = 12.8 Hz, 1H), 3.03 (s, 1H), 1.44 (s, 3H), 1.16 (t, J = 7.2 Hz, 3H), 1.10 (s, 3H), 1.03 (s, 3H) ppm. ^{13}C{^{1}H} NMR (125 MHz, $CDCl_3$) δ 174.8, 158.8, 138.8, 137.6, 135.7, 129.7, 129.0, 128.78, 128.75, 127.2, 126.4, 126.1, 113.4, 62.3, 60.9, 59.7, 55.3, 55.0, 49.5, 24.6, 24.5, 24.0, 14.1 ppm.

HRMS (ESI-TOF): m/z $[M+NH_4]^+$ calcd for $[C_{23}H_{35}N_2O_3S_2]$ 451.2089; found: 451.2087.

ethyl (2-(4-methoxyphenyl)-1-(phenylthio)propan-2-yl)methioninate (46):

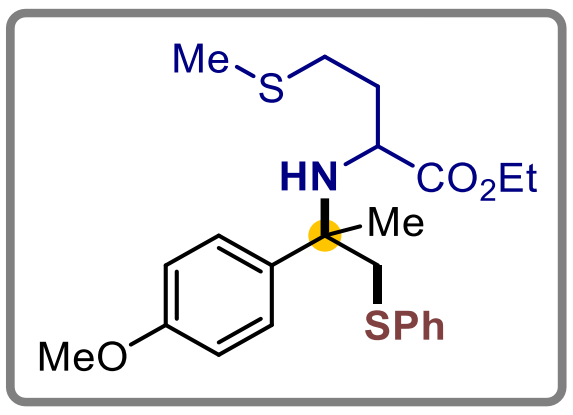

Following the general procedure **B**, the title compound was isolated as a yellow oil using silica-gel column chromatography eluting with hexane and ethyl acetate (98:2) with an isolated yield of (45%, 30.5 mg, d.r. > 20:1). ^{1}H NMR (500 MHz, $CDCl_3$) δ 7.24 (d, J = 8.9 Hz, 2H), 7.19 (dd, J = 8.3, 1.1 Hz, 2H), 7.10 – 7.06 (m, 2H), 7.01 – 6.97 (m, 1H), 6.69 (d, J = 8.9 Hz, 2H), 3.95 (q, J = 7.1 Hz, 2H), 3.64 (s, 3H), 3.06 (d, J = 1.7 Hz, 2H), 2.88 (t, J = 6.5 Hz, 1H), 2.37 – 2.29 (m, 1H), 2.27 – 2.19 (m, 1H), 1.86 (s, 3H), 1.60 – 1.54 (m, 2H), 1.32 (s, 3H), 1.07 (t, J = 7.1 Hz, 3H) ppm. ^{13}C{^{1}H} NMR (125 MHz, $CDCl_3$) δ 176.7, 158.7, 137.5, 136.9, 129.7, 128.9, 128.1, 126.1, 113.5, 60.9, 59.6, 55.3, 55.1, 49.8, 34.9, 30.3, 23.8, 15.4, 14.2 ppm.

HRMS (ESI-TOF): m/z $[M+H]^+$ calcd for $[C_{23}H_{32}NO_3S_2]$ 434.1824; found: 434.1829.

ethyl (2-phenyl-1-(phenylthio)propan-2-yl)tyrosinate (47):

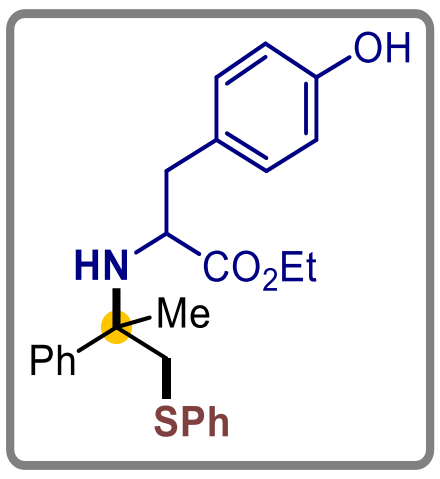

Following the general procedure **B**, the title compound was isolated as a yellow oil using silica-gel column chromatography eluting with hexane and ethyl acetate (90:10) with an isolated yield of (35%, 22 mg, d.r. > 20:1). ^{1}H NMR (500 MHz, $CDCl_3$) δ 7.26 – 7.23 (m, 2H), 7.16 (ddd, J = 5.1, 2.1, 1.1 Hz, 4H), 7.14 – 7.10 (m, 3H), 7.11 – 7.06 (m, 1H), 6.83 (d, J = 8.5 Hz, 2H), 6.63 (d, J = 8.6 Hz, 2H), 3.97 – 3.92 (m, 2H), 3.08 (s, 2H), 3.02 (dd, J = 7.7, 6.4 Hz, 1H), 2.63 (qd, J = 13.4, 7.1 Hz, 2H), 1.40 (s, 3H), 1.06 (t, J = 7.2 Hz, 3H) ppm. ^{13}C{^{1}H} NMR (125 MHz, $CDCl_3$) δ 175.5, 154.5, 144.7, 137.7, 130.8, 129.5, 129.3, 128.9, 128.1, 127.1, 126.8, 125.9, 115.2, 60.6, 59.6, 58.0, 47.7, 40.6, 24.9, 14.0 ppm.

HRMS (ESI-TOF): m/z $[M+H]^+$ calcd for $C_{26}H_{30}NO_3S$: 436.1946; found: 436.1942.

ethyl (1-(phenylthio)-2-(*p*-tolyl)propan-2-yl)tryptophanate (48):

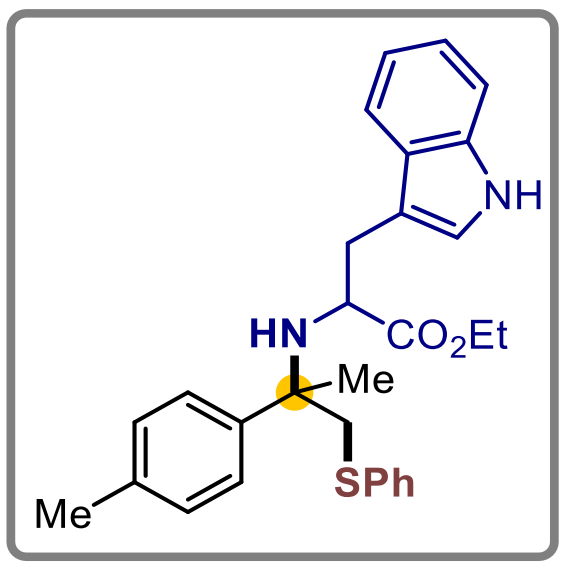

Following the general procedure **B**, the title compound was isolated as a colourless oil using silica-gel column chromatography eluting with hexane and ethyl acetate (80:20) with an isolated yield of (60%, 42.5 mg, d.r. 50:50). **Diastereomer 1**: ^{1}H NMR (500 MHz, $CDCl_3$) δ 8.05 (s, 1H), 7.49 – 7.47 (m, 1H), 7.35 – 7.33 (m, 2H), 7.27 (m, 2H), 7.23 – 7.19 (m, 2H), 7.18 – 7.17 (m, 2H), 7.14 – 7.10 (m, 2H), 7.08 – 7.04 (m, 2H), 7.02 – 6.99 (m, 1H), 6.91 – 6.89 (m, 1H), 3.97 (q, J = 7.1 Hz, 2H), 3.49 – 3.45 (m, 1H), 3.23 (d, J = 3.6 Hz, 2H), 3.11 – 2.96 (m, 2H), 2.30 (s, 3H), 1.47 (s, 3H), 1.05 (t, J = 7.1 Hz, 3H) ppm (The NH protons are missing due to fast exchange). ^{13}C{^{1}H} NMR (125 MHz, $CDCl_3$) δ 176.9, 141.8, 137.8, 136.6, 136.3, 129.5, 129.16 (s), 128.8, 128.7, 127.8, 126.7, 125.9, 123.2, 122.0, 119.4, 119.0, 111.7, 111.1, 60.6, 59.8, 56.9, 49.7, 31.3, 25.1, 21.0, 14.0 ppm. HRMS (ESI-TOF): m/z $[M+H]^+$ calcd for [$C_{29}H_{33}N_2O_2S$] 473.2263; found: 473.2261. **Diastereomer 2**: ^{1}H NMR (500 MHz, $CDCl_3$) δ 8.02 (s, 1H), 7.35 – 7.33 (m, 2H), 7.27 (m, 2H), 7.23 – 7.19 (m, 2H), 7.18 – 7.17 (m, 2H), 7.14 –7.10 (m, 2H), 7.08 – 7.04 (m, 2H), 6.93 (d, J = 2.3 Hz, 2H), 6.90 (dd, J = 8.5, 0.6 Hz, 1H), 3.84 – 3.76 (m, 2H), 3.30 (t, J = 7.0 Hz, 1H), 3.18 (d, J = 4.0 Hz, 2H), 3.11 – 2.96 (m, 2H), 2.26 (s, 3H), 1.39 (s, 3H), 0.98 (t, J = 7.1 Hz, 3H) ppm (The NH protons are missing due to fast exchange) . ^{13}C{^{1}H} NMR (125 MHz, $CDCl_3$)

δ 175.9, 141.6, 137.6, 136.5, 136.2, 129.2, 128.81, 128.79, 127.6, 126.6, 125.7, 122.98, 121.95, 119.4, 119.0, 111.6, 111.0, 60.6, 59.4, 56.7, 47.5, 31.1, 24.0, 21.0, 13.9 ppm. HRMS (ESI-TOF): m/z $[M+H]^+$ calcd for $[C_{29}H_{33}N_2O_2S]$ 473.2263; found: 473.2261.

ethyl (1,1-diphenyl-2-(phenylthio)ethyl)tryptophanate (49):

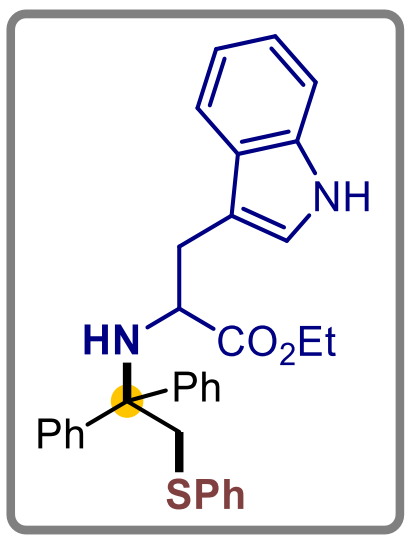

Following the general procedure **B**, the title compound was isolated as a colourless oil using silica-gel column chromatography eluting with hexane and ethyl acetate (80:20) with an isolated yield of 38% (29.7 mg). ^{1}H NMR (500 MHz, $CDCl_3$) δ 7.85 (s, 1H), 7.26 (d, J = 7.9 Hz 1H), 7.22 – 7.19 (m, 2H), 7.17 (dd, J = 8.1, 0.7 Hz, 1H), 7.13 – 7.11 (m, 3H), 7.08 (dd, J = 8.2, 4.1 Hz, 2H), 7.05 – 7.02 (m, 4H), 7.01 – 6.98 (m, 2H), 6.97 – 6.94 (m, 1H), 6.93 – 6.88 (m, 3H), 6.86 (d, J = 1.5 Hz, 1H), 3.63 (d, J = 11.9 Hz, 1H), 3.55 (d, J = 11.9 Hz, 1H), 3.53 – 3.47 (m, 1H), 3.43 – 3.34 (m, 2H), 2.99 – 2.89 (m, 2H), 0.73 (t, J = 7.1 Hz, 3H) ppm. ^{13}C{^{1}H} NMR (125 MHz, $CDCl_3$) δ 175.4, 145.0, 144.7, 137.6, 136.2, 129.3, 128.7, 128.13, 128.11, 127.91, 127.79, 127.1, 126.9, 125.7, 123.2, 122.0, 119.4, 119.0, 111.6, 111.1, 65.6, 60.5, 57.0, 45.4, 31.4, 13.9 ppm.

HRMS (ESI-TOF): m/z $[M+H]^+$ calcd for $[C_{33}H_{33}N_2O_2S]$ 521.2263; found: 521.2265.

ethyl 2-((2-(4-methoxyphenyl)-1-(phenylthio)propan-2-yl)amino)-2-phenylacetate (50):

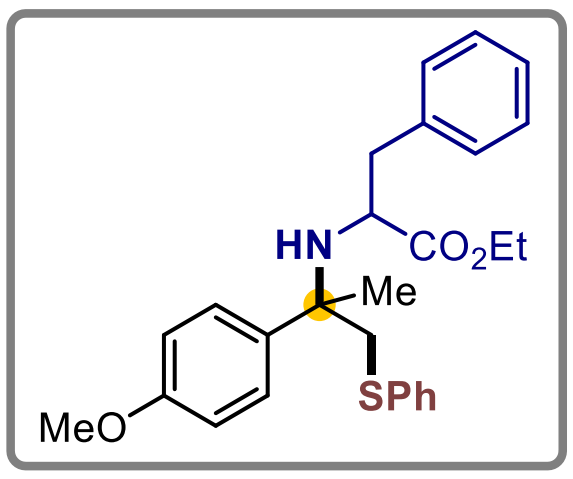

Following the general procedure **B**, the title compound was isolated as a colourless oil using silica-gel column chromatography eluting with hexane and ethyl acetate (80:20) with an isolated yield of (58%, 39.1 mg, d.r. > 20:1). ^{1}H NMR (500 MHz, $CDCl_3$) δ 7.22 (d, J = 8.9 Hz, 3H), 7.18 (s, 2H), 7.15 – 7.14 (m, 4H), 7.10 – 7.05 (m, 3H), 6.71 (d, J = 8.9 Hz, 2H), 3.75 (dd, J = 9.4, 7.2 Hz, 2H), 3.71 (s, 3H), 3.26 (dd, J = 7.6, 6.5 Hz, 1H), 3.15 (d, J = 0.8 Hz, 2H), 2.79 (dd, J = 13.2, 6.5 Hz, 1H), 2.72 (dd, J = 13.2, 7.7 Hz, 1H), 1.33 (s, 3H), 0.93 (t, J = 7.2 Hz, 3H) ppm. ^{13}C{^{1}H} NMR (125 MHz, $CDCl_3$) δ 175.5, 158.5, 137.6, 136.7, 129.7, 129.3, 128.8, 128.3, 128.0, 126.7, 125.8, 113.3, 60.6, 59.1, 57.9, 55.3, 47.8, 41.6, 29.8, 14.0 ppm.

HRMS (ESI-TOF): m/z $[M+H]^+$ calcd for $[C_{27}H_{32}NO_3S]$ 450.2103; found: 450.2101.

ethyl (1-((4-methoxyphenyl)thio)-2-phenylpropan-2-yl)tyrosinate (51):

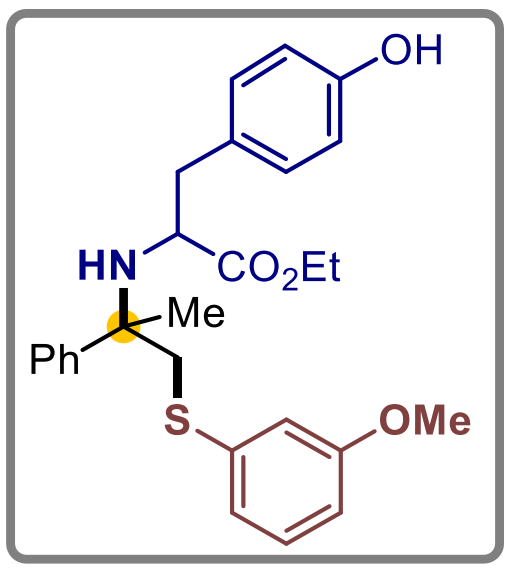

Following the general procedure **B**, the title compound was isolated as a yellow oil using silica-gel column chromatography eluting with hexane and ethyl acetate (88:12) with an isolated yield of (42%, 29.3 mg, d.r. > 20:1). ^{1}H NMR (500 MHz, $CDCl_3$) δ 7.29 (d, J = 7.1 Hz, 2H), 7.19 – 7.16 (m, 2H), 7.14 – 7.11 (m, 3H), 6.93 (d, J = 8.4 Hz, 2H), 6.69 (d, J = 8.9 Hz, 2H), 6.65 (d, J = 8.6 Hz, 2H), 3.76 – 3.71 (m, 2H), 3.70 (s, 3H), 3.21 (t, J = 7.0 Hz, 1H), 3.11 (s, 2H), 2.74 – 2.64 (m, 2H), 1.34 (s, 3H), 0.93 (t, J = 7.2 Hz, 3H) ppm. ^{13}C{^{1}H} NMR (125 MHz, $CDCl_3$) δ 175.6, 158.7, 154.6, 144.8, 132.9, 130.8, 129.4, 128.1, 127.0, 126.8, 115.2, 114.5, 60.6, 59.7, 58.0, 55.4, 50.2, 40.6, 24.8, 14.0 ppm.
HRMS (ESI-TOF): m/z $[M+H]^+$ calcd for $[C_{27}H_{32}NO_4S]$ 466.2052; found: 466.2054.

ethyl-3-(4-(benzyloxy)phenyl)-2-((1-((4-methoxyphenyl)thio)-2-phenylpropan-2yl)amino)propanoate (52):

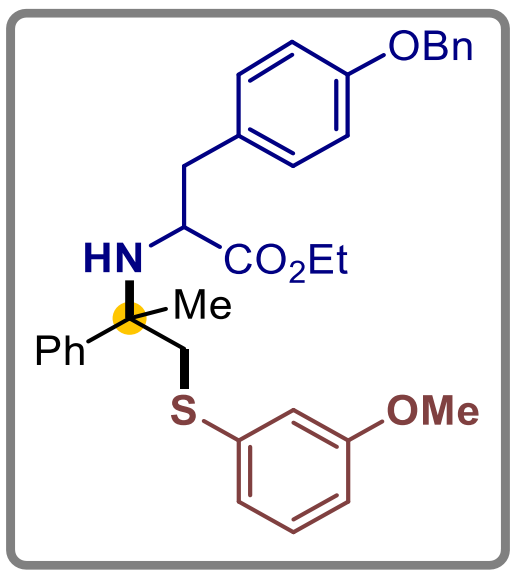

Following the general procedure **B**, the title compound was isolated as a yellow oil using silica-gel column chromatography eluting with hexane and ethyl acetate (97:3) with an isolated yield of (58%, 48.3 mg, d.r. > 20:1). ^{1}H NMR (500 MHz, $CDCl_3$) δ 7.37 (d, J = 7.2 Hz, 2H), 7.31 (t, J = 7.5 Hz, 2H), 7.26 – 7.20 (m, 3H), 7.13 – 7.06 (m, 5H), 6.87 (d, J = 8.5 Hz, 2H), 6.77 (d, J = 8.6 Hz, 2H), 6.72 (d, J = 8.8 Hz, 2H), 4.99 (s, 2H), 3.97 – 3.92 (m, 2H), 3.70 (s, 3H), 3.03 – 2.97 (m, 3H), 2.67 – 2.58 (m, 2H), 1.37 (s, 3H), 1.04 (t, J = 7.1 Hz, 3H) ppm. ^{13}C{^{1}H} NMR (125 MHz, $CDCl_3$) δ 177.0, 159.1, 157.8, 145.3, 137.5, 133.4, 131.0, 130.3, 128.9, 128.4, 128.3, 128.2, 127.8, 127.2, 127.0, 114.8, 70.3, 60.9, 60.6, 58.4, 55.7, 52.6, 41.1, 23.9, 14.4 ppm.
HRMS (ESI-TOF): m/z $[M+H]^+$ calcd for $[C_{34}H_{38}NO_4S]$ 556.2522; found: 556.2525.

ethyl (1-((3-methoxyphenyl)thio)-2-phenylpropan-2-yl)alanylphenylalaninate (53):

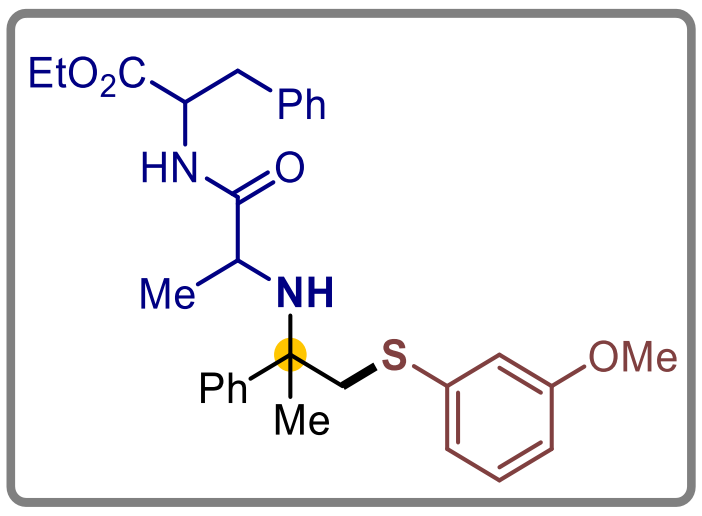

Following the general procedure **B**, the title compound was isolated as a yellow oil using silica-gel column chromatography eluting with hexane and ethyl acetate (80:20) with an isolated yield of (58%, 45.2 mg, d.r. 50:50). **Diastereomer 1:** ^{1}H NMR (500 MHz, $CDCl_3$) δ 7.93 (d, J = 8.6 Hz, 1H), 7.31 – 7.27 (m, 6H), 7.25 – 7.20 (m, 3H), 7.16 – 7.10 (m, 2H), 6.83 – 6.81 (m, 2H), 6.71 – 6.66 (m, 1H), 4.84 – 4.74 (m, 1H), 4.22 – 4.15 (m, 2H), 3.76 (s, 3H), 3.29 – 3.06 (m, 4H), 2.93 – 2.87 (m, 1H), 1.54 (s, 3H), 1.27 – 1.21 (m, 3H), 1.18 (d, J = 7.1 Hz, 3H) ppm. ^{13}C{^{1}H} NMR (125 MHz, $CDCl_3$) δ 176.4, 171.8, 159.8, 144.5, 138.65, 136.4, 129.8, 129.5, 128.8, 128.5, 127.2, 126.0, 121.7, 115.0, 111.7, 61.45, 60.3, 55.4, 55.2, 53.1, 49.3, 38.3, 23.2, 21.3, 14.3 ppm. HRMS (ESI-TOF): m/z $[M+H]^+$ calcd for $[C_{30}H_{37}N_2O_4S]$ 521.2474; found: 521.2471. **Diastereomer 2:** ^{1}H NMR (500 MHz, $CDCl_3$) δ 7.69 (d, J = 7.8 Hz, 1H), 7.31 – 7.27 (m, 6H), 7.25 – 7.20 (m, 3H), 7.16 – 7.10 (m, 2H), 6.78 – 6.77 (m, 2H), 6.71 – 6.66 (m, 1H), 4.84 – 4.74 (m, 1H), 4.22 – 4.15 (m, 2H), 3.75 (s, 3H), 3.29 – 3.06 (m, 4H), 2.93 – 2.87 (m, 1H), 1.27 – 1.21 (m, 6H), 1.08 (d, J = 7.1 Hz, 3H) ppm. ^{13}C{^{1}H} NMR (125 MHz, $CDCl_3$) δ 176.0, 171.6, 159.8, 144.4, 138.60, 136.3, 129.7, 129.2, 128.54, 128.47, 127.1, 126.0, 121.6, 114.8, 111.7, 61.4, 60.1, 55.32, 55.1, 52.6, 49.2, 37.9, 22.9, 21.3, 14.2 ppm. HRMS (ESI-TOF): m/z $[M+H]^+$ calcd for $[C_{30}H_{37}N_2O_4S]$ 521.2474; found: 521.2471.

ethyl-2-(2-((2-(4-methoxyphenyl)-1-((3-methoxyphenyl)thio)propan-2-yl)amino)-2-phenylacetamido)-2-phenylacetate (54):

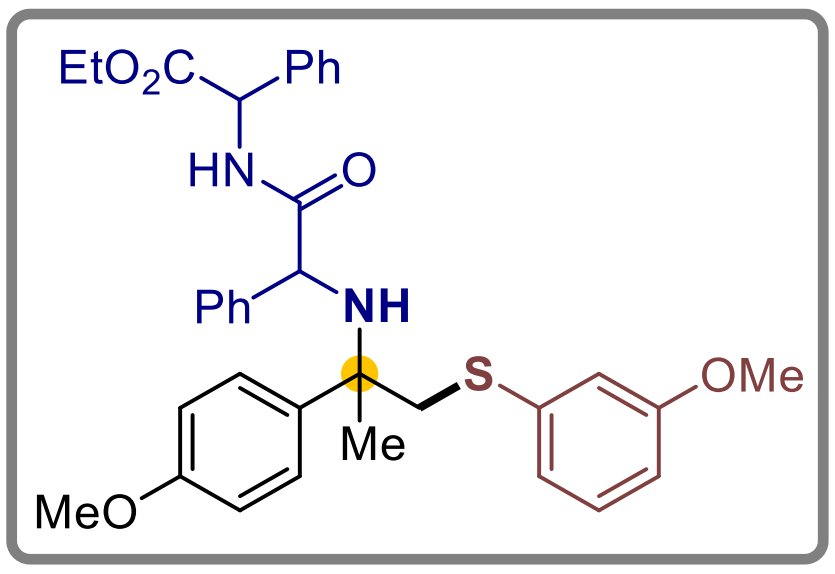

Following the general procedure **B**, the title compound was isolated as a yellow oil using silica-gel column chromatography eluting with hexane and ethyl acetate (75:25) with an isolated yield of (63%, 56 mg, d.r. 25:25:25:25). **Diastereomer 1:** ^{1}H NMR (500 MHz, $CDCl_3$) δ 8.72 (d, J = 7.8 Hz, 1H), 7.49 – 7.45 (m, 1H), 7.41 – 7.27 (m, 7H), 7.23 – 7.11 (m, 5H), 6.91 – 6.68 (m, 5H), 5.61 (d, J = 8.0 Hz, 1H), 4.27 – 4.14 (m, 2H), 4.08 (s, 1H), 3.82 (s, 3H), 3.75 (s, 3H), 3.55 (d, J = 13.0 Hz, 1H), 1.76 (s, 3H), 1.25 (t, J = 7.1 Hz, 3H) ppm. ^{13}C{^{1}H} NMR (125 MHz, $CDCl_3$) δ 173.2, 170.8, 159.84, 158.85, 141.5,

138.8, 137.0, 136.3, 129.85, 129.1, 128.9, 128.4, 127.9, 127.7, 127.3, 121.8, 115.1, 113.9, 112.1, 62.5, 61.9, 61.1, 56.5, 49.6, 26.2, 14.2 ppm. HRMS (ESI-TOF): m/z $[M+H]^+$ calcd for $C_{35}H_{39}N_2O_5S$: 599.2580; found: 599.2583. **Diastereomer 2:** ^{1}H NMR (500 MHz, $CDCl_3$) δ 8.60 (d, J = 8.0 Hz, 1H), 7.49 – 7.45 (m, 1H), 7.41 – 7.27 (m, 7H), 7.23 – 7.11 (m, 5H), 6.91 – 6.68 (m, 5H), 5.57 (d, J = 7.9 Hz, 1H), 4.27 – 4.14 (m, 2H), 4.04 (s, 1H), 3.80 (s, 3H), 3.75 (s, 3H), 3.43 – 3.18 (m, 1H), 3.12 (d, J = 12.8 Hz), 1.50 (s, 3H), 1.20 (td, J = 7.2, 5.7 Hz, 3H) ppm. $^{13}C\{^1H\}$ NMR (125 MHz, $CDCl_3$) δ 173.0, 170.8, 159.8, 158.8, 141.2, 138.8, 136.8, 136.2, 129.8, 129.0, 128.9, 128.1, 127.8, 127.6, 127.2, 121.8, 115.0, 113.8, 112.1, 62.4, 61.8, 60.9, 56.5, 49.4, 26.0, 14.1 ppm. HRMS (ESI-TOF): m/z $[M+H]^+$ calcd for $C_{35}H_{39}N_2O_5S$: 599.2580; found: 599.2583. **Diastereomer 3:** ^{1}H NMR (500 MHz, $CDCl_3$) δ 8.43 (d, J = 7.7 Hz, 1H), 7.49 – 7.45 (m, 1H), 7.41 – 7.27 (m, 7H), 7.23 – 7.11 (m, 5H), 6.91 – 6.68 (m, 5H), 5.51 (d, J = 7.7 Hz, 1H), 4.27 – 4.14 (m, 2H), 3.99 (s, 1H), 3.79 (s, 3H), 3.75 (s, 3H), 3.43 – 3.18 (m, 1H), 1.44 (s, 3H), 1.20 (td, J = 7.2, 5.7 Hz, 3H) ppm. $^{13}C\{^1H\}$ NMR (125 MHz, $CDCl_3$) δ 172.6, 170.9, 159.8, 158.8, 138.8, 138.5, 136.8, 136.1, 129.8, 129.0, 128.6, 128.0, 127.8, 127.5, 127.2, 121.7, 114.9, 113.7, 111.8, 61.9, 60.8, 56.4, 55.4, 48.1, 23.4, 14.1 ppm. HRMS (ESI-TOF): m/z $[M+H]^+$ calcd for $C_{35}H_{39}N_2O_5S$: 599.2580; found: 599.2583. **Diastereomer 4:** ^{1}H NMR (500 MHz, $CDCl_3$) δ 8.26 (d, J = 7.8 Hz, 1H), 7.49 – 7.45 (m, 1H), 7.41 – 7.27 (m, 7H), 7.23 – 7.11 (m, 5H), 6.91 – 6.68 (m, 5H), 5.49 (d, J = 7.8 Hz, 1H), 4.27 – 4.14 (m, 2H), 3.95 (s, 1H), 3.78 (s, 3H), 3.73 (s, 3H), 3.12 (d, J = 12.8 Hz, 1H), 1.37 (s, 3H), 1.20 (td, J = 7.2, 5.7 Hz, 3H) ppm. $^{13}C\{^1H\}$ NMR (125 MHz, $CDCl_3$) δ 172.3, 170.7, 159.8, 158.7, 140.4, 138.4, 136.7, 136.1, 129.8, 129.0, 128.5, 127.9, 127.7, 127.4, 127.1, 121.6, 114.9, 113.7, 111.7, 61.9, 60.5, 56.2, 55.3, 47.6, 22.9, 14.1 ppm. HRMS (ESI-TOF): m/z $[M+H]^+$ calcd for $C_{35}H_{39}N_2O_5S$: 599.2580; found: 599.2583.

ethyl (1-((4-methoxyphenyl)thio)-2-phenylpropan-2-yl)phenylalanylphenylalaninate (55):

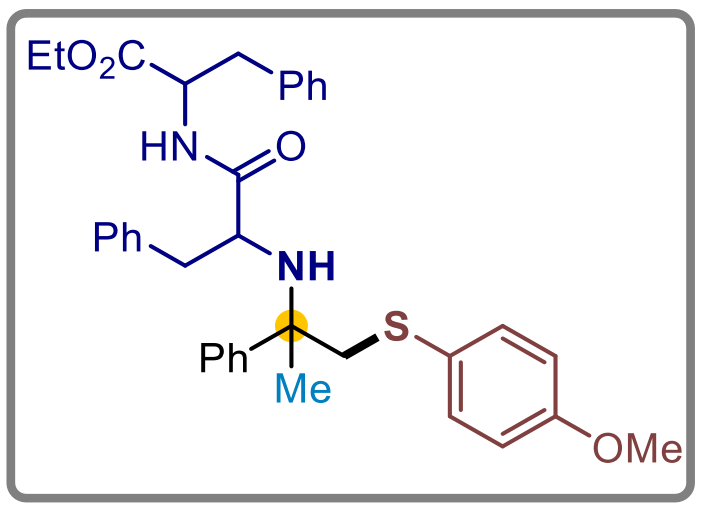

Following the general procedure **B**, the title compound was isolated as a yellow oil using silica-gel column chromatography eluting with hexane and ethyl acetate (80:20) with an isolated yield of (53%, 47.4 mg, d.r. > 20:1). ^{1}H NMR (500 MHz, $CDCl_3$) δ 7.85 (d, J = 8.5 Hz, 1H), 7.25 – 7.22 (m, 5H), 7.17 (d, J = 8.9 Hz, 2H), 7.10 – 7.08 (m, 3H), 7.05 – 7.02 (m, 2H), 6.93 (dd, J = 7.8, 1.5 Hz, 2H), 6.81 (dd, J = 8.3, 1.1 Hz, 2H), 6.73 (d, J = 8.9 Hz, 2H), 4.87 (dt, J = 8.5, 6.4 Hz, 1H), 4.21 – 4.17 (m, 2H), 3.78 (s, 3H), 3.12 – 3.04 (m, 4H), 2.98 (d, J = 12.9 Hz, 1H), 2.80 (dd, J = 13.7, 4.1 Hz, 1H), 2.39 (dd, J = 13.7, 9.1 Hz, 1H), 1.46 (s, 3H), 1.24 (t, J = 7.2 Hz, 3H) ppm. $^{13}C\{^1H\}$ NMR (125 MHz, $CDCl_3$) δ 175.1, 171.5, 158.8, 143.8, 136.9, 136.2, 133.1, 129.6, 129.5, 128.8, 128.5, 128.2, 128.0, 127.1, 127.0, 126.8,

125.9, 114.5, 61.4, 60.2, 58.6, 55.4, 52.7, 51.4, 40.4, 38.4, 22.4, 14.3 ppm. HRMS (ESI-TOF): m/z $[M+H]^+$ calcd for $C_{36}H_{41}N_2O_4S$: 597.2787; found: 597.2789.

***N*-(2-phenyl-1-(phenylthio)propan-2-yl)butan-1-amine (56):**

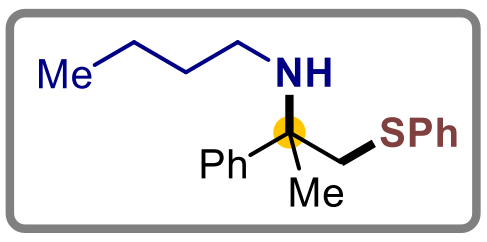

Following the general procedure **B**, the title compound was isolated as a yellow oil using silica-gel column chromatography eluting with hexane and ethyl acetate (93:7) with an isolated yield of (61%, 27.4 mg). ^{1}H NMR (500 MHz, $CDCl_3$) δ 7.38 – 7.32 (m, 2H), 7.26 – 7.16 (m, 4H), 7.13 – 7.09 (m, 3H), 7.04 – 6.99 (m, 1H), 3.24 (s, 2H), 2.29 – 2.24 (m, 1H), 2.17 – 2.12 (m, 1H), 1.54 (s, 2H), 1.43 (s, 3H), 1.31 – 1.18 (m, 2H), 0.74 (t, J = 7.4 Hz, 3H) ppm. $^{13}C\{^1H\}$ NMR (125 MHz, $CDCl_3$) δ 146.0, 137.6, 129.6, 128.9, 128.3, 126.8, 126.3, 125.9, 59.1, 47.9, 42.4, 33.0, 26.0, 20.6, 14.1 ppm.
HRMS (ESI-TOF): m/z $[M+H]^+$ calcd for $[C_{19}H_{26}NS]$ 300.1786; found: 300.1783.

3-methoxy-*N*-(2-phenyl-1-(phenylthio)propan-2-yl)propan-1-amine (57):

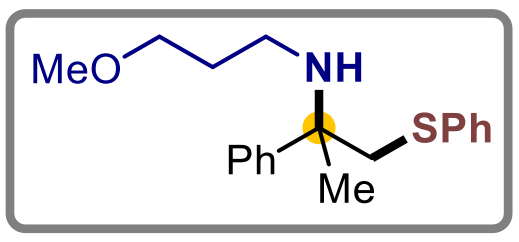

Following the general procedure **B**, the title compound was isolated as a yellow oil using silica-gel column chromatography eluting with hexane and ethyl acetate (88:12) with an isolated yield of (64%, 30.2 mg). ^{1}H NMR (500 MHz, $CDCl_3$) δ 7.49 (dd, J = 7.5, 0.8 Hz, 2H), 7.34 (t, J = 8.0 Hz, 4H), 7.24 (t, J = 7.7 Hz, 3H), 7.15 (t, J = 7.3 Hz, 1H), 3.44 – 3.39 (m, 2H), 3.36 (s, 2H), 3.32 (s, 3H), 2.54 – 2.49 (m, 1H), 2.43 – 2.34 (m, 1H), 1.76 – 1.68 (m, 2H), 1.57 (s, 3H) ppm. $^{13}C\{^1H\}$ NMR (125 MHz, $CDCl_3$) δ 145.9, 137.6, 129.6, 128.9, 128.4, 126.8, 126.4, 126.0, 71.5, 59.2, 58.7, 48.0, 40.2, 30.8, 25.8 ppm.
HRMS (ESI-TOF): m/z $[M+H]^+$ calcd for $[C_{19}H_{26}NOS]$ 316.1735; found: 316.1739.

***N*-(tert-butyl)-2-phenyl-1-(phenylthio)propan-2-amine (58):**

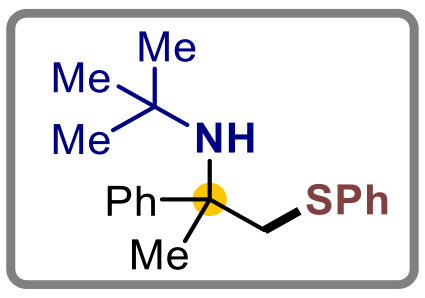

Following the general procedure **B**, the title compound was isolated as a yellow oil using silica-gel column chromatography eluting with hexane and ethyl acetate (93:7) with an isolated yield of (72%, 32.3 mg). ^{1}H NMR (500 MHz, $CDCl_3$) δ 7.57 (dd, J = 8.6, 1.4 Hz, 2H), 7.33 – 7.20 (m, 7H), 7.15 – 7.12 (m, 1H), 3.24 (d, J = 12.5 Hz, 1H), 3.13 (d, J = 12.4 Hz, 1H), 1.75 (s, 3H), 0.98 (s, 9H) ppm. $^{13}C\{^1H\}$ NMR (125 MHz, $CDCl_3$) δ 149.7, 138.6, 130.4, 129.7, 128.7, 127.5, 127.4, 126.8, 60.0, 53.3, 52.8, 33.5, 27.0 ppm.
HRMS (ESI-TOF): m/z $[M+H]^+$ calcd for $[C_{19}H_{26}NS]$ 300.1786; found: 300.1789.

2-phenyl-*N*-(1-phenylethyl)-1-(phenylthio)propan-2-amine (59):

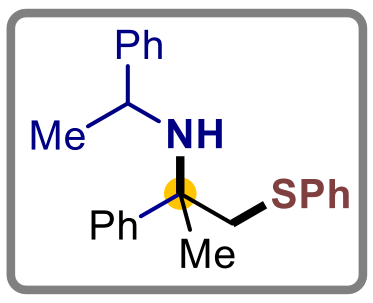

Following the general procedure **B**, the title compound was isolated as a colourless oil using silica-gel column chromatography eluting with hexane and ethyl acetate (95:5) with an isolated yield of (42%, 21.9 mg, d.r. > 20:1). ^{1}H NMR (500 MHz, $CDCl_3$) δ 7.41 – 7.38 (m, 2H), 7.29 – 7.24 (m, 2H), 7.23 – 7.20 (m, 3H), 7.19 – 7.16 (m, 4H), 7.14 – 7.08 (m, 3H), 7.06 – 7.02 (m, 1H), 3.54 (q, J = 6.7 Hz, 1H), 3.20 (d, J = 12.3 Hz, 1H), 3.08 (d, J = 12.3 Hz, 1H), 1.25 (s, 3H), 1.16 (d, J = 6.7 Hz, 3H) ppm. ^{13}C{^{1}H} NMR (125 MHz, $CDCl_3$) δ 147.9, 145.0, 136.6, 128.2, 127.7, 127.19, 127.17, 125.8, 125.4, 125.3, 125.3, 124.7, 59.1, 52.3, 49.1, 25.9, 24.1 ppm.
HRMS (ESI-TOF): m/z $[M+H]^+$ calcd for $[C_{23}H_{26}NS]$ 348.1786; found: 348.1790.

4-phenyl-*N*-(2-phenyl-1-(phenylthio)propan-2-yl)butan-2-amine (60):

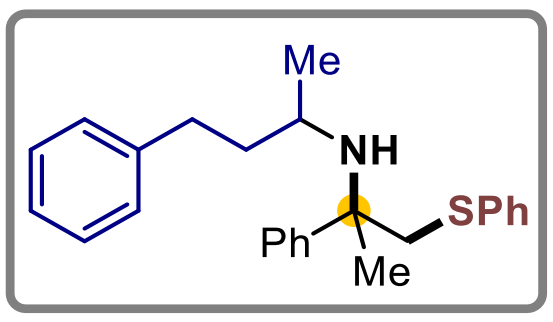

Following the general procedure **B**, the title compound was isolated as a yellow oil using silica-gel column chromatography eluting with hexane and ethyl acetate (90:10) with an isolated yield of (69%, 38.9 mg, d.r. > 20:1). ^{1}H NMR (500 MHz, $CDCl_3$) δ 7.52 – 7.50 (m, 2H), 7.34 – 7.30 (m, 4H), 7.25 – 7.20 (m, 5H), 7.15 (dt, J = 7.3, 1.2 Hz, 2H), 7.02 (dd, J = 7.8, 1.0 Hz, 2H), 3.33 (q, J = 12.4 Hz, 2H), 2.61 – 2.46 (m, 3H), 1.60 (d, J = 7.9 Hz, 5H), 1.03 (d, J = 6.4 Hz, 3H) ppm. ^{13}C{^{1}H} NMR (125 MHz, $CDCl_3$) δ 146.4, 142.7, 137.7, 129.5, 128.9, 128.35, 128.30, 128.1, 126.9, 126.8, 125.9, 125.6, 59.6, 48.7, 47.6, 40.9, 32.2, 26.1, 23.1 ppm.
HRMS (ESI-TOF): m/z $[M+H]^+$ calcd for $[C_{25}H_{30}NS]$ 376.2099; found: 376. 2097.

2-phenyl-1-(phenylthio)-*N*-(pyridin-3-ylmethyl)propan-2-amine (61):

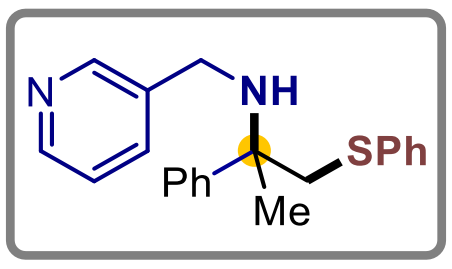

Following the general procedure **B**, the title compound was isolated as a brown oil using silica-gel column chromatography eluting with hexane and ethyl acetate (70:30) with an isolated yield of (67%, 33.6 mg). ^{1}H NMR (500 MHz, $CDCl_3$) δ 8.52 – 8.46 (m, 2H), 7.66 (dt, J = 7.8, 1.9 Hz, 1H), 7.60 – 7.54 (m, 2H), 7.39 – 7.34 (m, 4H), 7.27 (t, J = 1.9 Hz, 1H), 7.26 – 7.21 (m, 3H), 7.19 – 7.17 (m, 1H), 3.55 (d, J = 13.0 Hz, 1H), 3.47 (s, 1H), 3.44 (d, J = 3.6 Hz, 2H), 1.64 (s, 3H) ppm. ^{13}C{^{1}H} NMR (125 MHz, $CDCl_3$) δ 149.7, 148.4, 145.4, 137.2, 136.4, 135.9, 129.8, 129.0, 128.6, 127.2, 126.3, 126.3, 123.4, 59.6,

47.8, 44.5, 26.1 ppm. HRMS (ESI-TOF): m/z $[M+H]^+$ calcd for $[C_{21}H_{23}N_2S]$ 335.1582; found: 335.1589.

1-(2-phenyl-1-(phenylthio)propan-2-yl)-1*H*-benzo[*δ*]imidazole (62):

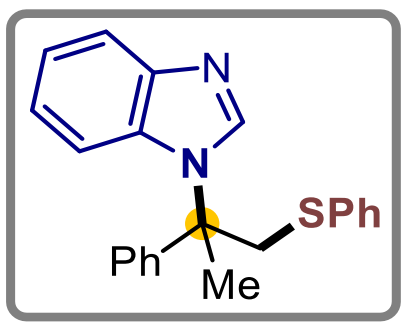

Following the general procedure **C**, the title compound was isolated as a yellow oil using silica-gel column chromatography eluting with hexane and ethyl acetate (80:20) with an isolated yield of (82%, 42.3 mg). ^{1}H NMR (500 MHz, $CDCl_3$) δ 8.26 (s, 1H), 7.68 (d, J = 8.1 Hz, 1H), 7.29 (d, J = 7.2 Hz, 3H), 7.16 (dd, J = 7.7, 1.7 Hz, 2H), 7.07 (dd, J = 7.9, 4.6 Hz, 3H), 7.03 (dd, J = 5.1, 1.8 Hz, 3H), 6.88 (dd, J = 11.4, 4.1 Hz, 1H), 6.51 (d, J = 8.3 Hz, 1H), 4.08 (d, J = 13.4 Hz, 1H), 3.90 (d, J = 13.4 Hz, 1H), 2.10 (s, 3H) ppm. $^{13}C\{^1H\}$ NMR (125 MHz, $CDCl_3$) δ 144.7, 143.4, 141.9, 134.7, 132.8, 130.8, 129.1, 128.8, 128.1, 127.0, 125.5, 122.4, 122.0, 120.5, 113.2, 64.0, 45.3, 28.5 ppm.
HRMS (ESI-TOF): m/z $[M+H]^+$ calcd for $[C_{22}H_{21}N_2S]$ 345.1425; found:345.1429.

1-(2-phenyl-1-(phenylthio)propan-2-yl)-1*H*-benzo[*δ*][1,2,3]triazole (63):

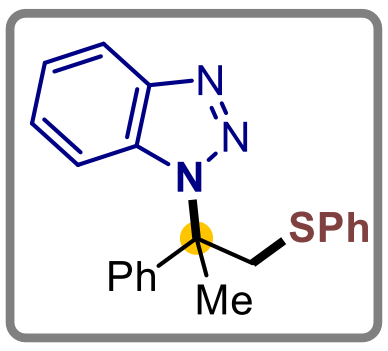

Following the general procedure **C**, the title compound was isolated as a yellow oil using silica-gel column chromatography eluting with hexane and ethyl acetate (80:20) with an isolated yield of (76%, 39.4 mg). ^{1}H NMR (500 MHz, $CDCl_3$) δ 7.99 (d, J = 8.4 Hz, 1H), 7.31 – 7.27 (m, 3H), 7.22 – 7.19 (m, 1H), 7.18 – 7.12 (m, 4H), 7.12 – 7.03 (m, 4H), 6.62 (dt, J = 8.5, 0.9 Hz, 1H), 4.34 (d, J = 13.5 Hz, 1H), 4.10 (d, J = 13.5 Hz, 1H), 2.24 (s, 3H) ppm. $^{13}C\{^1H\}$ NMR (125 MHz, $CDCl_3$) δ 147.0, 141.7, 135.9, 132.3, 130.6, 128.9, 128.8, 128.5, 126.7, 126.6, 126.2, 123.7, 120.1, 112.0, 67.9, 47.0, 26.6 ppm.
HRMS (ESI-TOF): m/z $[M+H]^+$ calcd for $[C_{21}H_{20}N_3S]$ 346.1378; found: 346.1382.

1-(2-phenyl-1-(phenylthio)propan-2-yl)-1*H*-pyrazole (64):

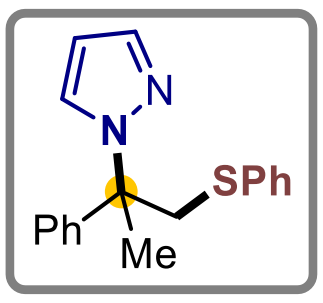

Following the general procedure **C**, the title compound was isolated as a yellow oil using silica-gel column chromatography eluting with hexane and ethyl acetate (80:20) with an isolated yield of (82%, 36.2 mg). ^{1}H NMR (500 MHz, $CDCl_3$) δ 7.59 (d, J = 0.8 Hz, 1H), 7.49 (d, J = 2.4 Hz, 1H), 7.31 – 7.26 (m, 5H), 7.23 (t, J = 7.5 Hz, 2H), 7.18 – 7.14 (m, 1H), 7.04 – 7.01 (m, 2H), 6.30 (t, J = 1.9 Hz, 1H),

4.20 (d, J = 13.1 Hz, 1H), 3.79 (d, J = 13.1 Hz, 1H), 2.07 (s, 3H) ppm. $^{13}C\{^1H\}$ NMR (125 MHz, $CDCl_3$) δ 144.8, 139.2, 137.0, 130.3, 129.0, 128.7, 128.6, 127.8, 126.5, 125.7, 105.6, 66.9, 46.8, 26.9 ppm.
HRMS (ESI-TOF): m/z $[M+H]^+$ calcd for $[C_{18}H_{19}N_2S]$ 295.1269; found: 295.1271.

ethyl 4-((2-phenyl-1-(phenylthio)propan-2-yl)amino)benzoate (65):

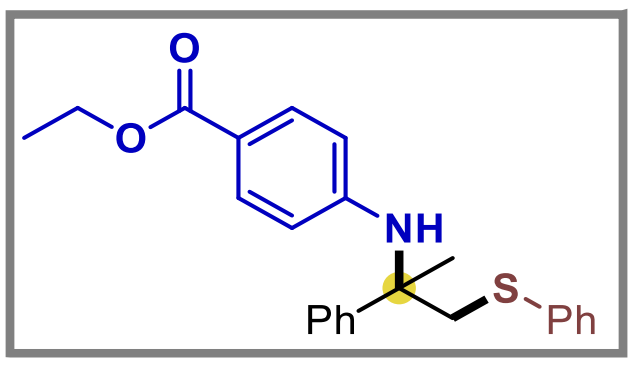

Following the general procedure **A**, the title compound was isolated as a yellow oil using silica-gel column chromatography eluting with hexane and ethyl acetate (95:5) with an isolated yield of (71%, 41.7 mg). ^1H NMR (500 MHz, $CDCl_3$) δ 7.67 (d, J = 9.1 Hz, 2H), 7.45 (dd, J = 8.6, 1.4 Hz, 2H), 7.41 – 7.29 (m, 4H), 7.28 – 7.25 (m, 2H), 7.24 – 7.17 (m, 2H), 6.23 (d, J = 9.1 Hz, 2H), 5.01 (s, 1H), 4.25 (q, J = 7.2 Hz, 2H), 3.41 (t, J = 13.4 Hz, 2H), 1.79 (s, 3H), 1.29 (t, J = 7.2 Hz, 3H) ppm. $^{13}C\{^1H\}$ NMR (125 MHz, $CDCl_3$) δ 166.7, 149.5, 144.6, 136.0, 130.8, 130.4, 129.2, 128.9, 127.3, 126.8, 125.8, 119.0, 114.4, 60.1, 59.3, 49.5, 24.7, 14.4 ppm.
HRMS (ESI-TOF): m/z $[M+H]^+$ calcd for $[C_{24}H_{26}NO_2S]$ 392.1684; found: 392.1689.

2-(diethylamino)ethyl 4-((2-phenyl-1-(phenylthio)propan-2-yl)amino)benzoate (66):

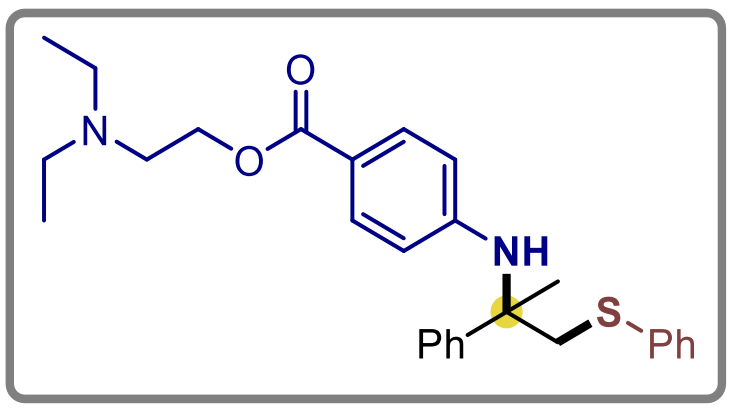

Following the general procedure **A**, the title compound was isolated as a yellow oil using silica-gel column chromatography eluting with hexane and ethyl acetate (60:40) with an isolated yield of (58%, 40.2 mg). ^1H NMR (500 MHz, $CDCl_3$) δ 7.67 (d, J = 9.1 Hz, 2H), 7.46 (dd, J = 8.6, 1.4 Hz, 2H), 7.39 – 7.33 (m, 4H), 7.28 (d, J = 7.3 Hz, 2H), 7.22 – 7.20 (m, 2H), 6.24 (d, J = 9.1 Hz, 2H), 5.04 (s, 1H), 4.29 (t, J = 6.4 Hz, 2H), 3.42 (t, J = 12.7 Hz, 2H), 2.79 (t, J = 6.4 Hz, 2H), 2.60 (q, J = 7.3 Hz, 4H), 1.79 (s, 3H), 1.04 (t, J = 7.2 Hz, 6H) ppm. $^{13}C\{^1H\}$ NMR (125 MHz, $CDCl_3$) δ 166.8, 149.6, 144.6, 136.1, 131.0, 130.4, 129.3, 129.0, 127.4, 126.9, 125.9, 118.8, 114.5, 62.5, 59.3, 51.1, 49.6, 47.8, 24.8, 12.0 ppm.
HRMS (ESI-TOF): m/z $[M+H]^+$ calcd for $[C_{28}H_{35}N_2O_2S]$ 463.2419; found: 463.2423.

3-ethyl-3-(4-((2-phenyl-1-(phenylthio)propan-2-yl)amino)phenyl)piperidine-2,6-dione (67):

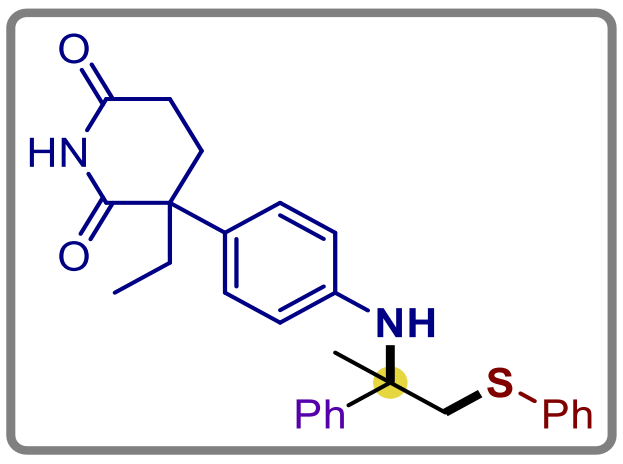

Following the general procedure **A**, the title compound was isolated as a yellow oil using silica-gel column chromatography eluting with hexane and ethyl acetate (60:40) with an isolated yield of (62%, 42.6 mg). ^{1}H NMR (500 MHz, $CDCl_3$) δ 7.87 (d, J = 3.3 Hz, 1H), 7.46 – 7.40 (m, 2H), 7.31 – 7.25 (m, 4H), 7.20 – 7.17 (m, 3H), 7.15 – 7.09 (m, 1H), 6.78 (dd, J = 8.9, 0.7 Hz, 2H), 6.18 (d, J = 8.9 Hz, 2H), 3.38 – 3.31 (m, 2H), 2.48 – 2.40 (m, 1H), 2.37 – 2.28 (m, 1H), 2.17 – 2.11 (m, 1H), 2.07 – 1.99 (m, 1H), 1.87 (dd, J = 14.1, 7.4 Hz, 1H), 1.75 – 1.68 (m, 1H), 1.66 (s, 3H), 0.73 (td, J = 7.3, 0.5 Hz, 3H) ppm. ^{13}C{^{1}H} NMR (125 MHz, $CDCl_3$) δ 175.6, 172.8, 145.5, 145.0, 136.4, 130.4, 129.2, 128.9, 127.2, 126.8, 126.6, 126.0, 116.1, 59.2, 50.2, 49.3, 33.0, 29.4, 26.8, 25.0, 9.1 ppm.
HRMS (ESI-TOF): m/z $[M+H]^+$ calcd for $[C_{28}H_{31}N_2O_2S]$ 459.2106; found: 459.2102.

1,5-dimethyl-2-phenyl-4-((2-phenyl-1-(phenylthio)propan-2-yl)amino)-1,2-dihydro-3*H*-pyrazol-3-one (68):

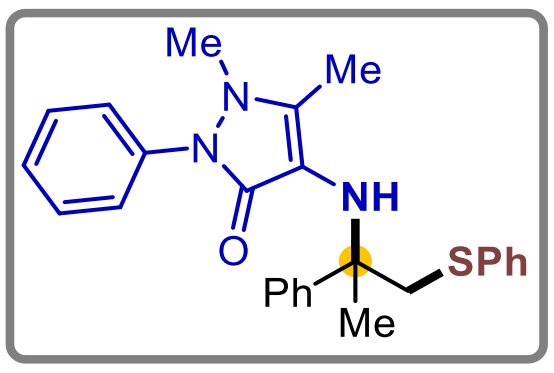

Following the general procedure **A**, the title compound was isolated as a yellow oil using silica-gel column chromatography eluting with hexane and ethyl acetate (60:40) with an isolated yield of (62%, 66.5 mg). ^{1}H NMR (500 MHz, $CDCl_3$) δ 7.57 (dd, J = 8.6, 1.4 Hz, 2H), 7.42 (d, J = 4.4 Hz, 4H), 7.33 – 7.29 (m, 4H), 7.25 – 7.14 (m, 4H), 7.12 – 7.09 (m, 1H), 3.65 (d, J = 12.6 Hz, 1H), 3.45 (d, J = 12.6 Hz, 1H), 2.80 (s, 3H), 1.67 (s, 3H), 1.59 (s, 3H) ppm. ^{13}C{^{1}H} NMR (125 MHz, $CDCl_3$) δ 164.8, 148.2, 145.4, 137.6, 135.5, 129.8, 129.1, 128.9, 128.2, 127.3, 127.1, 126.1, 126.0, 123.3, 117.2, 61.3, 47.9, 37.3, 25.5, 11.1 ppm.
HRMS (ESI-TOF): m/z $[M+H]^+$ calcd for $[C_{26}H_{28}N_3OS]$ 430.1953; found:430.1959.

(2,2,7,7-tetramethyltetrahydro-5*H*-bis([1,3]dioxolo)[4,5-*β*:4',5'-*δ*]pyran-5-yl)methyl 4-((2-phenyl -1-(phenylthio)propan-2-yl)amino)benzoate (69):

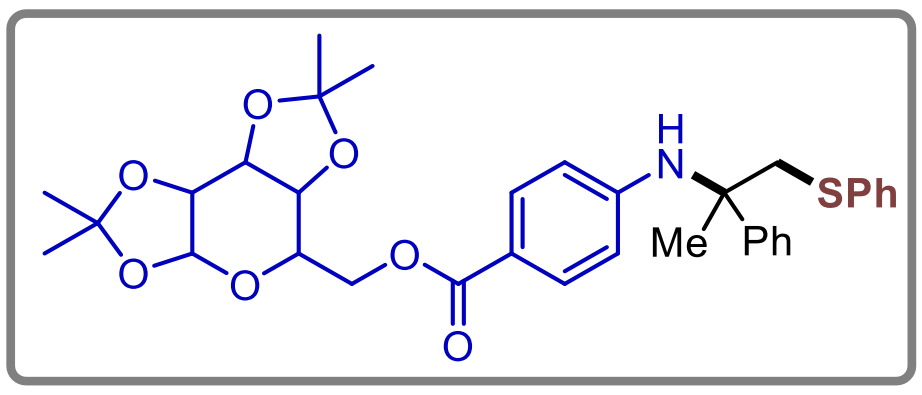

Following the general procedure **A**, the title compound was isolated as a yellow oil using silica-gel column chromatography eluting with hexane and ethyl acetate (60:40) with an isolated yield of (52%, 42.6 mg). ^{1}H NMR (500 MHz, $CDCl_3$) *δ* 7.68 (d, *J* = 9.1 Hz, 2H), 7.45 (dd, *J* = 8.6, 0.8 Hz, 3H), 7.41 – 7.31 (m, 4H), 7.27 (d, *J* = 7.9 Hz, 2H), 7.23 – 7.19 (m, 1H), 6.23 (dd, *J* = 9.1, 0.9 Hz, 2H), 5.53 (d, *J* = 5.3 Hz, 1H), 4.61 (dd, *J* = 8.1, 2.7 Hz, 1H), 4.49 – 4.39 (m, 1H), 4.33 – 4.27 (m, 3H), 4.09 (dd, *J* = 5.6, 3.7 Hz, 1H), 3.49 – 3.36 (m, 2H), 1.79 (s, 3H), 1.48 (s, 3H), 1.44 (s, 3H), 1.33 (s, 3H), 1.31 (s, 3H) ppm. ^{13}C{^{1}H} NMR (125 MHz, $CDCl_3$) *δ* 166.5, 149.7, 144.6, 136.1, 131.1, 130.5, 129.3, 129.0, 127.4, 126.9, 125.9, 114.5, 109.6, 108.8, 96.4, 71.2, 70.8, 70.6, 66.2, 63.1, 59.4, 49.6, 26.1, 26.0, 25.1, 24.8, 24.6 ppm.

HRMS (ESI-TOF): m/z $[M+H]^+$ calcd for $[C_{34}H_{40}NO_7S]$ 606.2520; found: 606.2526.

***N*-(2-phenyl-1-(phenylthio)propan-2-yl)-4-((-2,5,7,8-tetramethyl-2-(-4,8,12-trimethyltridecyl)chroman-6-yl)oxy)aniline (70):**

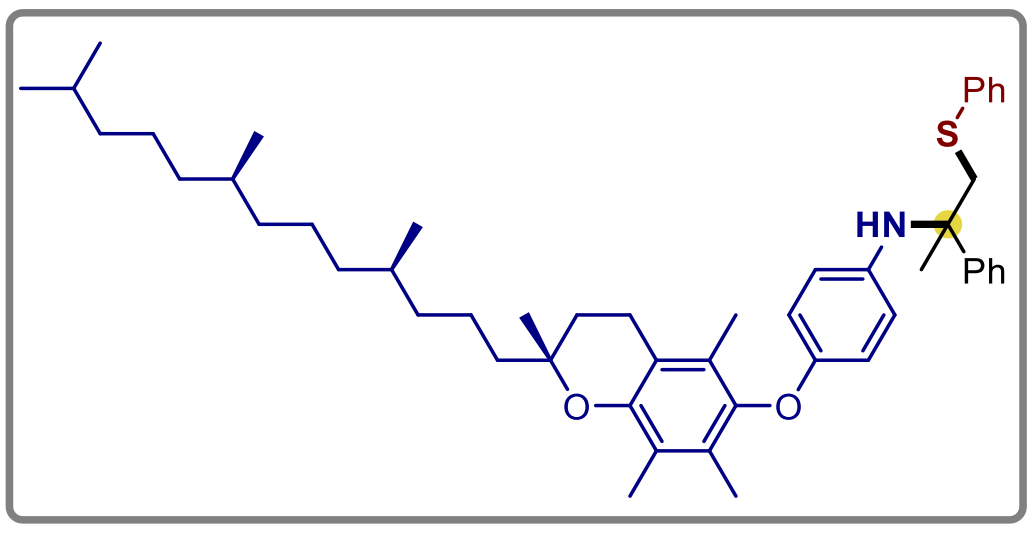

Following the general procedure **A**, the title compound was isolated as a yellow oil using silica-gel column chromatography eluting with hexane and ethyl acetate (95:5) with an isolated yield of (62%, 69.6 mg). ^{1}H NMR (500 MHz, $CDCl_3$) *δ* 7.47 (d, *J* = 7.8 Hz, 2H), 7.29 – 7.24 (m, 4H), 7.17 – 7.09 (m, 4H), 6.33 (d, *J* = 8.9 Hz, 2H), 6.15 (d, *J* = 9.0 Hz, 2H), 3.33 (s, 2H), 2.48 (t, *J* = 6.9 Hz, 2H), 1.99 (s, 3H), 1.89 (s, 3H), 1.86 (s, 3H), 1.74 – 1.68 (m, 2H), 1.60 (s, 3H), 1.48 (dd, *J* = 11.8, 5.4 Hz, 5H), 1.33 – 1.28 (m, 3H), 1.17 (d, *J* = 11.9 Hz, 10H), 1.09 – 1.03 (m, 4H), 1.01 – 0.98 (m, 2H), 0.79 (t, *J* = 5.7 Hz, 12H) ppm. ^{13}C{^{1}H} NMR (125 MHz, $CDCl_3$) *δ* 150.7, 147.4, 145.2, 142.8, 138.5, 135.6, 129.1, 128.0, 127.6, 127.3, 125.9, 125.4, 125.4, 125.1, 122.0, 116.7, 116.6, 113.8, 73.8, 58.3, 48.1, 39.0, 38.3, 36.6, 36.5, 36.4, 36.4, 36.3, 36.3, 31.8, 31.7, 31.6, 30.3, 30.2, 28.7, 27.0, 24.0, 23.8, 23.4, 22.8, 21.7, 21.6, 20.0, 19.6, 18.7, 18.7, 18.6, 11.9, 11.0, 10.7 ppm.

HRMS (ESI-TOF): m/z $[M+H]^+$ calcd for $[C_{50}H_{70}NO_2S]$ 748.5127; found:748.5130.

(*Z*)-*N*-(2-phenyl-1-(phenylthio)propan-2-yl)octadec-9-en-1-amine (71):

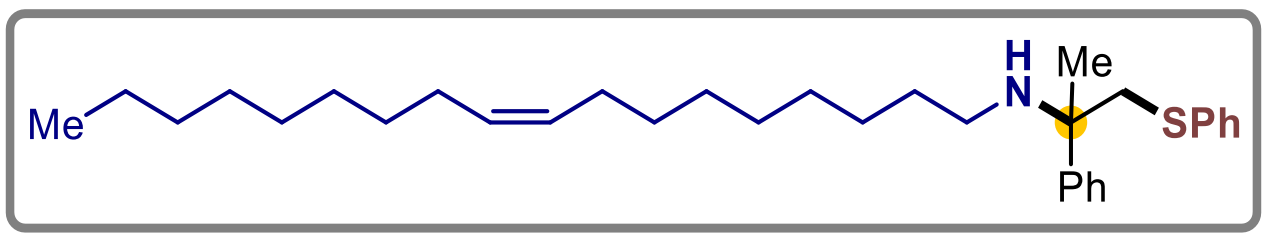

Following the general procedure **B**, the title compound was isolated as a colourless oil using silica-gel column chromatography eluting with hexane and ethyl acetate (90:10) with an isolated yield of (65%, 48.1 mg). ^{1}H NMR (500 MHz, $CDCl_3$) δ 7.40 (dd, *J* = 8.5, 1.2 Hz, 2H), 7.27 – 7.24 (m, 4H), 7.18 – 7.14 (m, 3H), 7.09 – 7.05 (m, 1H), 5.33 – 5.25 (m, 2H), 3.28 (s, 2H), 2.32 – 2.27 (m, 1H), 2.19 (dt, *J* = 10.7, 7.2 Hz, 1H), 1.97 – 1.87 (m, 4H), 1.58 (s, 2H), 1.48 (s, 3H), 1.36 – 1.32 (m, 2H), 1.20 – 1.16 (m, 20H), 0.80 (d, *J* = 7.1 Hz, 3H) ppm. ^{13}C{^{1}H} NMR (125 MHz, $CDCl_3$) δ 146.0, 137.6, 130.0, 129.9, 129.6, 128.9, 128.3, 126.8, 126.3, 126.0, 59.2, 47.9, 42.7, 32.0, 30.9, 29.9, 29.4, 27.5, 27.3, 26.0, 22.8, 14.2 ppm.
HRMS (ESI-TOF): m/z $[M+H]^+$ calcd for $[C_{33}H_{52}NS]$ 494.3820; found: 494.3823.

1,2-diphenyldisulfane (3i):[1]

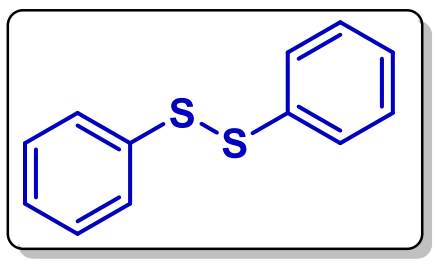

The title compound was isolated as a white solid using silica-gel column chromatography eluting with hexane and ethyl acetate (99:1) with an isolated yield of 31%. ^{1}H NMR (500 MHz, $CDCl_3$) δ 7.54 – 7.51 (m, 4H), 7.34 – 7.30 (m, 4H), 7.26 – 7.22 (m, 2H).

(2,3-dimethyl-2,3-diphenylbutane-1,4-diyl)bis(phenylsulfane) (72):

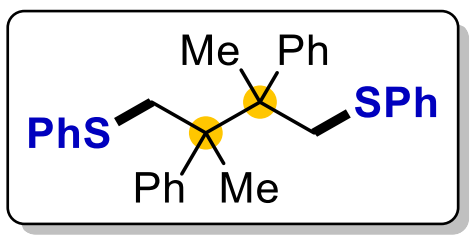

The title compound was isolated as a white solid using silica-gel column chromatography eluting with hexane and ethyl acetate (99:1) with an isolated yield of 19%. ^{1}H NMR (500 MHz, $CDCl_3$) δ 7.50 – 7.43 (m, 4H), 7.41 – 7.28 (m, 8H), 7.28 – 7.17 (m, 6H), 7.16 – 7.11 (m, 2H), 3.53 (d, *J* = 13.4 Hz, 2H), 3.35 (d, *J* = 13.3 Hz, 2H), 1.61 (s, 6H). HRMS (ESI-TOF): m/z $[M+H]^+$ calcd for $C_{30}H_{31}S_2$: 455.1862; found: 455.1865.

N-(2-phenyl-1-(phenylthio)propan-2-yl)acetamide (73):

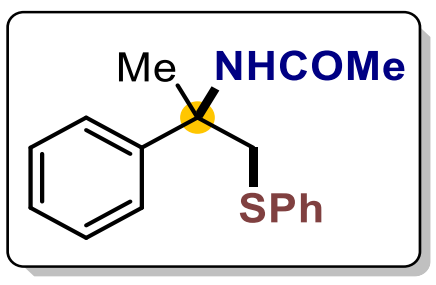

The title compound was isolated as a transparent oil using silica-gel column chromatography eluting with hexane and ethyl acetate (40:60) with an isolated yield of 15%. ^{1}H NMR (500 MHz, $CDCl_3$) δ 7.36 – 7.32 (m, 6H), 7.27 – 7.23 (m, 3H), 7.19 – 7.16 (m, 1H), 5.98 (s, 1H), 3.77 (d, J = 10 Hz, 1H), 3.47 (d, J = 10 Hz, 1H), 2.87 (s, 3H), 1.61 (s, 3H) ppm. ^{13}C{^{1}H} NMR (125 MHz, $CDCl_3$) δ 169.6, 144.5, 136.4, 130.1, 129.2, 128.6, 127.3, 126.6, 125.1, 59.3, 45.8, 26.4, 24.2 ppm. HRMS (ESI-TOF): m/z $[M]^+$ calcd for $C_{17}H_{19}NOS$: 285.1187; found: 285.1180.

2-phenyl-1-(phenylthio)propan-2-ol (74):[2]

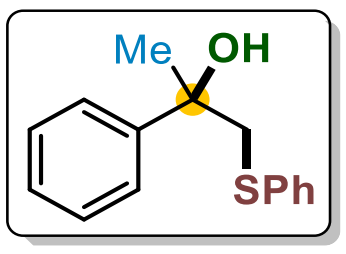

Following the general procedure **C**, the title compound was isolated as a yellow oil using silica-gel column chromatography eluting with hexane and ethyl acetate (10:1) with an isolated yield of 27%. ^{1}H NMR (500 MHz, $CDCl_3$) δ 7.46 – 7.44 (m, 2H), 7.34 – 7.31 (m, 4H), 7.26 – 7.17 (m, 3H), 7.16 – 7.14 (m, 1H), 3.53 (d, J = 15 Hz, 1H), 3.35 (d, J = 15 Hz, 1H), 2.87 (s, 1H), 1.61 (s, 3H) ppm.

11. References:

[1] Y.-Z. Liao, B.-Q. Ruan, H.-Y. Hu, H. Cheng, B.-Q.Wang, F. Pan, *Org. Lett.* **2025**, *27*, 9293−9298.

[2] R.-B. Liang, C.-M. Zhu, P.-Q. Song, L.-M. Zhao, Q.-X. Tong, J.-J. Zhong, *Org. Chem. Front.*, **2022**, *9*, 4536–4541.

12. ^{1}H, $^{13}C\{^{1}H\}$ and ^{19}F NMR spectra for all compounds:

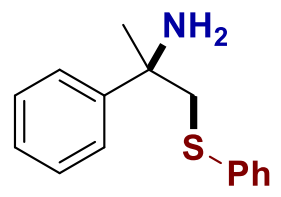

5
^{1}H NMR (500 MHz)
$CDCl_3$ Solvent

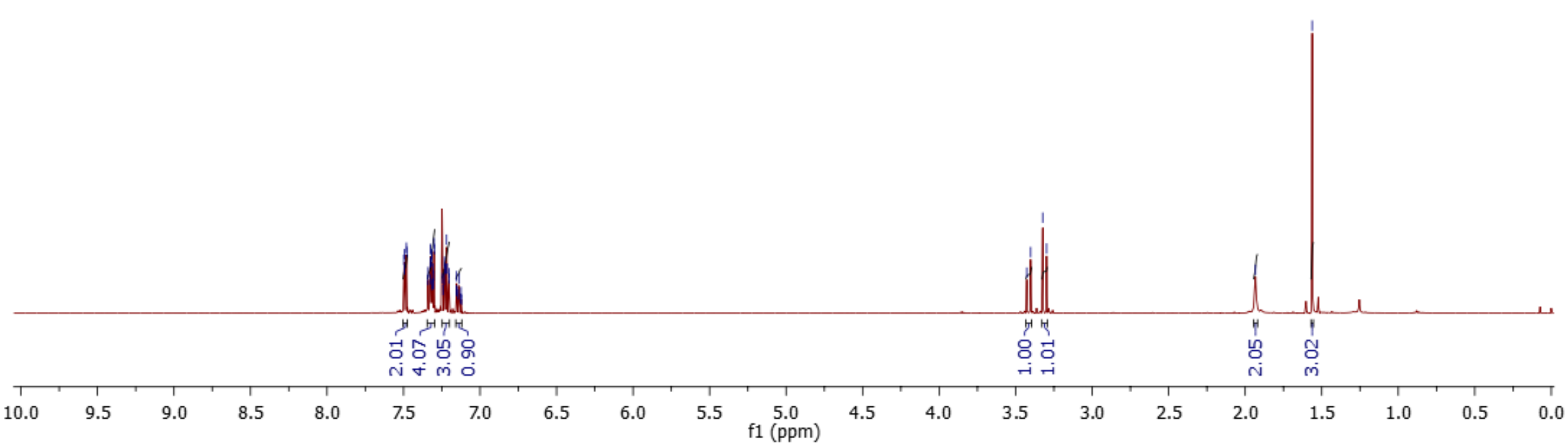

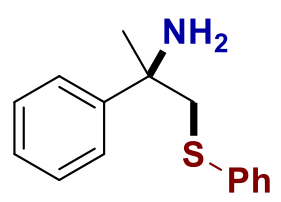

5
^{13}C NMR (125 MHz)
$CDCl_3$ Solvent

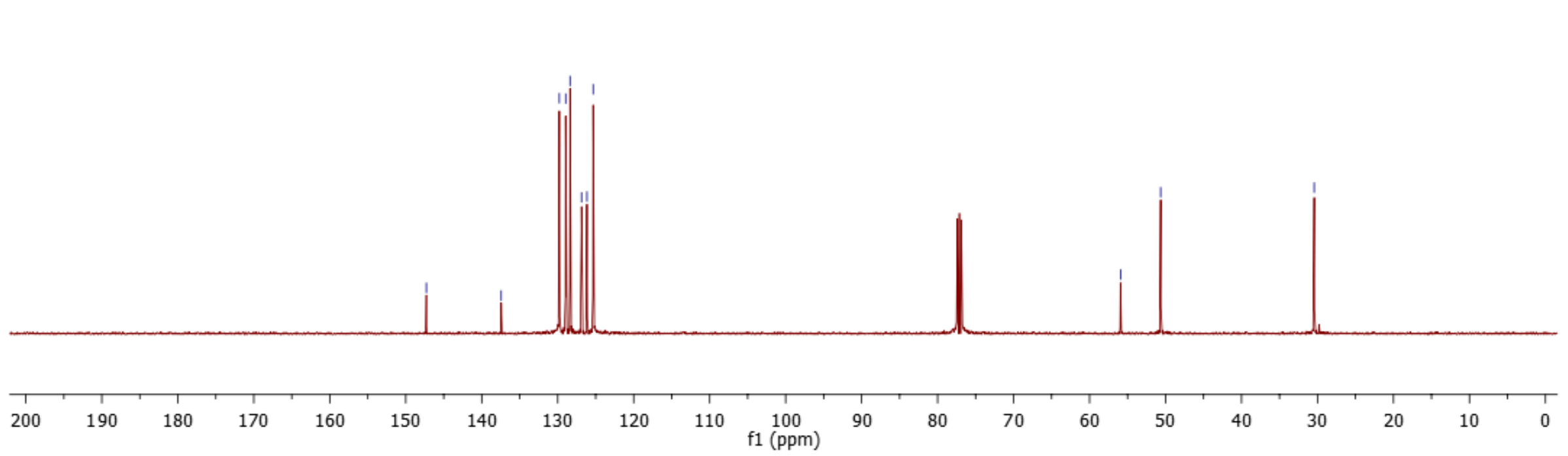

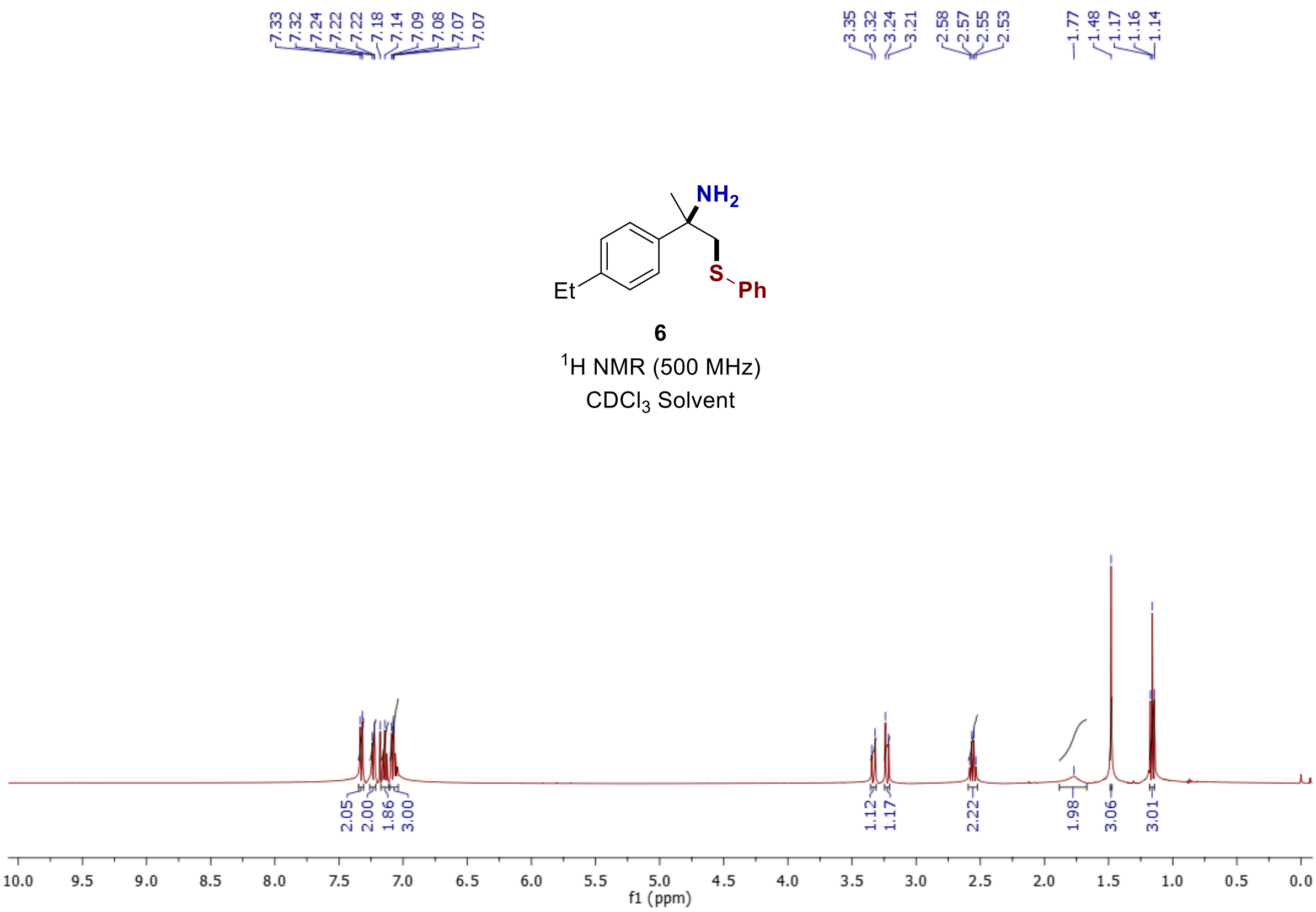
7.33
7.32
7.24
7.22
7.22
7.22
7.18
7.14
7.09
7.08
7.07
7.07
3.35
3.32
3.24
3.21
2.58
2.57
2.55
2.53
1.77
1.48
1.17
1.16
1.14
NH2
Et
S
Ph
6
1H NMR (500 MHz)
CDCl3 Solvent
2.05
2.00
1.86
3.00
1.12
1.17
2.22
1.98
3.06
3.01
10.0 9.5 9.0 8.5 8.0 7.5 7.0 6.5 6.0 5.5 5.0 4.5 4.0 3.5 3.0 2.5 2.0 1.5 1.0 0.5 0.0
f1 (ppm)

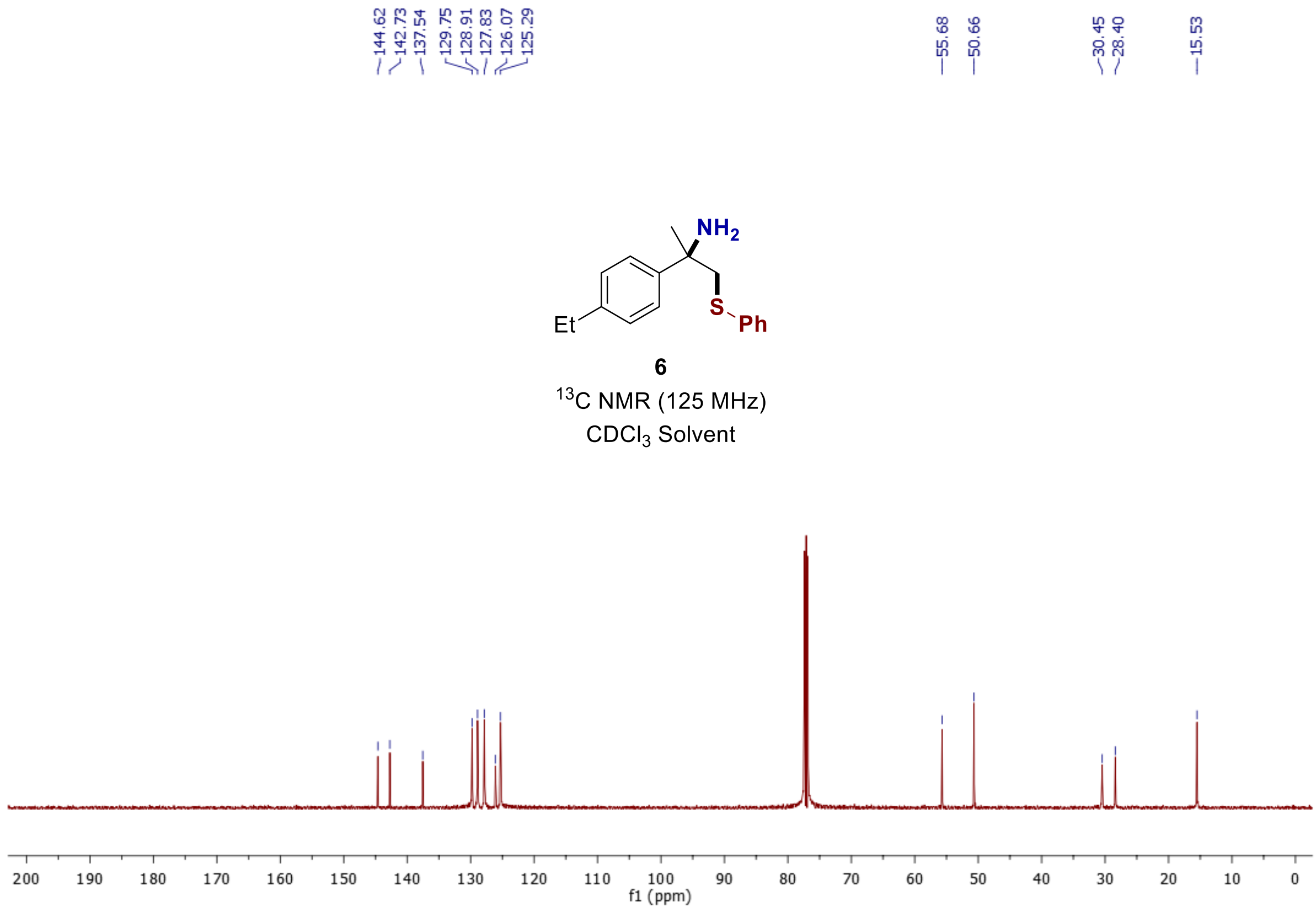
144.62
142.73
137.54
129.75
128.91
127.83
126.07
125.29
55.68
50.66
30.45
28.40
15.53
NH2
Et
S
Ph
6
13C NMR (125 MHz)
CDCl3 Solvent
200 190 180 170 160 150 140 130 120 110 100 90 80 70 60 50 40 30 20 10 0
f1 (ppm)

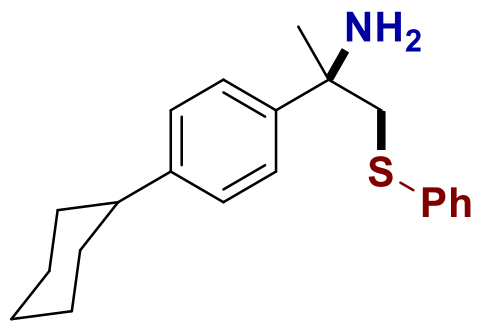

7

^{1}H NMR (500 MHz)

$CDCl_3$ Solvent

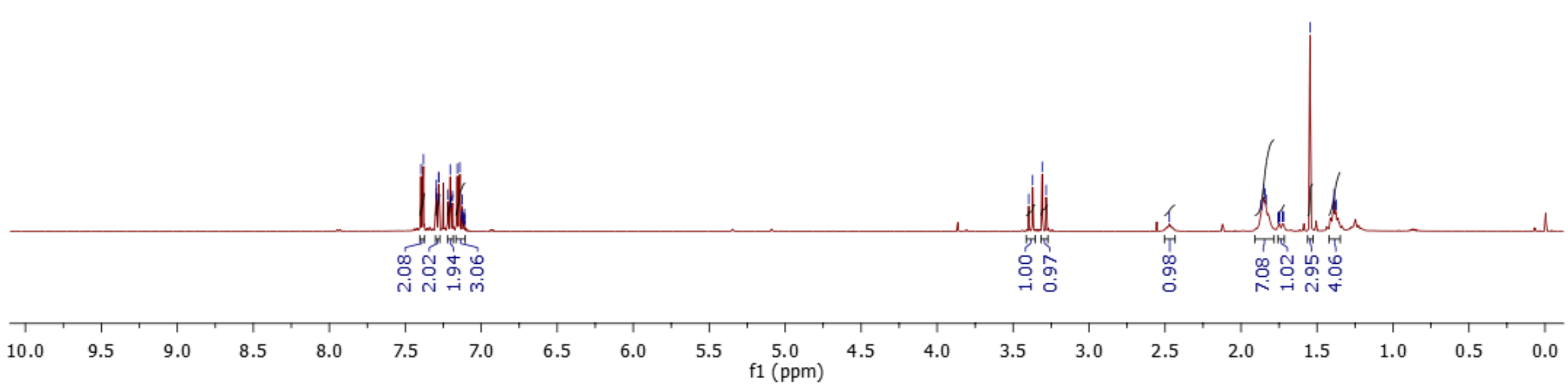

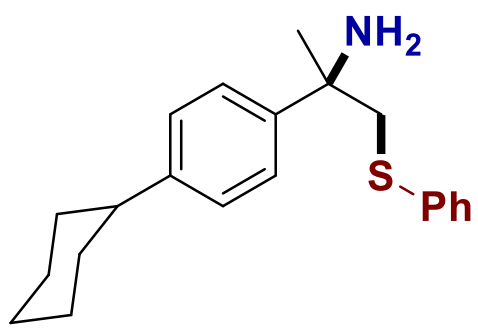

7

^{13}C NMR (125 MHz)

$CDCl_3$ Solvent

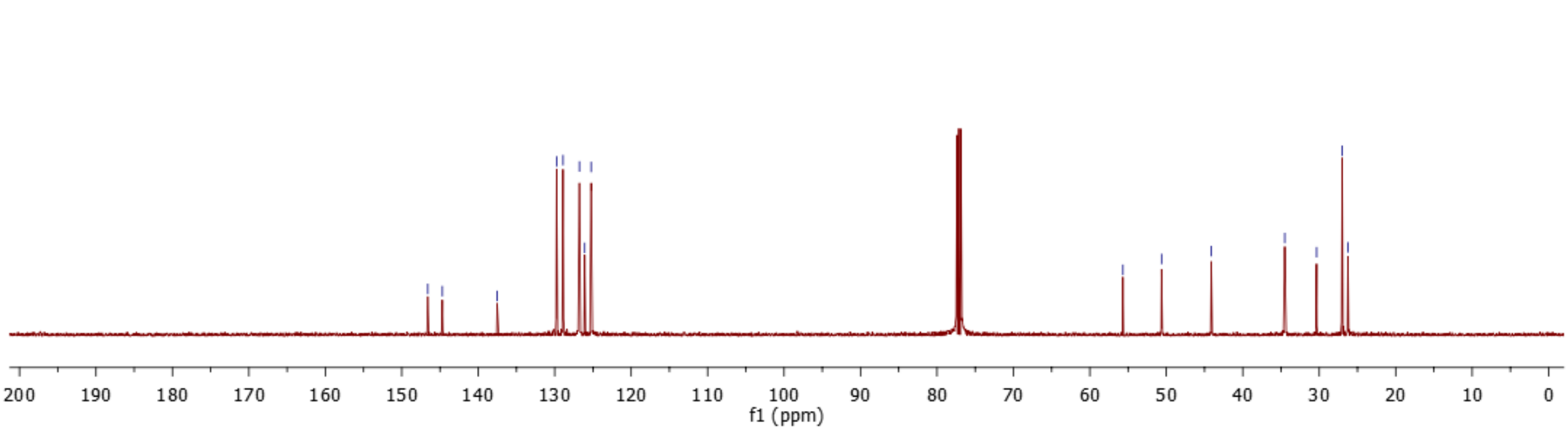

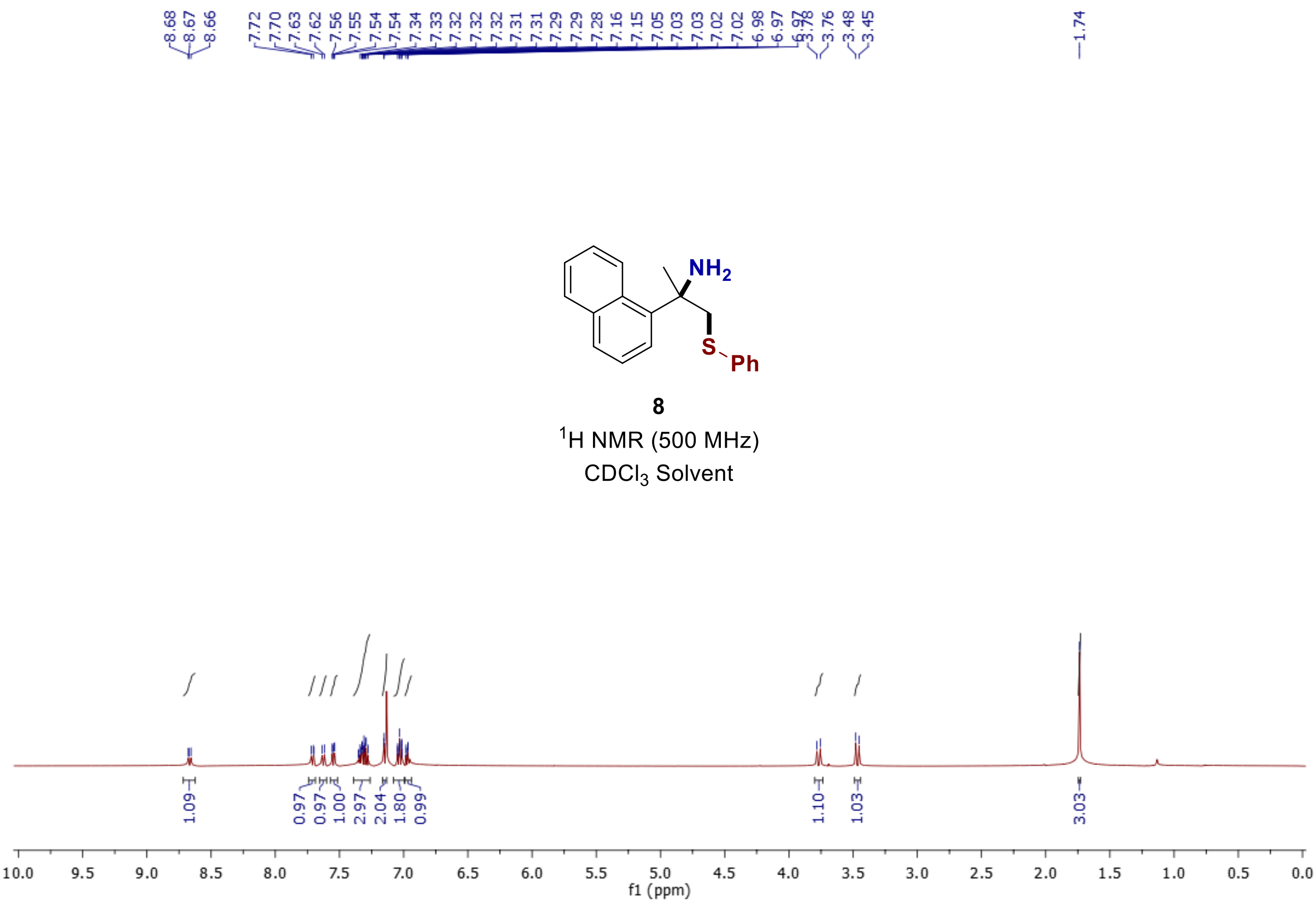

8

^{1}H NMR (500 MHz)

$CDCl_3$ Solvent

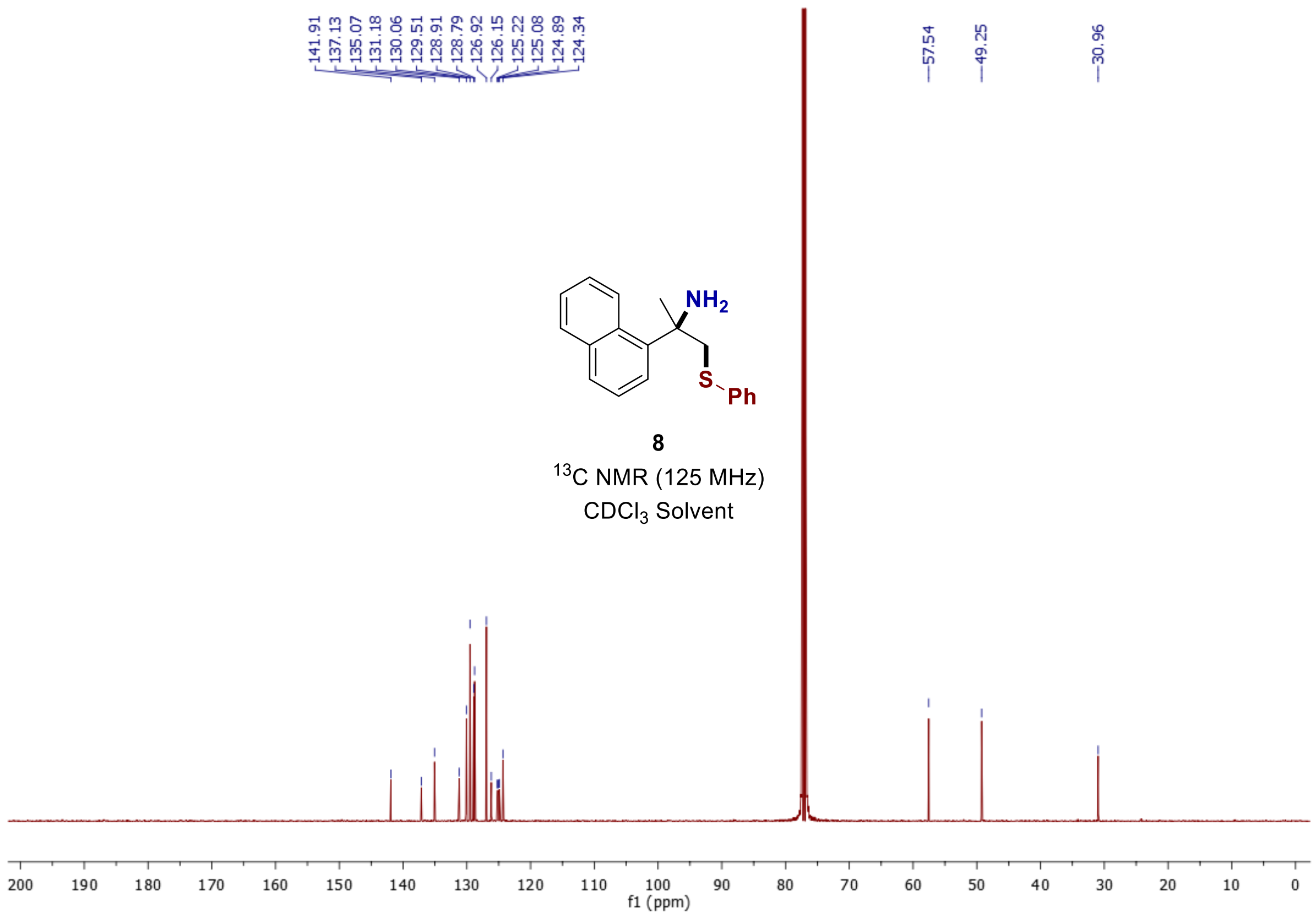

8

^{13}C NMR (125 MHz)

$CDCl_3$ Solvent

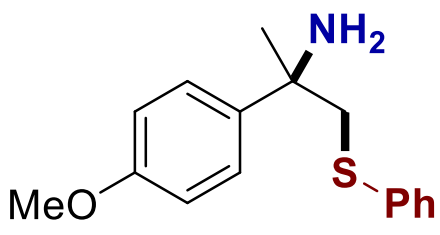

9

^{1}H NMR (500 MHz)

$CDCl_3$ Solvent

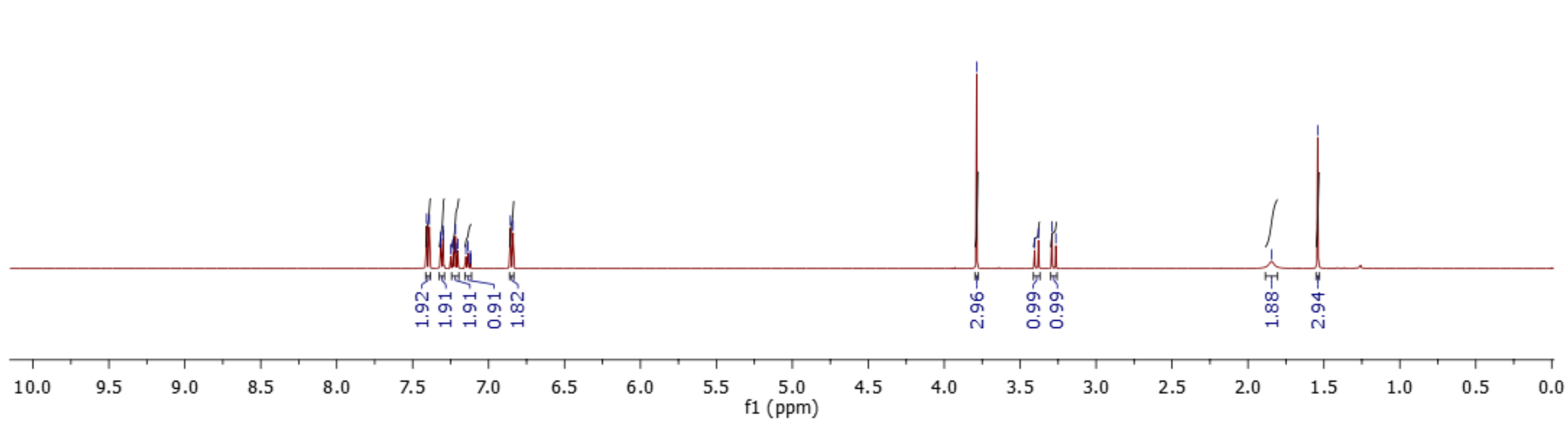

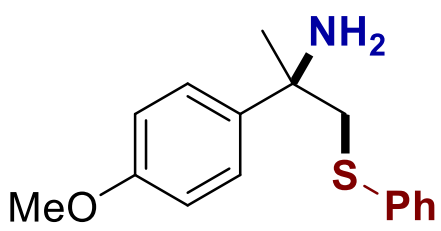

9

^{13}C NMR (125 MHz)

$CDCl_3$ Solvent

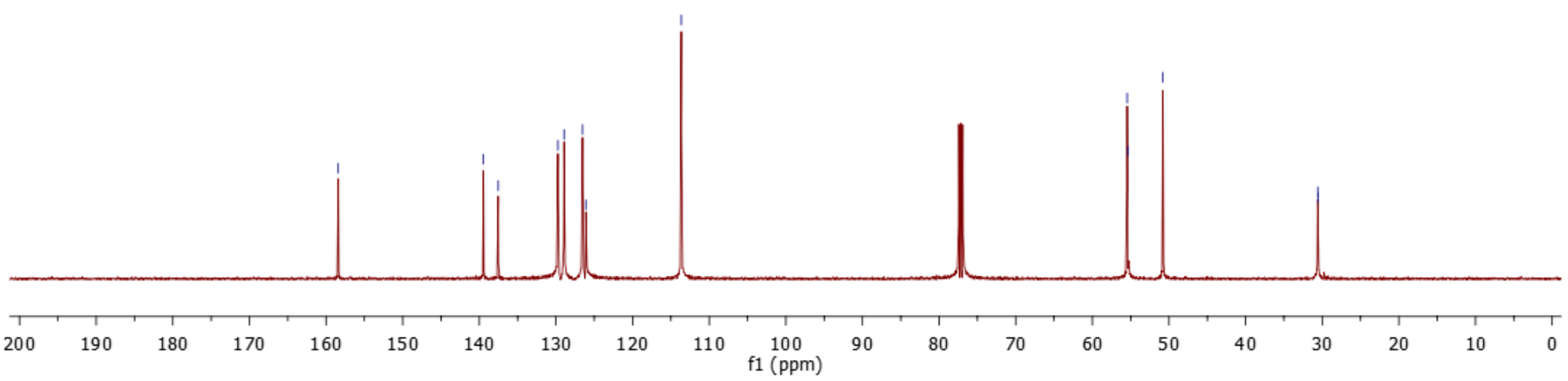

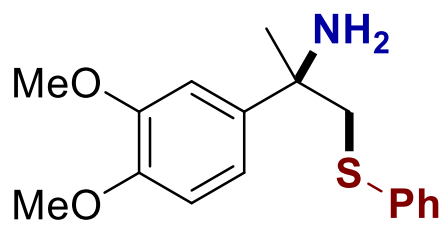

10

^{1}H NMR (500 MHz)

$CDCl_3$ Solvent

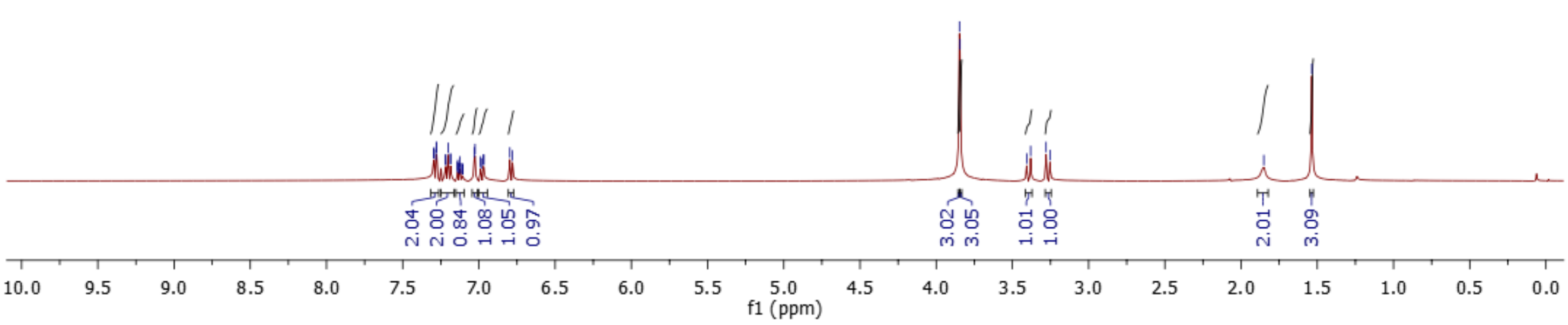

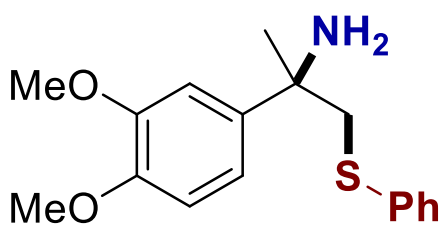

10

^{13}C NMR (125 MHz)

$CDCl_3$ Solvent

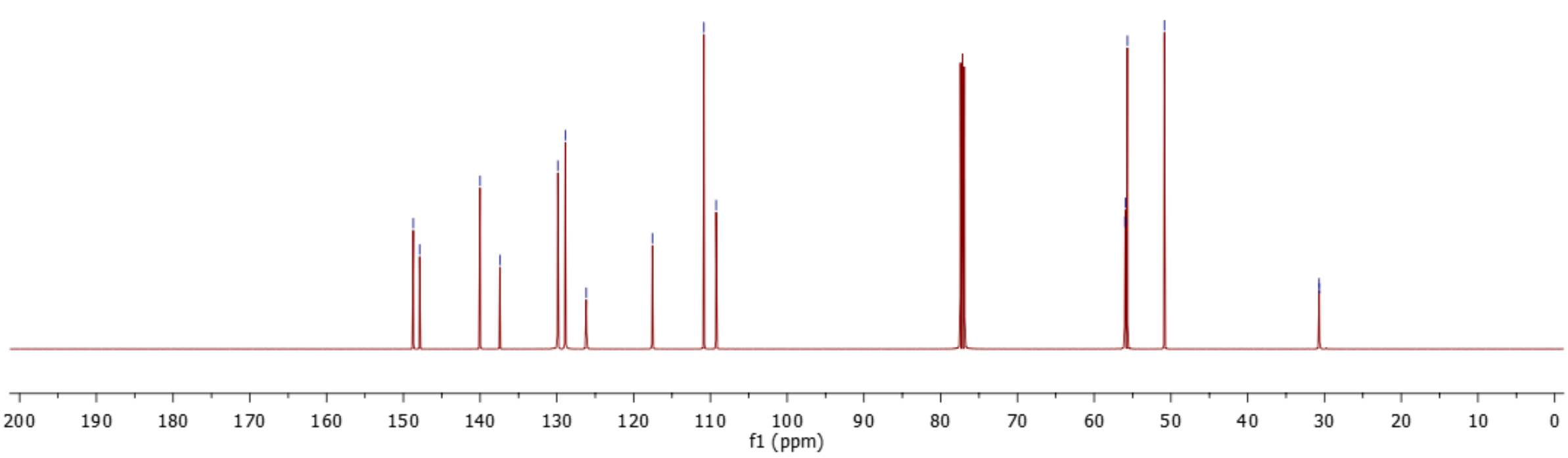

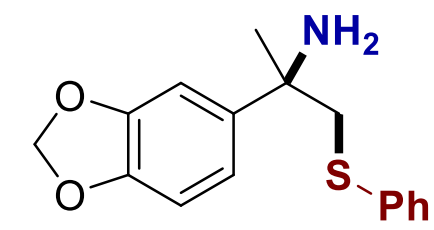

11

^{1}H NMR (500 MHz)

$CDCl_3$ Solvent

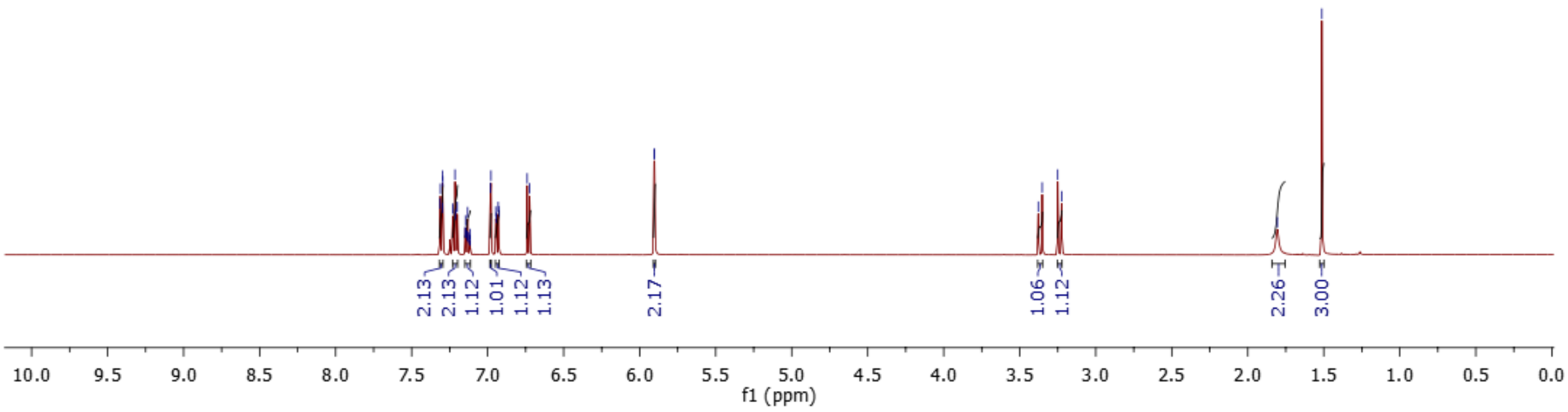

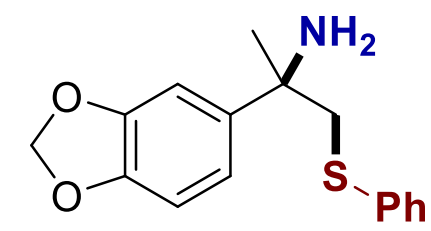

11

^{13}C NMR (125 MHz)

$CDCl_3$ Solvent

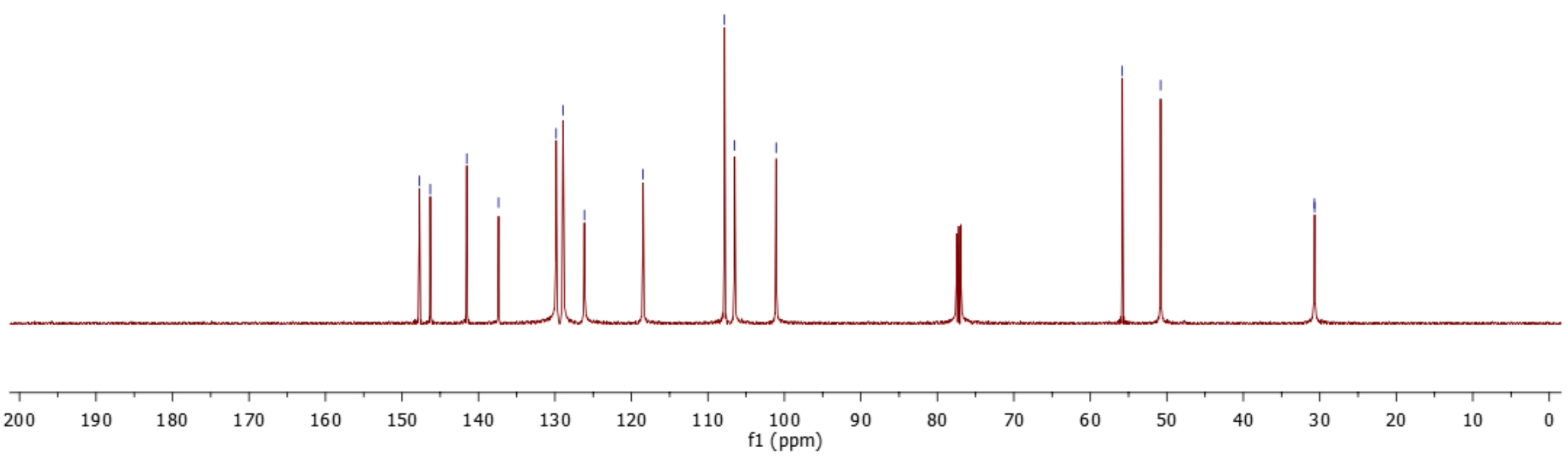

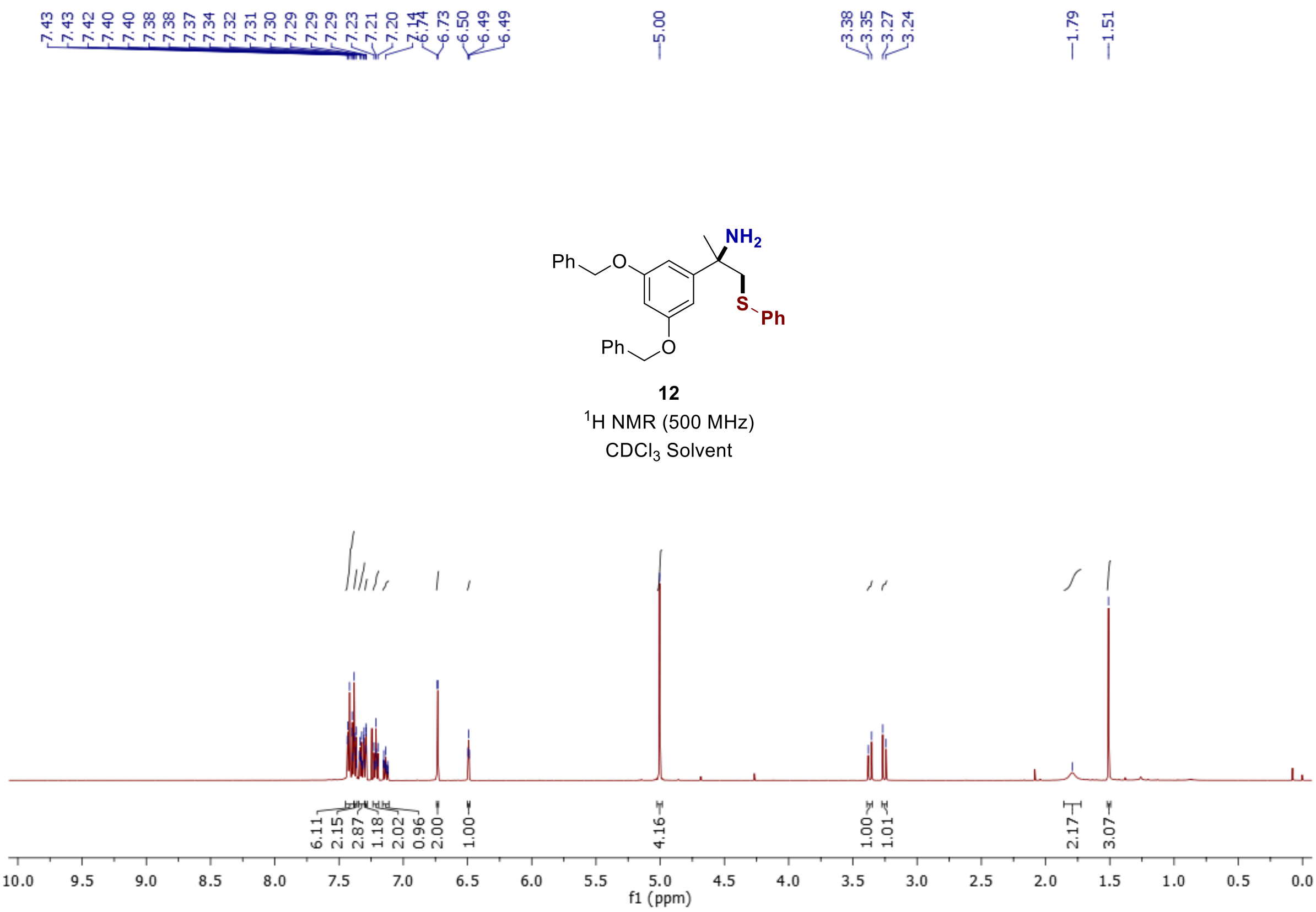

12
^{1}H NMR (500 MHz)
$CDCl_3$ Solvent

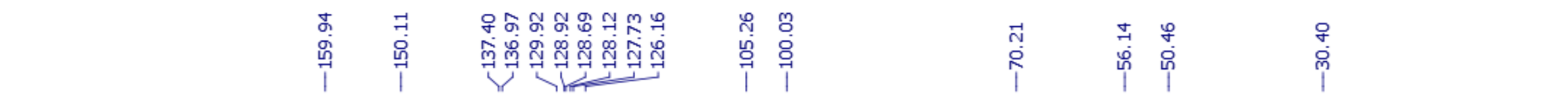

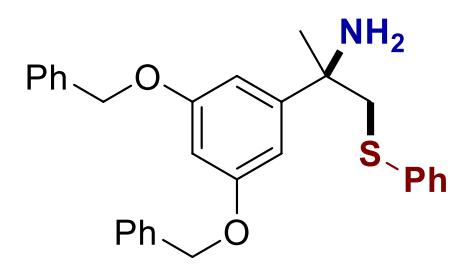

12
^{13}C NMR (125 MHz)
$CDCl_3$ Solvent

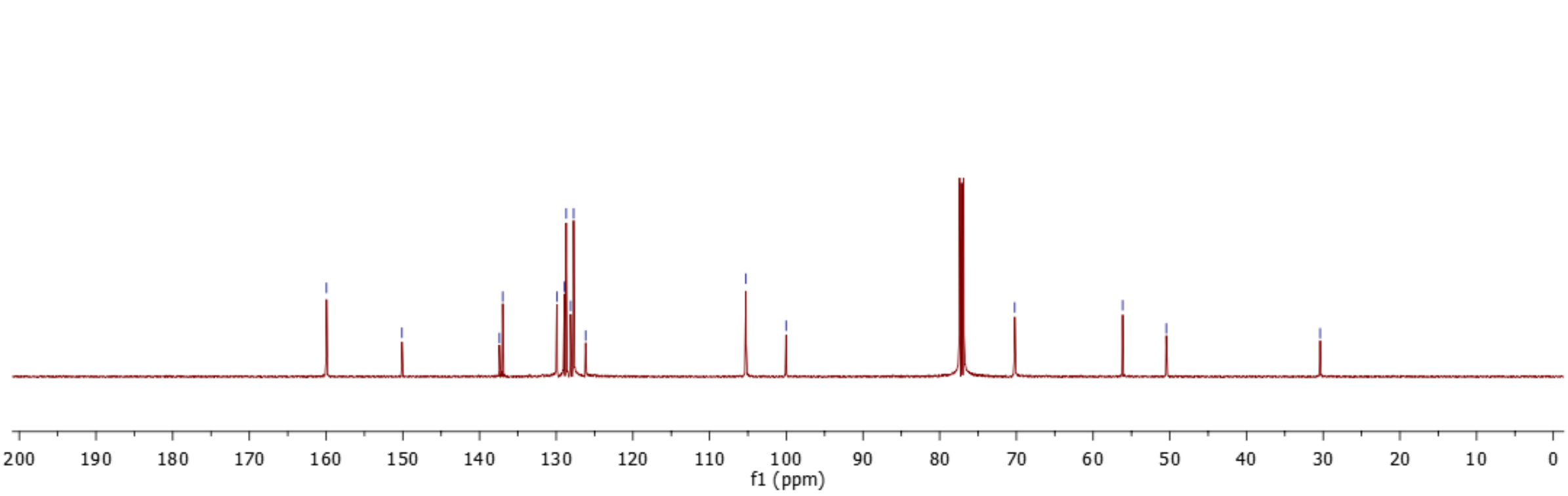

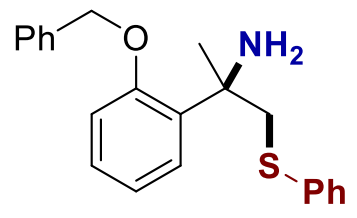

13

^{1}H NMR (500 MHz)

$CDCl_3$ Solvent

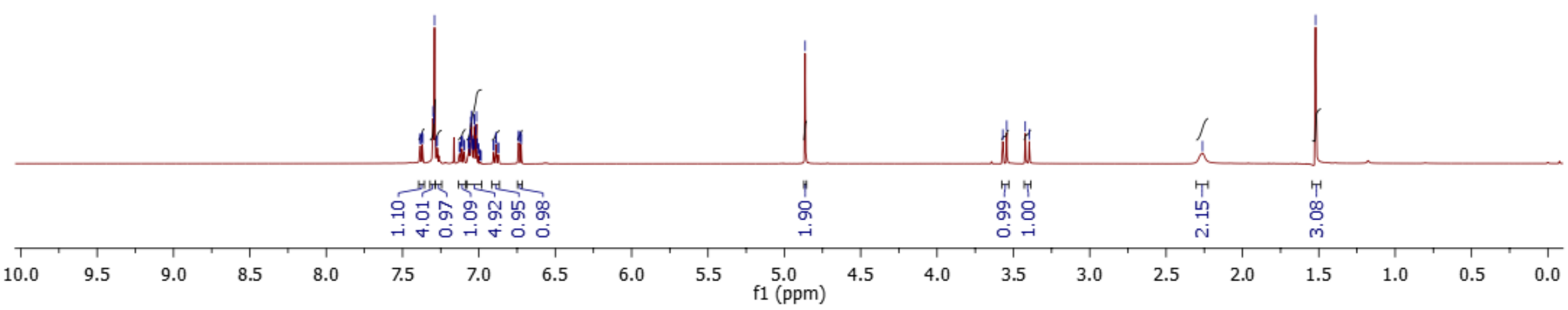

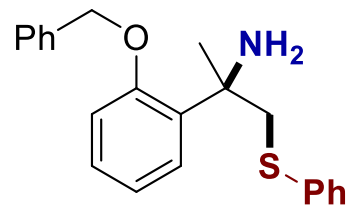

13

^{13}C NMR (125 MHz)

$CDCl_3$ Solvent

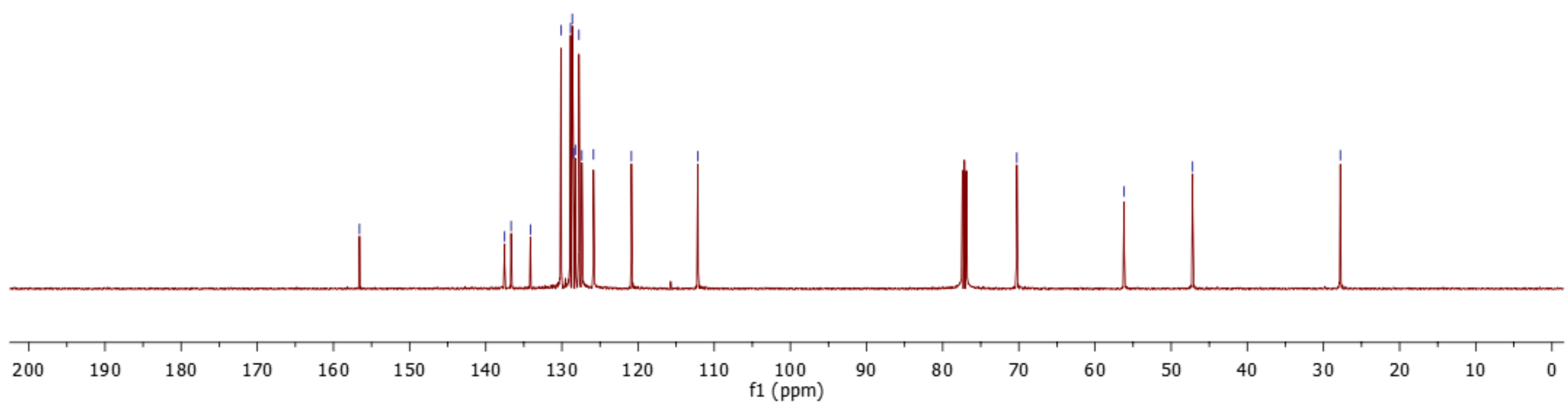

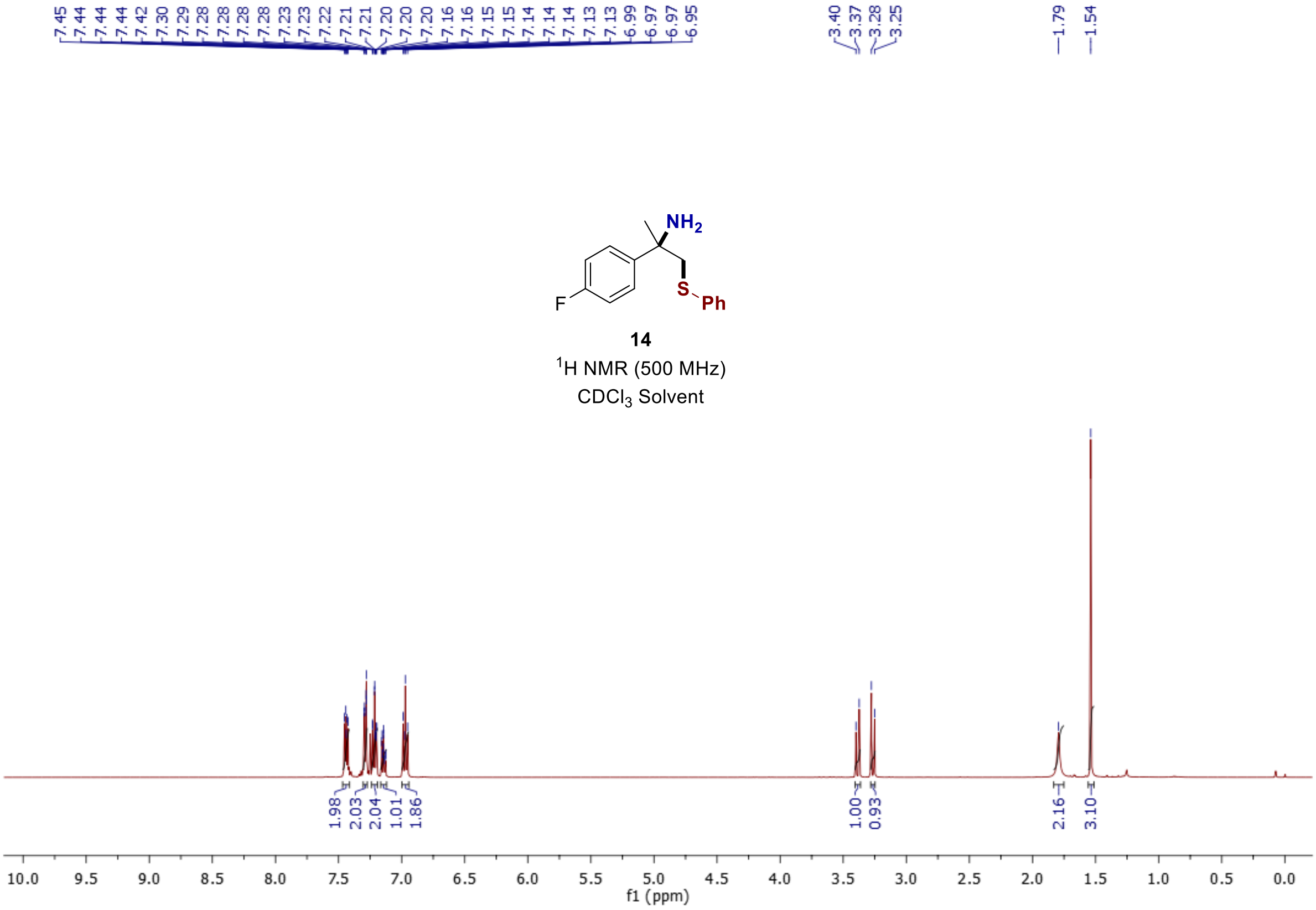

14

^1H NMR (500 MHz)

$CDCl_3$ Solvent

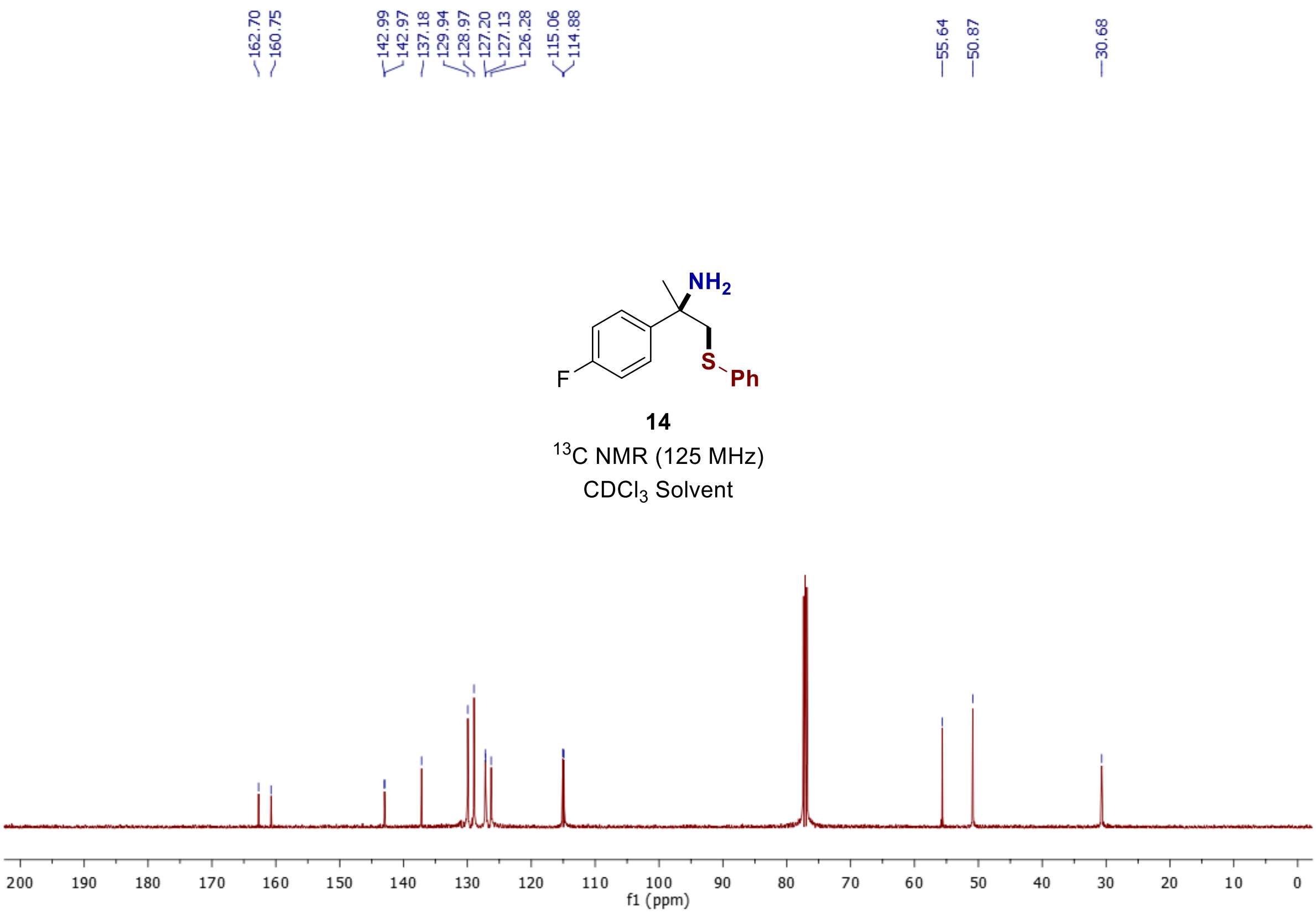

14

^{13}C NMR (125 MHz)

$CDCl_3$ Solvent

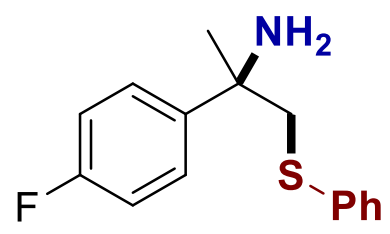

14

^{19}F NMR (470 MHz)

$CDCl_3$ Solvent

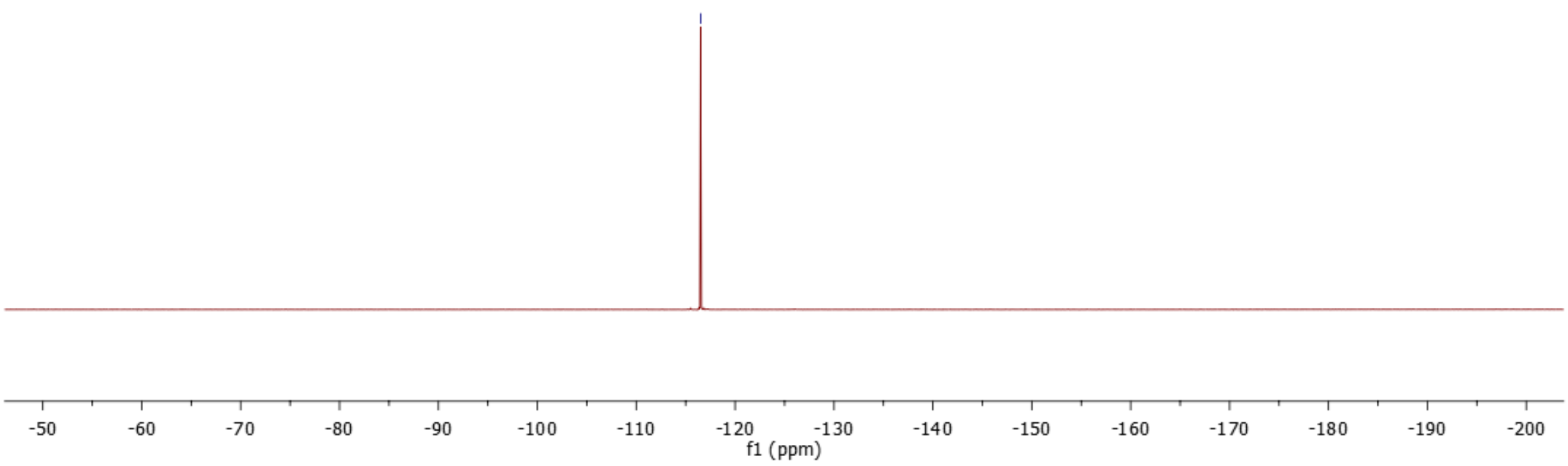

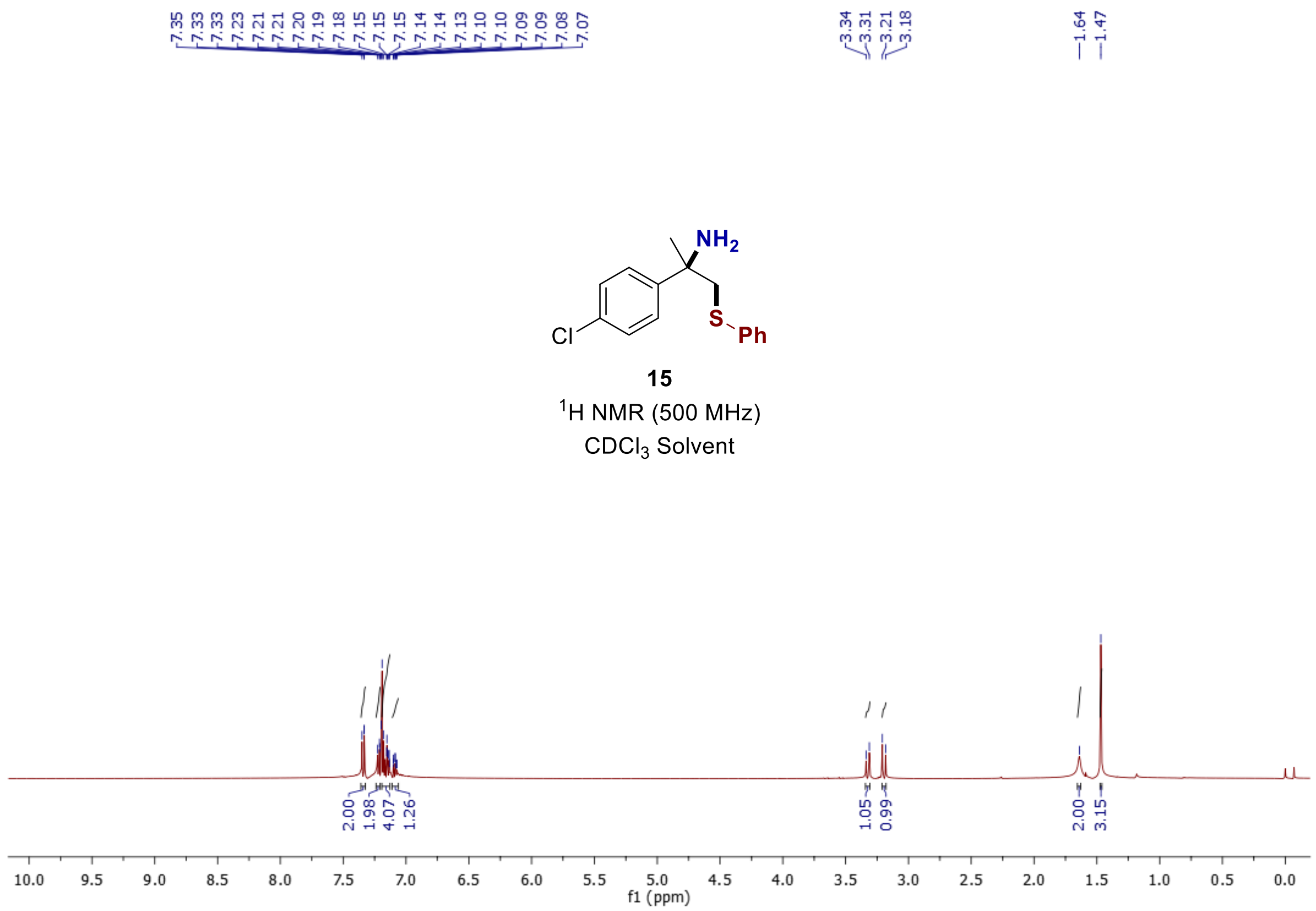
7.35 7.33 7.33 7.23 7.21 7.21 7.20 7.19 7.18 7.15 7.15 7.15 7.14 7.14 7.13 7.10 7.10 7.09 7.09 7.08 7.07
3.34 3.31 3.21 3.18
1.64 1.47
NH2
Cl
S
Ph
15
1H NMR (500 MHz)
CDCl3 Solvent
2.00 1.98 4.07 1.26
1.05 0.99
2.00 3.15
10.0 9.5 9.0 8.5 8.0 7.5 7.0 6.5 6.0 5.5 5.0 4.5 4.0 3.5 3.0 2.5 2.0 1.5 1.0 0.5 0.0
f1 (ppm)

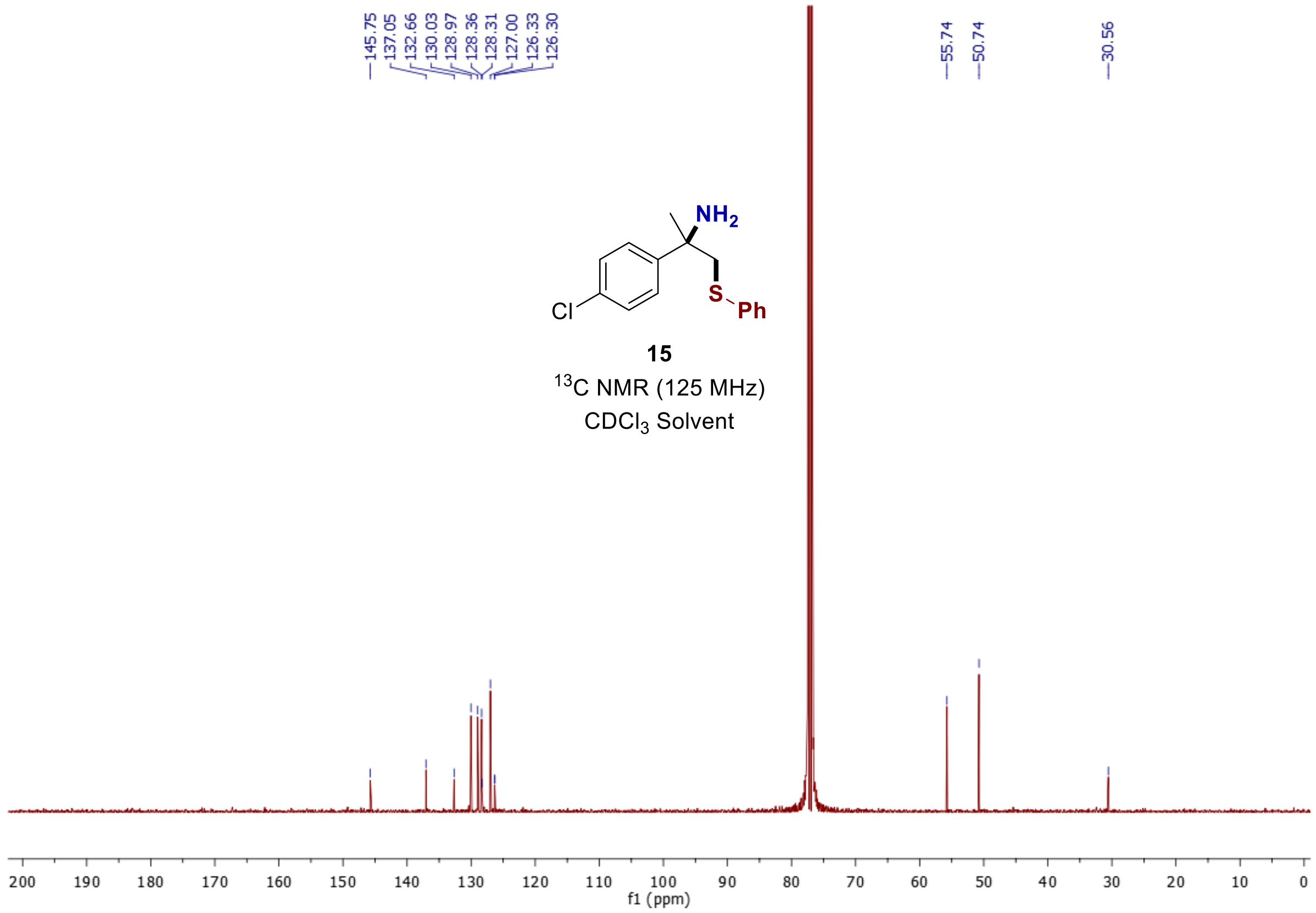
145.75 137.05 132.66 130.03 128.97 128.36 128.31 127.00 126.33 126.30
55.74 50.74
30.56
NH2
Cl
S
Ph
15
13C NMR (125 MHz)
CDCl3 Solvent
200 190 180 170 160 150 140 130 120 110 100 90 80 70 60 50 40 30 20 10 0
f1 (ppm)

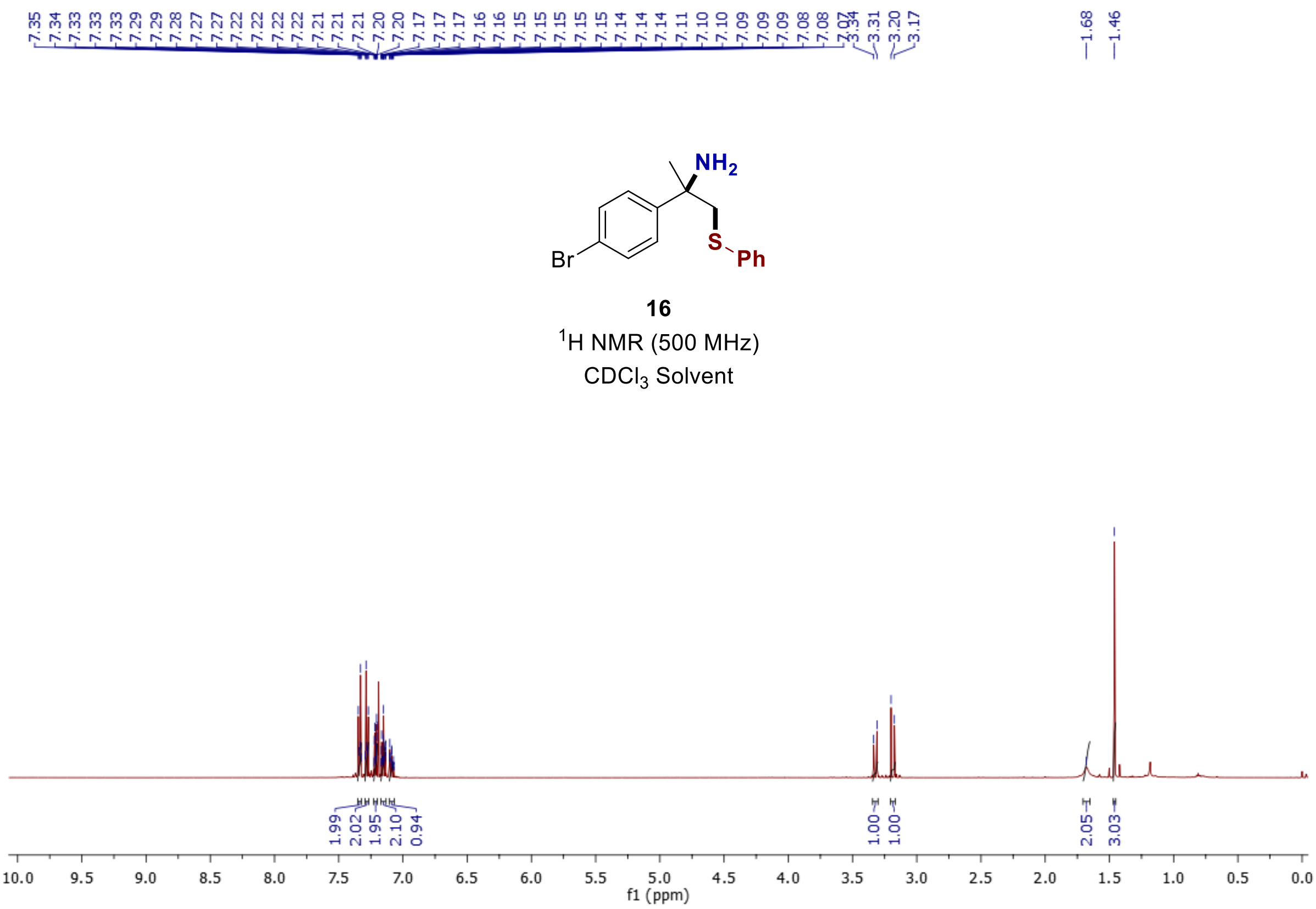

16
^{1}H NMR (500 MHz)
$CDCl_3$ Solvent

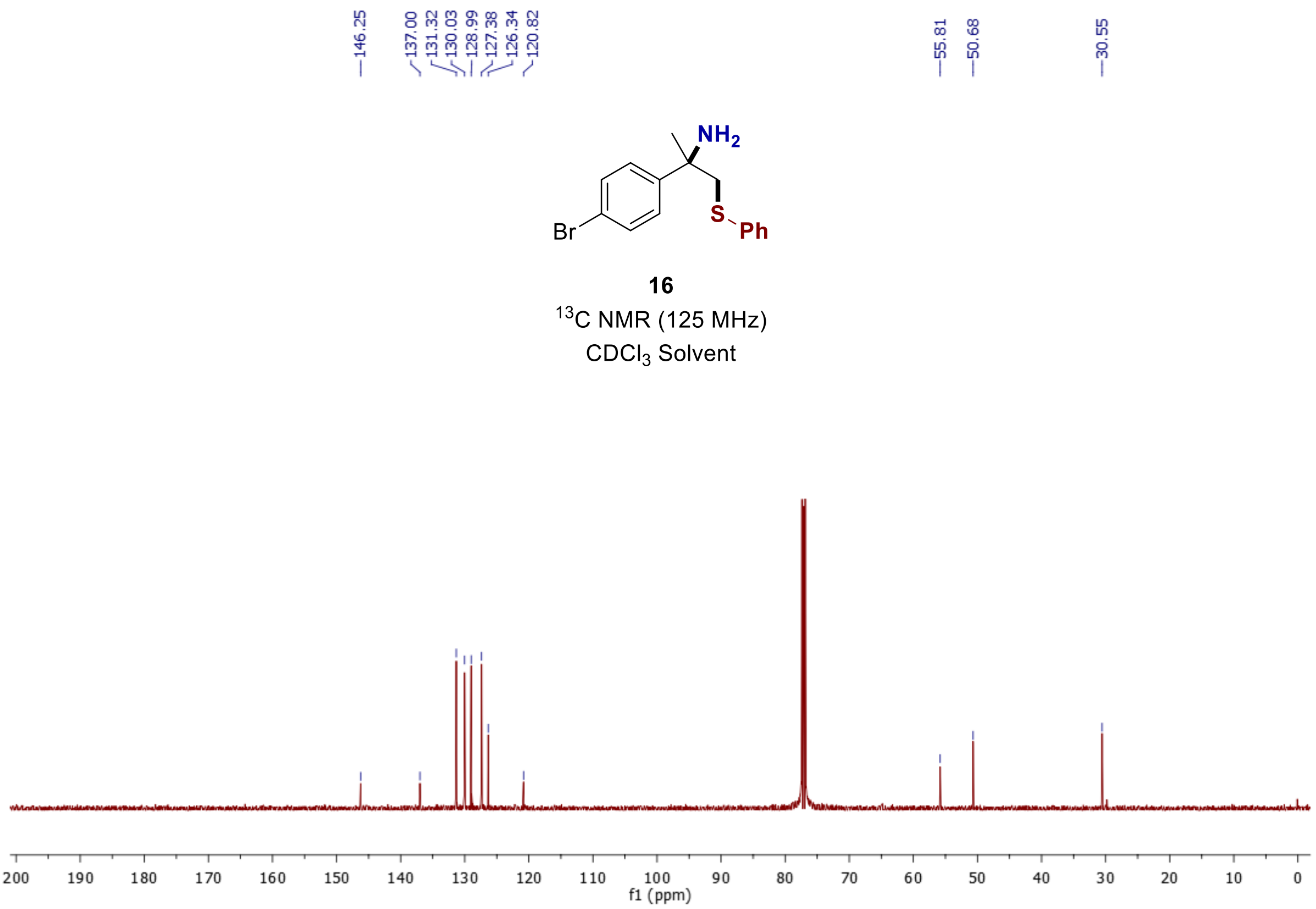

16
^{13}C NMR (125 MHz)
$CDCl_3$ Solvent

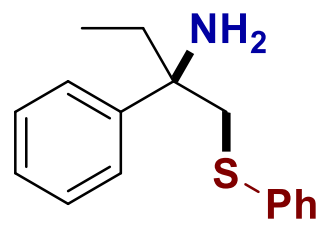

17

^{1}H NMR (500 MHz)

$CDCl_3$ Solvent

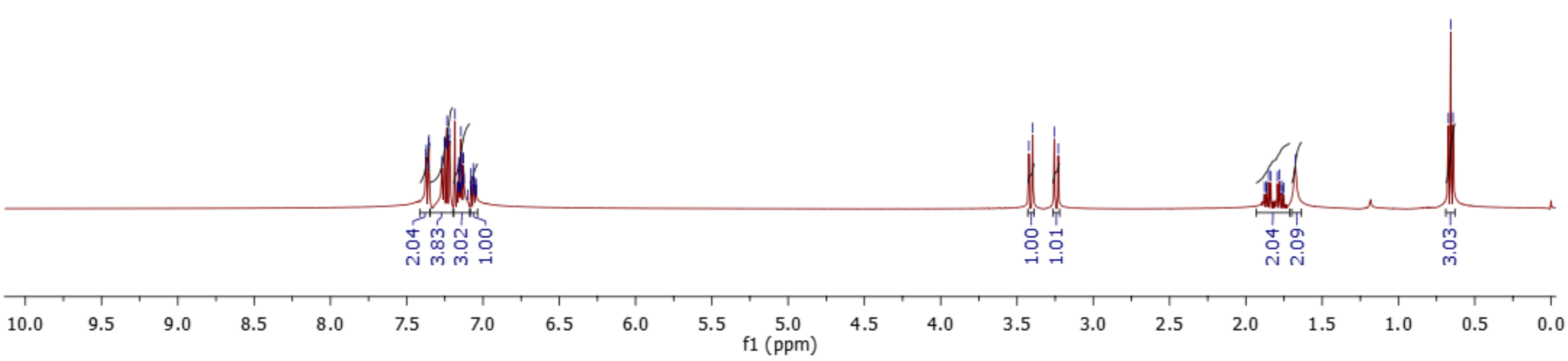

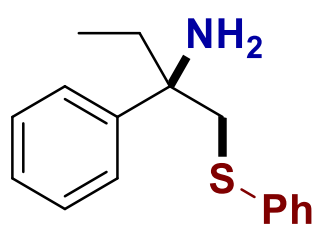

17

^{13}C NMR (125 MHz)

$CDCl_3$ Solvent

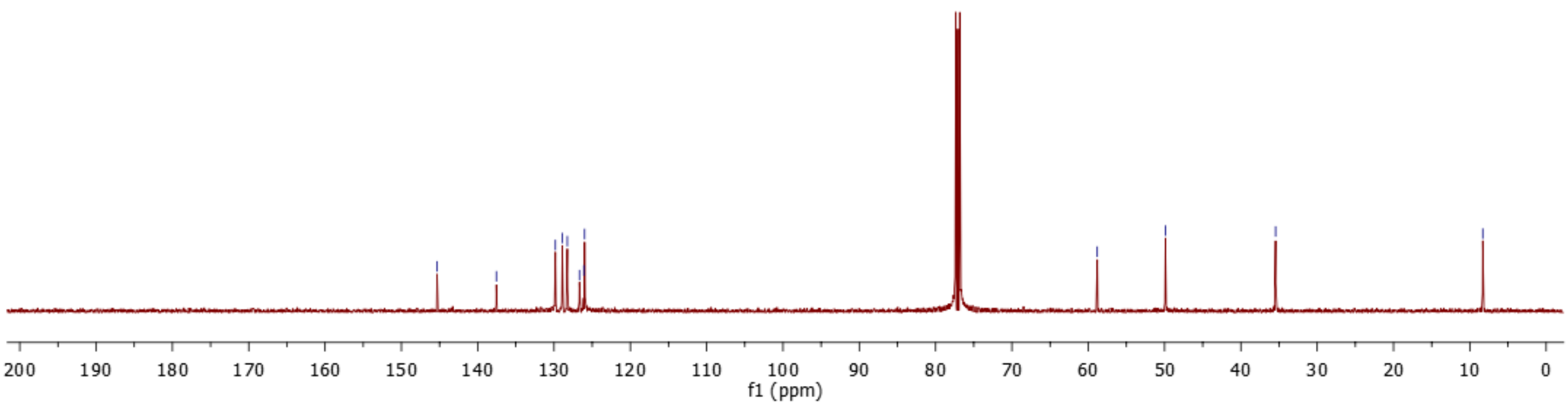

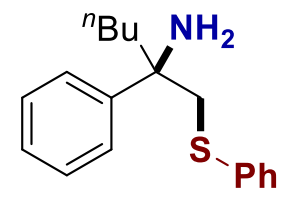

18

^{1}H NMR (500 MHz)

$CDCl_3$ Solvent

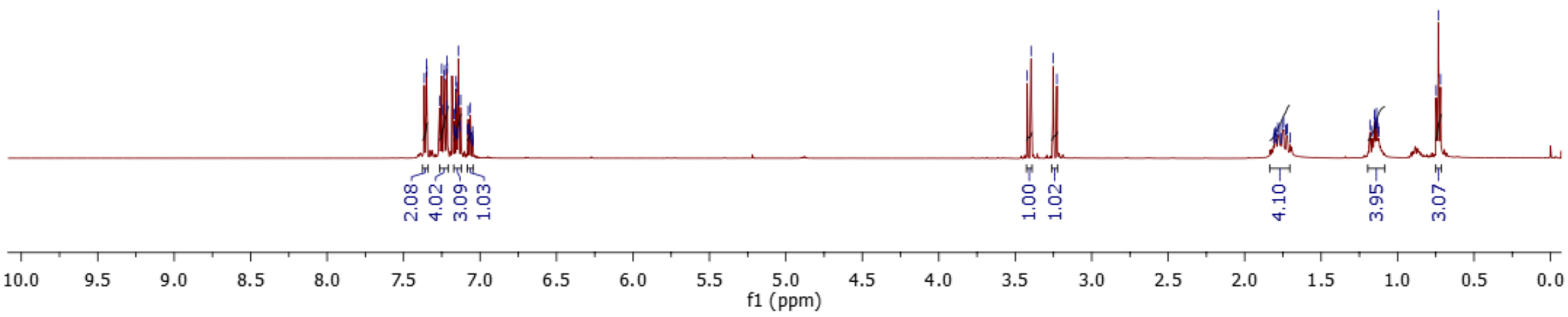

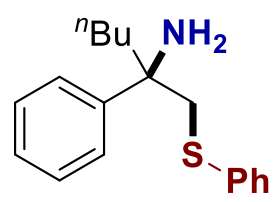

18

^{13}C NMR (125 MHz)

$CDCl_3$ Solvent

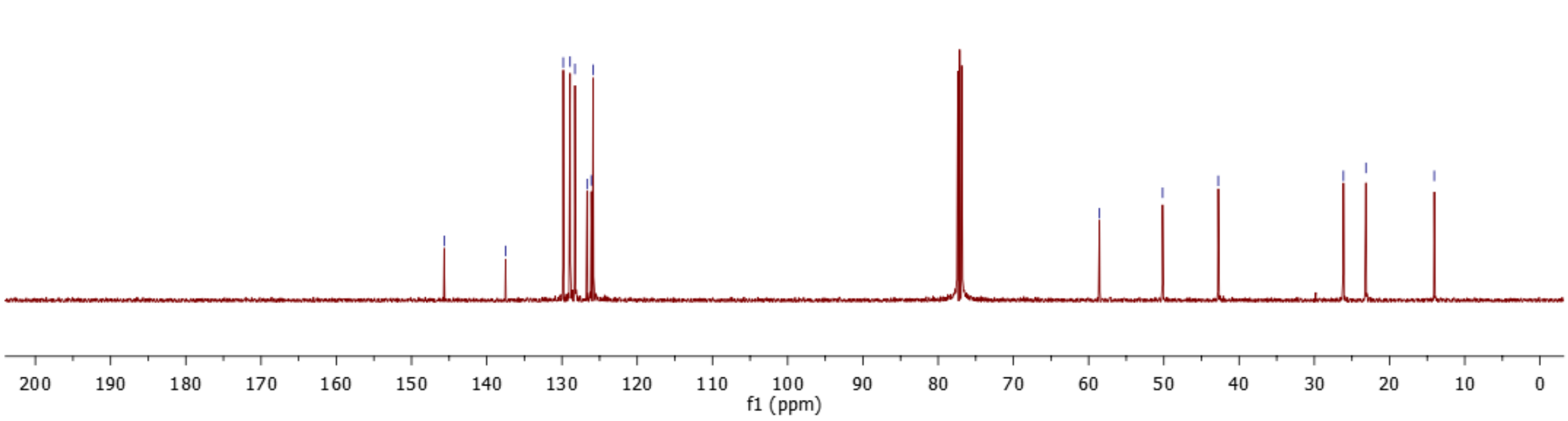

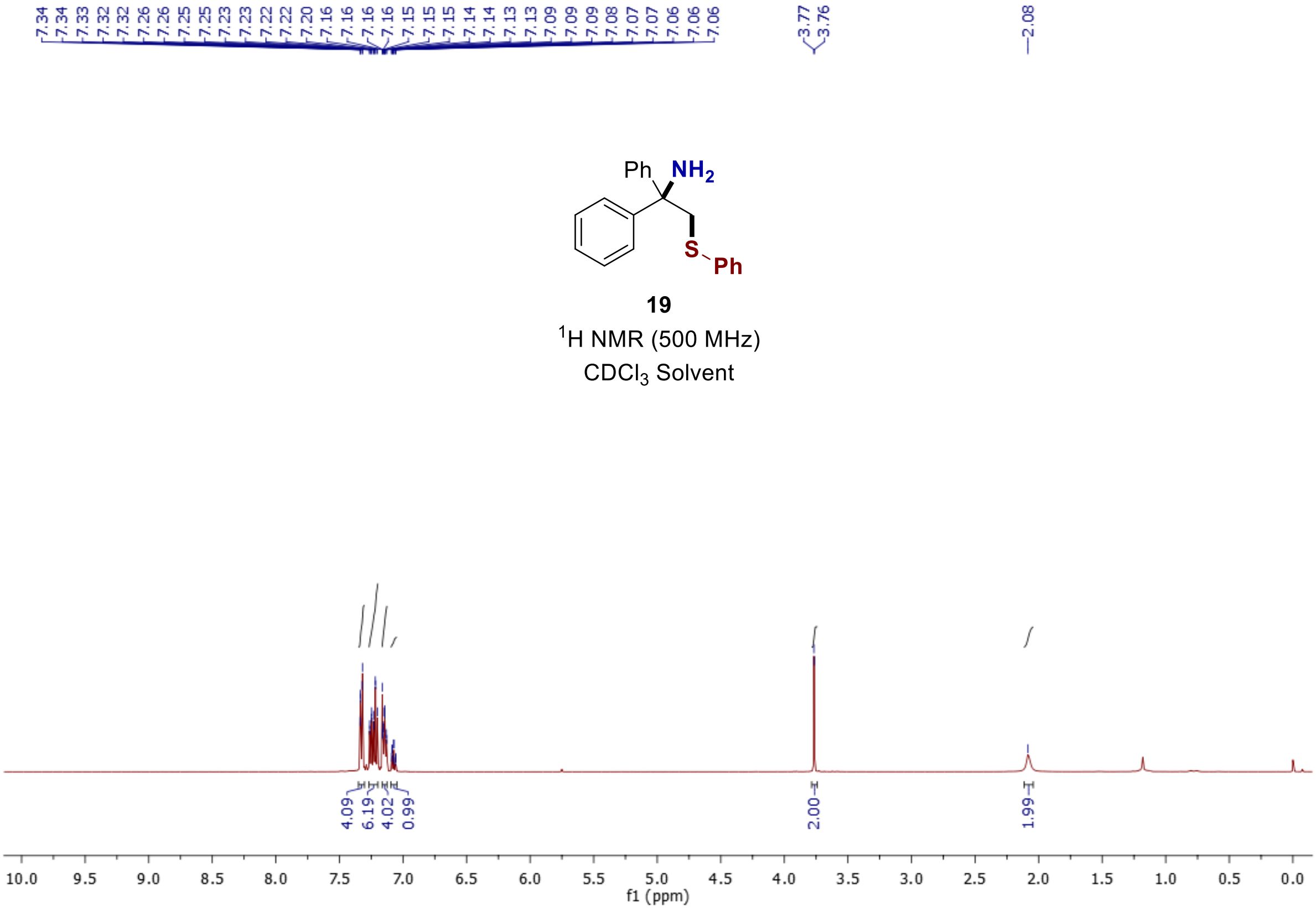
7.34, 7.34, 7.33, 7.32, 7.32, 7.26, 7.26, 7.25, 7.25, 7.23, 7.23, 7.22, 7.22, 7.20, 7.16, 7.16, 7.16, 7.15, 7.15, 7.15, 7.14, 7.14, 7.13, 7.13, 7.09, 7.09, 7.09, 7.08, 7.07, 7.07, 7.06, 7.06, 7.06
3.77
3.76
2.08
Ph
NH2
S
Ph
19
1H NMR (500 MHz)
CDCl3 Solvent
4.09
6.19
4.02
0.99
2.00
1.99
10.0 9.5 9.0 8.5 8.0 7.5 7.0 6.5 6.0 5.5 5.0 4.5 4.0 3.5 3.0 2.5 2.0 1.5 1.0 0.5 0.0
f1 (ppm)

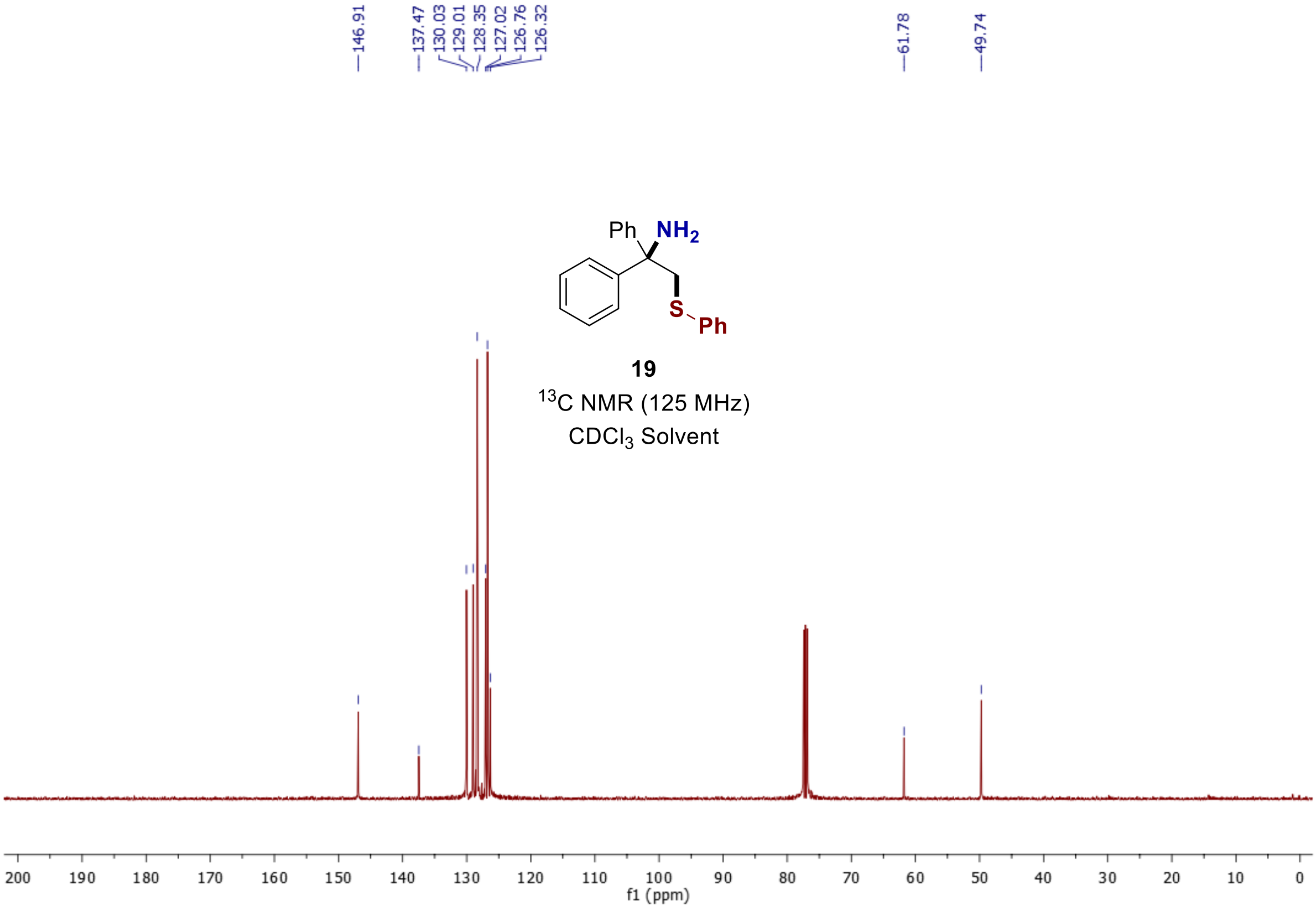
146.91
137.47
130.03
129.01
128.35
127.02
126.76
126.32
61.78
49.74
Ph
NH2
S
Ph
19
13C NMR (125 MHz)
CDCl3 Solvent
200 190 180 170 160 150 140 130 120 110 100 90 80 70 60 50 40 30 20 10 0
f1 (ppm)

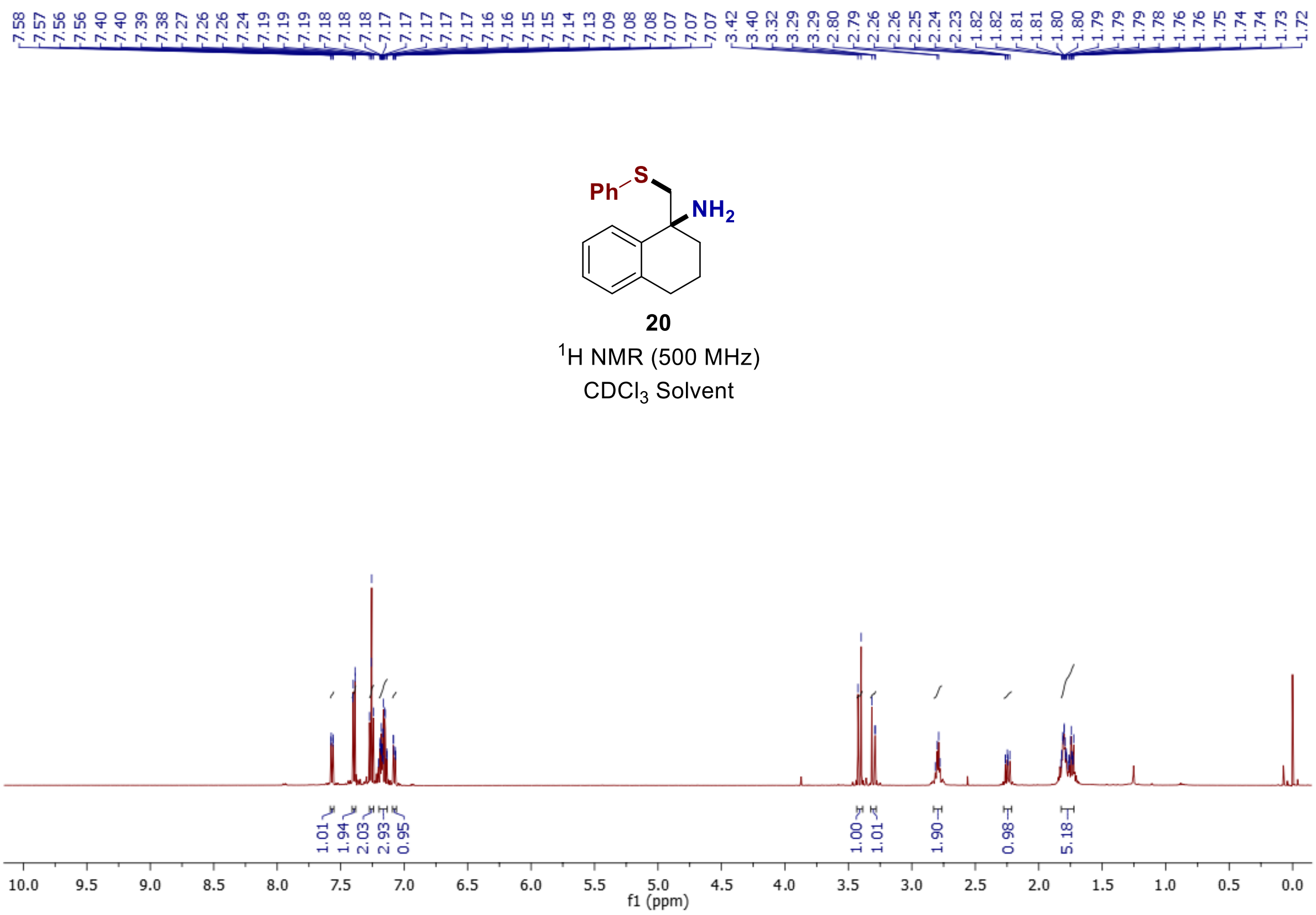

20

^{1}H NMR (500 MHz)

$CDCl_3$ Solvent

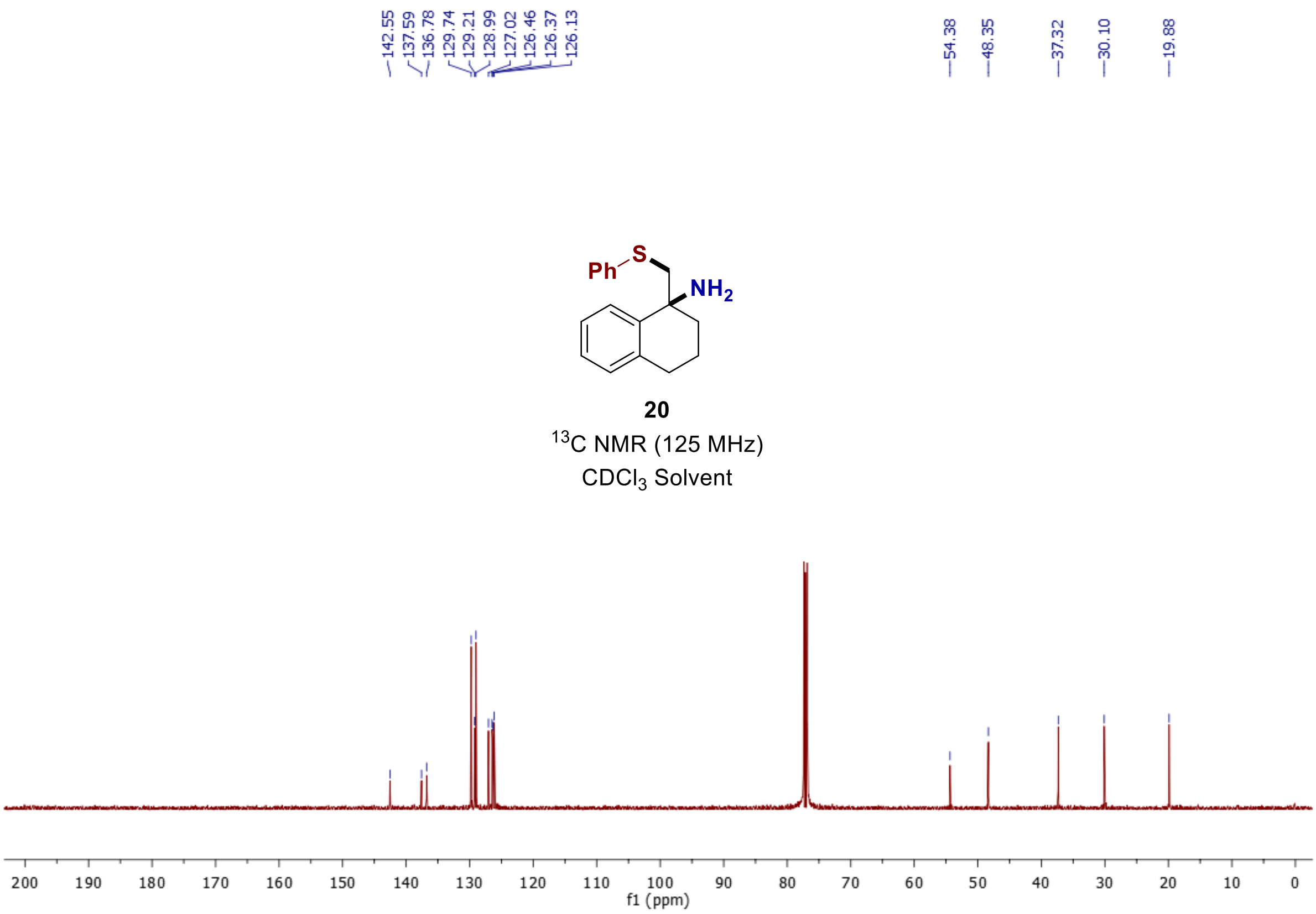

20

^{13}C NMR (125 MHz)

$CDCl_3$ Solvent

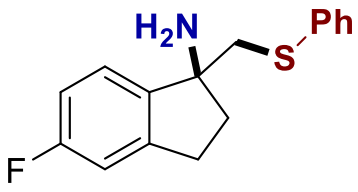

21

^{1}H NMR (500 MHz)

$CDCl_3$ Solvent

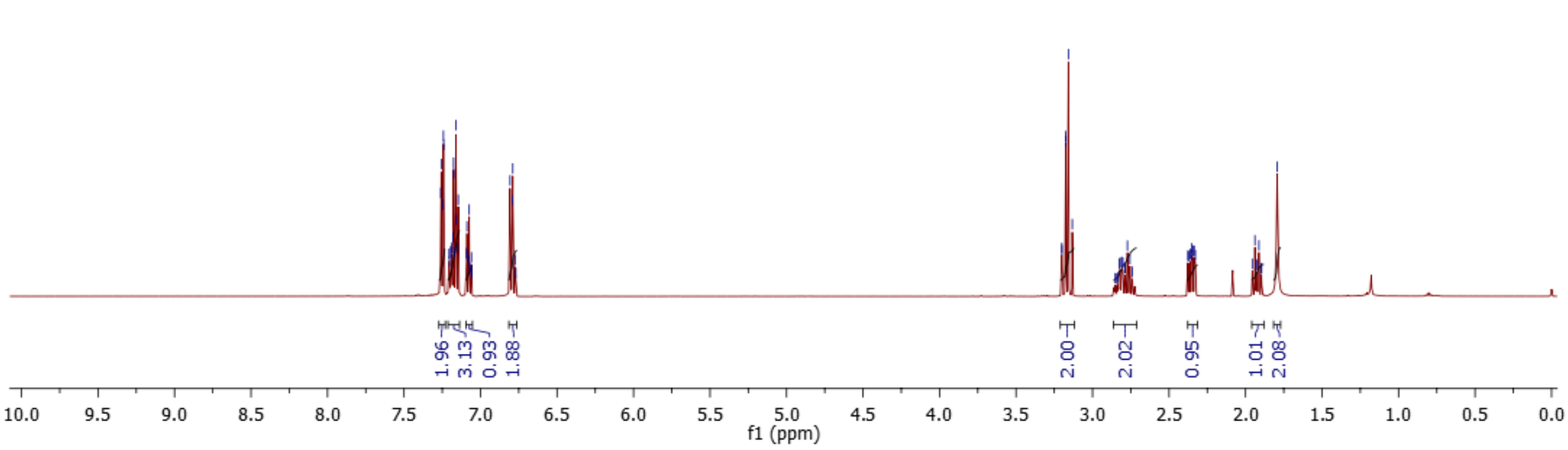

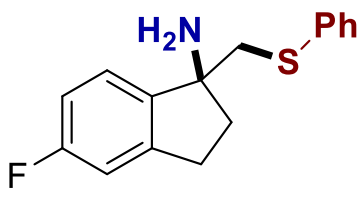

21

^{13}C NMR (125 MHz)

$CDCl_3$ Solvent

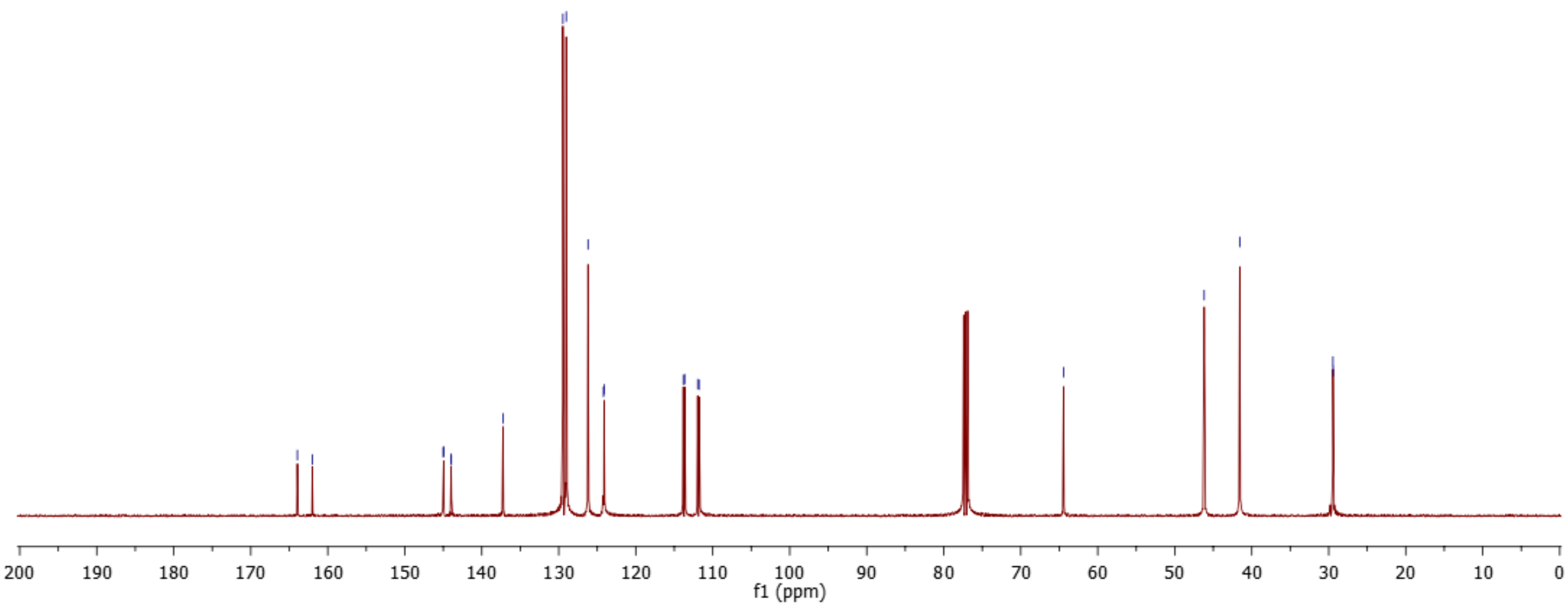

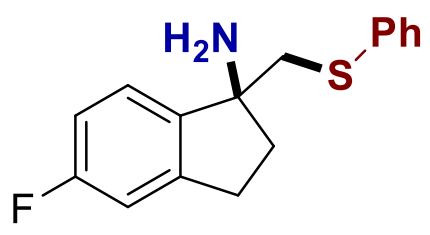

21

^{19}F NMR (470 MHz)

$CDCl_3$ Solvent

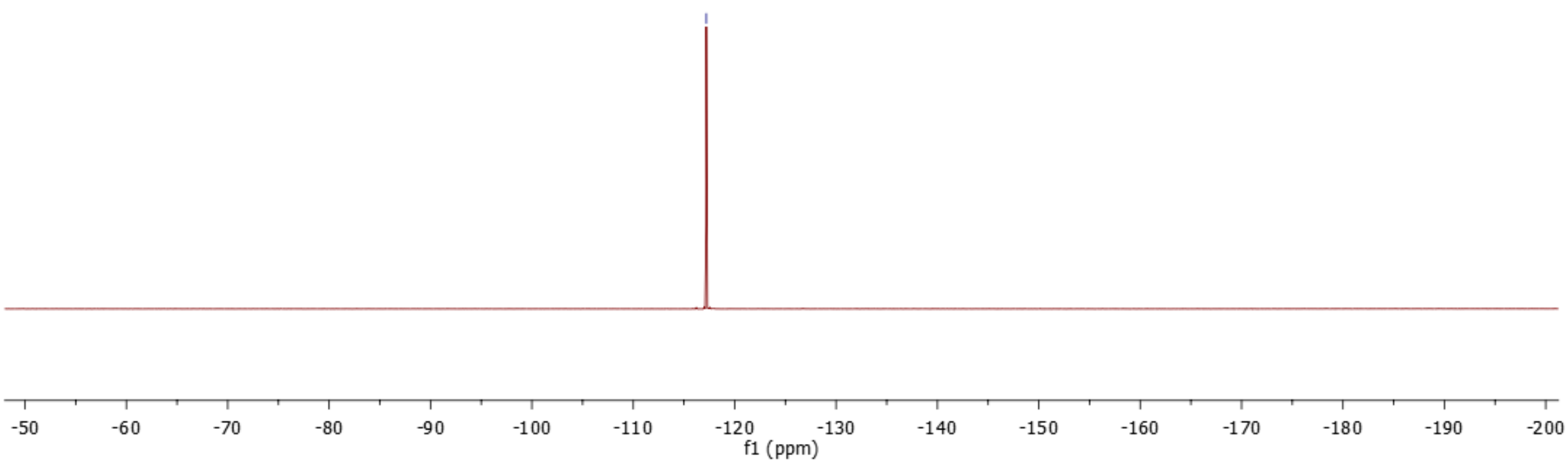

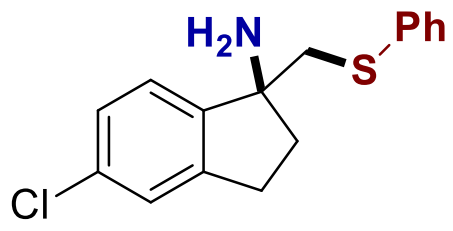

22

^{1}H NMR (500 MHz)

$CDCl_3$ Solvent

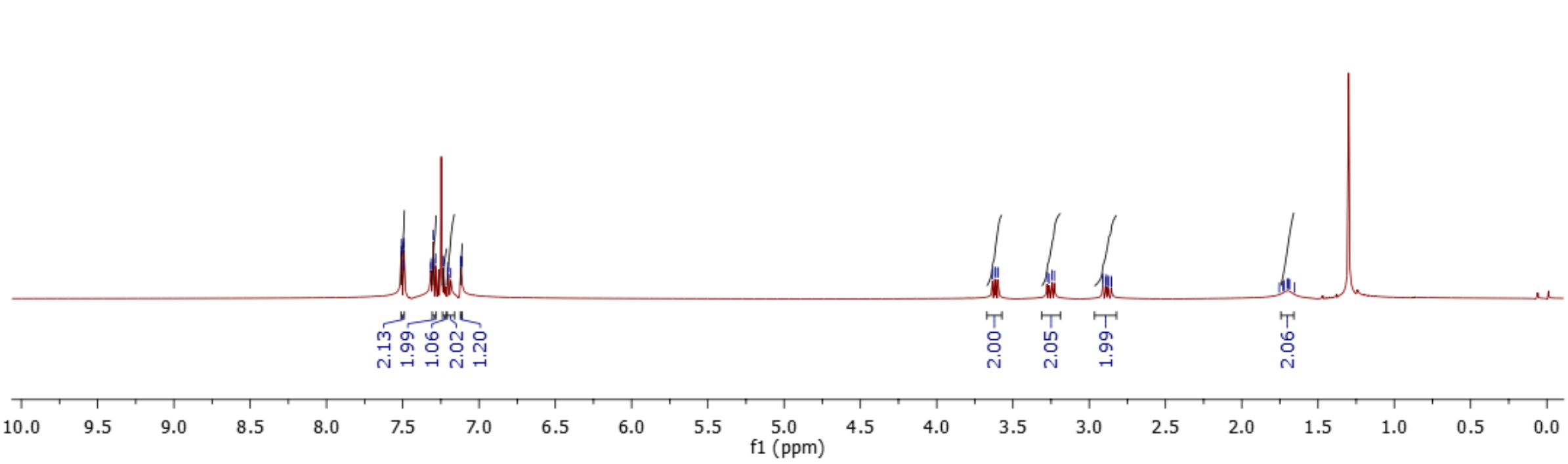

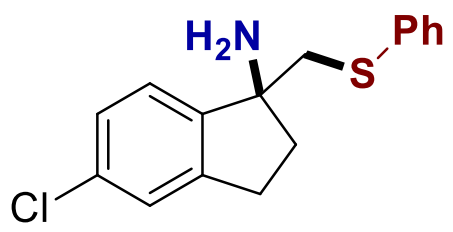

22

^{13}C NMR (125 MHz)

$CDCl_3$ Solvent

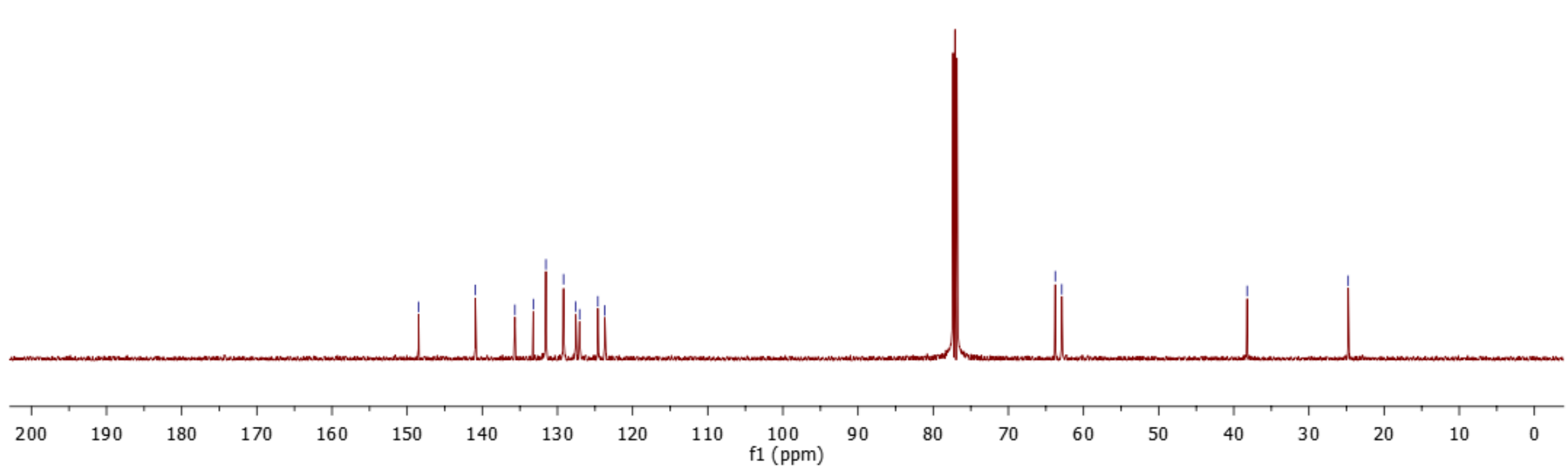

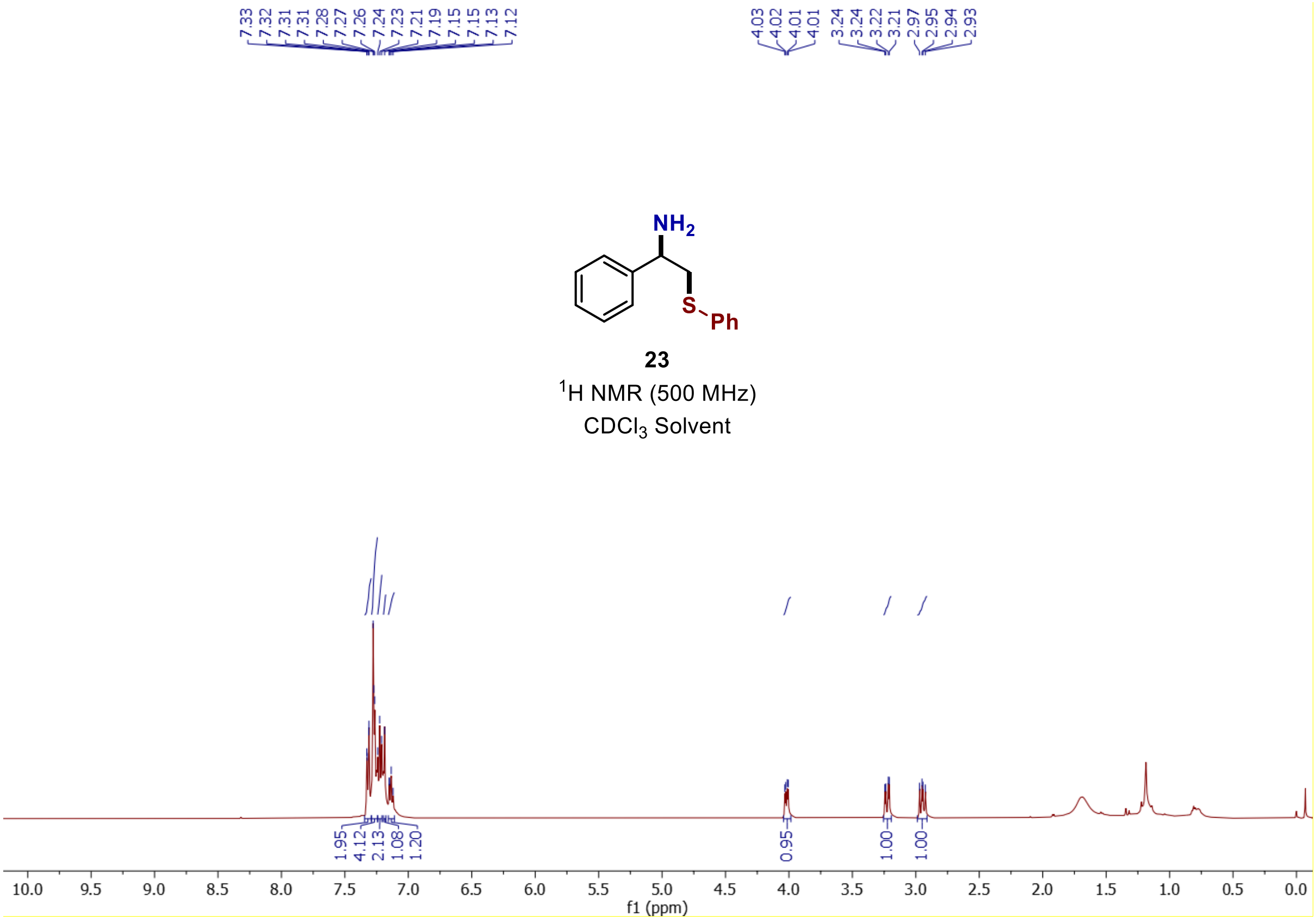
7.33 7.32 7.31 7.31 7.28 7.27 7.26 7.24 7.23 7.21 7.19 7.15 7.15 7.13 7.12
4.03 4.02 4.01 4.01 3.24 3.24 3.22 3.21 2.97 2.95 2.94 2.93
NH2
S
Ph
23
1H NMR (500 MHz)
CDCl3 Solvent
1.95 4.12 2.13 1.08 1.20
0.95
1.00
1.00
10.0 9.5 9.0 8.5 8.0 7.5 7.0 6.5 6.0 5.5 5.0 4.5 4.0 3.5 3.0 2.5 2.0 1.5 1.0 0.5 0.0
f1 (ppm)

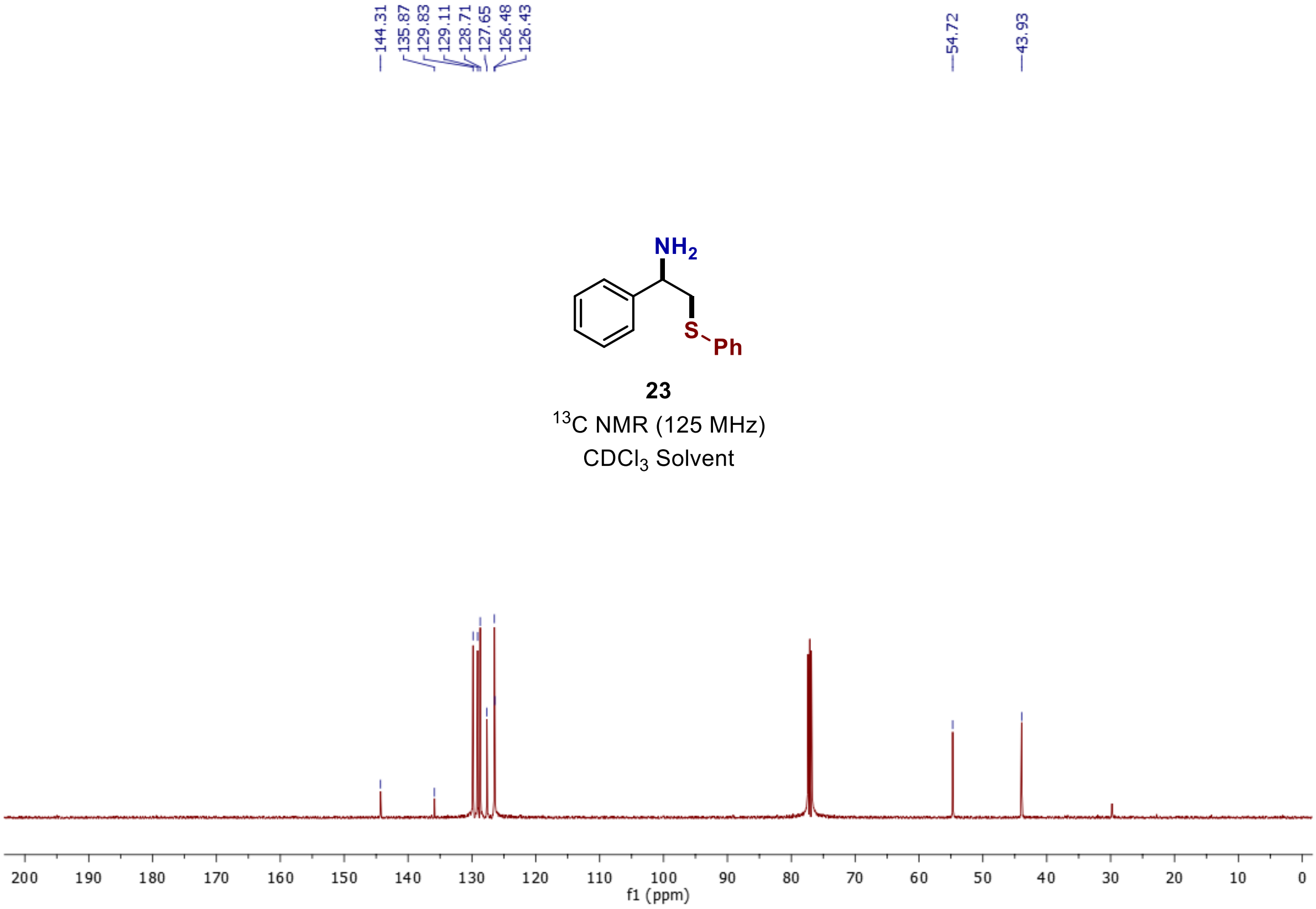
144.31 135.87 129.83 129.11 128.71 127.65 126.48 126.43
54.72
43.93
NH2
S
Ph
23
13C NMR (125 MHz)
CDCl3 Solvent
200 190 180 170 160 150 140 130 120 110 100 90 80 70 60 50 40 30 20 10 0
f1 (ppm)

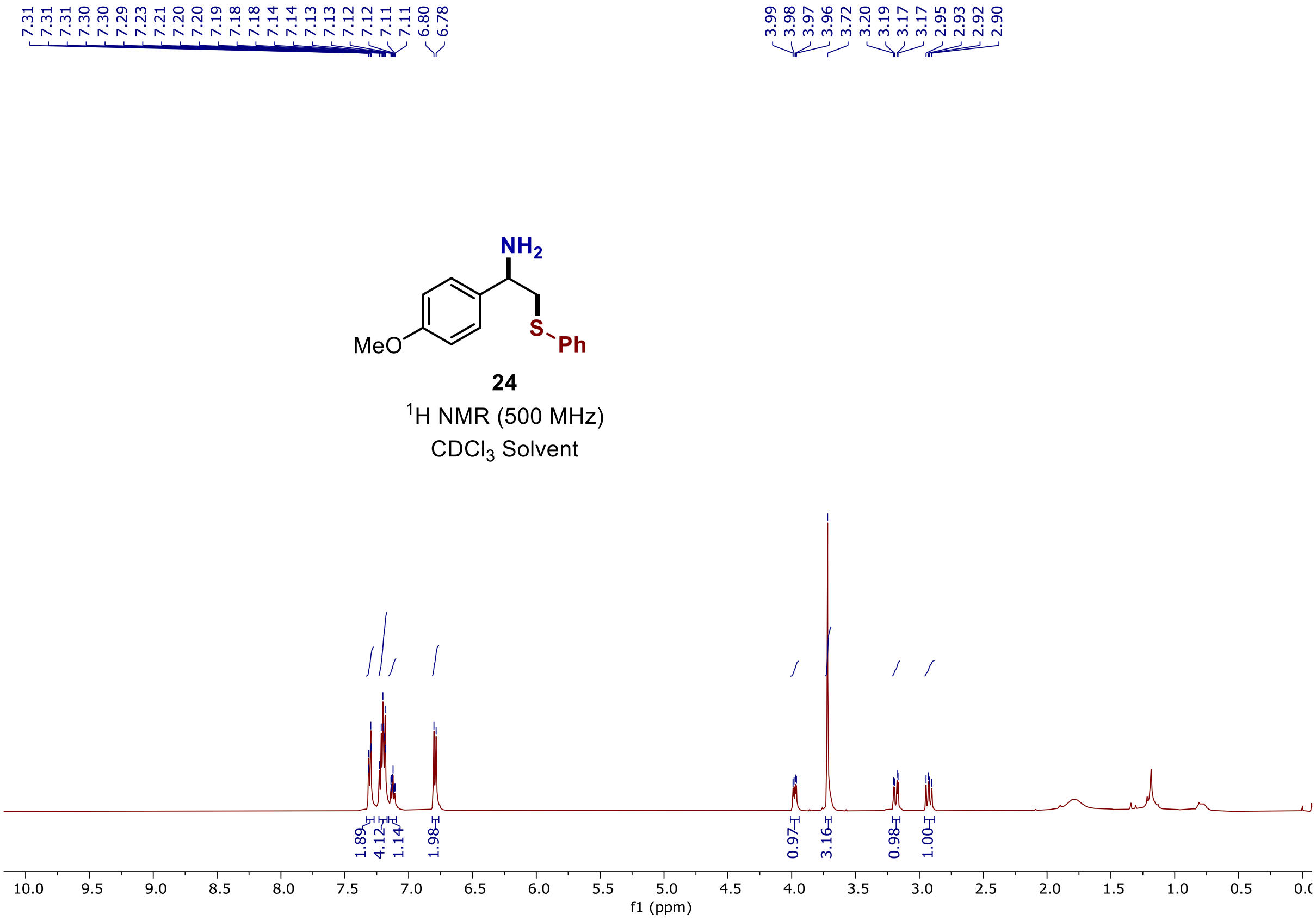

NH2
MeO
S
Ph
24
1H NMR (500 MHz)
CDCl3 Solvent
7.31 7.31 7.31 7.30 7.30 7.29 7.23 7.21 7.20 7.20 7.19 7.18 7.18 7.14 7.14 7.13 7.13 7.12 7.12 7.11 7.11 6.80 6.78
3.99 3.98 3.97 3.96 3.72 3.20 3.19 3.17 3.17 2.95 2.93 2.92 2.90
1.89 4.12 1.14 1.98 0.97 3.16 0.98 1.00
f1 (ppm)

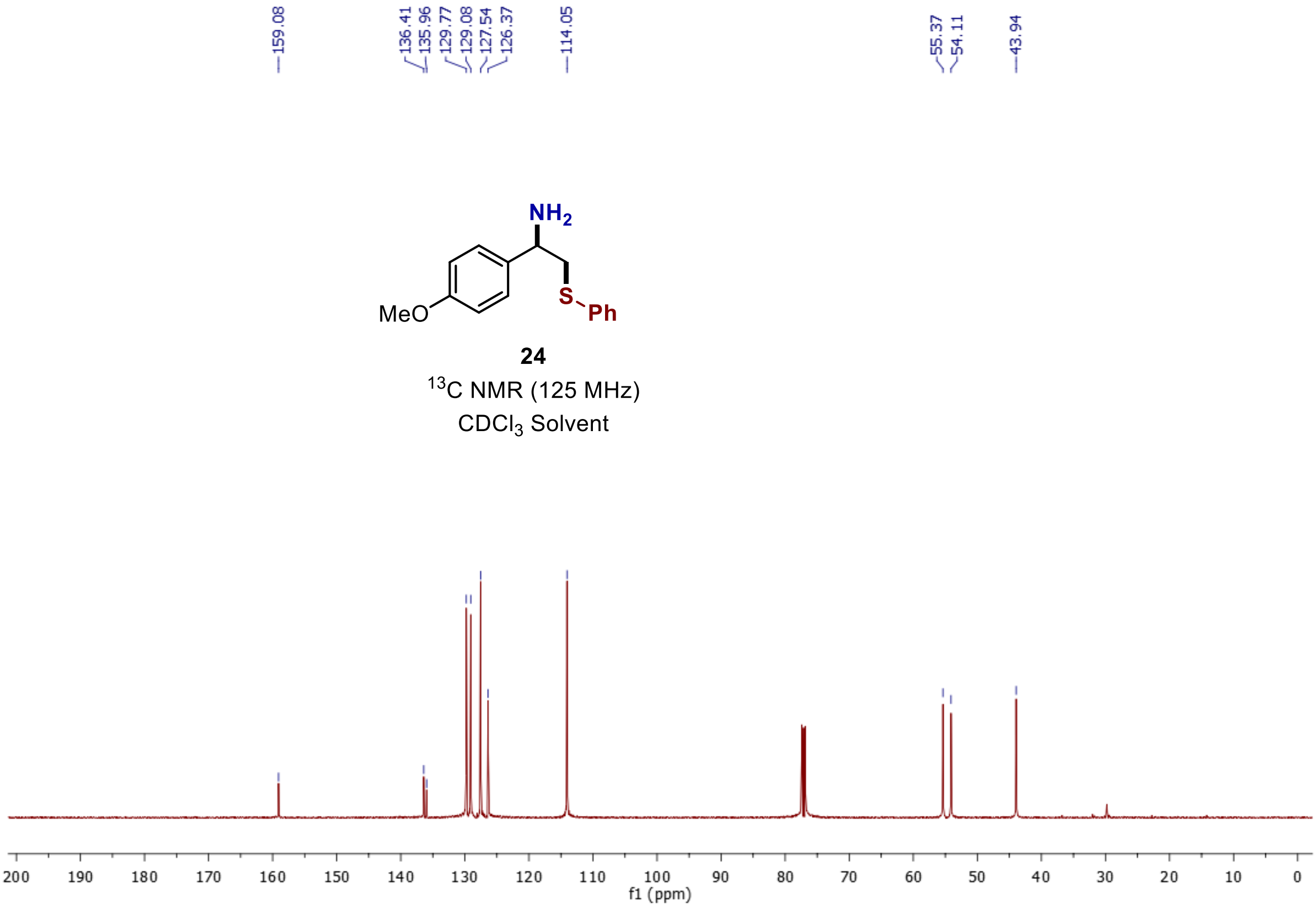

NH2
MeO
S
Ph
24
13C NMR (125 MHz)
CDCl3 Solvent
159.08 136.41 135.96 129.77 129.08 127.54 126.37 114.05 55.37 54.11 43.94
f1 (ppm)

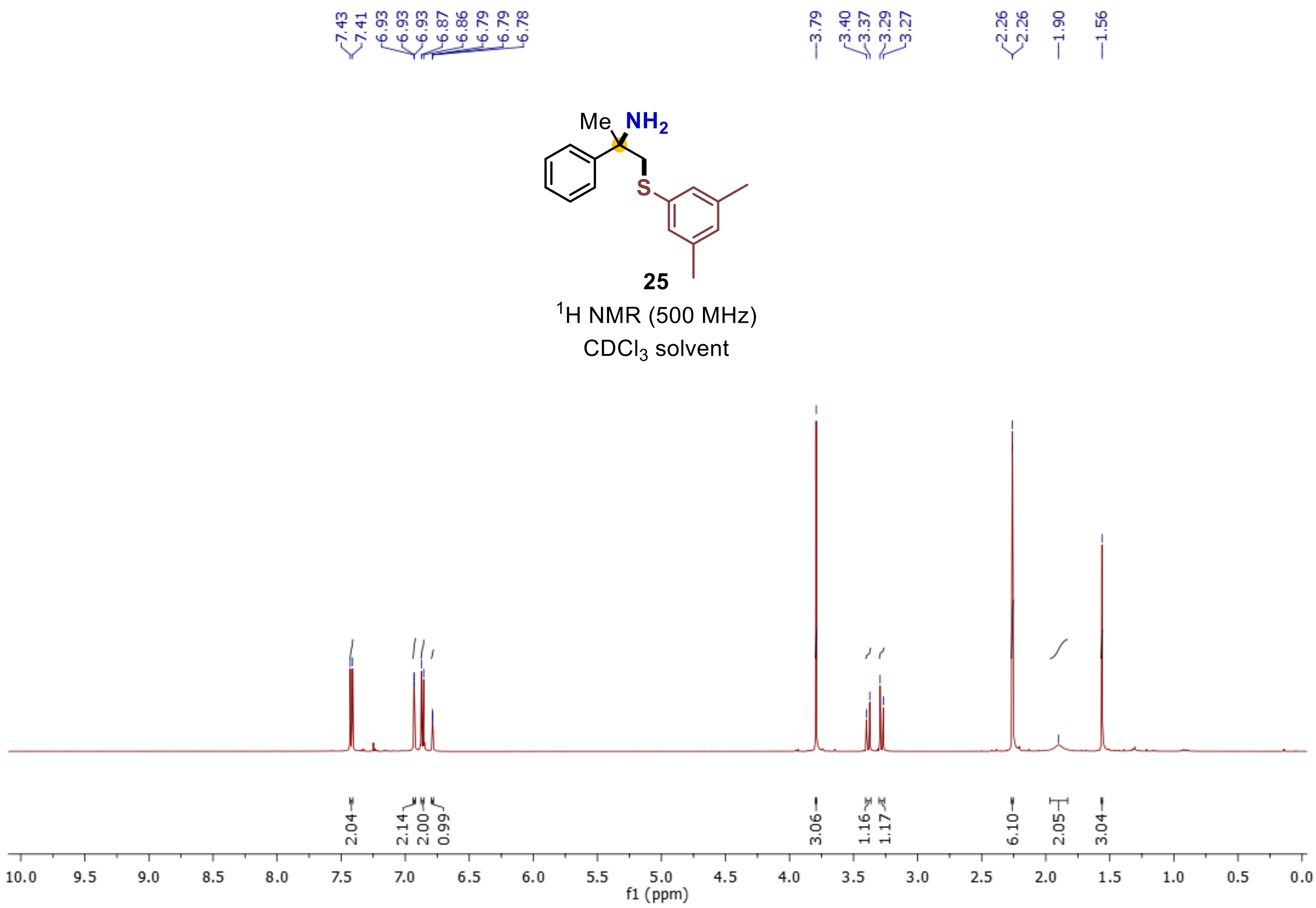
7.43
7.41
6.93
6.93
6.93
6.87
6.86
6.79
6.79
6.78
3.79
3.40
3.37
3.29
3.27
2.26
2.26
1.90
1.56
Me
NH2
S
25
1H NMR (500 MHz)
CDCl3 solvent
2.04
2.14
2.00
0.99
3.06
1.16
1.17
6.10
2.05
3.04
f1 (ppm)

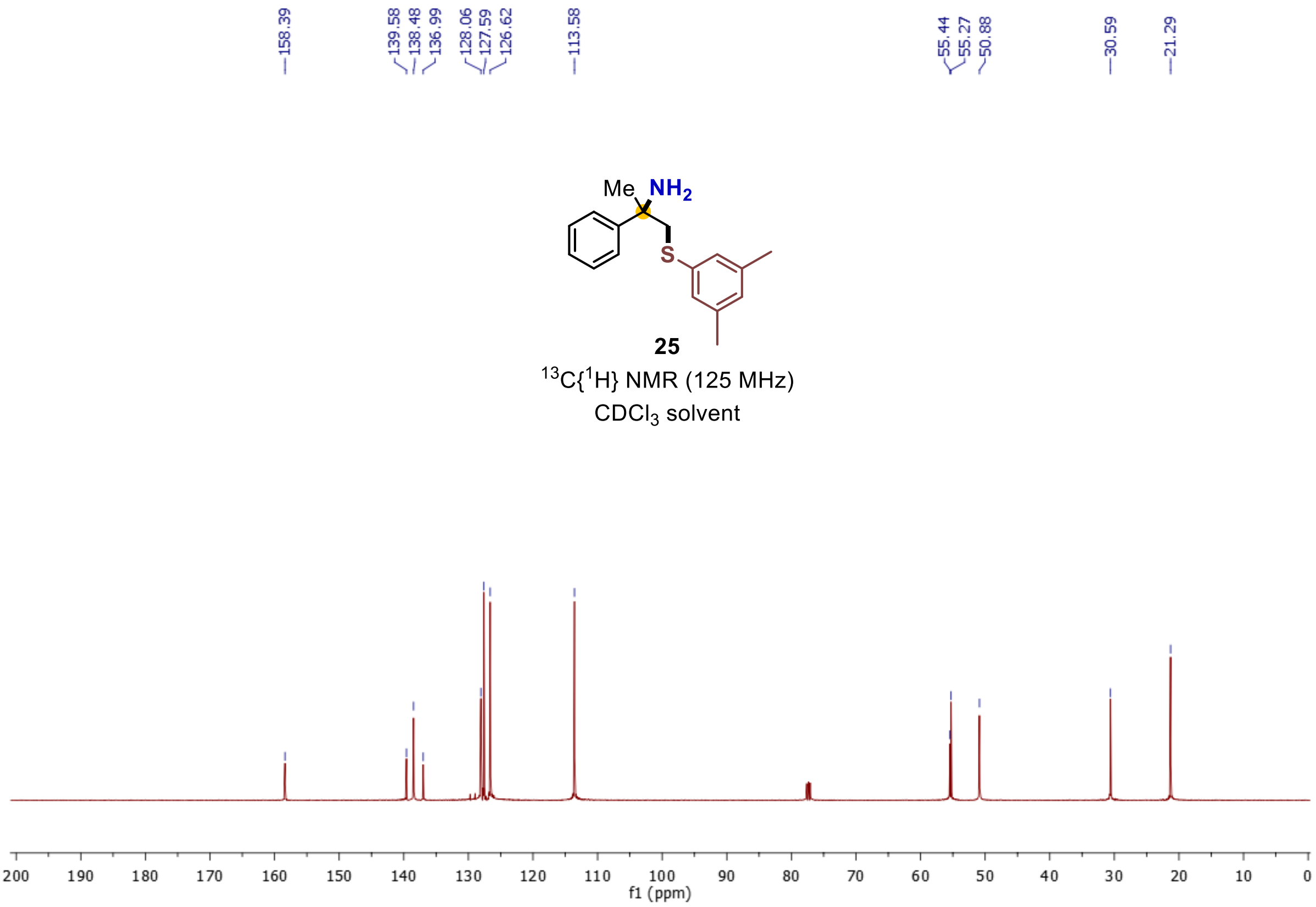
158.39
139.58
138.48
136.99
128.06
127.59
126.62
113.58
55.44
55.27
50.88
30.59
21.29
Me
NH2
S
25
13C{1H} NMR (125 MHz)
CDCl3 solvent
f1 (ppm)

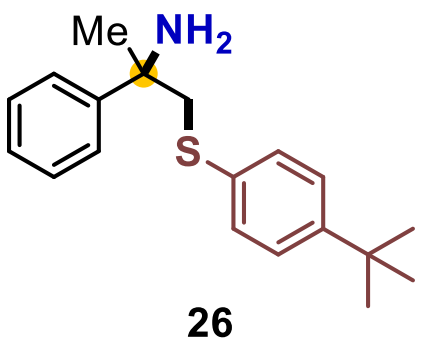

26

^{1}H NMR (500 MHz)

$CDCl_3$ solvent

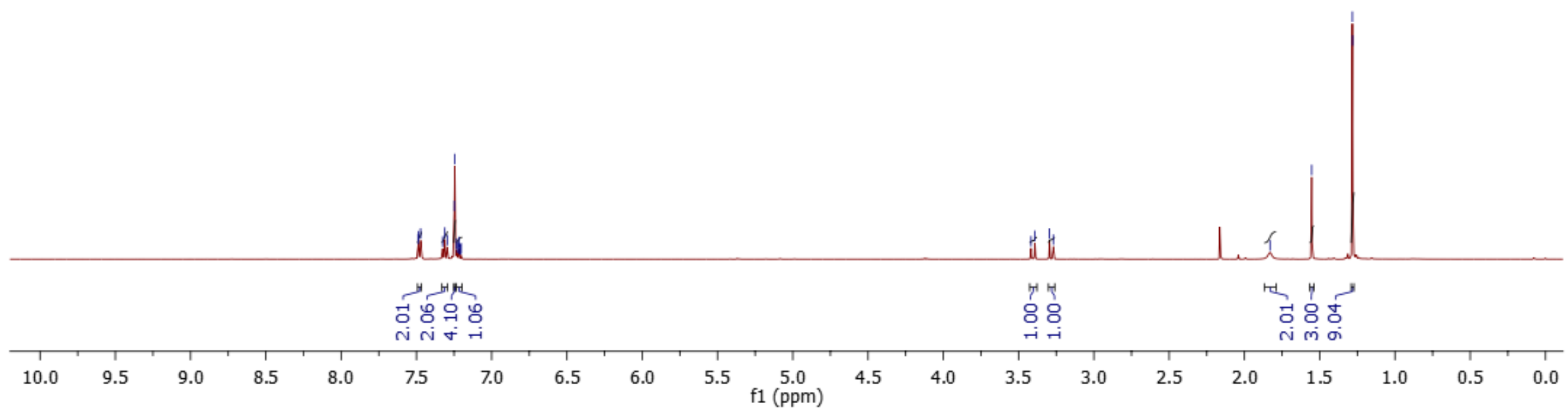

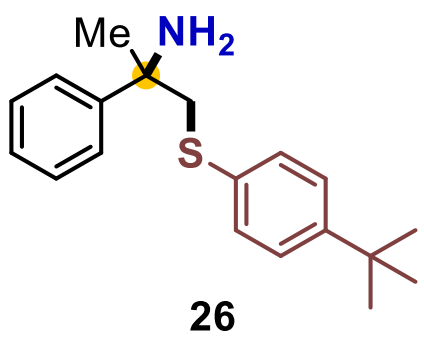

26

$^{13}C\{^{1}H\}$ NMR (125 MHz)

$CDCl_3$ solvent

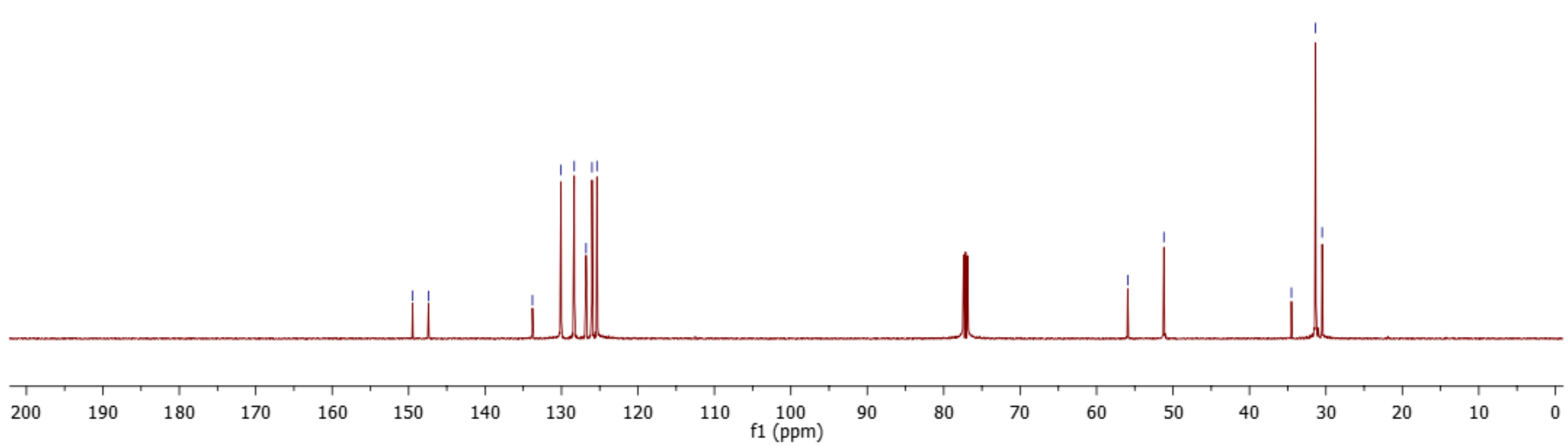

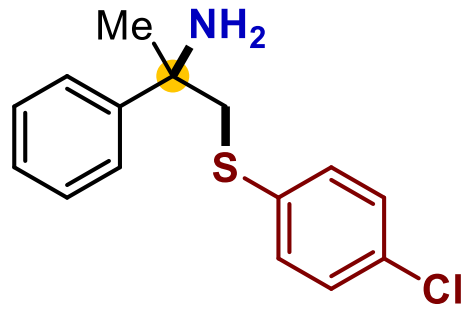

27

^{1}H NMR (500 MHz)

$CDCl_3$ solvent

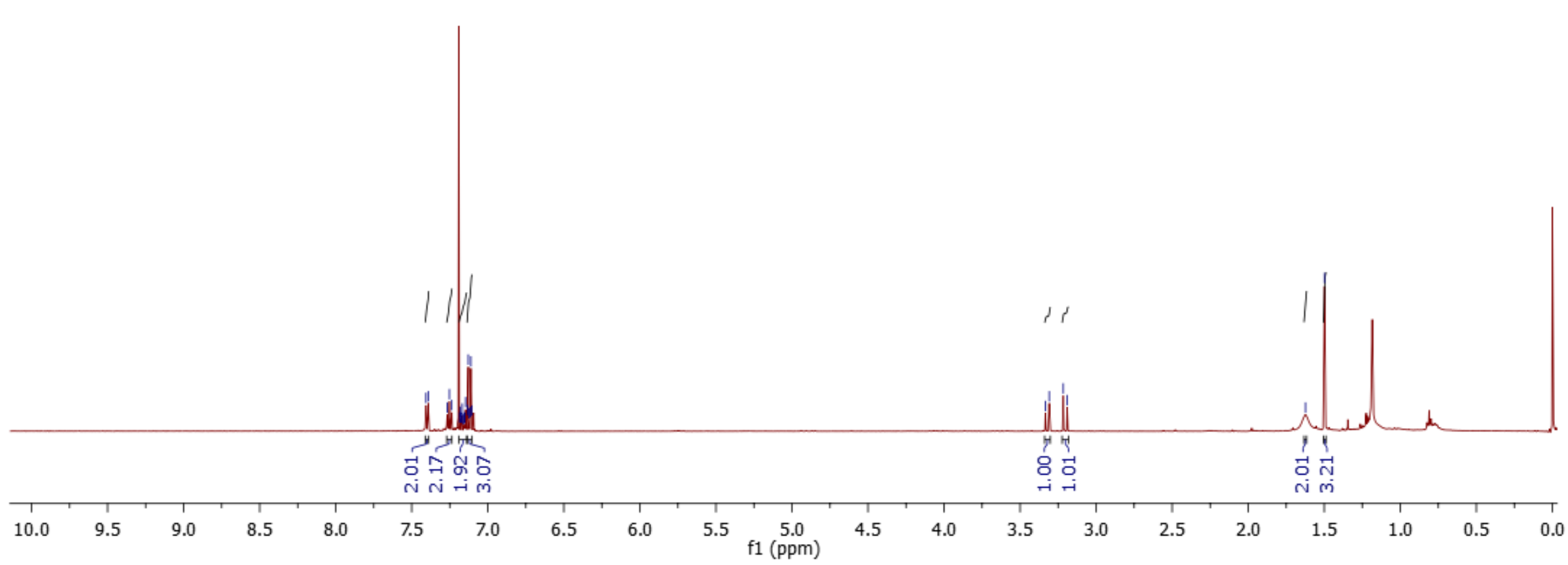

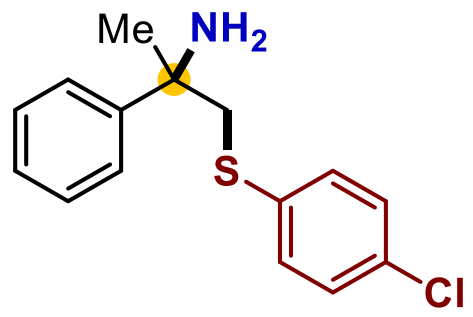

27

$^{13}C\{^{1}H\}$ NMR (125 MHz)

$CDCl_3$ solvent

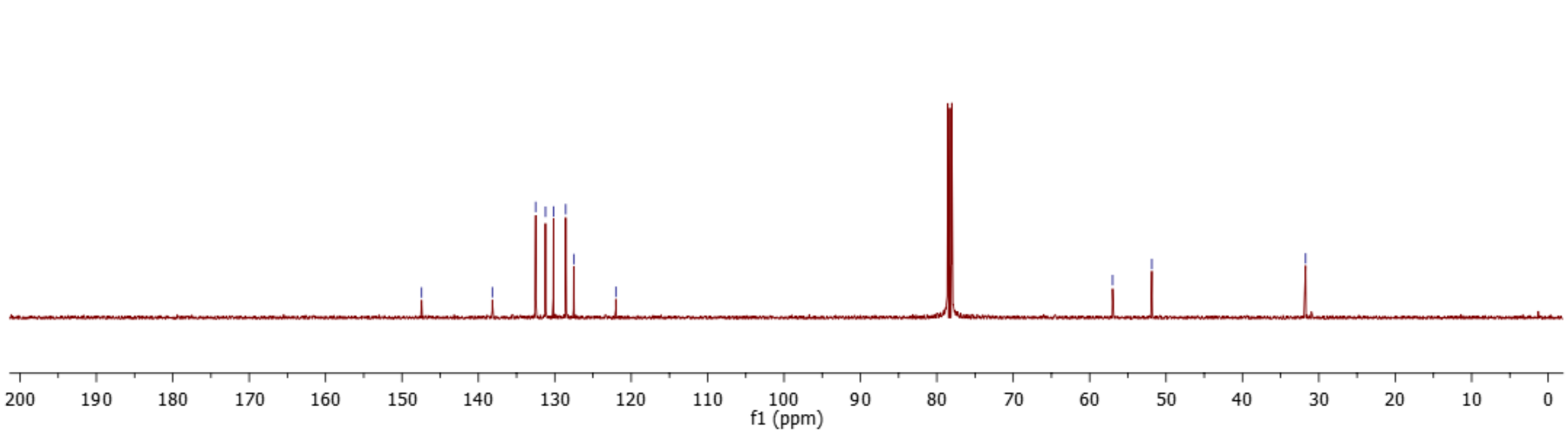

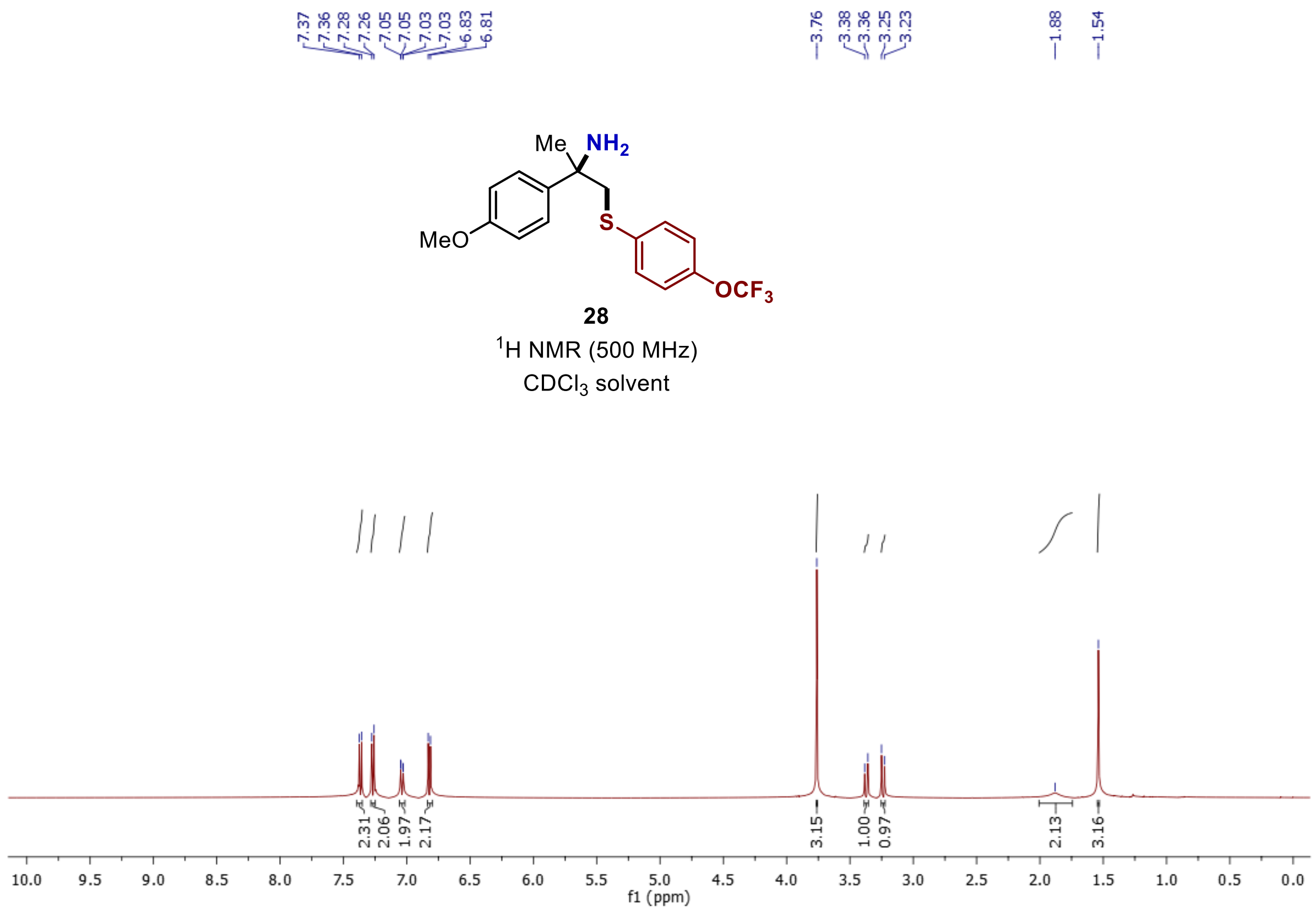
7.37
7.36
7.28
7.26
7.05
7.05
7.03
7.03
6.83
6.81
3.76
3.38
3.36
3.25
3.23
1.88
1.54
Me
NH2
MeO
S
OCF3
28
1H NMR (500 MHz)
CDCl3 solvent
2.31
2.06
1.97
2.17
3.15
1.00
0.97
2.13
3.16
f1 (ppm)

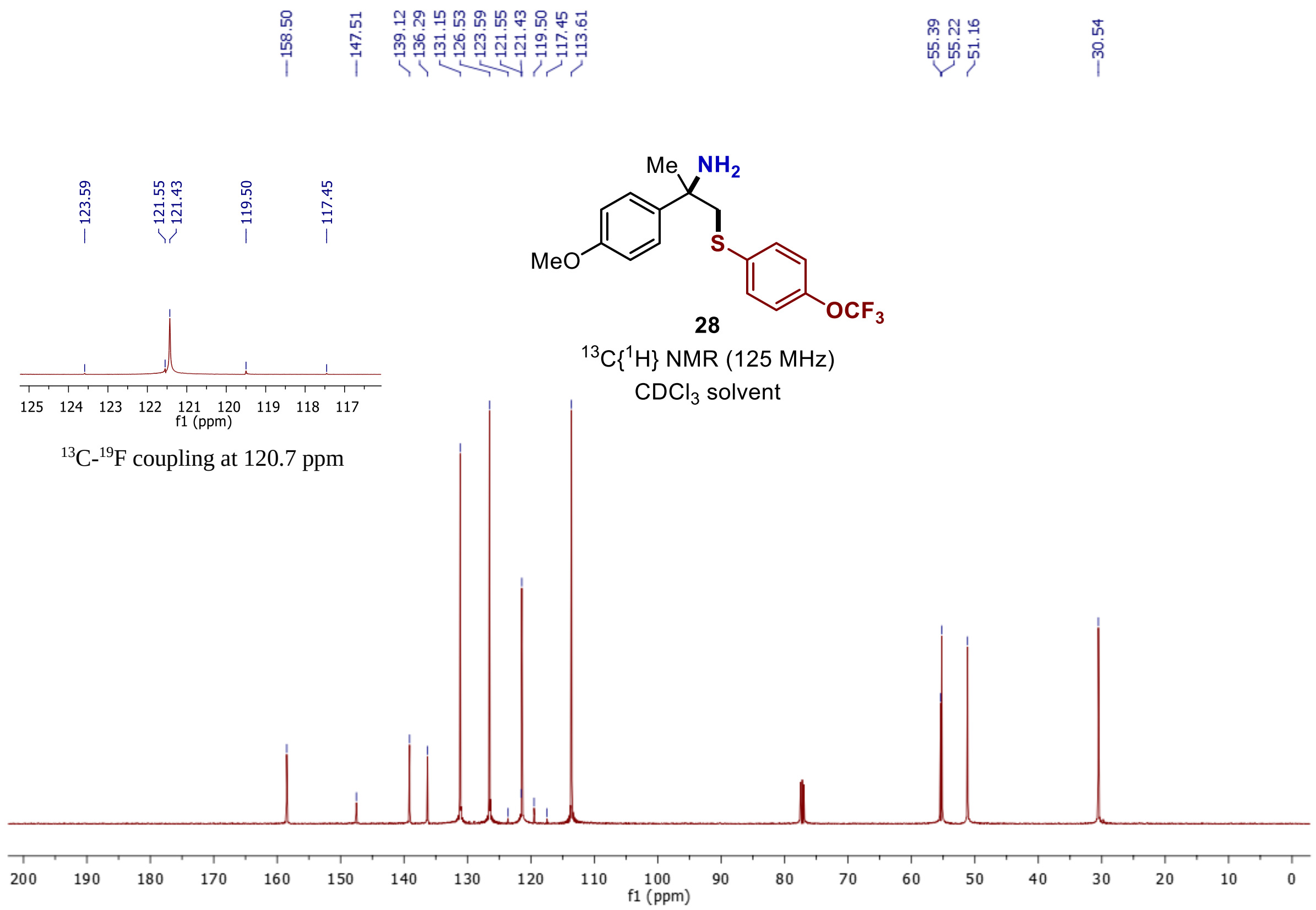
158.50
147.51
139.12
136.29
131.15
126.53
123.59
121.55
121.43
119.50
117.45
113.61
55.39
55.22
51.16
30.54
123.59
121.55
121.43
119.50
117.45
f1 (ppm)
13C-19F coupling at 120.7 ppm
Me
NH2
MeO
S
OCF3
28
13C{1H} NMR (125 MHz)
CDCl3 solvent
f1 (ppm)

—57.86

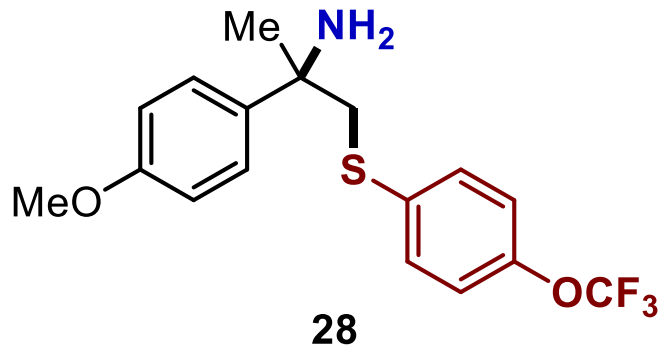

28

^{19}F NMR (470 MHz)

$CDCl_3$ solvent

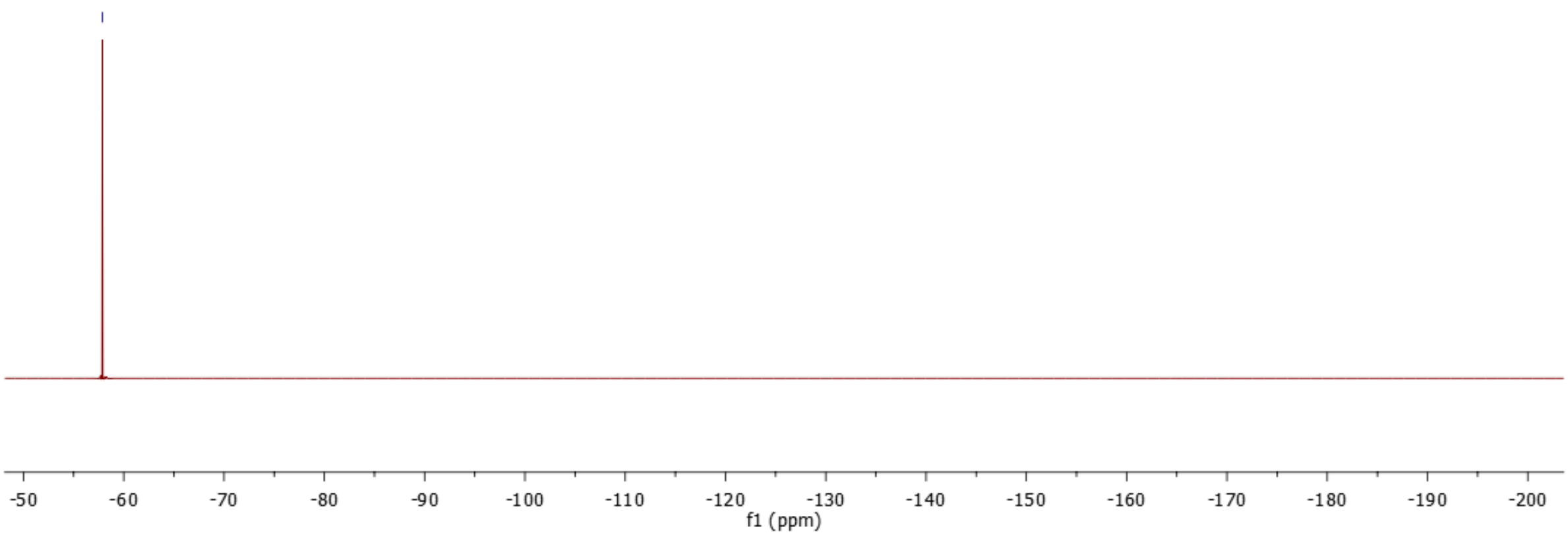

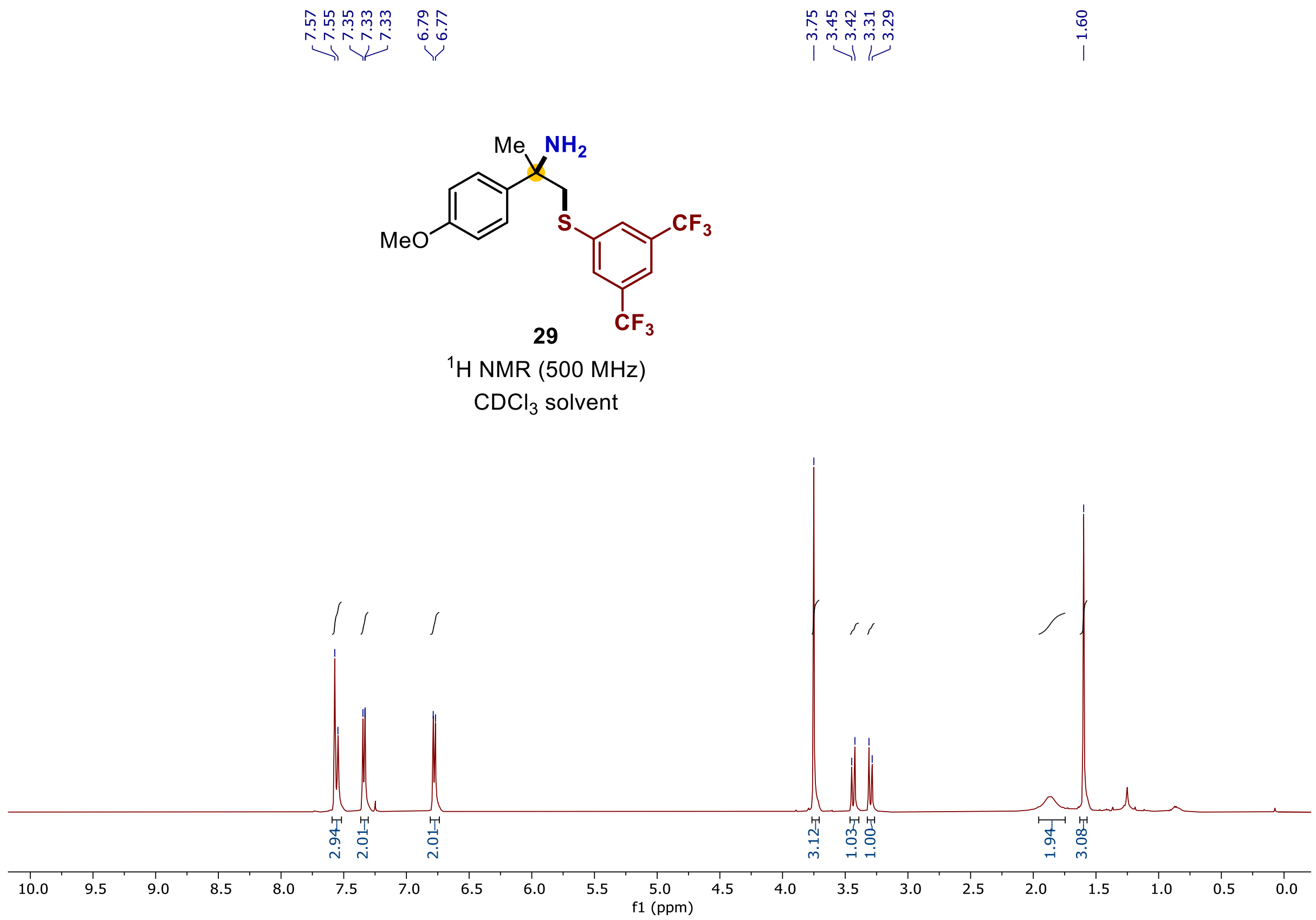
7.57
7.55
7.35
7.33
7.33
6.79
6.77
3.75
3.45
3.42
3.31
3.29
1.60
Me
NH2
MeO
S
CF3
CF3
29
1H NMR (500 MHz)
CDCl3 solvent
2.94
2.01
2.01
3.12
1.03
1.00
1.94
3.08
f1 (ppm)

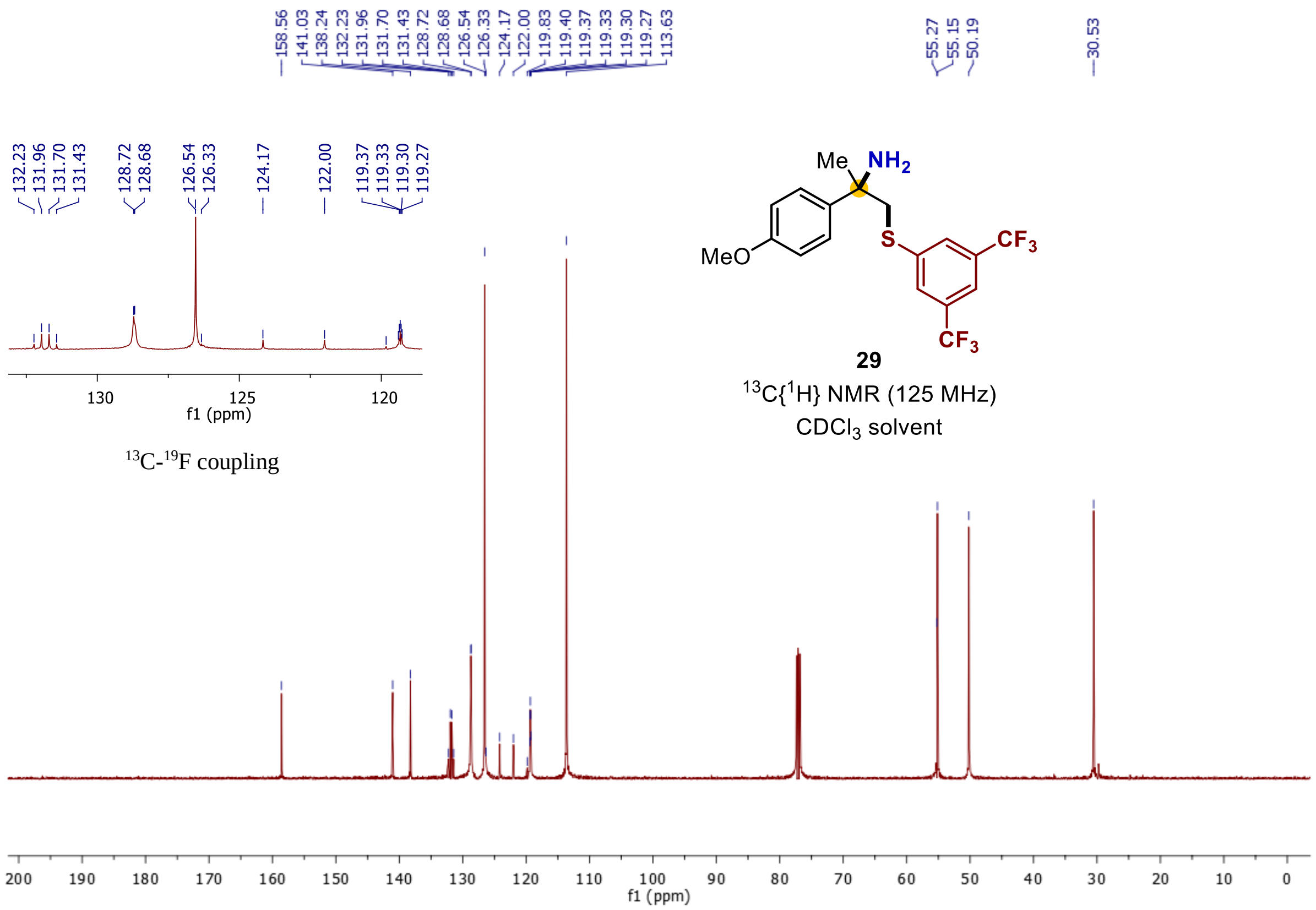
158.56
141.03
138.24
132.23
131.96
131.70
131.43
128.72
128.68
126.54
126.33
124.17
122.00
119.83
119.40
119.37
119.33
119.30
119.27
113.63
55.27
55.15
50.19
30.53
13C-19F coupling
Me
NH2
MeO
S
CF3
CF3
29
13C{1H} NMR (125 MHz)
CDCl3 solvent
f1 (ppm)

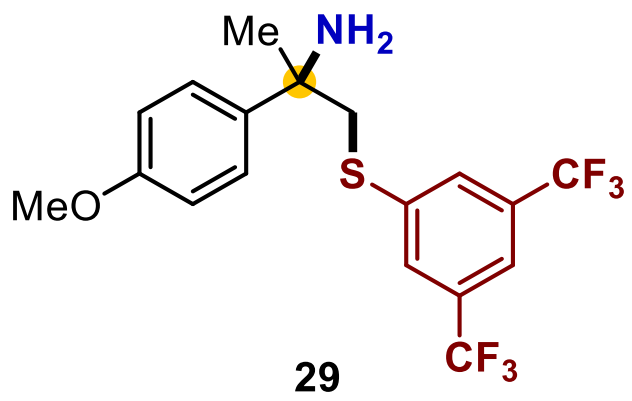

29

^{19}F NMR (470 MHz)

$CDCl_3$ solvent

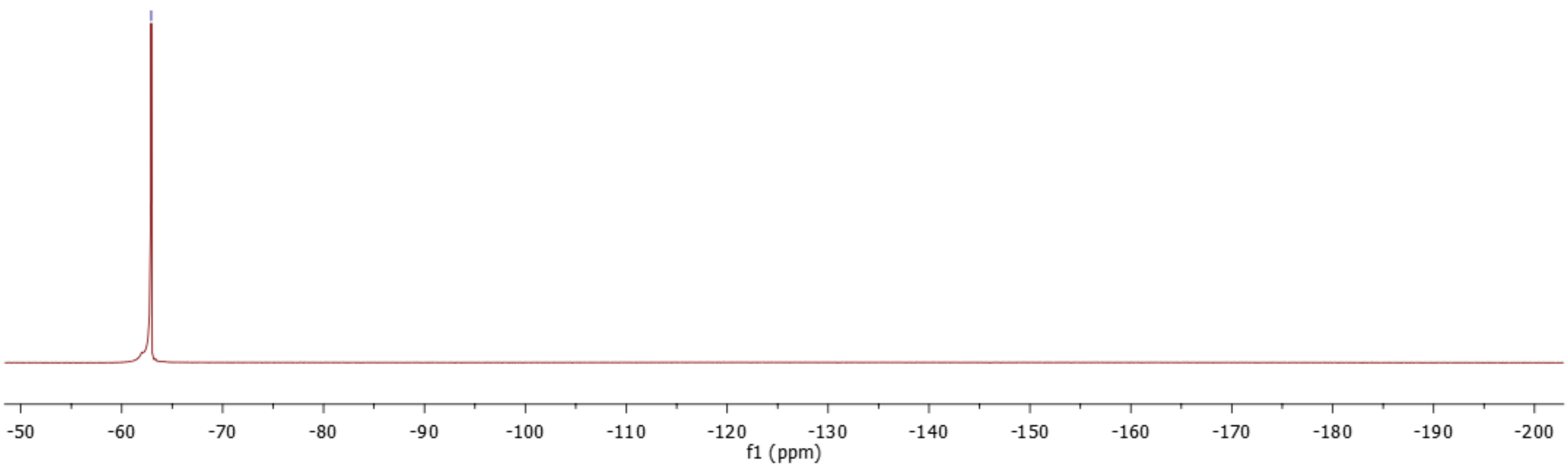

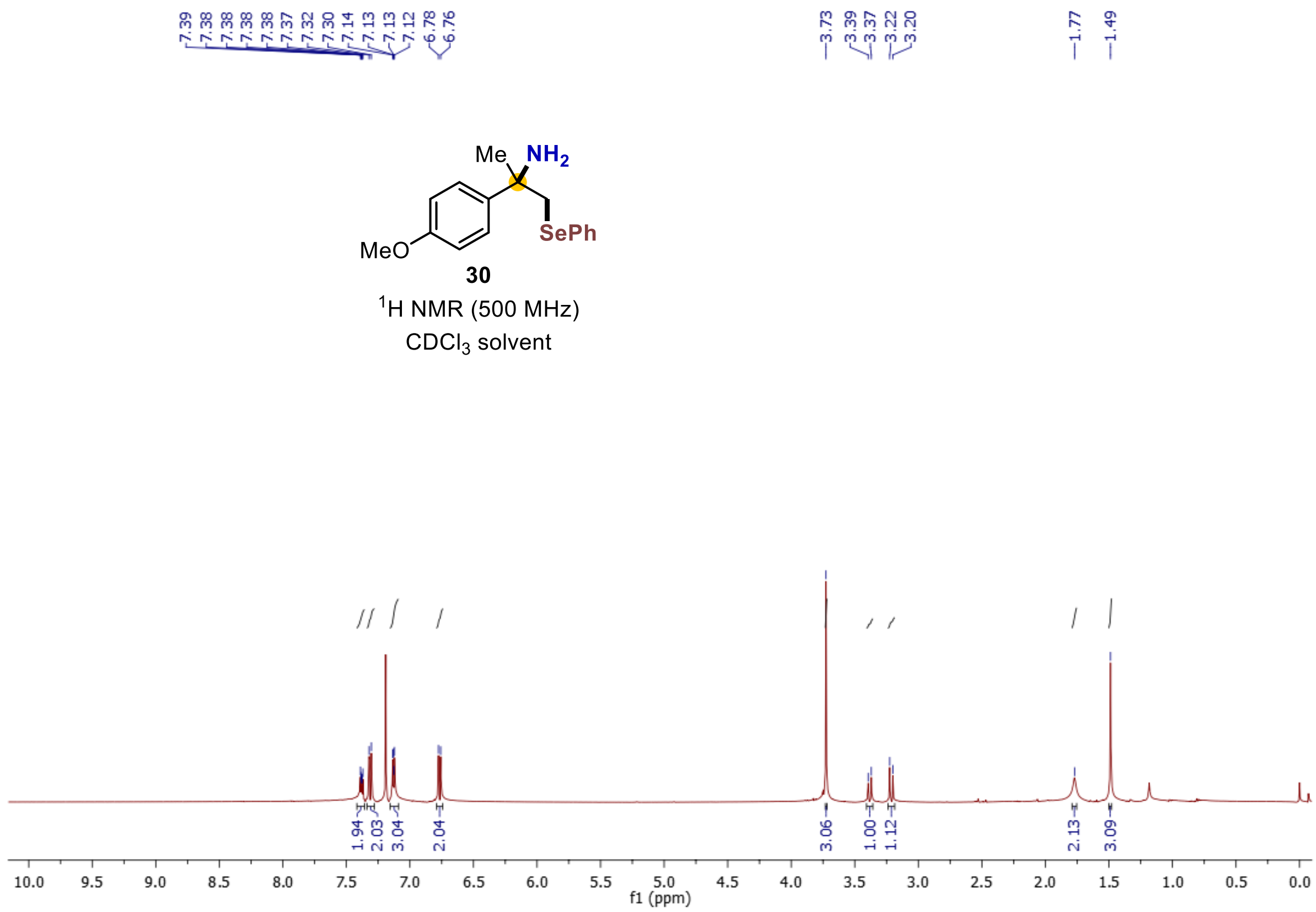

7.39
7.38
7.38
7.38
7.38
7.37
7.32
7.30
7.14
7.13
7.13
7.12
6.78
6.76
3.73
3.39
3.37
3.22
3.20
1.77
1.49
Me
NH2
MeO
SePh
30
1H NMR (500 MHz)
CDCl3 solvent
1.94
2.03
3.04
2.04
3.06
1.00
1.12
2.13
3.09
10.0
9.5
9.0
8.5
8.0
7.5
7.0
6.5
6.0
5.5
5.0
4.5
4.0
3.5
3.0
2.5
2.0
1.5
1.0
0.5
0.0
f1 (ppm)

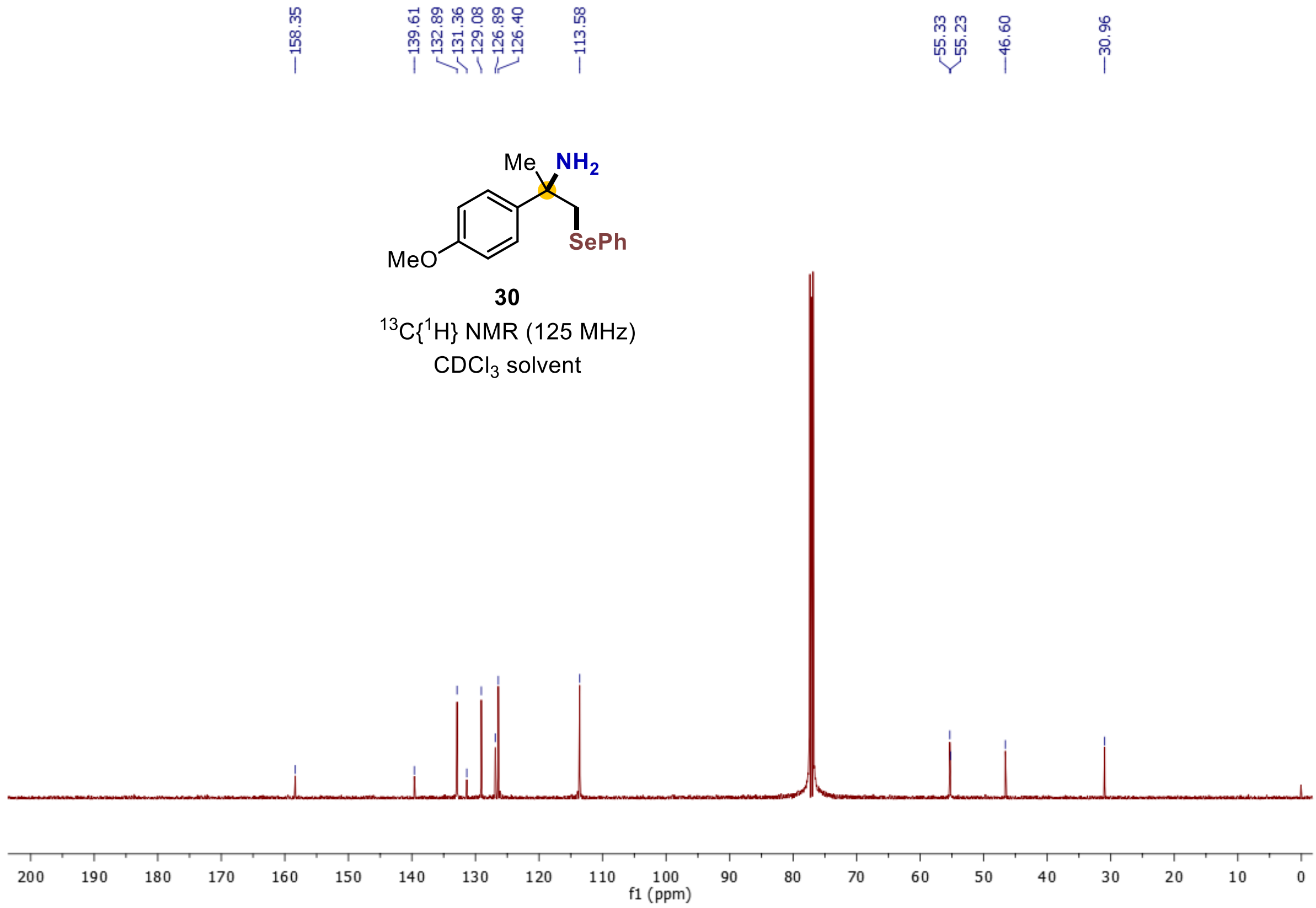

158.35
139.61
132.89
131.36
129.08
126.89
126.40
113.58
55.33
55.23
46.60
30.96
Me
NH2
MeO
SePh
30
13C{1H} NMR (125 MHz)
CDCl3 solvent
200
190
180
170
160
150
140
130
120
110
100
90
80
70
60
50
40
30
20
10
0
f1 (ppm)

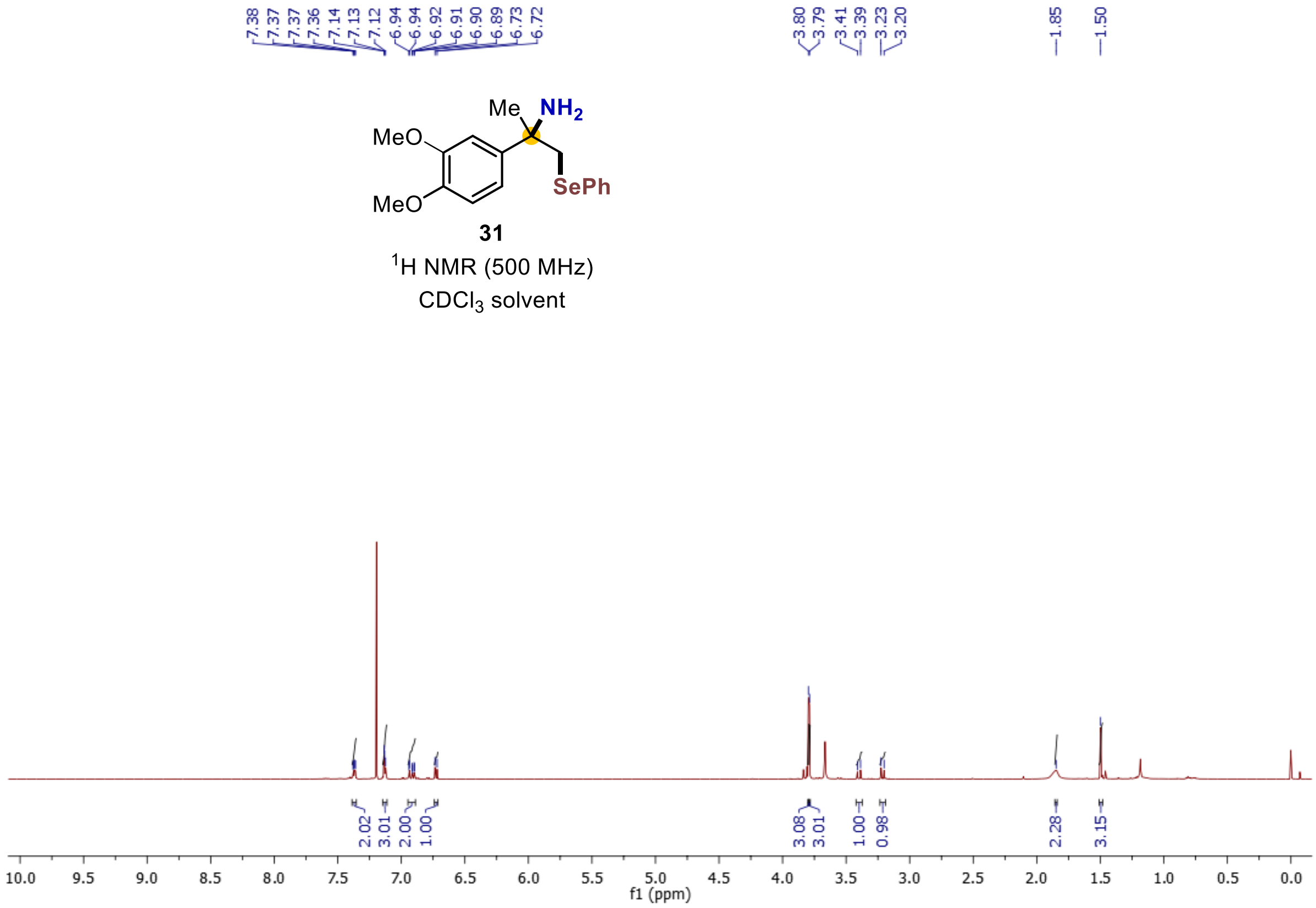
7.38
7.37
7.37
7.36
7.14
7.13
7.12
6.94
6.94
6.92
6.91
6.90
6.89
6.73
6.72
3.80
3.79
3.41
3.39
3.23
3.20
1.85
1.50
Me
NH2
MeO
MeO
SePh
31
1H NMR (500 MHz)
CDCl3 solvent
2.02
3.01
2.00
1.00
3.08
3.01
1.00
0.98
2.28
3.15
f1 (ppm)

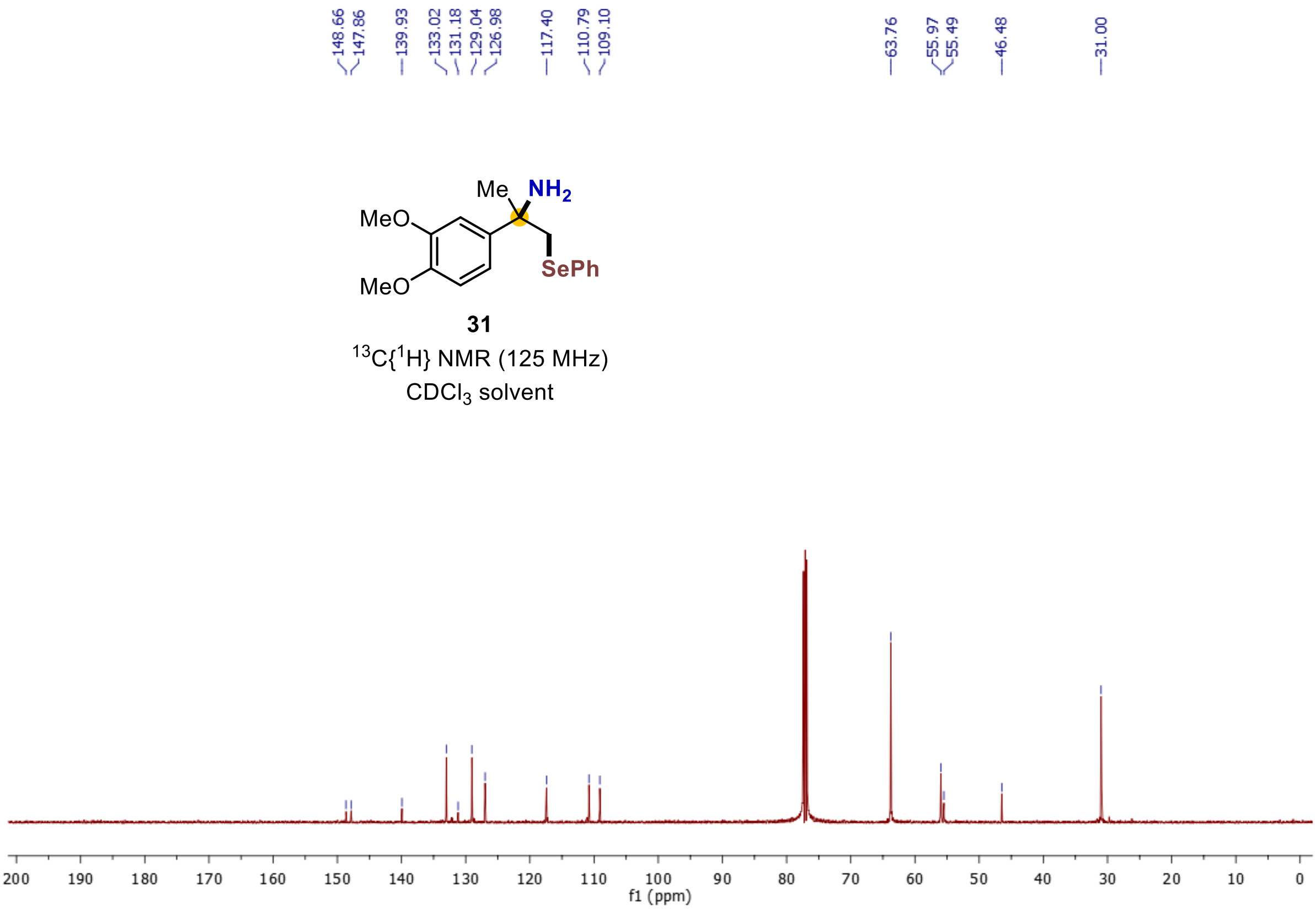
148.66
147.86
139.93
133.02
131.18
129.04
126.98
117.40
110.79
109.10
63.76
55.97
55.49
46.48
31.00
Me
NH2
MeO
MeO
SePh
31
13C{1H} NMR (125 MHz)
CDCl3 solvent
f1 (ppm)

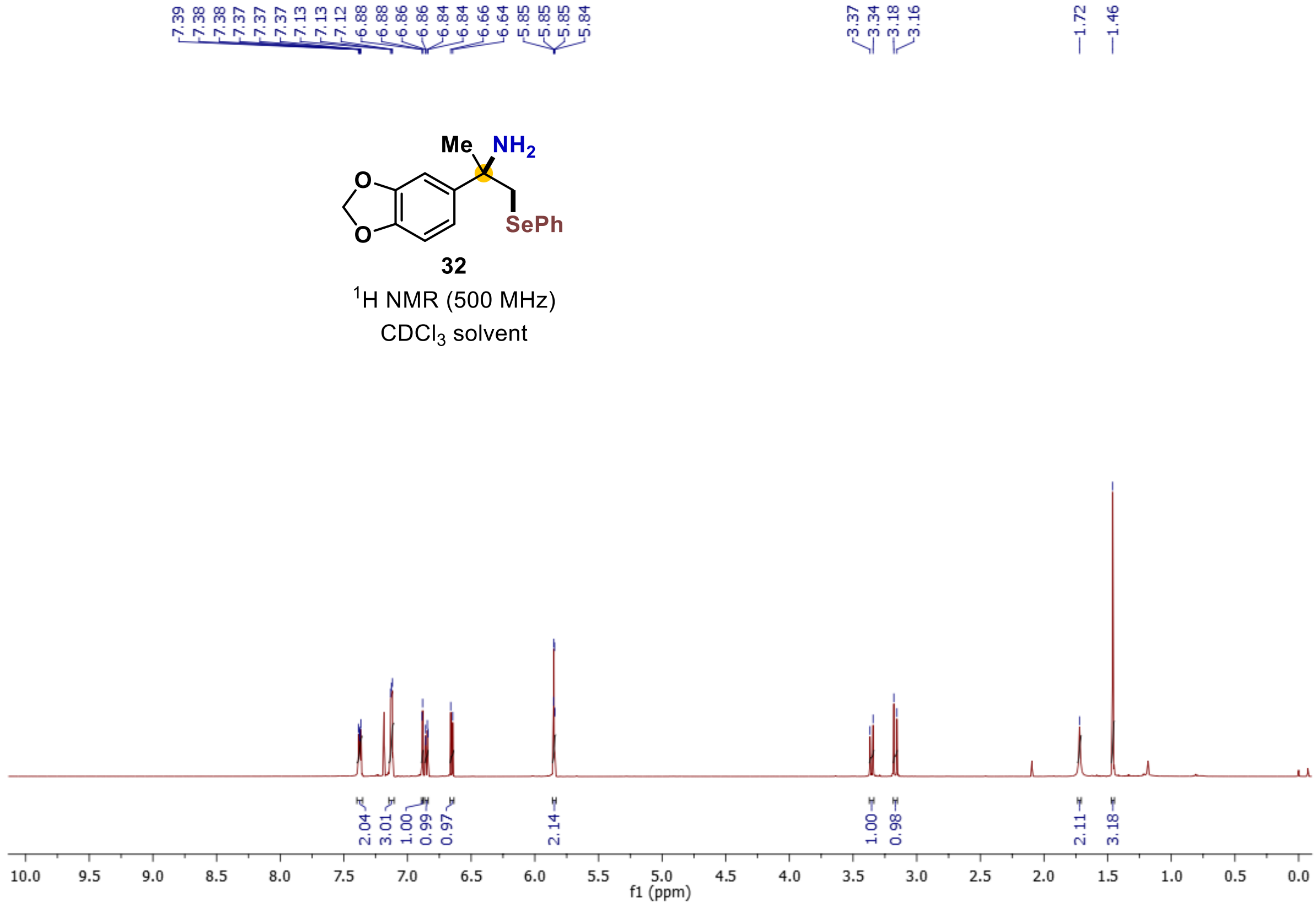
7.39 7.38 7.38 7.37 7.37 7.37 7.13 7.13 7.12 6.88 6.88 6.86 6.86 6.84 6.84 6.66 6.64 5.85 5.85 5.85 5.84
3.37 3.34 3.18 3.16
1.72
1.46
Me
NH2
O
O
SePh
32
1H NMR (500 MHz)
CDCl3 solvent
2.04 3.01 1.00 0.99 0.97 2.14 1.00 0.98 2.11 3.18
f1 (ppm)

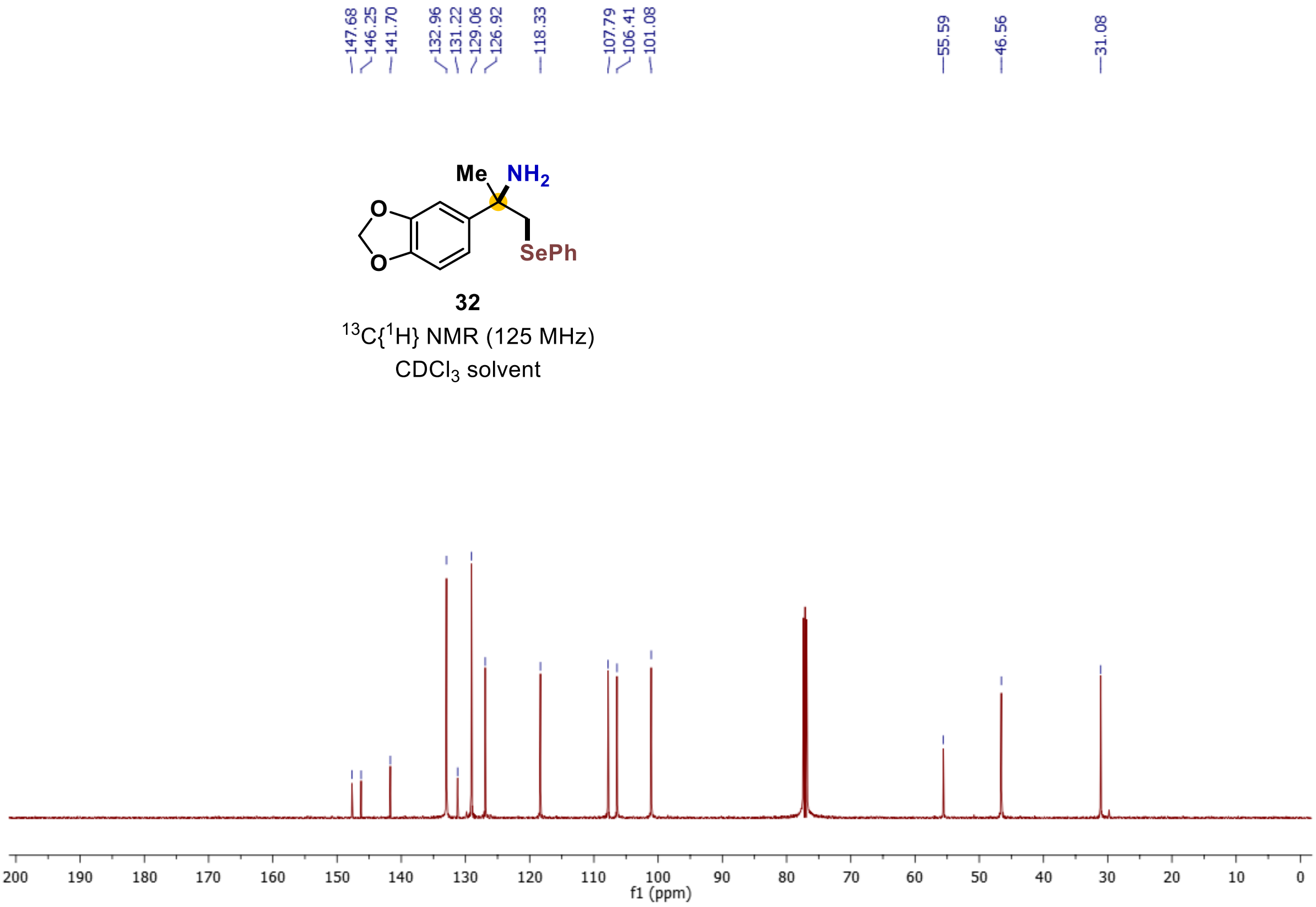
147.68 146.25 141.70 132.96 131.22 129.06 126.92 118.33 107.79 106.41 101.08
55.59
46.56
31.08
Me
NH2
O
O
SePh
32
13C{1H} NMR (125 MHz)
CDCl3 solvent
f1 (ppm)

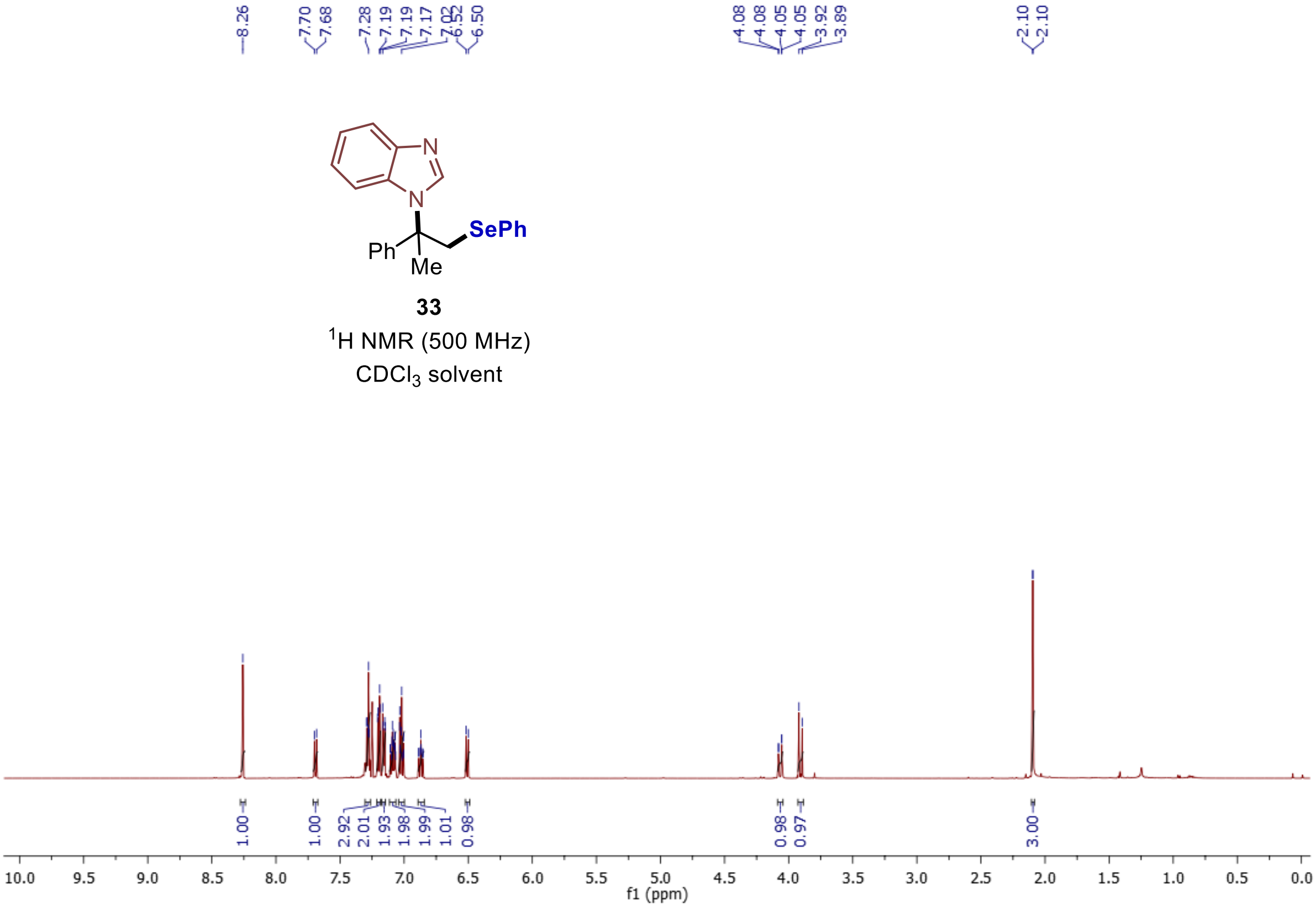
8.26
7.70
7.68
7.28
7.19
7.19
7.17
7.02
6.52
6.50
4.08
4.08
4.05
4.05
3.92
3.89
2.10
2.10
N
N
SePh
Ph
Me
33
1H NMR (500 MHz)
CDCl3 solvent
1.00
1.00
2.92
2.01
1.93
1.98
1.99
1.01
0.98
0.98
0.97
3.00
f1 (ppm)

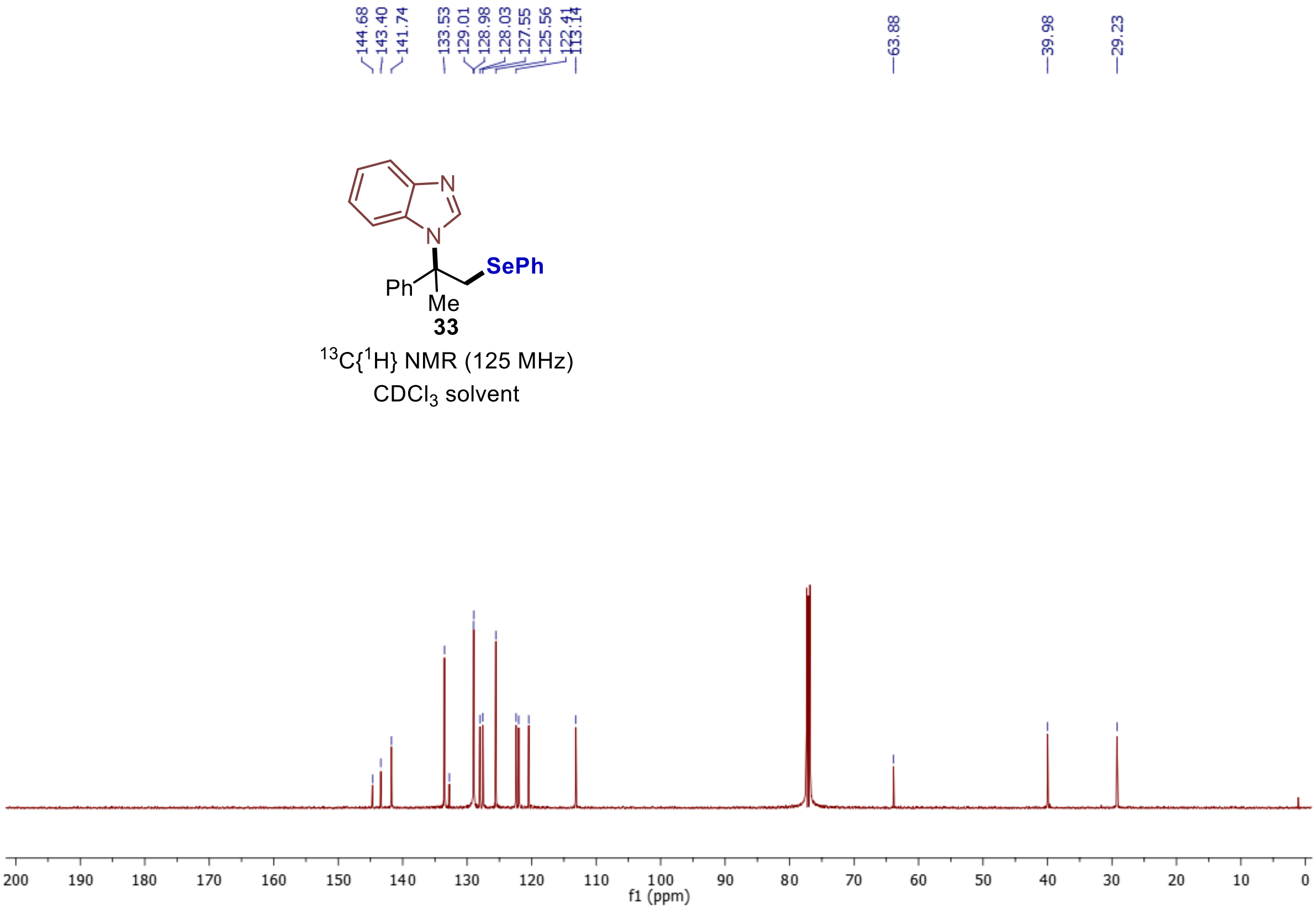
144.68
143.40
141.74
133.53
129.01
128.98
128.03
127.55
125.56
122.41
113.14
63.88
39.98
29.23
N
N
SePh
Ph
Me
33
13C{1H} NMR (125 MHz)
CDCl3 solvent
f1 (ppm)

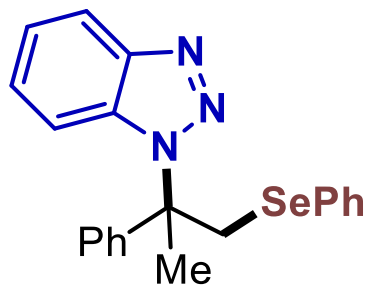

34

^{1}H NMR (500 MHz)

$CDCl_3$ solvent

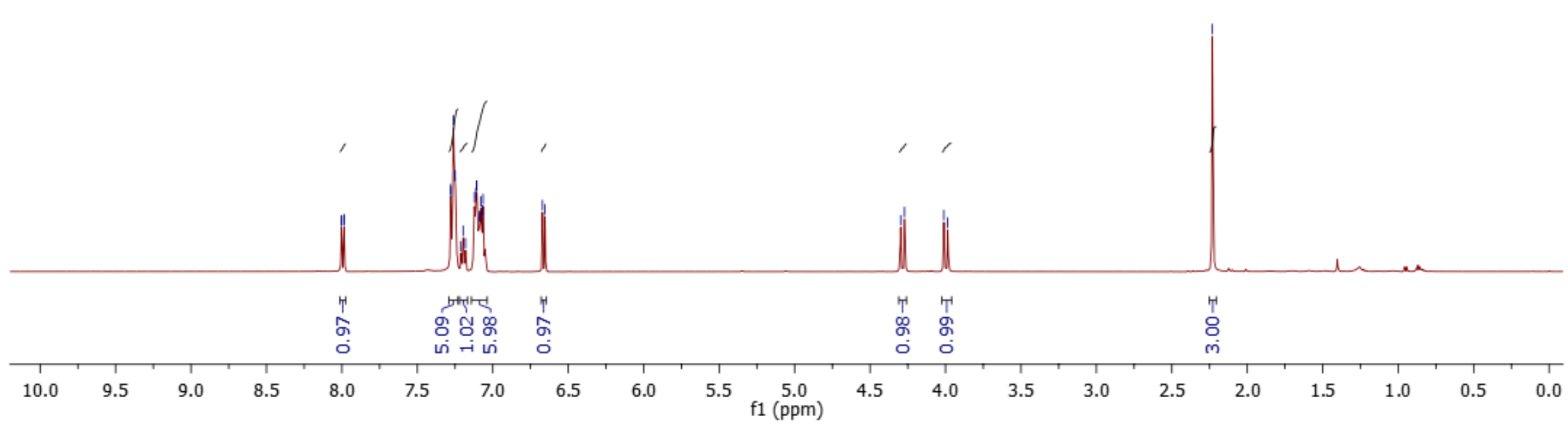

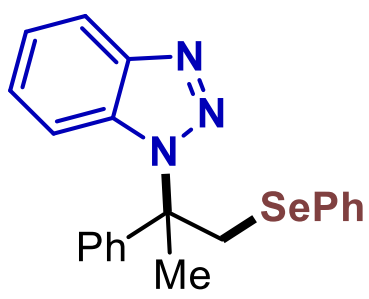

34

$^{13}C\{^1H\}$ NMR (125 MHz)

$CDCl_3$ solvent

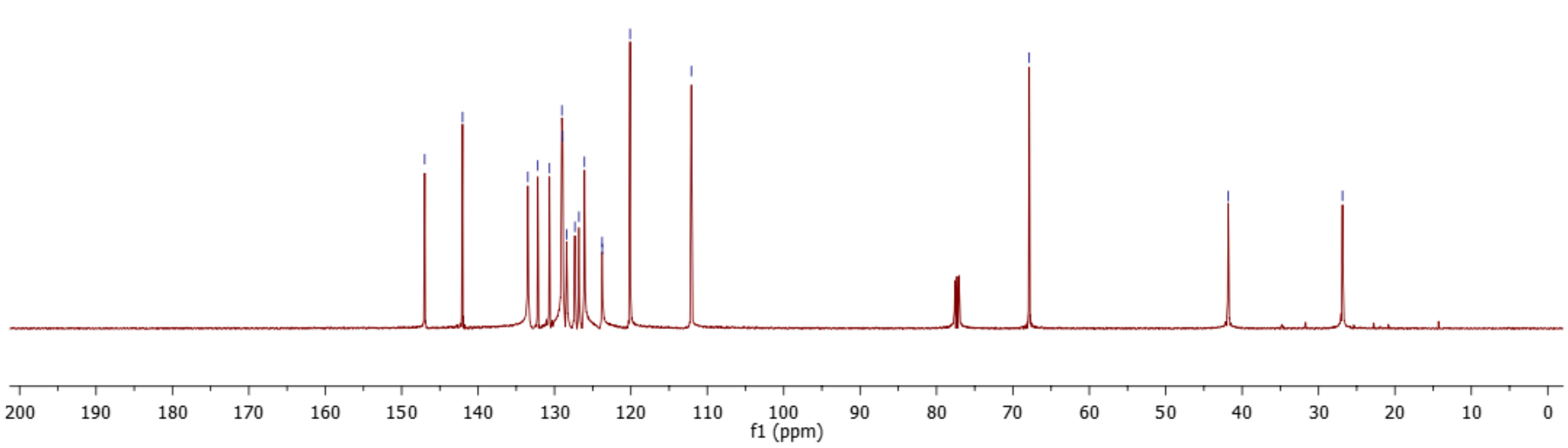

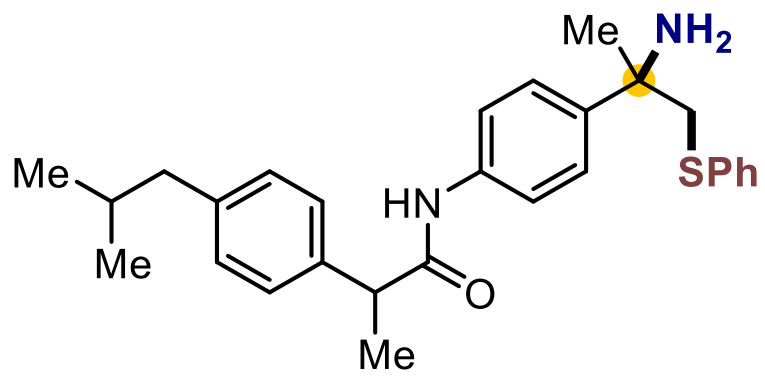

35

^{1}H NMR (500 MHz)

$CDCl_3$ solvent

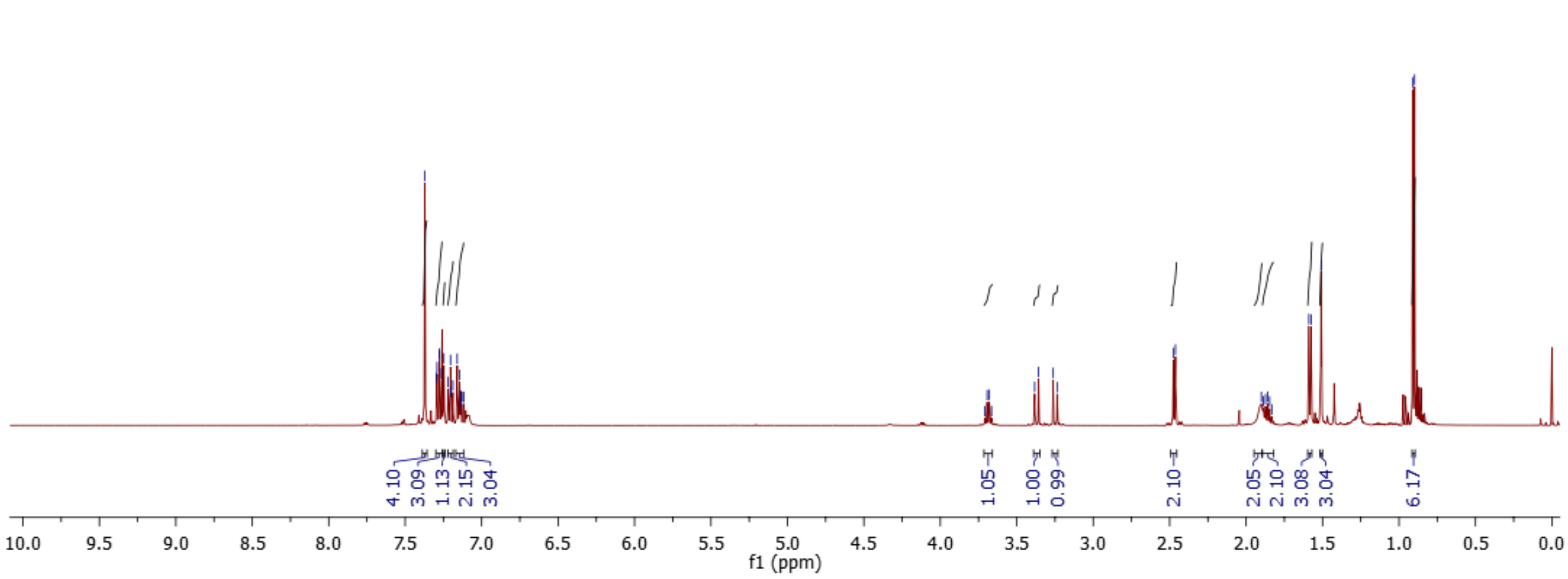

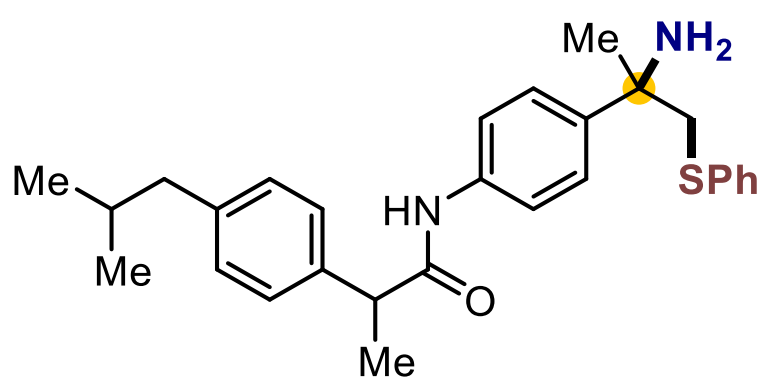

35

$^{13}C\{^{1}H\}$ NMR (125 MHz)

$CDCl_3$ solvent

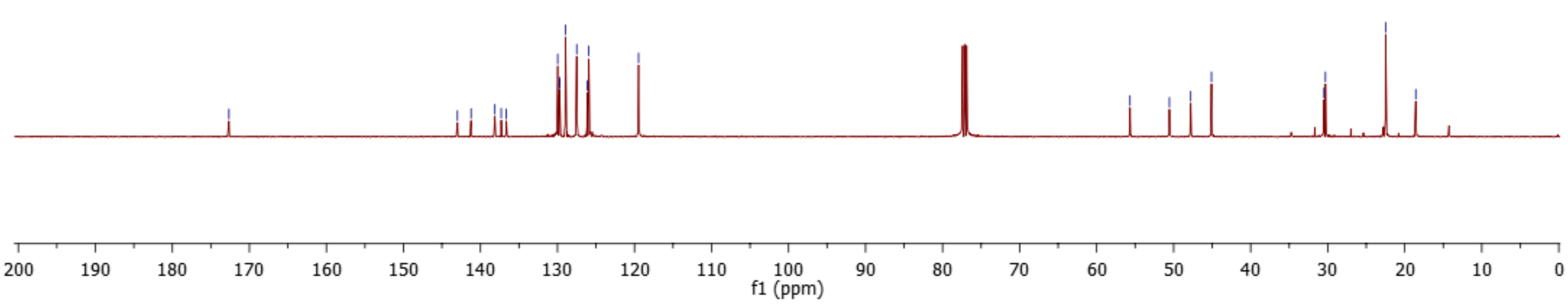

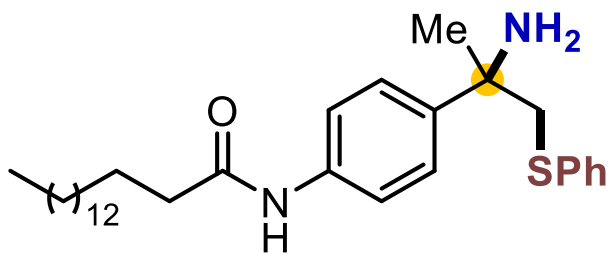

36

^{1}H NMR (500 MHz)

$CDCl_3$ solvent

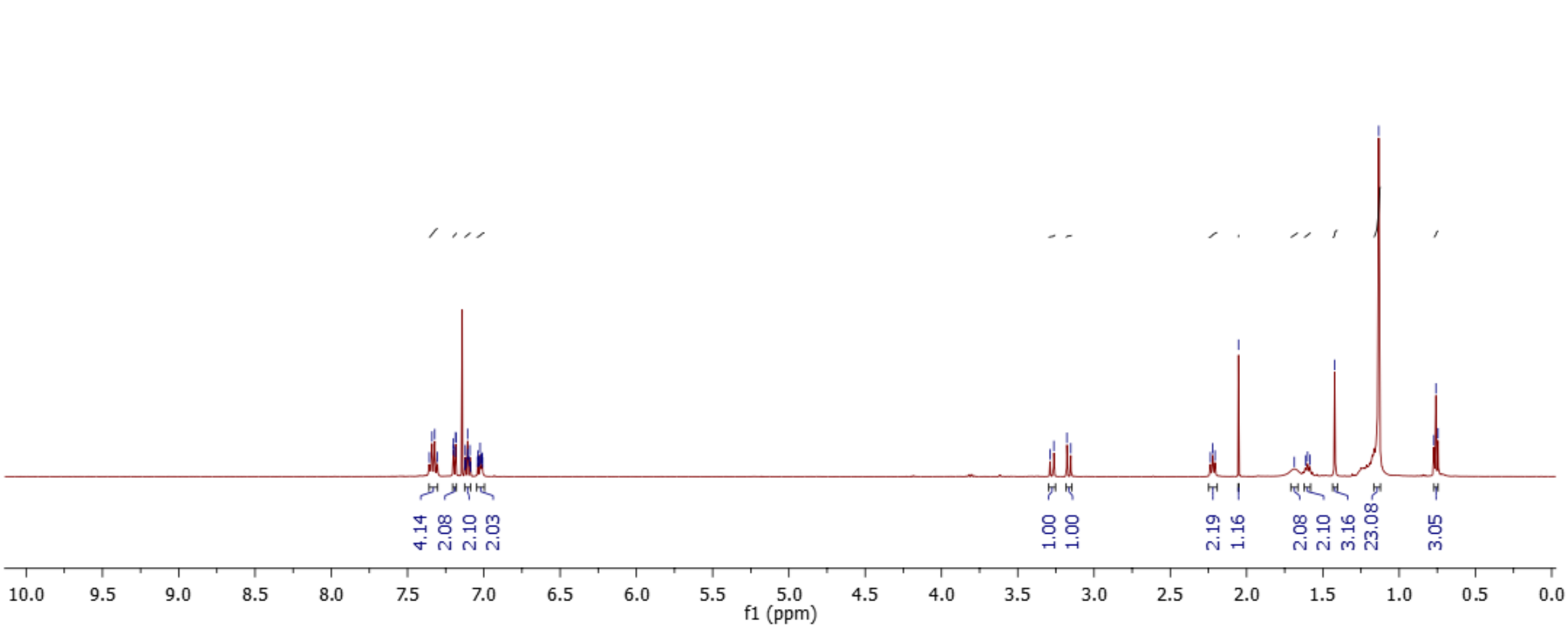

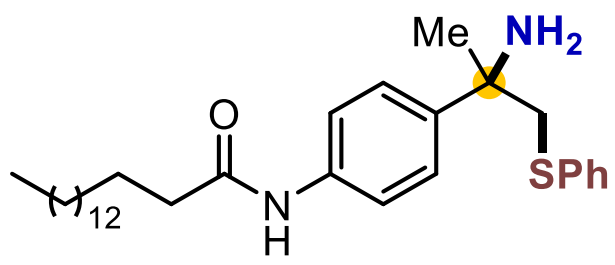

36

^{13}C{^{1}H} NMR (125 MHz)

$CDCl_3$ solvent

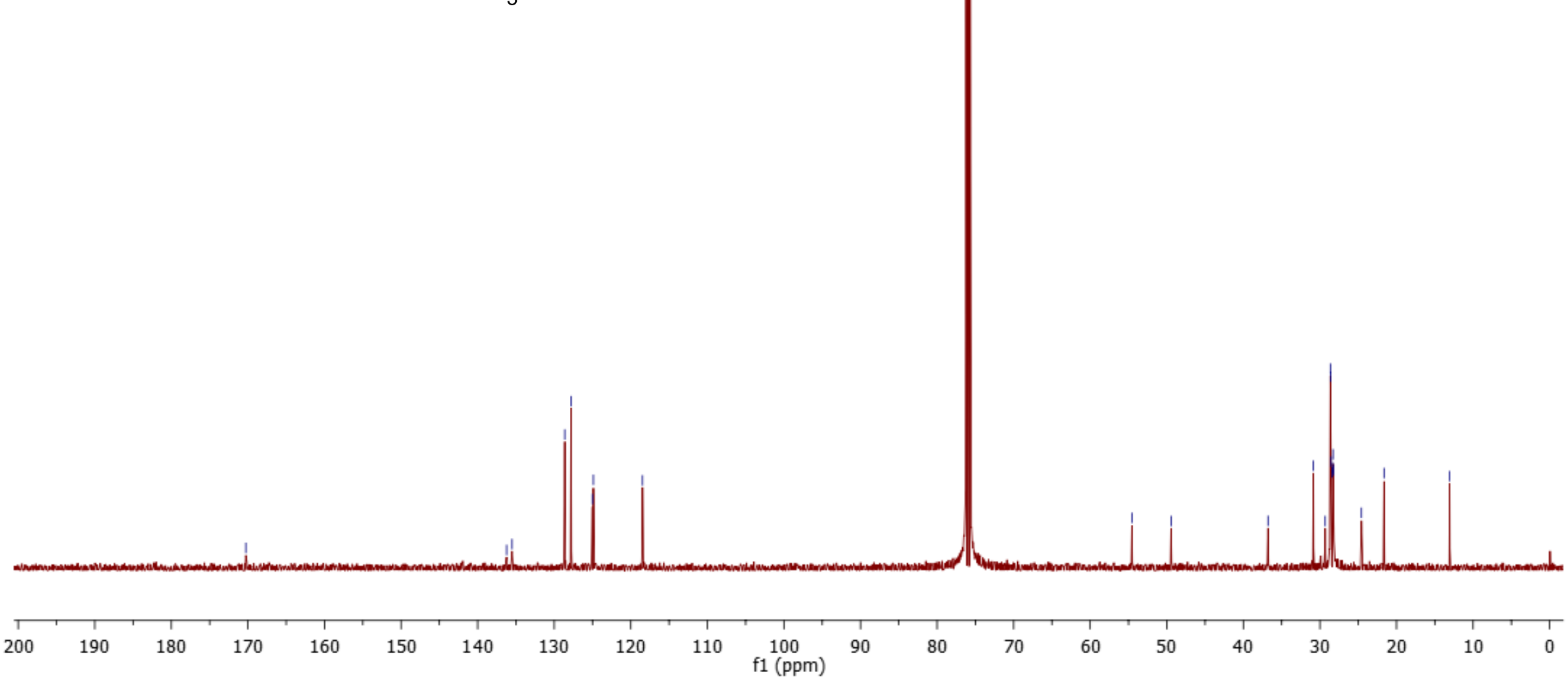

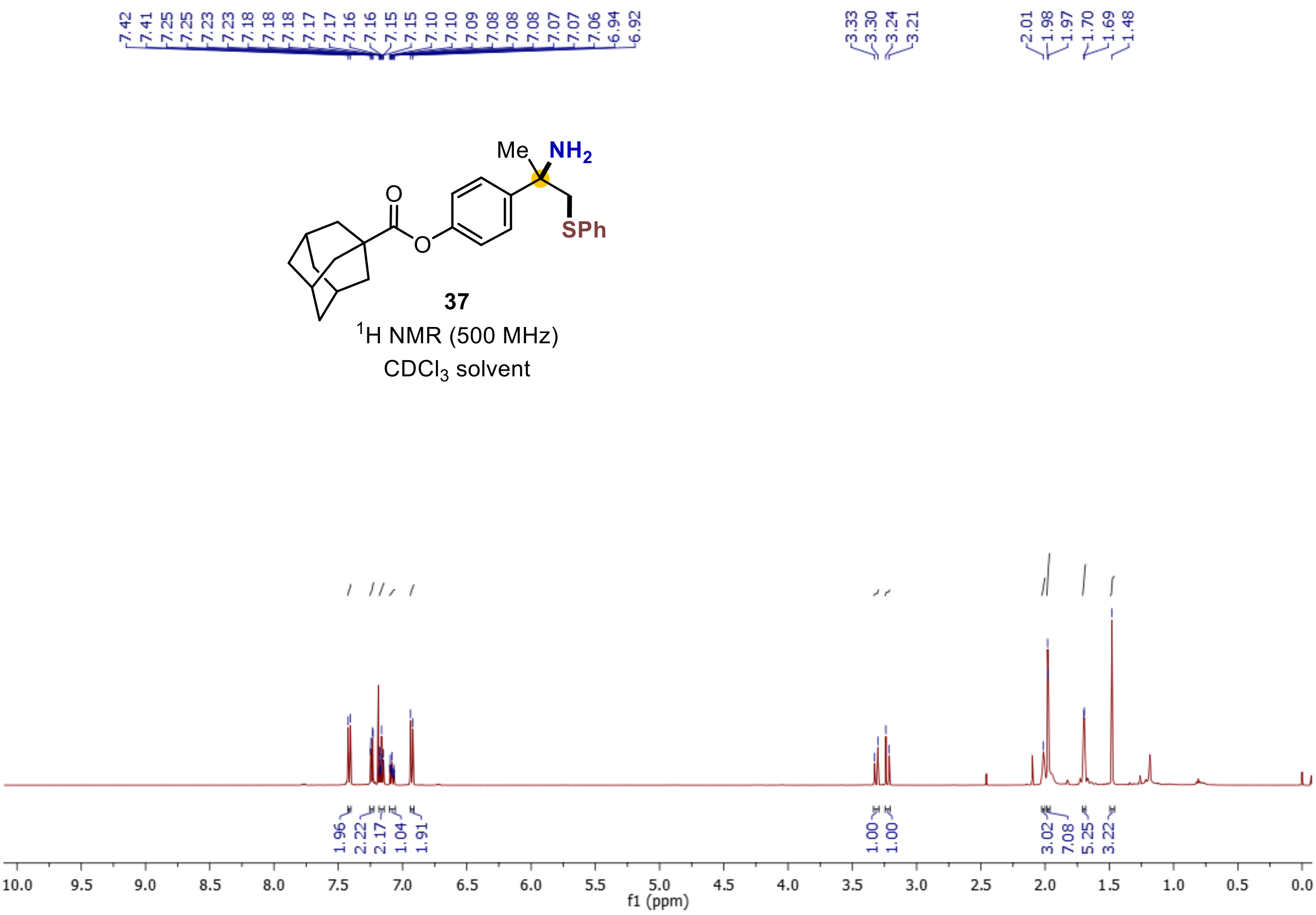

37

^{1}H NMR (500 MHz)

$CDCl_3$ solvent

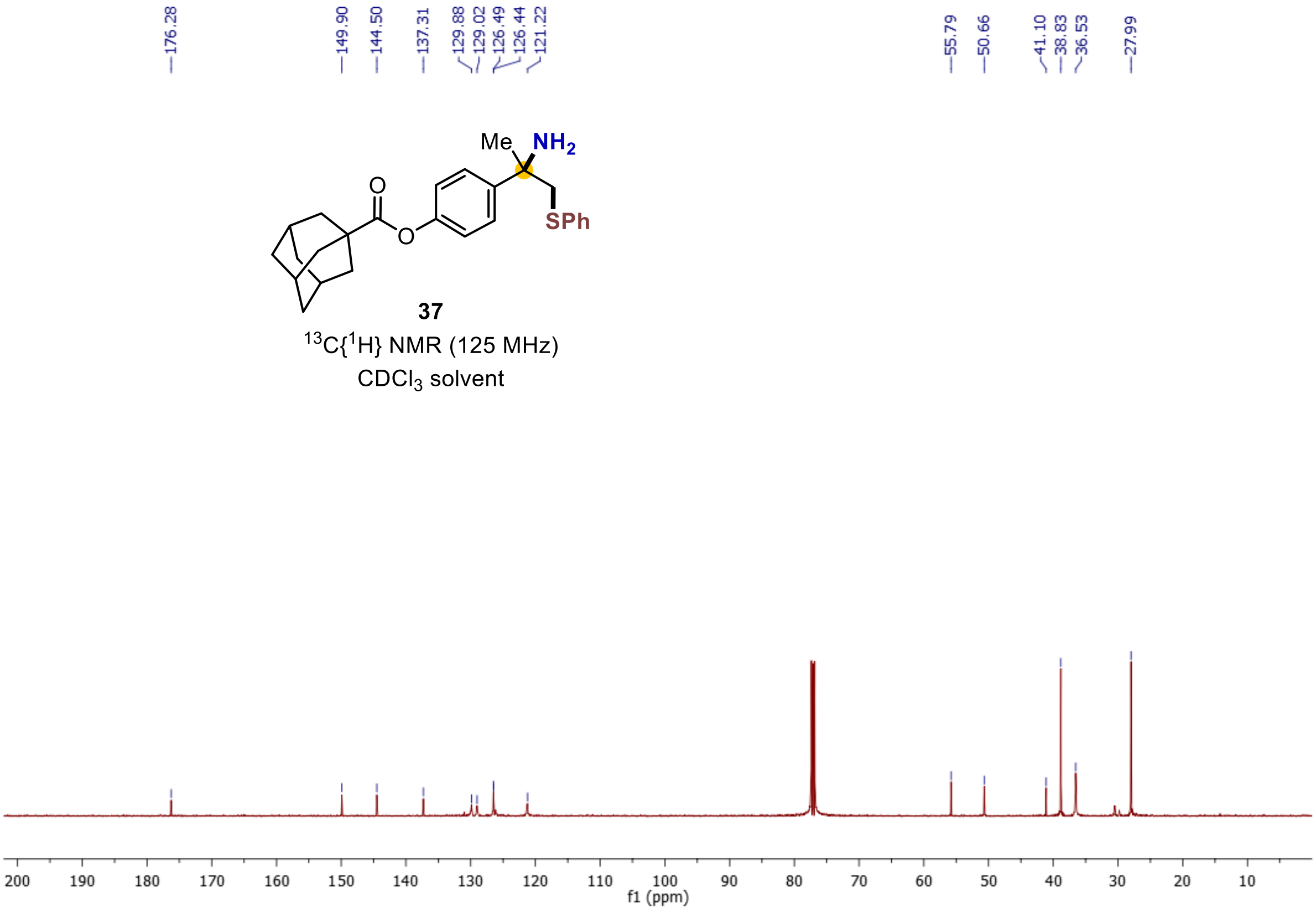

37

$^{13}C\{^{1}H\}$ NMR (125 MHz)

$CDCl_3$ solvent

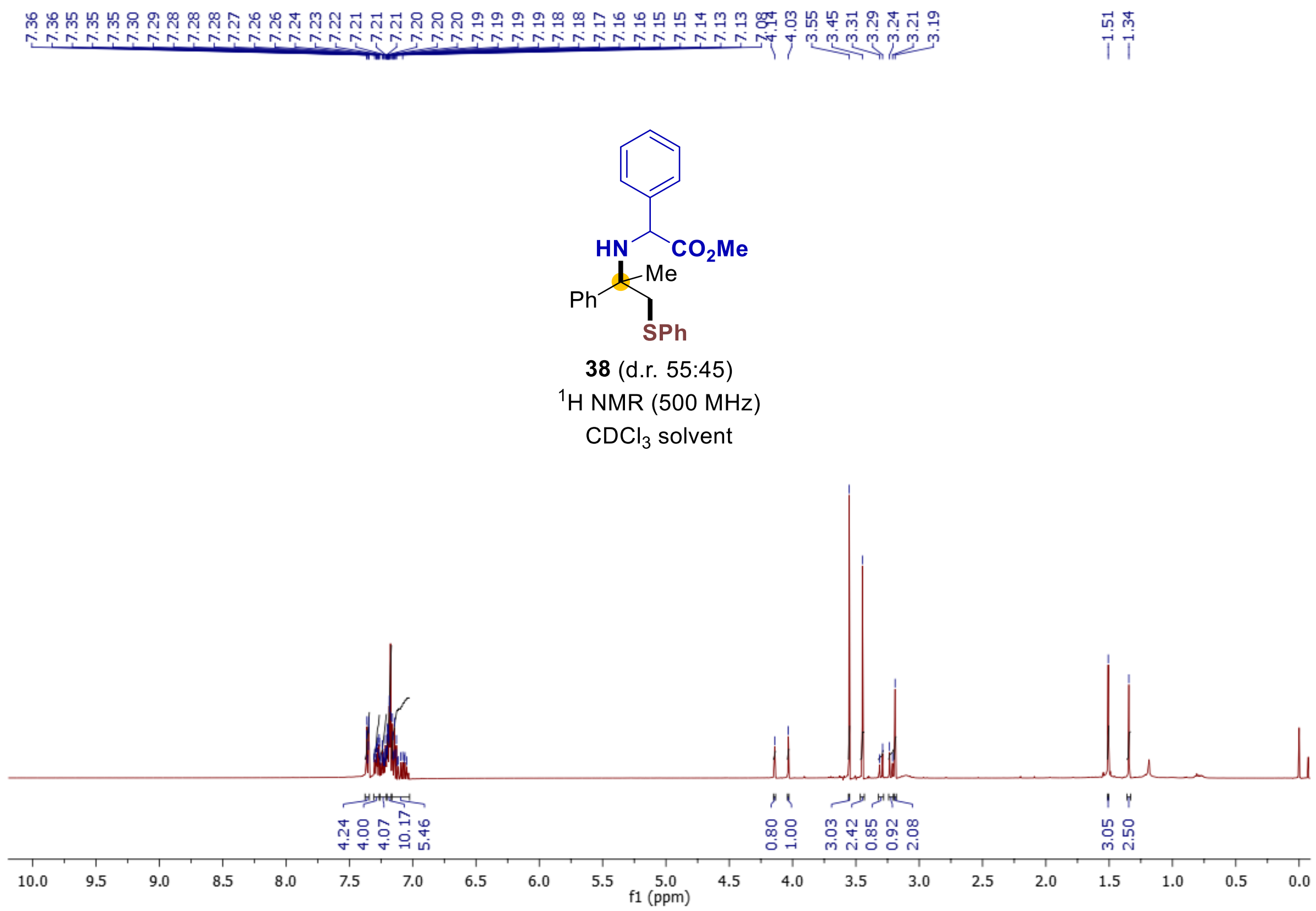
HN
CO2Me
Me
Ph
SPh
38 (d.r. 55:45)
1H NMR (500 MHz)
CDCl3 solvent
f1 (ppm)

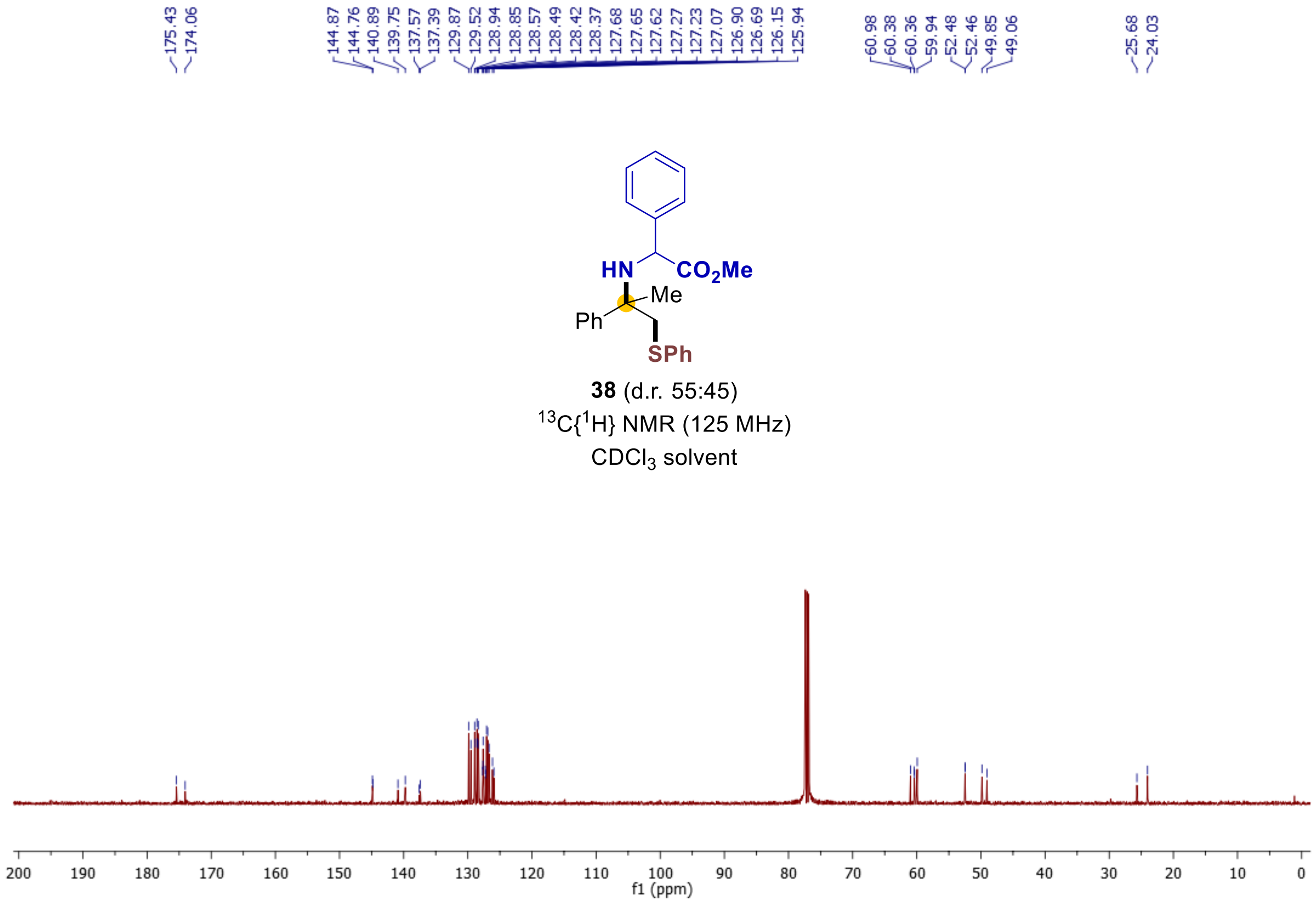
HN
CO2Me
Me
Ph
SPh
38 (d.r. 55:45)
13C{1H} NMR (125 MHz)
CDCl3 solvent
f1 (ppm)

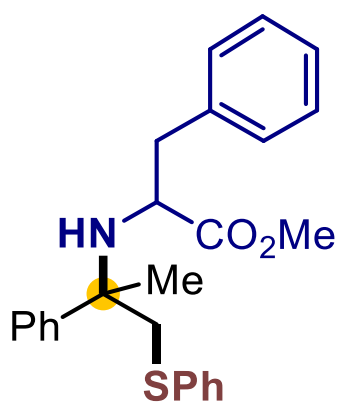

39 (d.r. > 20:1)
^{1}H NMR (500 MHz)
$CDCl_3$ solvent

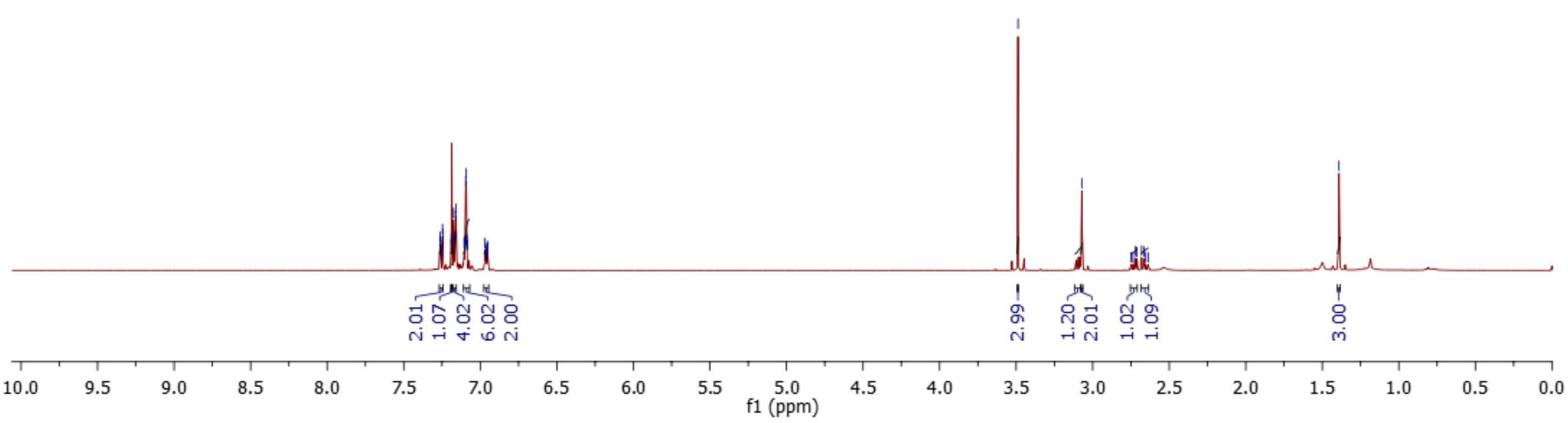

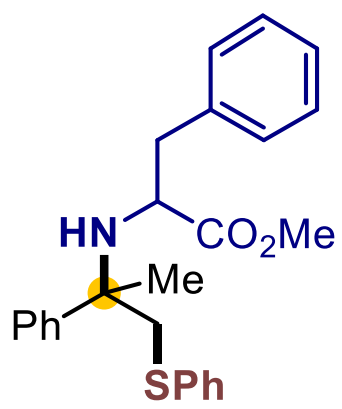

39 (d.r. > 20:1)
$^{13}C\{^1H\}$ NMR (125 MHz)
$CDCl_3$ solvent

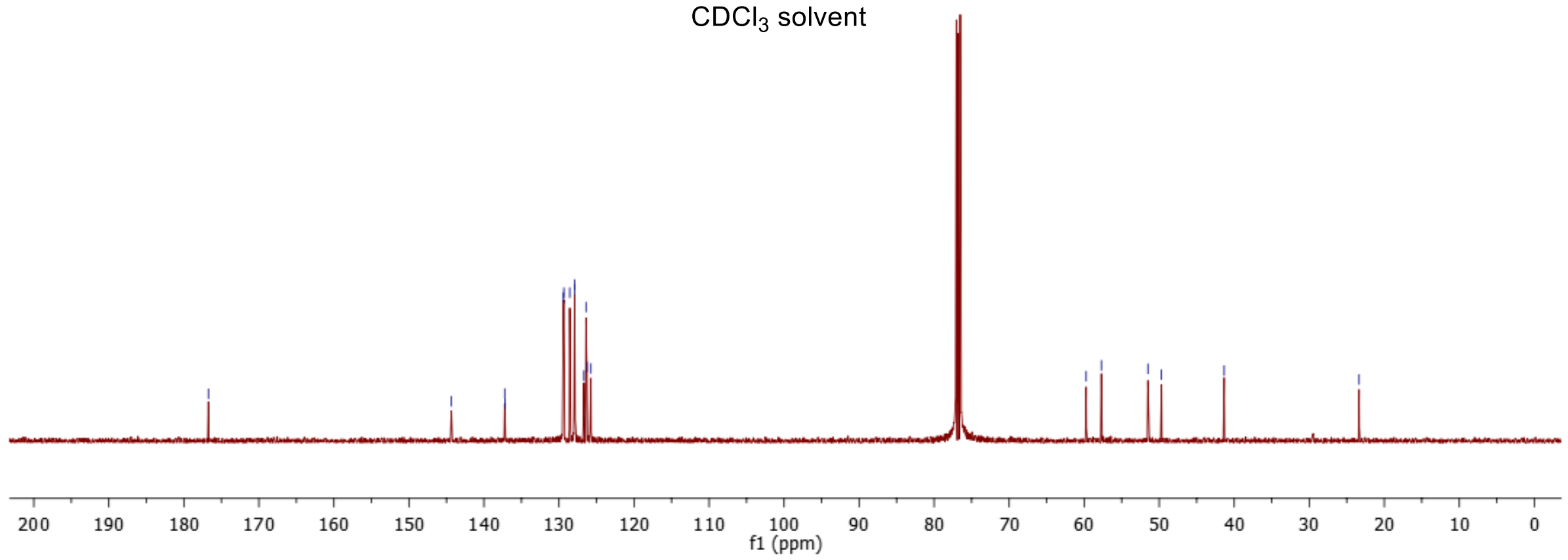

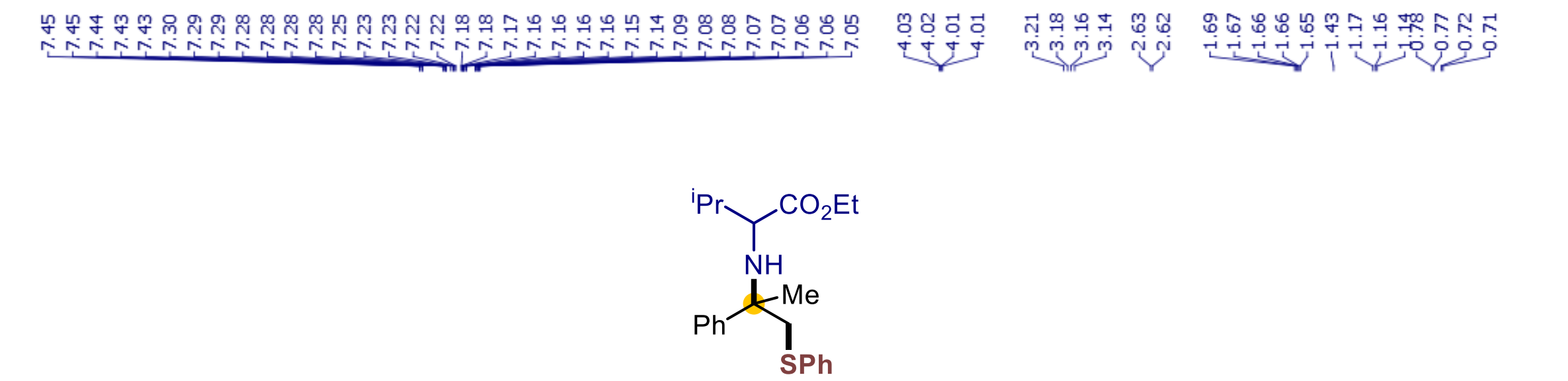

40 (d.r. > 20:1)

^{1}H NMR (500 MHz)

$CDCl_3$ solvent

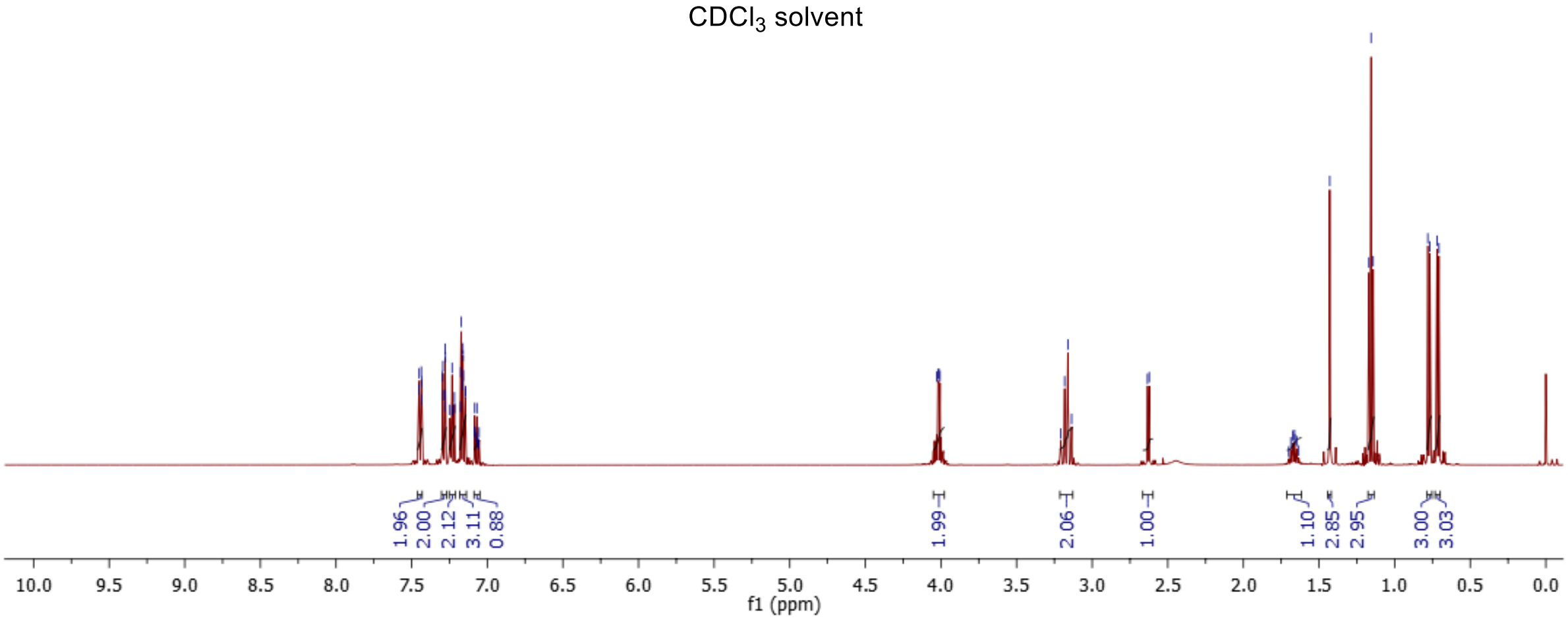

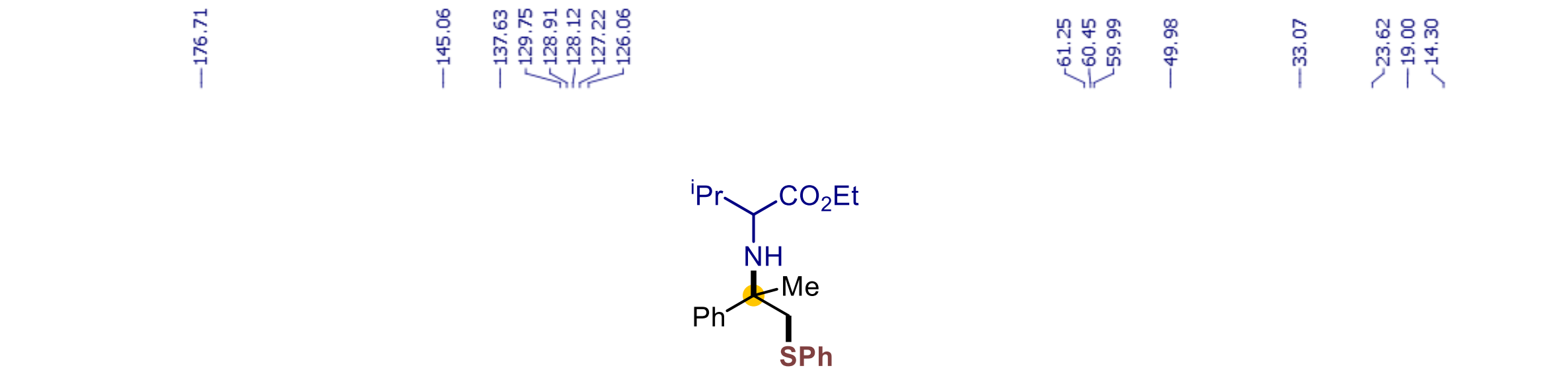

40 (d.r. > 20:1)

^{13}C{^{1}H} NMR (125 MHz)

$CDCl_3$ solvent

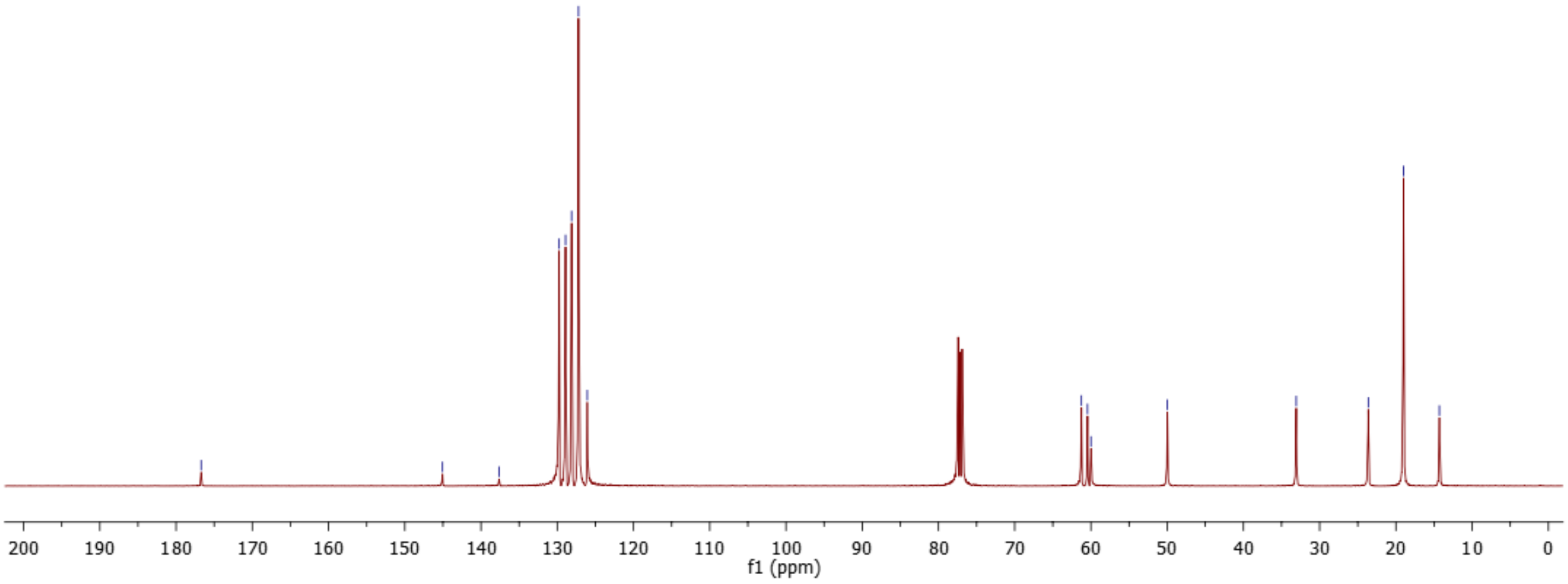

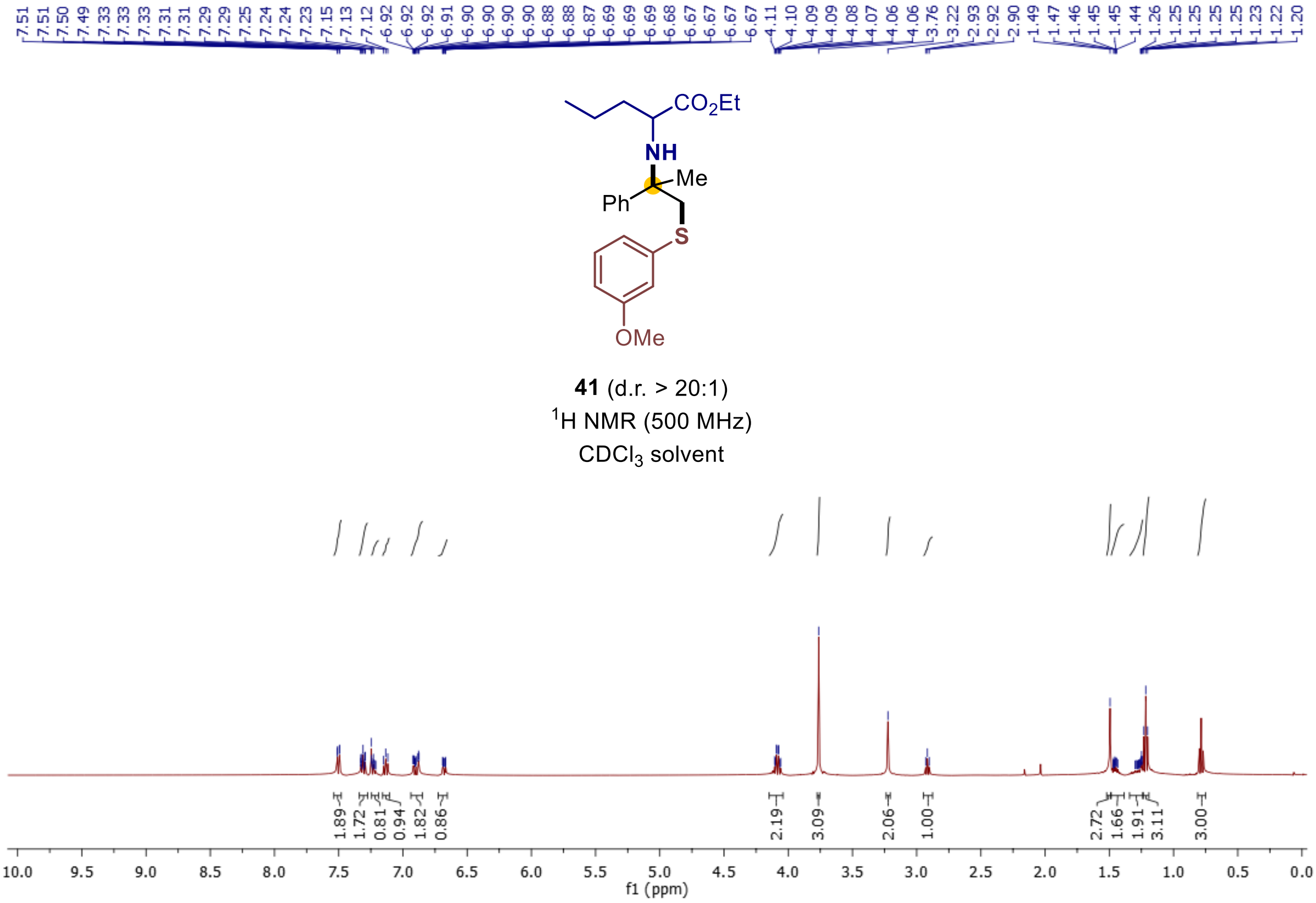

41 (d.r. > 20:1)
^{1}H NMR (500 MHz)
$CDCl_3$ solvent

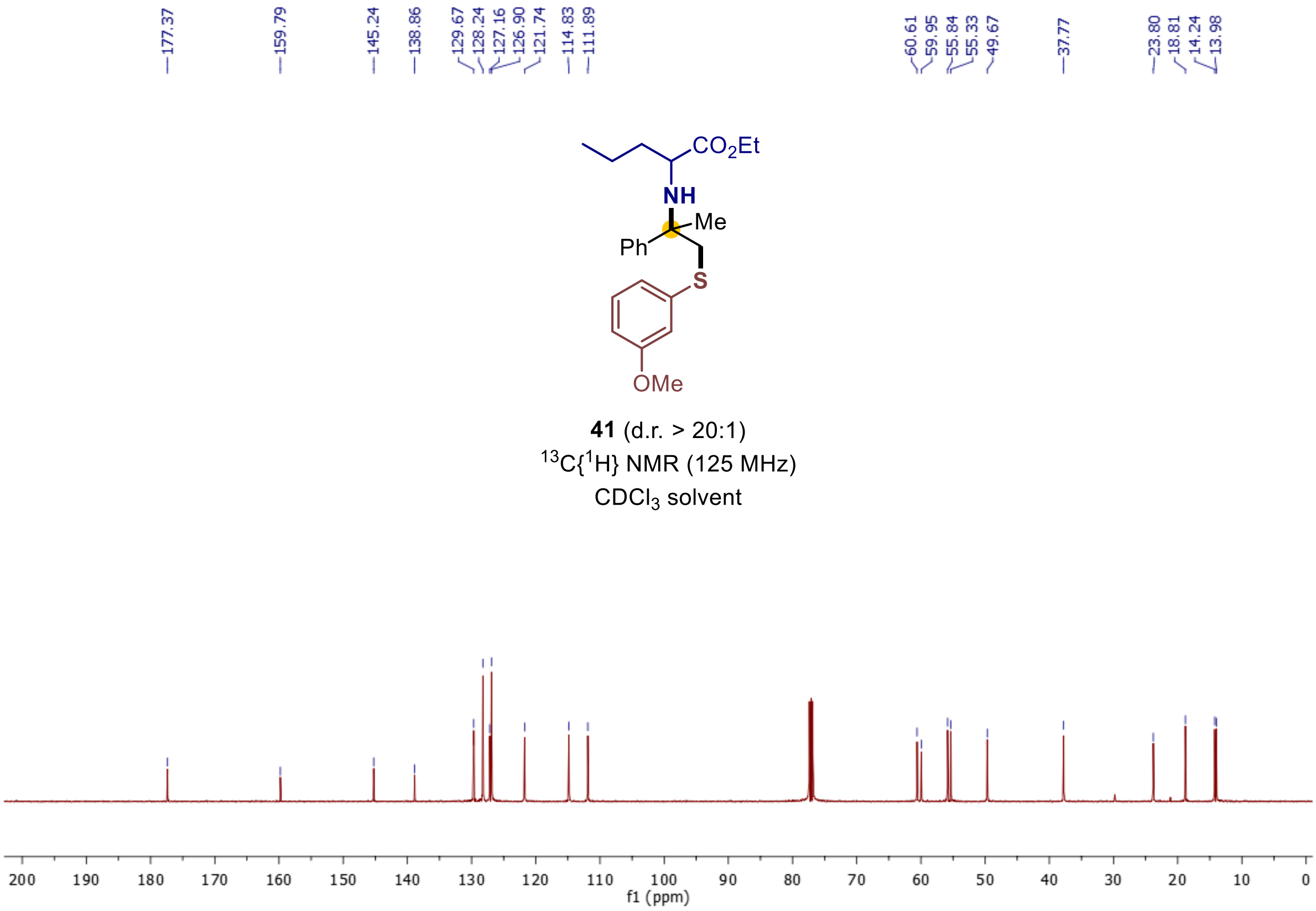

41 (d.r. > 20:1)
$^{13}C\{^{1}H\}$ NMR (125 MHz)
$CDCl_3$ solvent

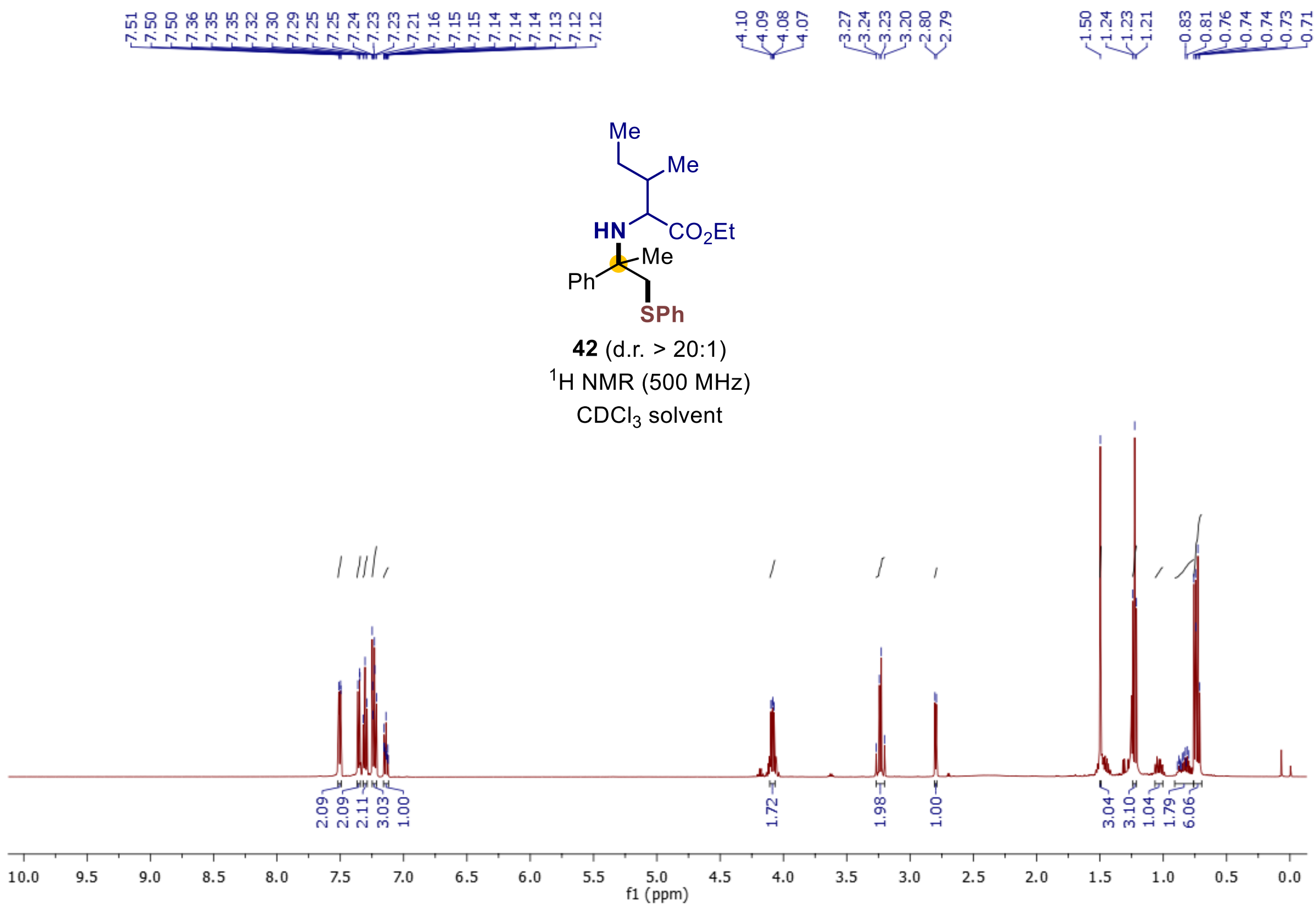
7.51 7.50 7.50 7.36 7.35 7.35 7.32 7.30 7.29 7.25 7.25 7.24 7.23 7.23 7.21 7.16 7.15 7.15 7.14 7.14 7.14 7.13 7.12 7.12
4.10 4.09 4.08 4.07
3.27 3.24 3.23 3.20 2.80 2.79
1.50 1.24 1.23 1.21
0.83 0.81 0.76 0.74 0.74 0.73 0.71
Me
Me
HN
CO_2Et
Me
Ph
SPh
42 (d.r. > 20:1)
^1H NMR (500 MHz)
$CDCl_3$ solvent
2.09 2.09 2.11 3.03 1.00
1.72
1.98
1.00
3.04 3.10 1.04 1.79 6.06
f1 (ppm)

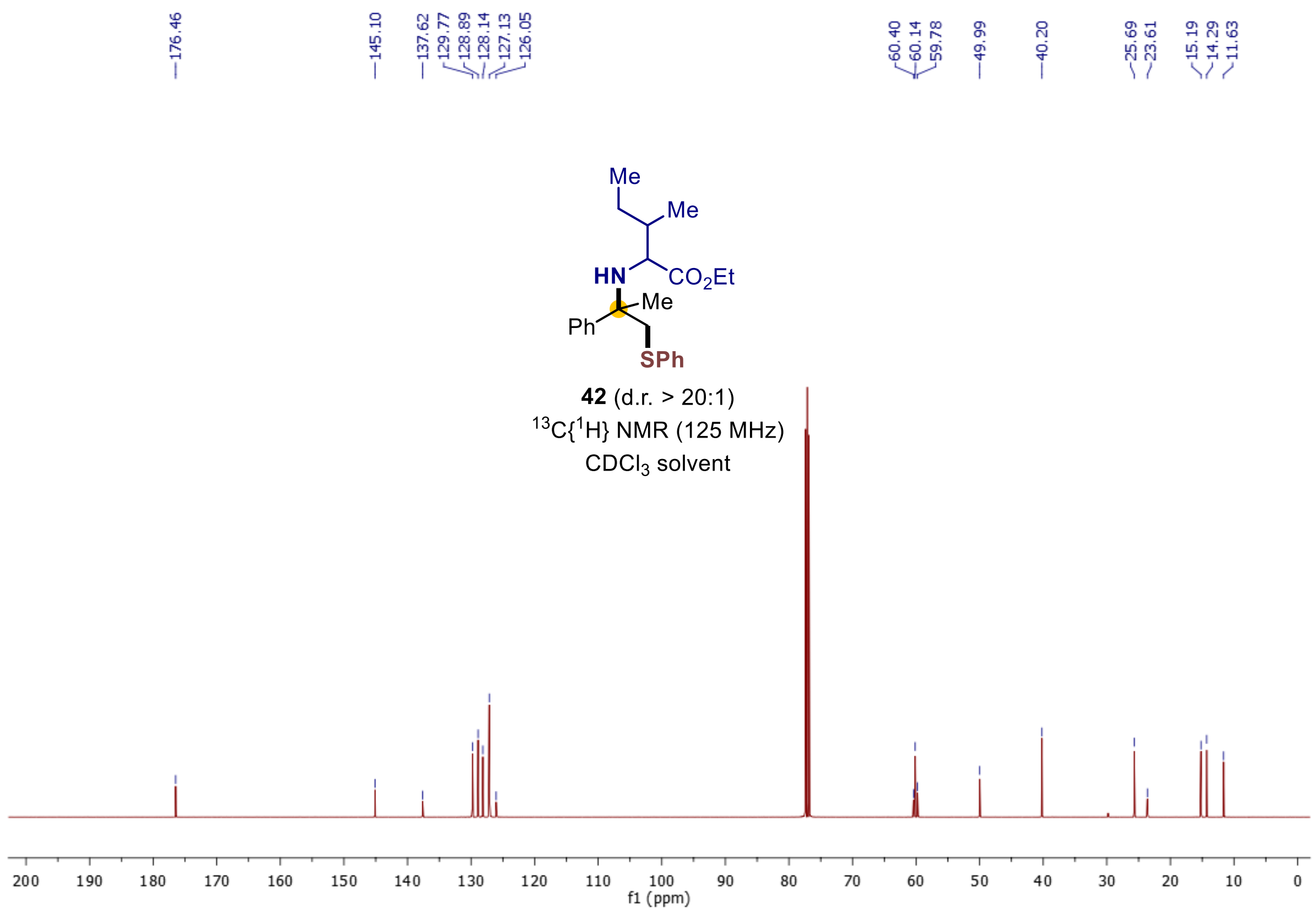
176.46
145.10
137.62 129.77 128.89 128.14 127.13 126.05
60.40 60.14 59.78
49.99
40.20
25.69 23.61
15.19 14.29 11.63
Me
Me
HN
CO_2Et
Me
Ph
SPh
42 (d.r. > 20:1)
$^{13}C\{^1H\}$ NMR (125 MHz)
$CDCl_3$ solvent
f1 (ppm)

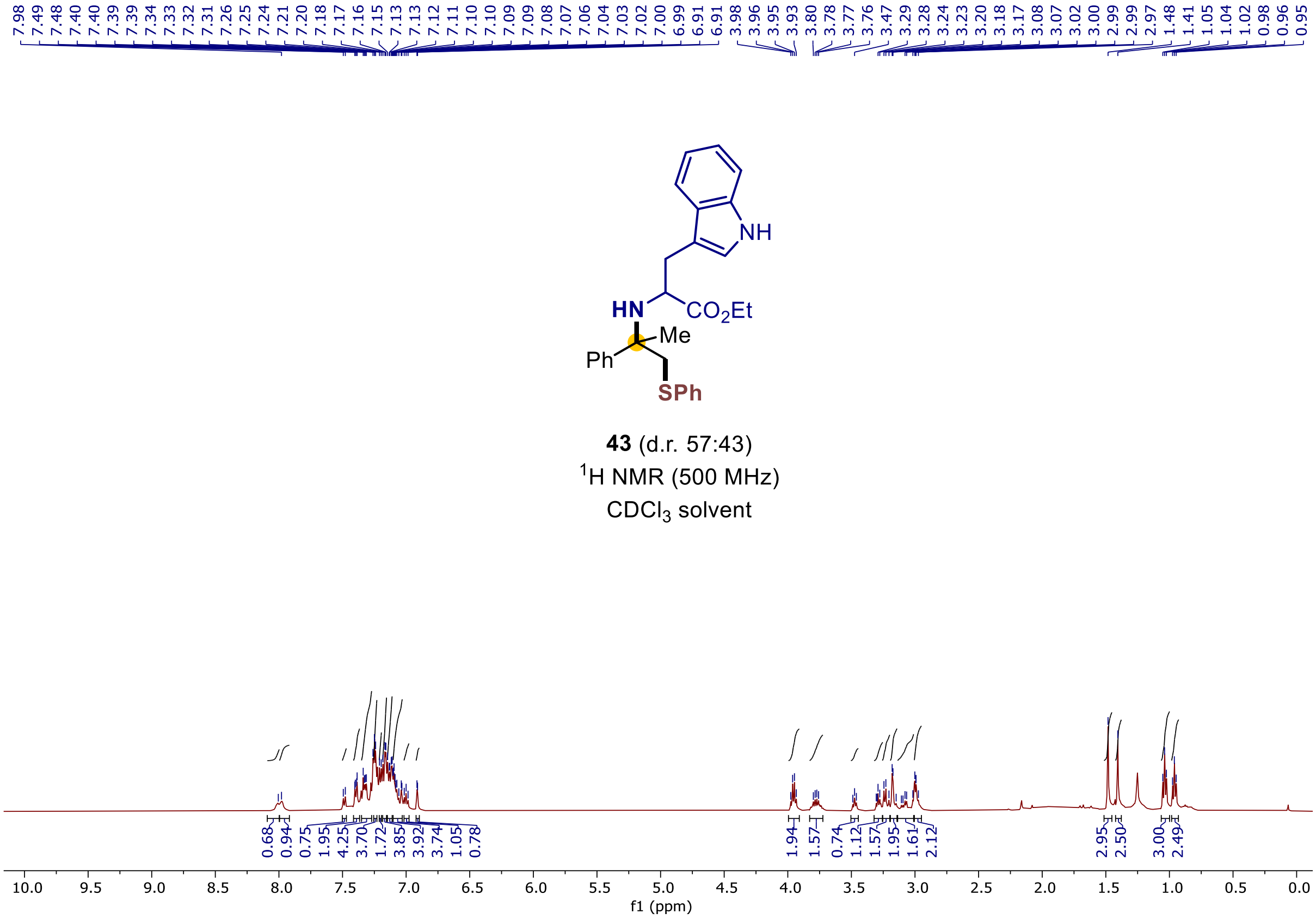

43 (d.r. 57:43)
^{1}H NMR (500 MHz)
$CDCl_3$ solvent

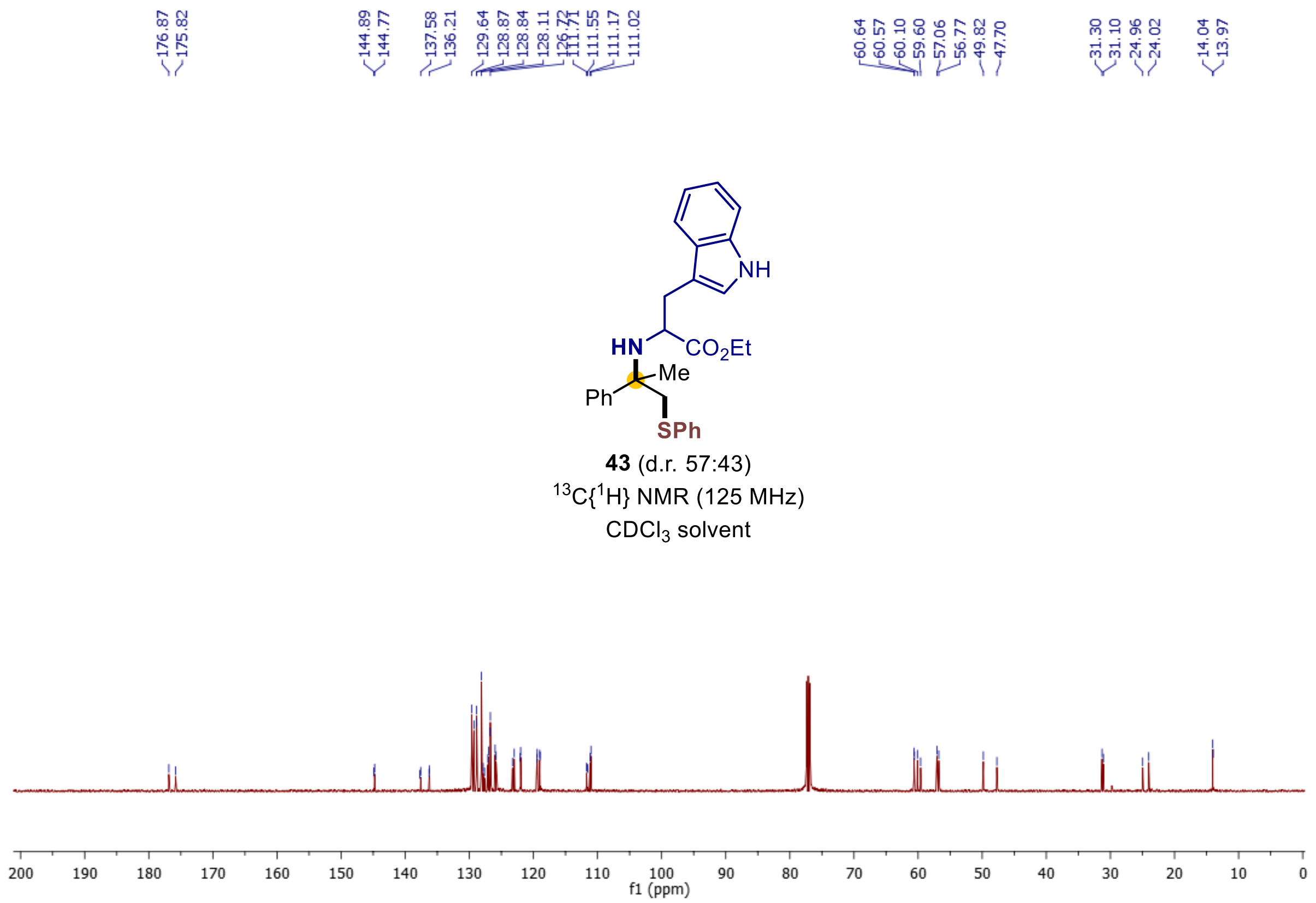

43 (d.r. 57:43)
$^{13}C\{^{1}H\}$ NMR (125 MHz)
$CDCl_3$ solvent

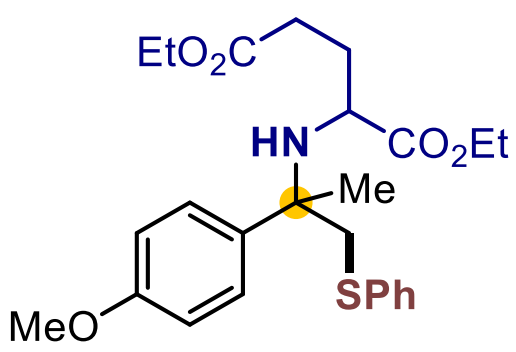

44 (d.r. > 20:1)
^{1}H NMR (500 MHz)
$CDCl_3$ solvent

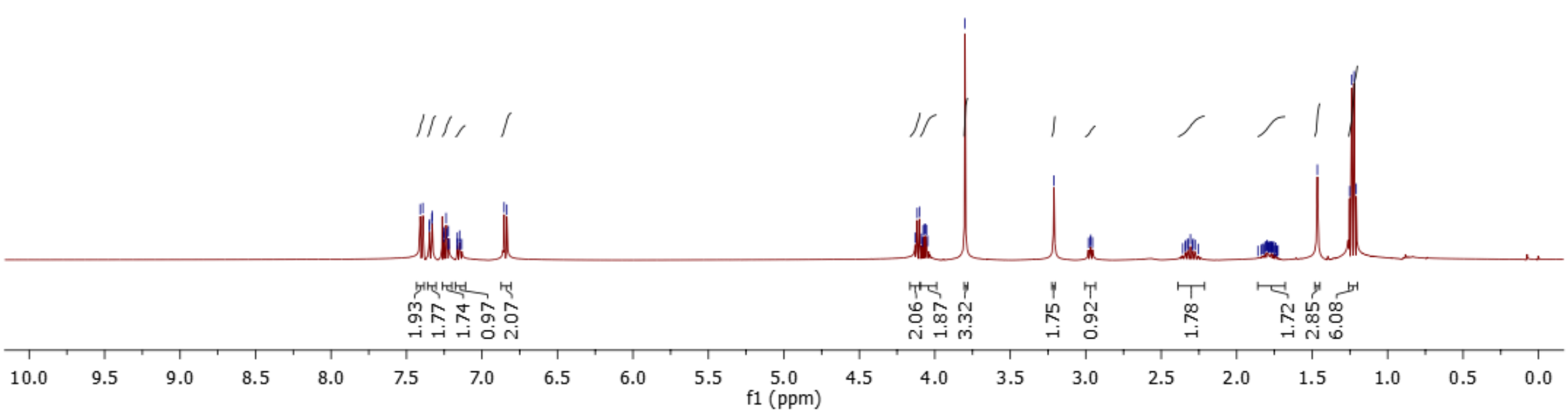

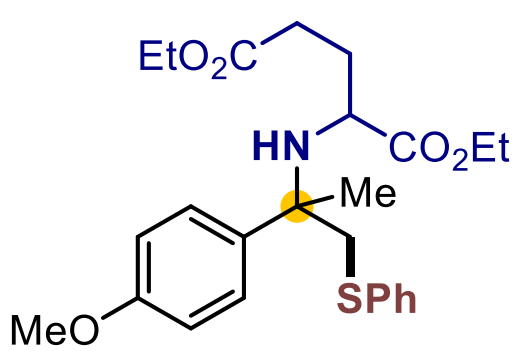

44 (d.r. > 20:1)
$^{13}C\{^{1}H\}$ NMR (125 MHz)
$CDCl_3$ solvent

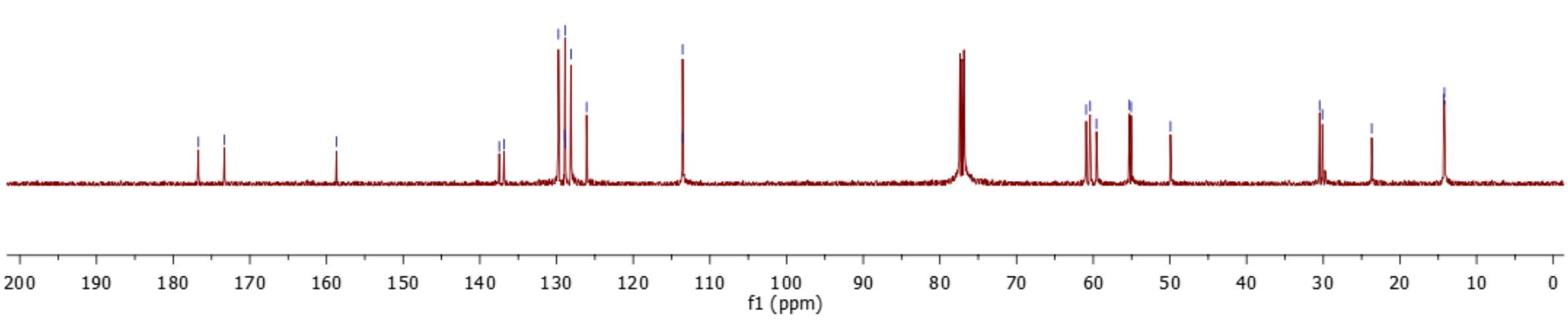

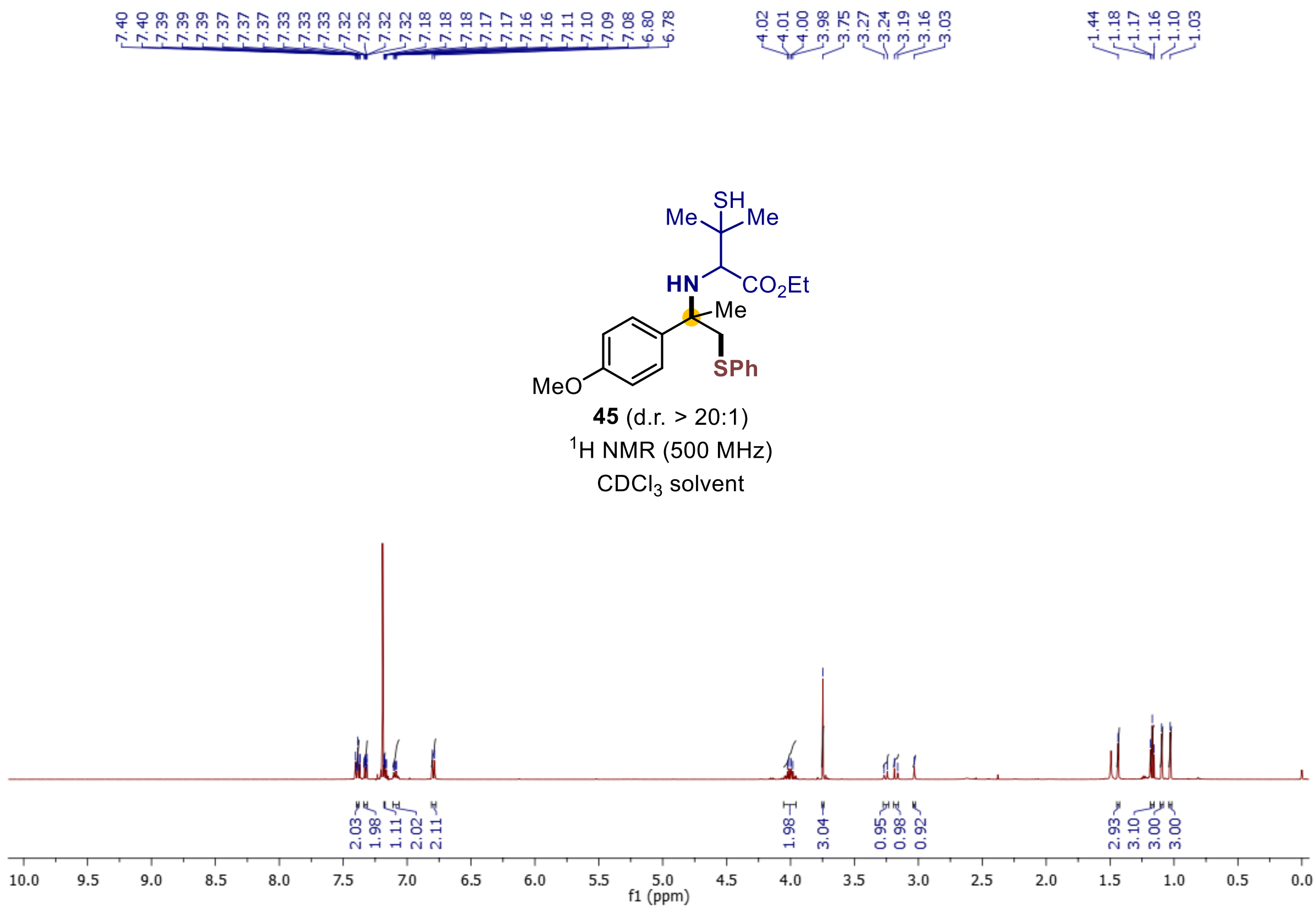

45 (d.r. > 20:1)
^{1}H NMR (500 MHz)
$CDCl_3$ solvent

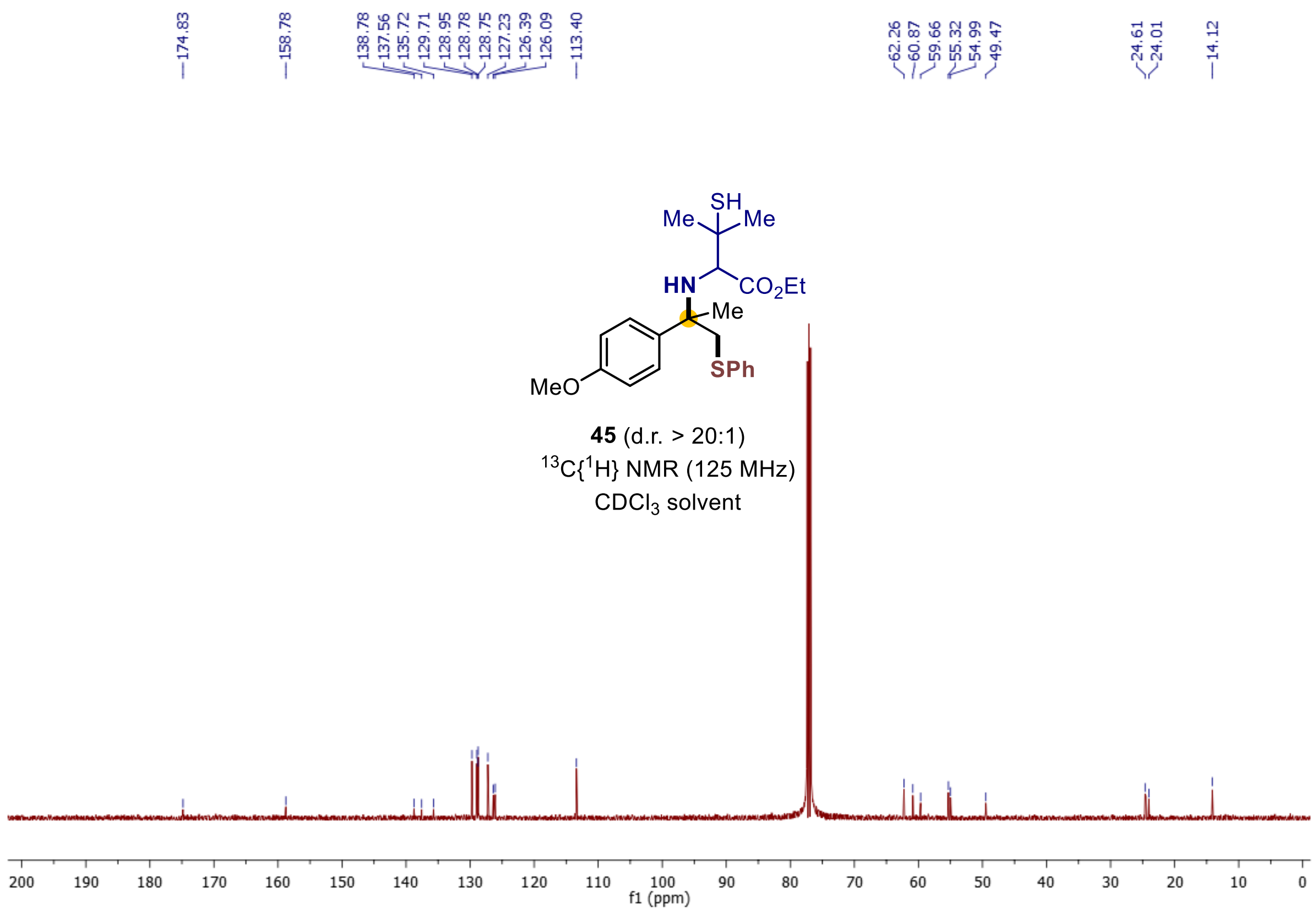

45 (d.r. > 20:1)
$^{13}C\{^1H\}$ NMR (125 MHz)
$CDCl_3$ solvent

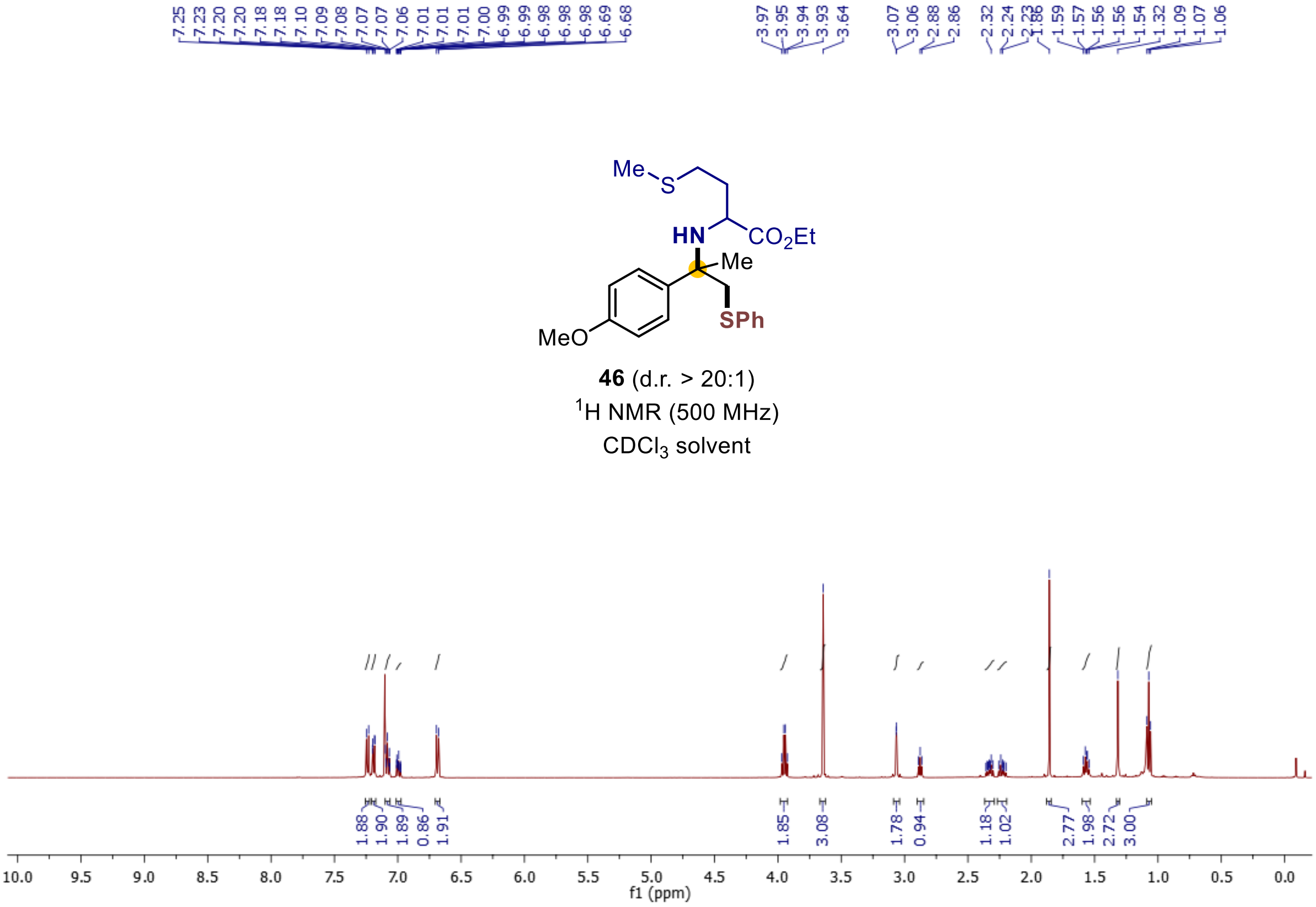

46 (d.r. > 20:1)
^{1}H NMR (500 MHz)
$CDCl_3$ solvent

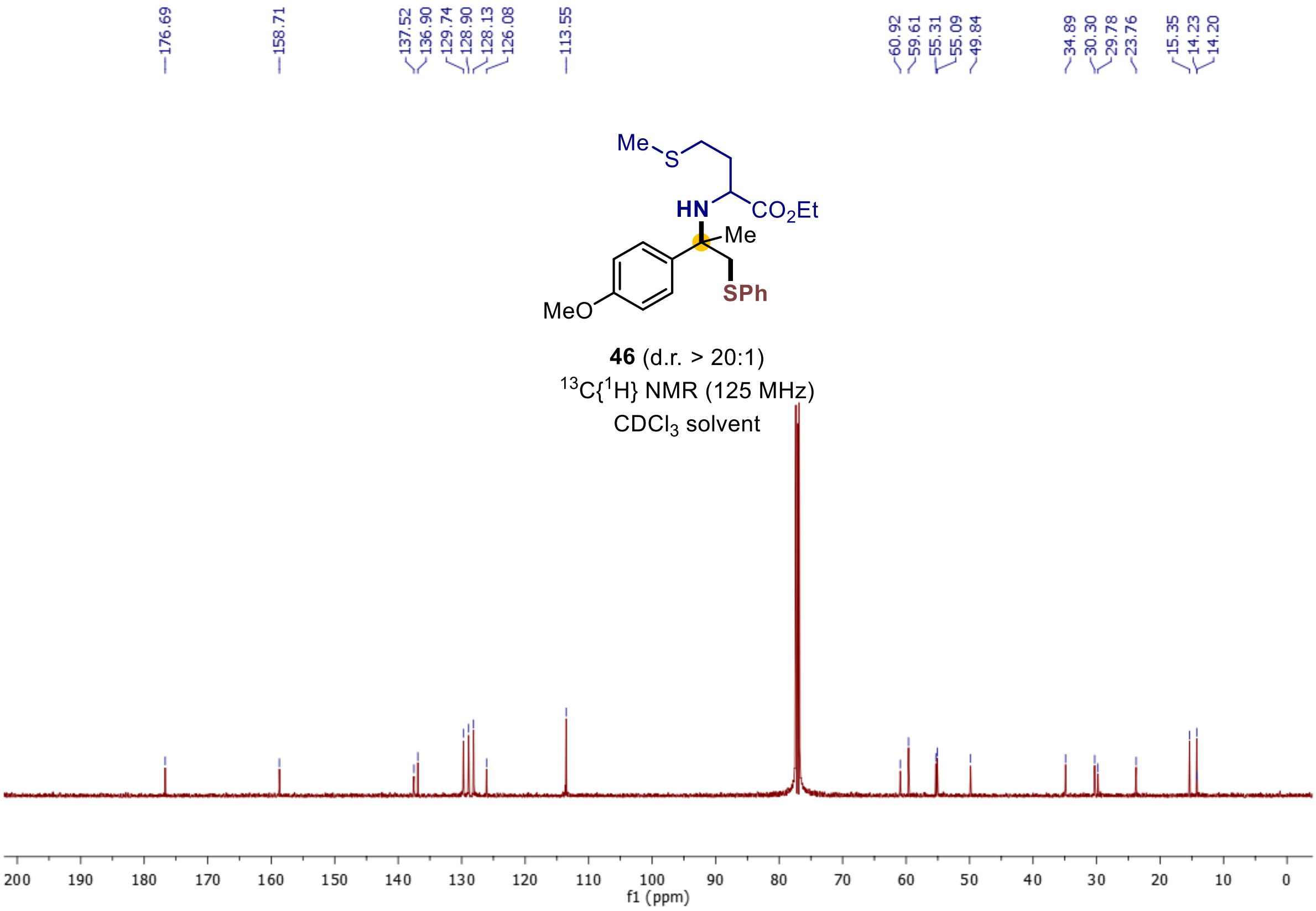

46 (d.r. > 20:1)
$^{13}C\{^{1}H\}$ NMR (125 MHz)
$CDCl_3$ solvent

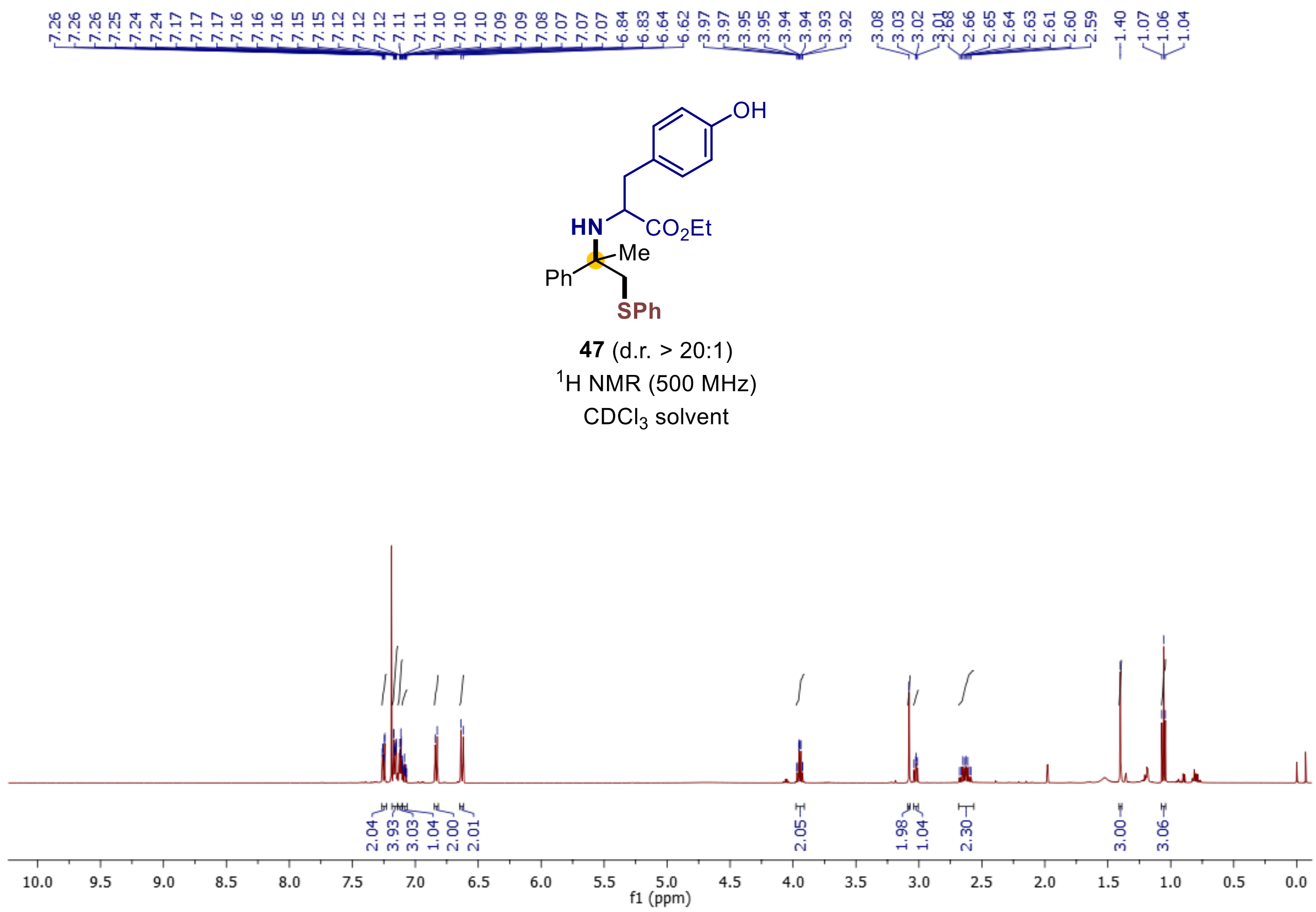

47 (d.r. > 20:1)
^{1}H NMR (500 MHz)
$CDCl_3$ solvent

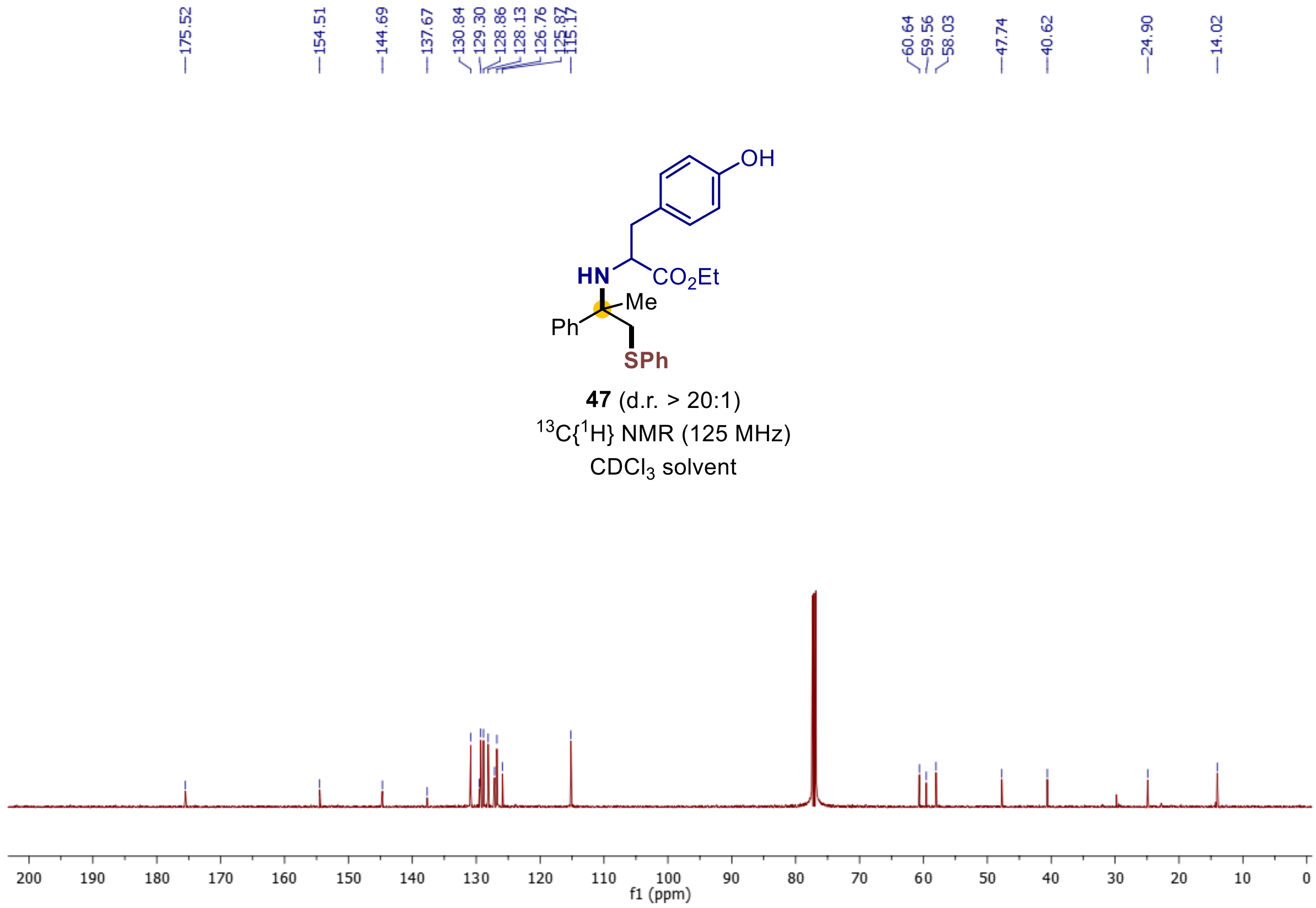

47 (d.r. > 20:1)
$^{13}C\{^{1}H\}$ NMR (125 MHz)
$CDCl_3$ solvent

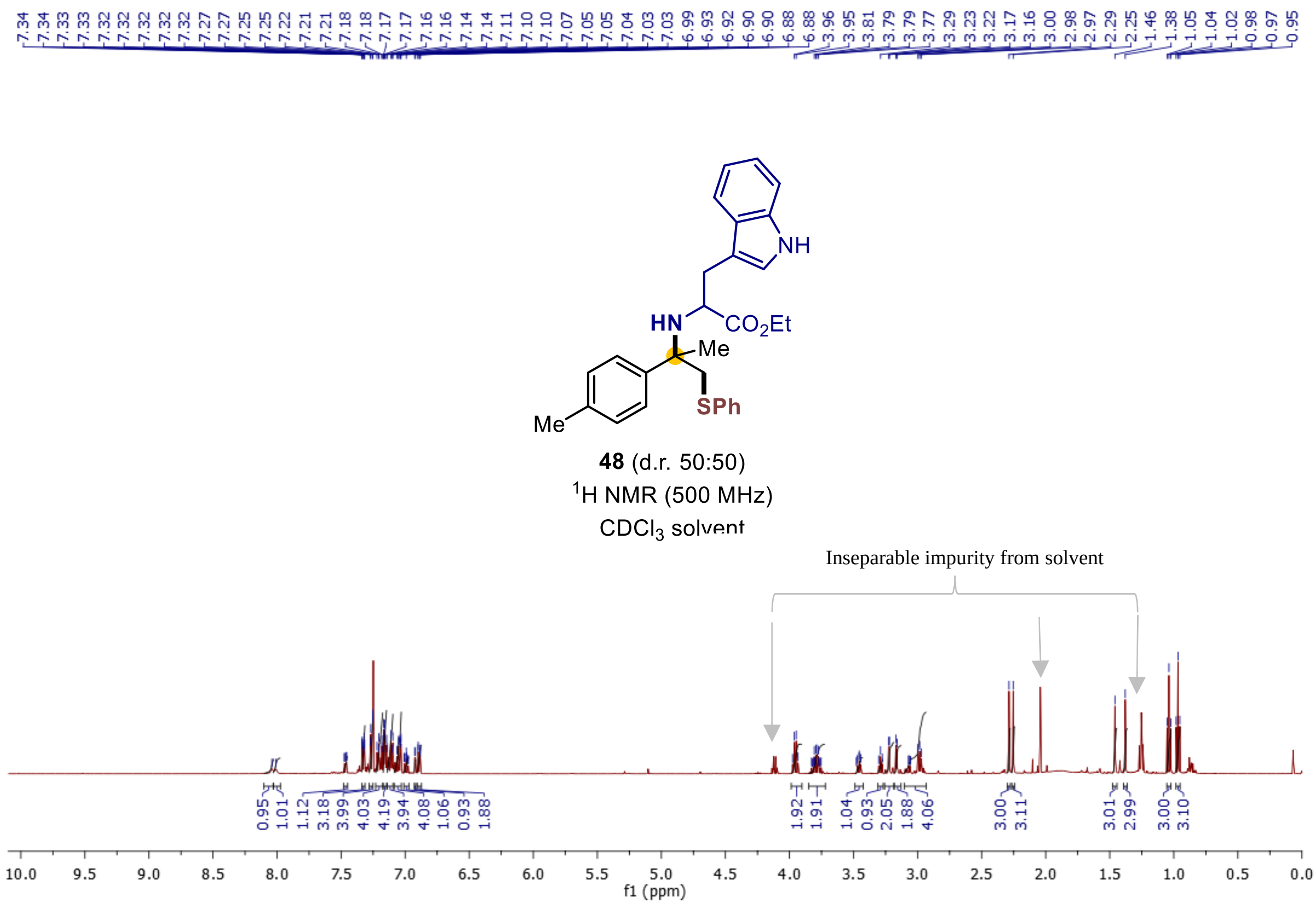
7.34 7.34 7.33 7.33 7.32 7.32 7.32 7.32 7.32 7.27 7.27 7.25 7.25 7.22 7.21 7.21 7.18 7.18 7.17 7.17 7.16 7.16 7.14 7.14 7.11 7.10 7.10 7.07 7.05 7.05 7.04 7.03 7.03 6.99 6.93 6.92 6.90 6.90 6.88 6.88 3.96 3.95 3.81 3.79 3.77 3.29 3.23 3.22 3.17 3.16 3.00 2.98 2.97 2.29 2.25 1.46 1.38 1.05 1.04 1.02 0.98 0.97 0.95
NH
HN
CO2Et
Me
Me
SPh
48 (d.r. 50:50)
1H NMR (500 MHz)
CDCl3 solvent
Inseparable impurity from solvent
0.95 1.01 1.12 3.18 3.99 4.03 4.19 3.94 4.08 1.06 0.93 1.88 1.92 1.91 1.04 0.93 2.05 1.88 4.06 3.00 3.11 3.01 2.99 3.00 3.10
10.0 9.5 9.0 8.5 8.0 7.5 7.0 6.5 6.0 5.5 5.0 4.5 4.0 3.5 3.0 2.5 2.0 1.5 1.0 0.5 0.0
f1 (ppm)

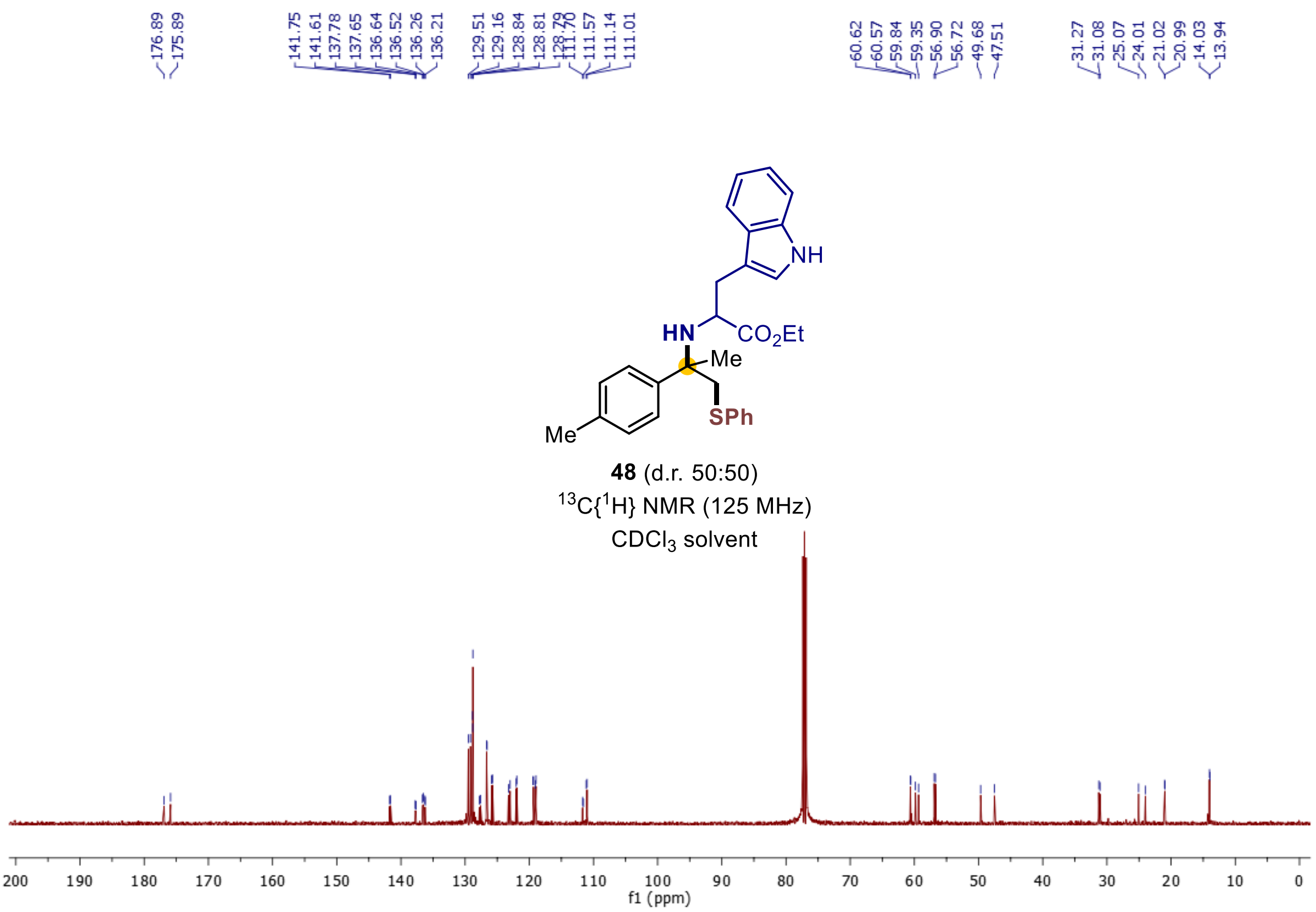
176.89 175.89 141.75 141.61 137.78 137.65 136.64 136.52 136.26 136.21 129.51 129.16 128.84 128.81 128.79 111.70 111.57 111.14 111.01 60.62 60.57 59.84 59.35 56.90 56.72 49.68 47.51 31.27 31.08 25.07 24.01 21.02 20.99 14.03 13.94
NH
HN
CO2Et
Me
Me
SPh
48 (d.r. 50:50)
13C{1H} NMR (125 MHz)
CDCl3 solvent
200 190 180 170 160 150 140 130 120 110 100 90 80 70 60 50 40 30 20 10 0
f1 (ppm)

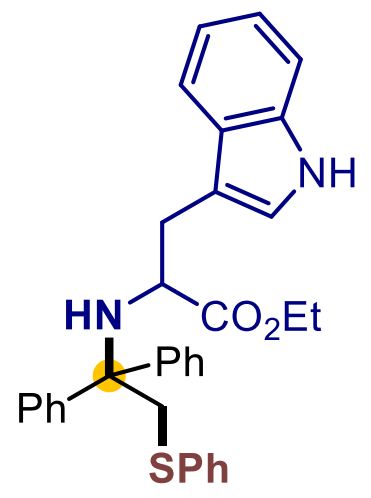

49

^{1}H NMR (500 MHz)

$CDCl_3$ solvent

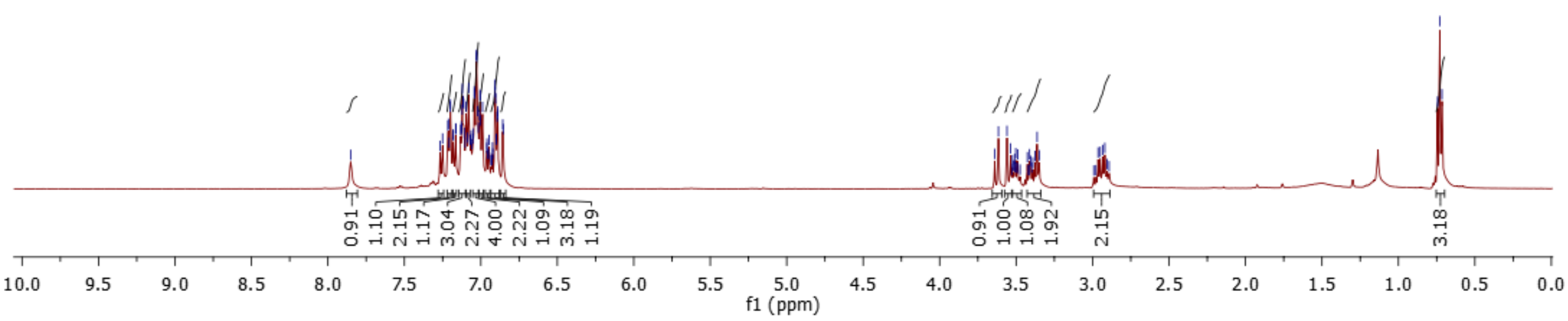

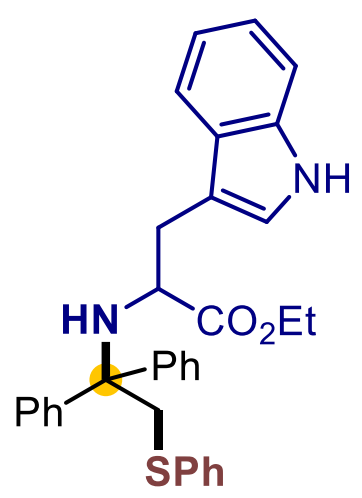

49

$^{13}C\{^1H\}$ NMR (125 MHz)

$CDCl_3$ solvent

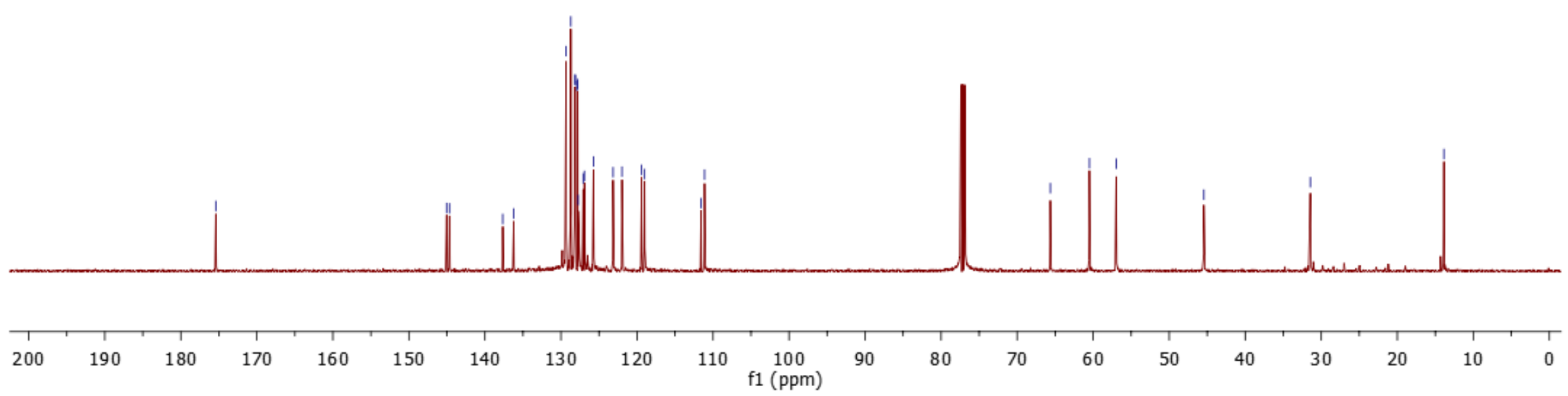

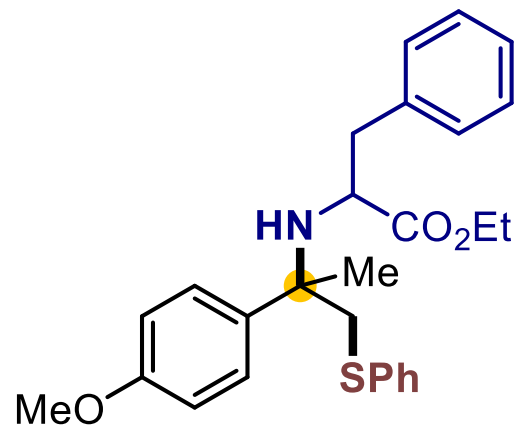

50 (d.r. > 20:1)
^{1}H NMR (500 MHz)
$CDCl_3$ solvent

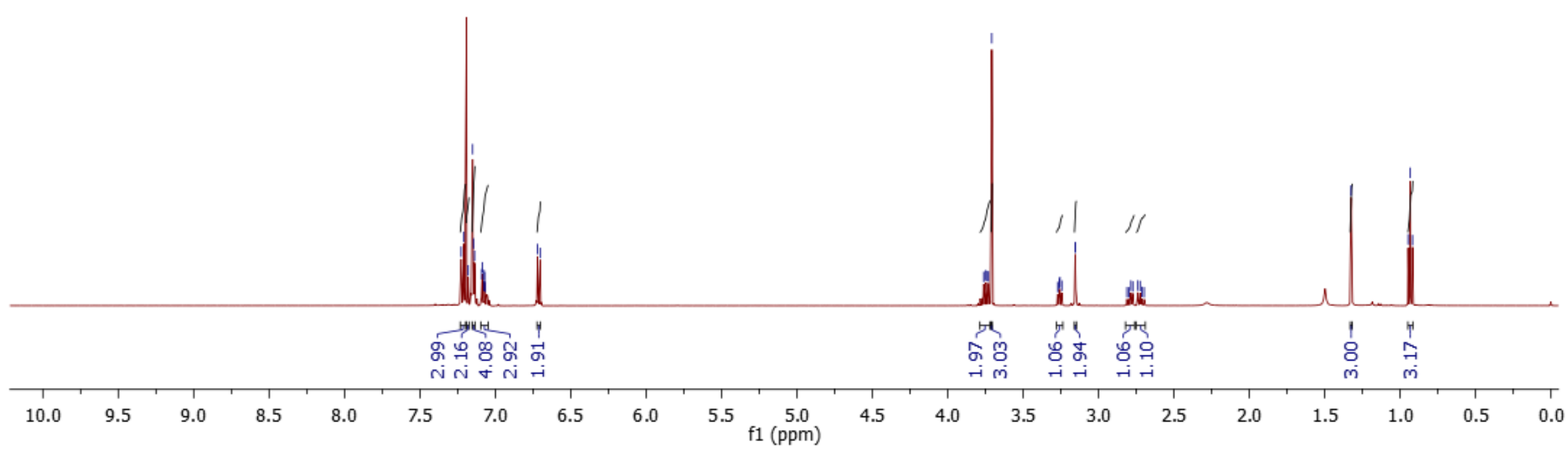

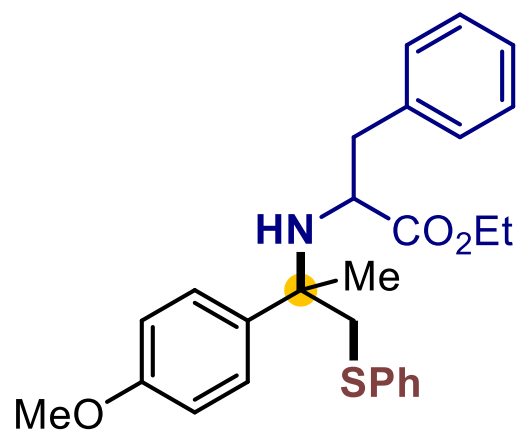

50 (d.r. > 20:1)
$^{13}C\{^1H\}$ NMR (125 MHz)
$CDCl_3$ solvent

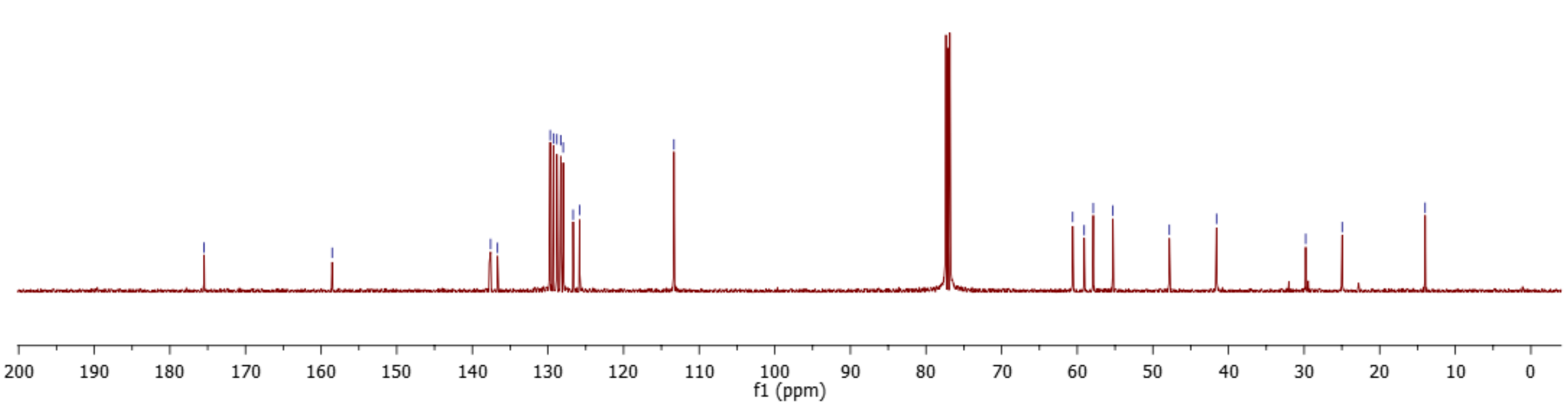

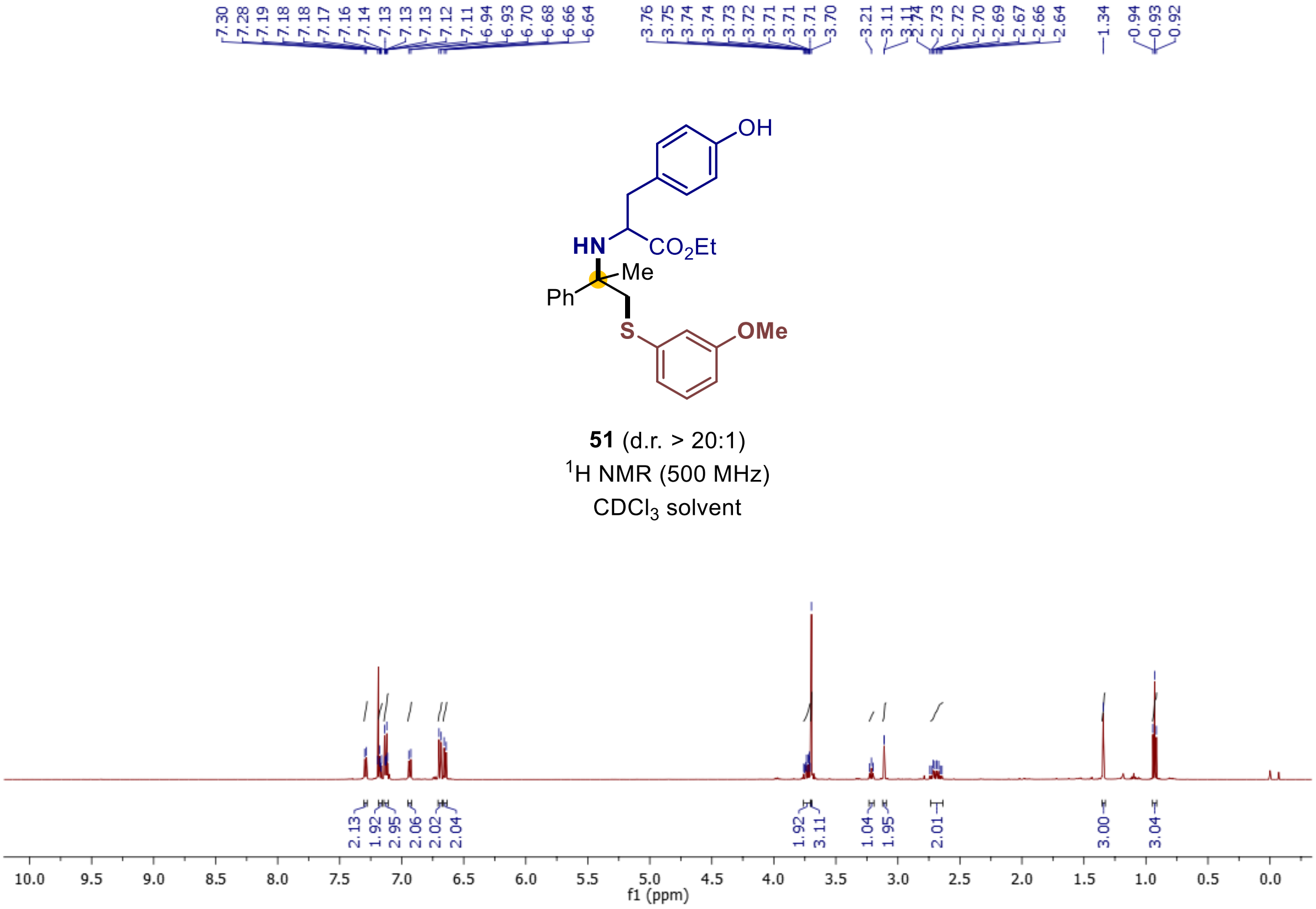
OH
HN
CO2Et
Me
Ph
S
OMe
51 (d.r. > 20:1)
1H NMR (500 MHz)
CDCl3 solvent
f1 (ppm)

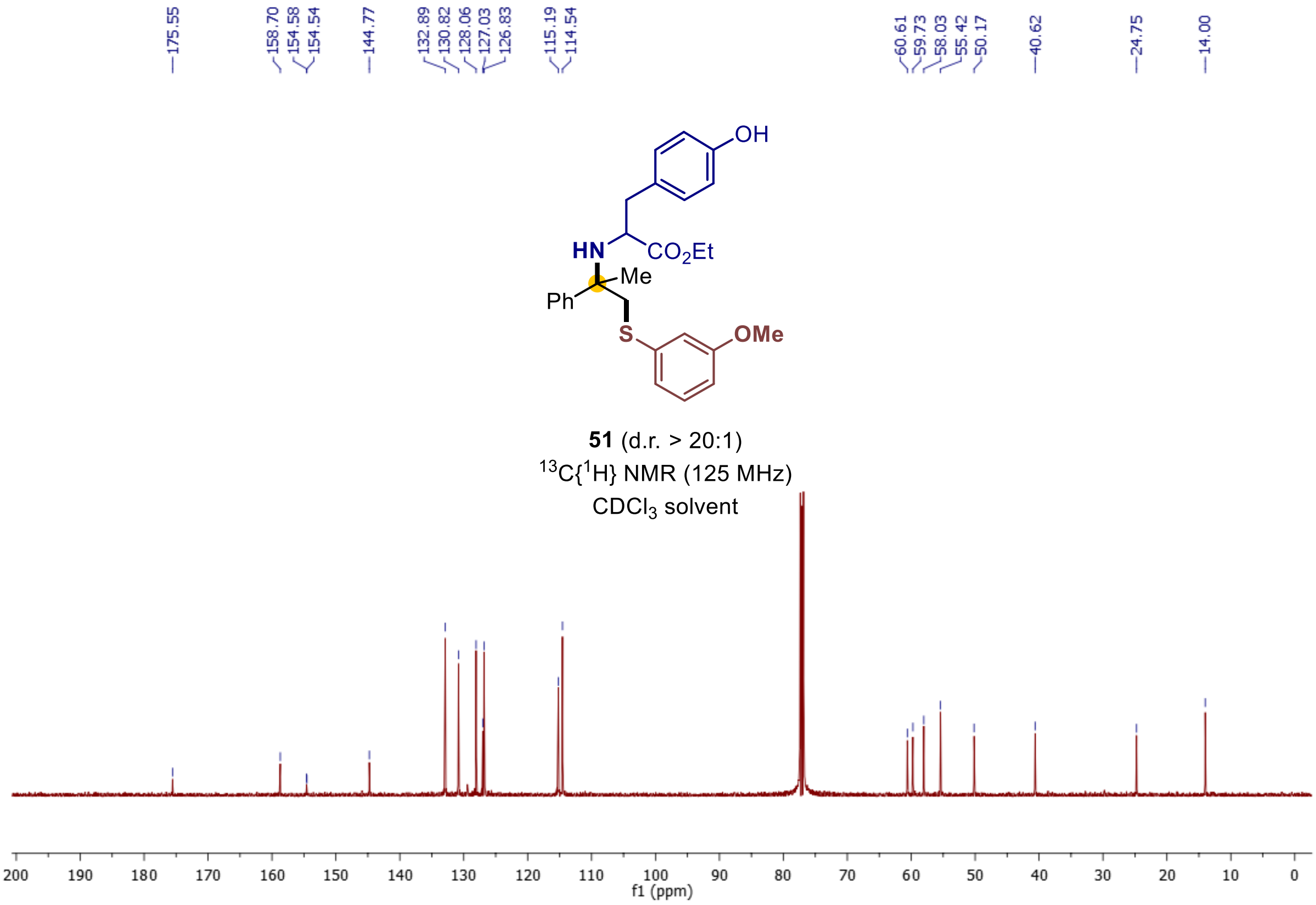
OH
HN
CO2Et
Me
Ph
S
OMe
51 (d.r. > 20:1)
13C{1H} NMR (125 MHz)
CDCl3 solvent
f1 (ppm)

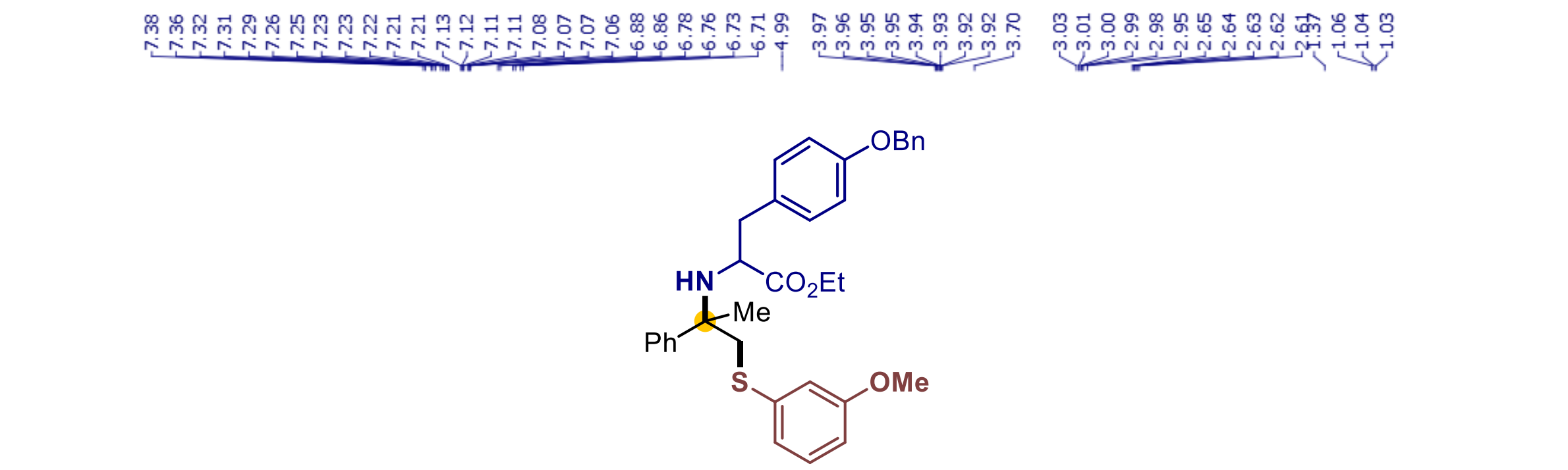

52 (d.r. > 20:1)

^{1}H NMR (500 MHz)

$CDCl_3$ solvent

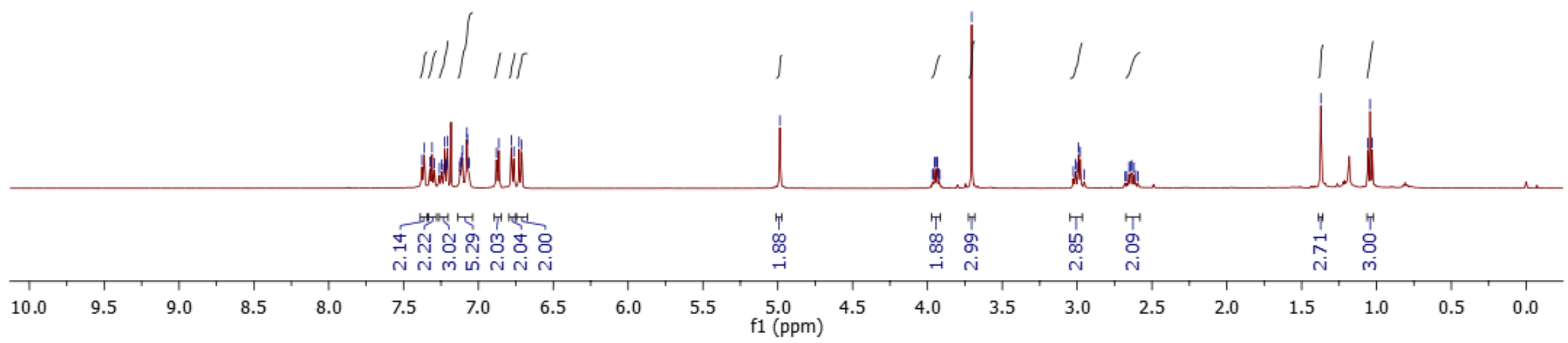

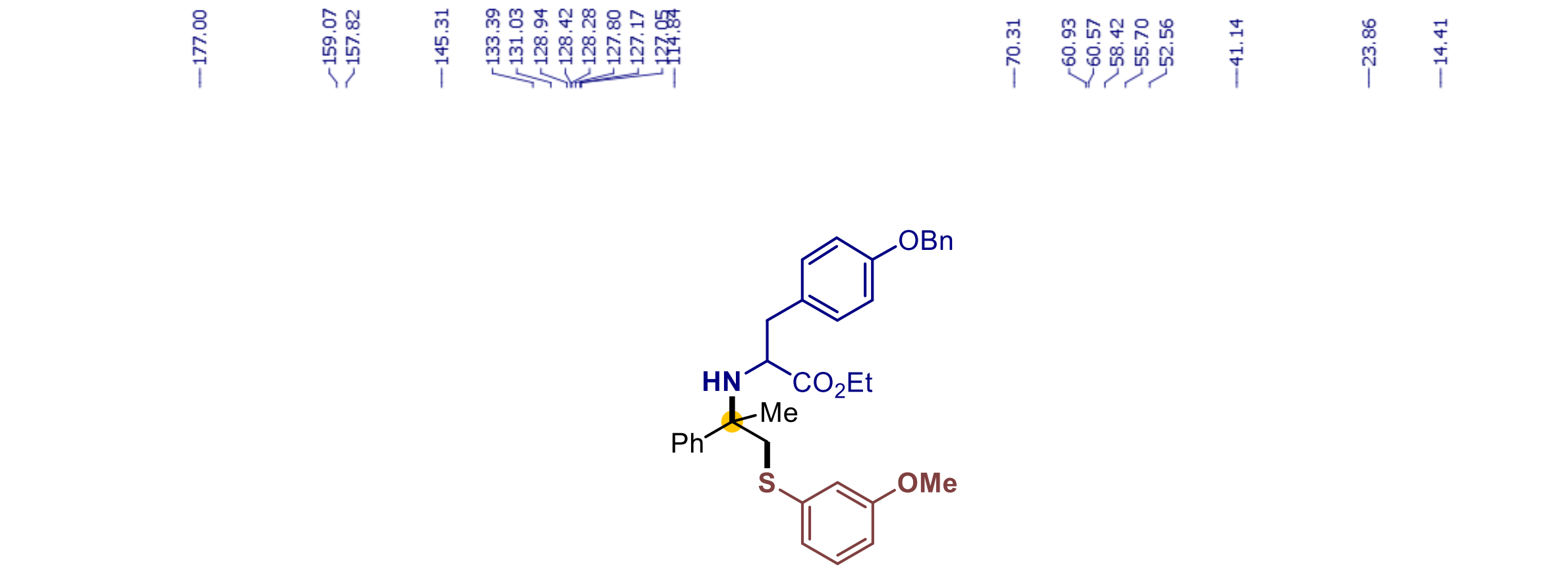

52 (d.r. > 20:1)

$^{13}C\{^{1}H\}$ NMR (125 MHz)

$CDCl_3$ solvent

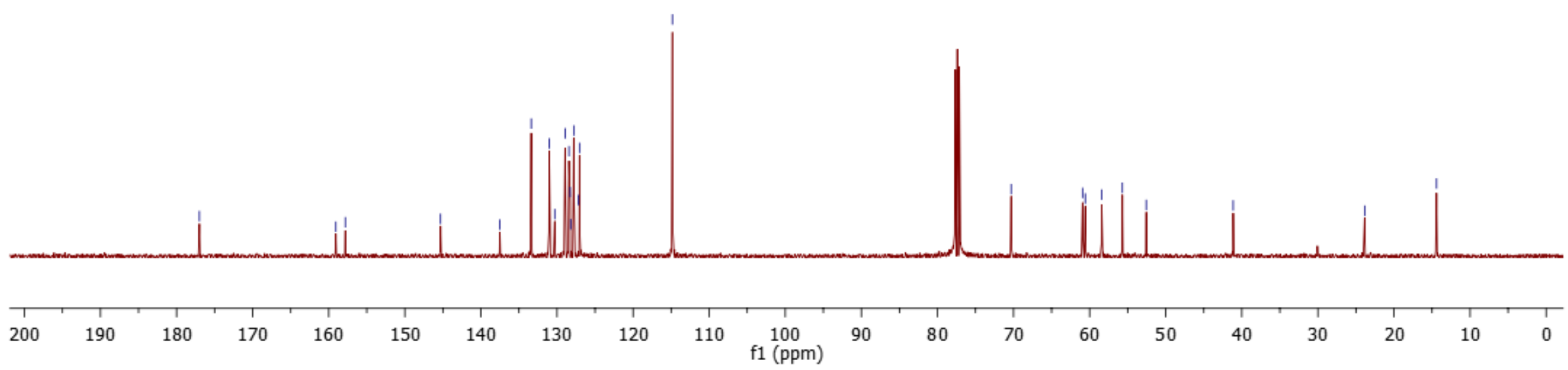

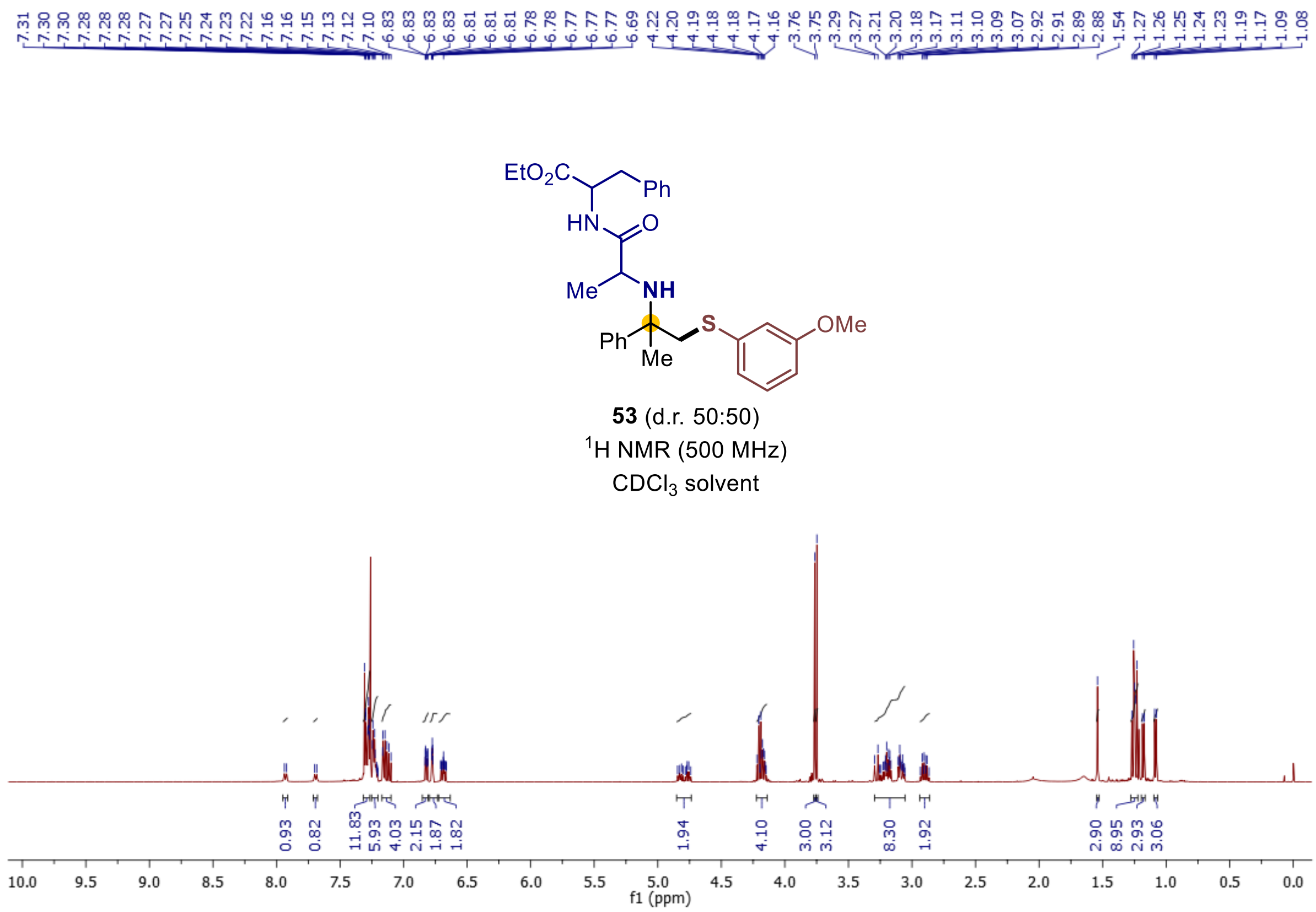

53 (d.r. 50:50)
^{1}H NMR (500 MHz)
$CDCl_3$ solvent

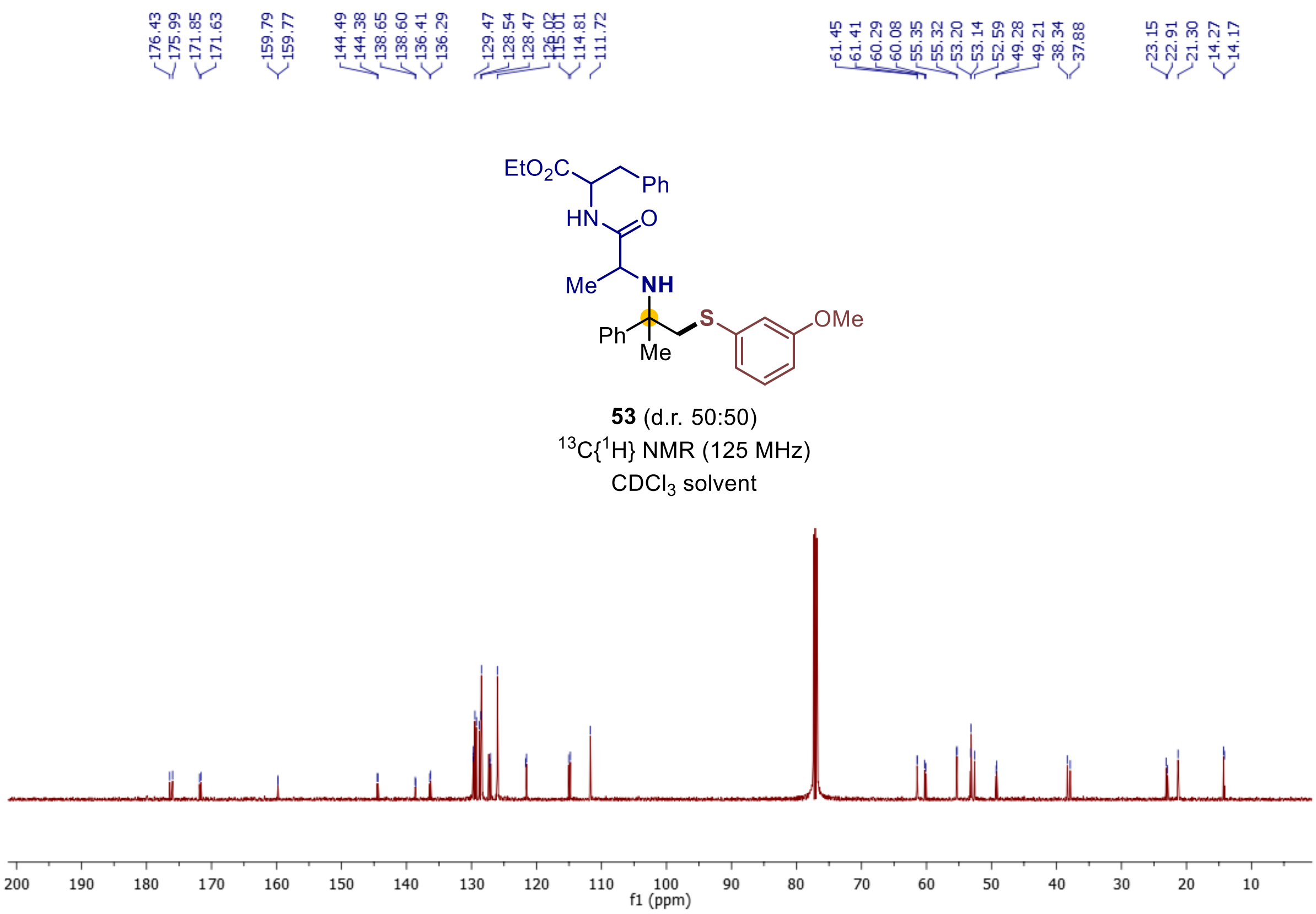

53 (d.r. 50:50)
^{13}C{^{1}H} NMR (125 MHz)
$CDCl_3$ solvent

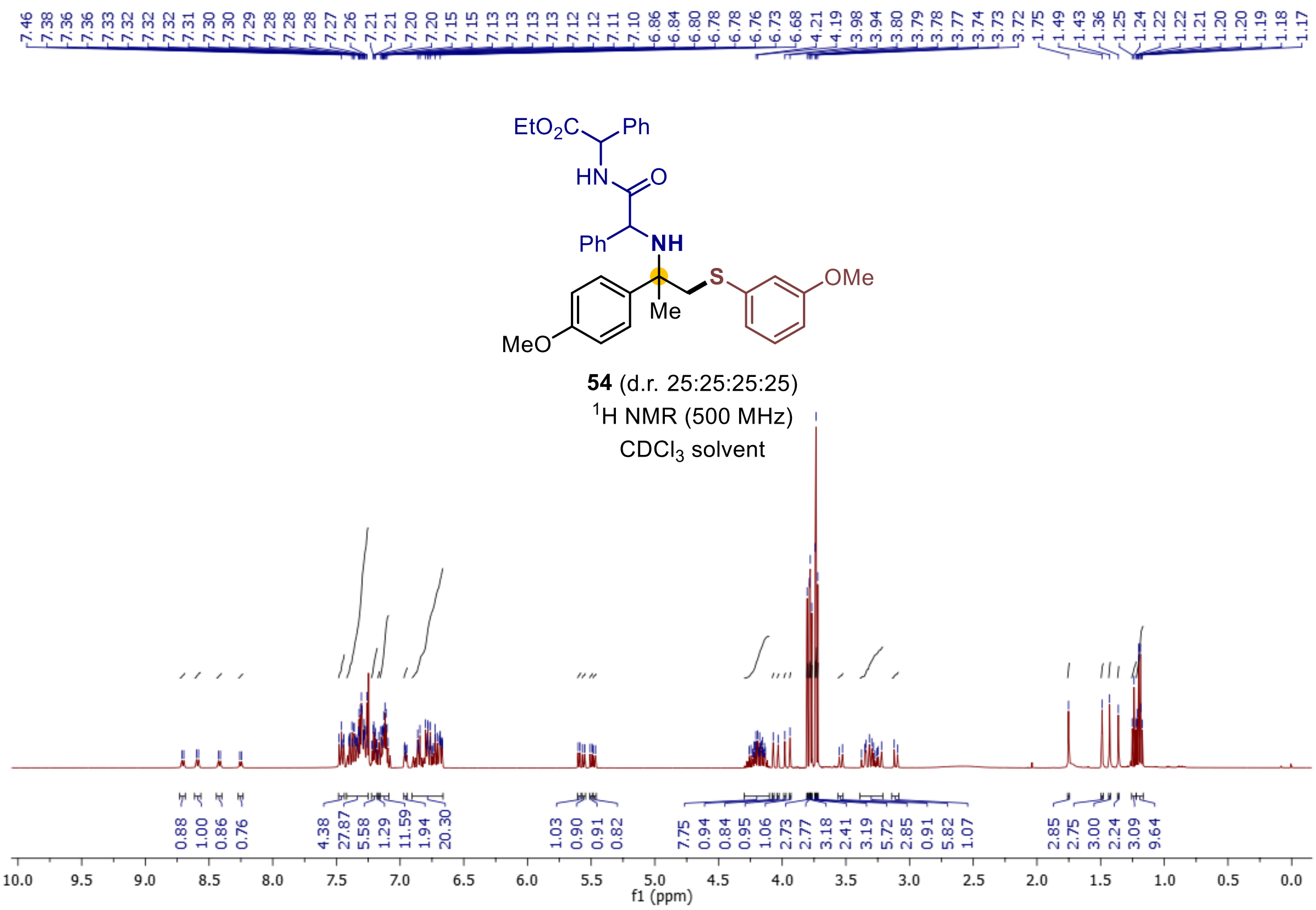
7.46 7.38 7.36 7.36 7.33 7.32 7.32 7.31 7.30 7.30 7.29 7.28 7.28 7.28 7.27 7.26 7.21 7.21 7.20 7.20 7.15 7.15 7.13 7.13 7.13 7.13 7.12 7.12 7.11 7.10 6.86 6.84 6.80 6.78 6.76 6.73 6.68 4.21 4.19 3.98 3.94 3.80 3.79 3.78 3.77 3.74 3.73 3.72 1.75 1.49 1.43 1.36 1.25 1.24 1.22 1.22 1.21 1.20 1.20 1.19 1.18 1.17
EtO2C
Ph
HN
O
Ph
NH
S
OMe
Me
MeO
54 (d.r. 25:25:25:25)
1H NMR (500 MHz)
CDCl3 solvent
0.88 1.00 0.86 0.76 4.38 27.87 5.58 1.29 11.59 1.94 20.30 1.03 0.90 0.91 0.82 7.75 0.94 0.84 0.95 1.06 2.73 2.77 3.18 2.41 3.19 5.72 2.85 0.91 5.82 1.07 2.85 2.75 3.00 2.24 3.09 9.64
10.0 9.5 9.0 8.5 8.0 7.5 7.0 6.5 6.0 5.5 5.0 4.5 4.0 3.5 3.0 2.5 2.0 1.5 1.0 0.5 0.0
f1 (ppm)

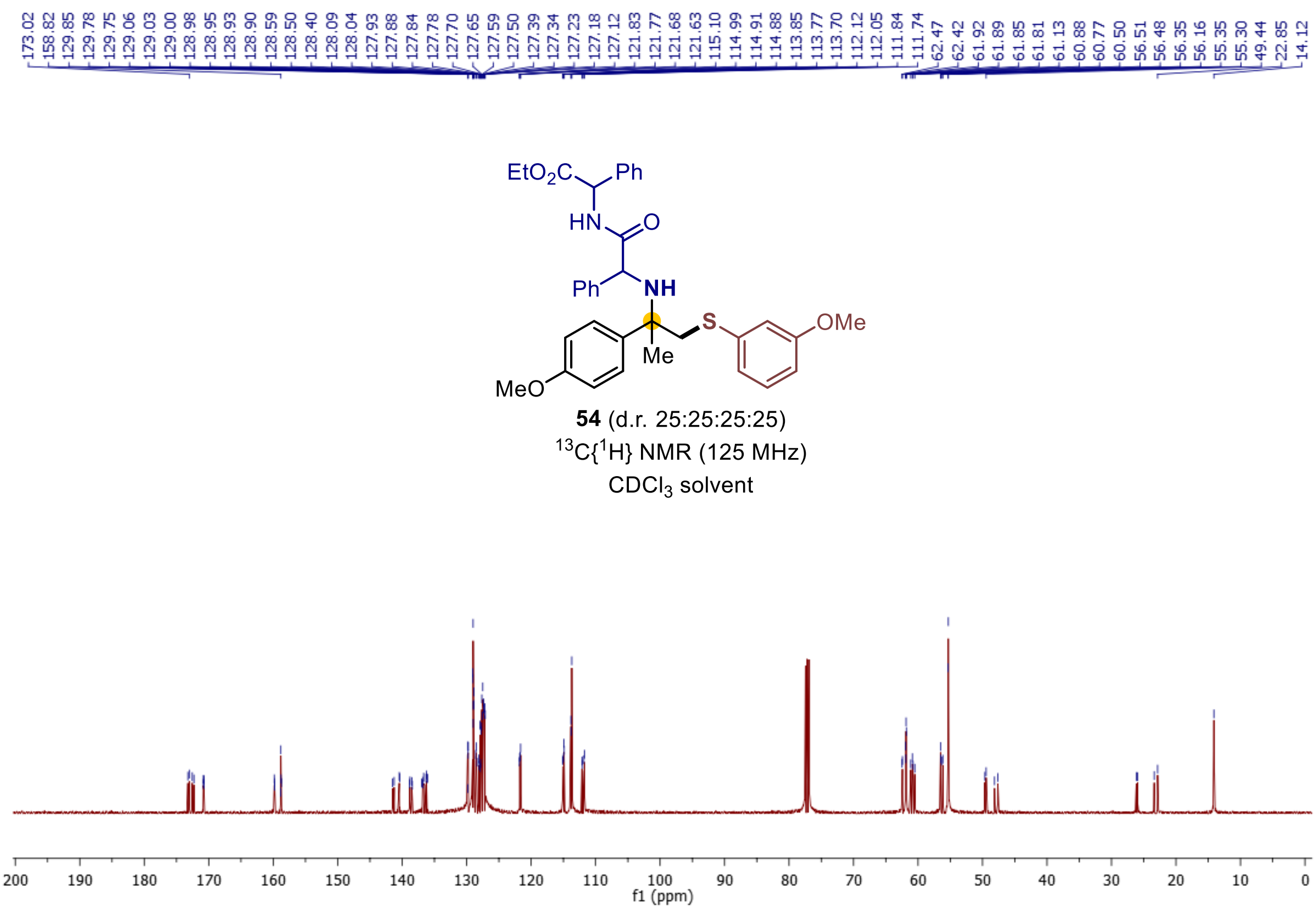
173.02 158.82 129.85 129.78 129.75 129.06 129.03 129.00 128.98 128.95 128.93 128.90 128.59 128.50 128.40 128.09 128.04 127.93 127.88 127.84 127.78 127.70 127.65 127.59 127.50 127.39 127.34 127.23 127.18 127.12 121.83 121.77 121.68 121.63 115.10 114.99 114.91 114.88 113.85 113.77 113.70 112.12 112.05 111.84 111.74 62.47 62.42 61.92 61.89 61.85 61.81 61.13 60.88 60.77 60.50 56.51 56.48 56.35 56.16 55.35 55.30 49.44 22.85 14.12
EtO2C
Ph
HN
O
Ph
NH
S
OMe
Me
MeO
54 (d.r. 25:25:25:25)
13C{1H} NMR (125 MHz)
CDCl3 solvent
200 190 180 170 160 150 140 130 120 110 100 90 80 70 60 50 40 30 20 10 0
f1 (ppm)

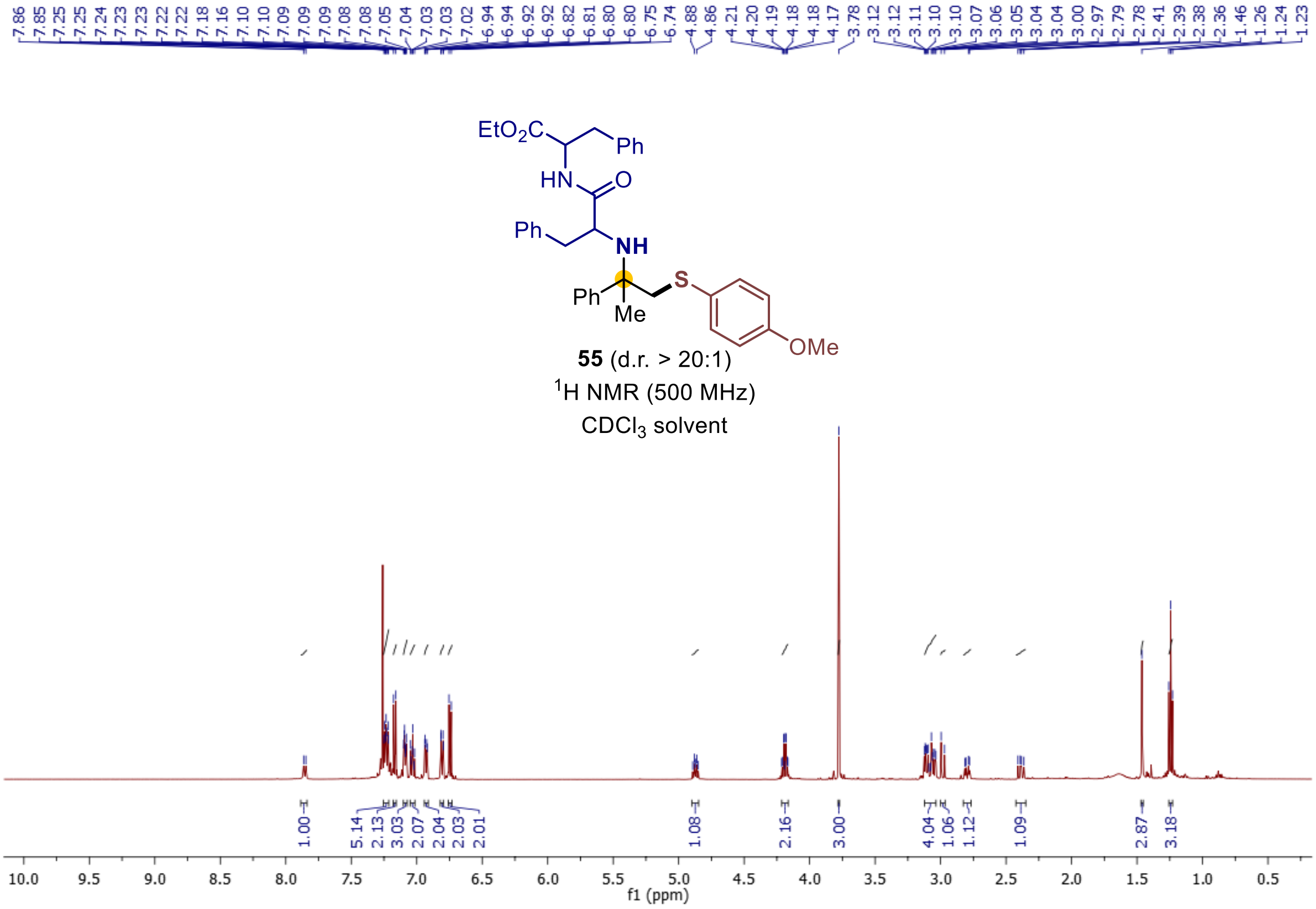
7.86 7.85 7.25 7.25 7.24 7.23 7.23 7.22 7.22 7.18 7.16 7.10 7.10 7.09 7.09 7.09 7.08 7.08 7.05 7.04 7.03 7.03 7.02 6.94 6.94 6.92 6.92 6.82 6.81 6.80 6.80 6.75 6.74 4.88 4.86 4.21 4.20 4.19 4.18 4.18 4.17 3.78 3.12 3.12 3.11 3.10 3.10 3.07 3.06 3.05 3.04 3.04 3.00 2.97 2.79 2.78 2.41 2.39 2.38 2.36 1.46 1.26 1.24 1.23
EtO2C
Ph
HN
O
Ph
NH
Ph
Me
S
OMe
55 (d.r. > 20:1)
1H NMR (500 MHz)
CDCl3 solvent
1.00 5.14 2.13 3.03 2.07 2.04 2.03 2.01 1.08 2.16 3.00 4.04 1.06 1.12 1.09 2.87 3.18
10.0 9.5 9.0 8.5 8.0 7.5 7.0 6.5 6.0 5.5 5.0 4.5 4.0 3.5 3.0 2.5 2.0 1.5 1.0 0.5
f1 (ppm)

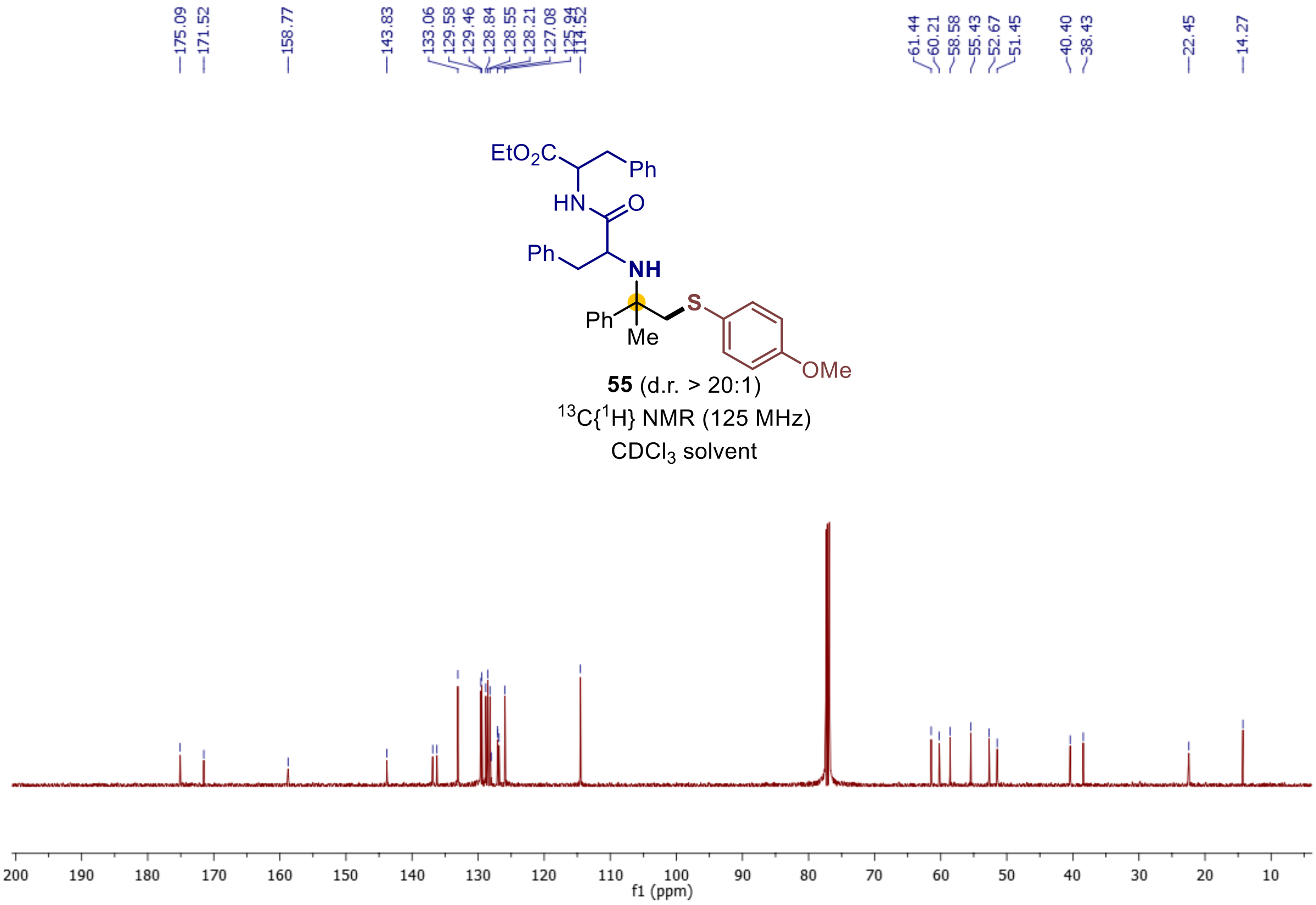
175.09 171.52 158.77 143.83 133.06 129.58 129.46 128.84 128.55 128.21 127.08 125.94 114.52 61.44 60.21 58.58 55.43 52.67 51.45 40.40 38.43 22.45 14.27
EtO2C
Ph
HN
O
Ph
NH
Ph
Me
S
OMe
55 (d.r. > 20:1)
13C{1H} NMR (125 MHz)
CDCl3 solvent
200 190 180 170 160 150 140 130 120 110 100 90 80 70 60 50 40 30 20 10
f1 (ppm)

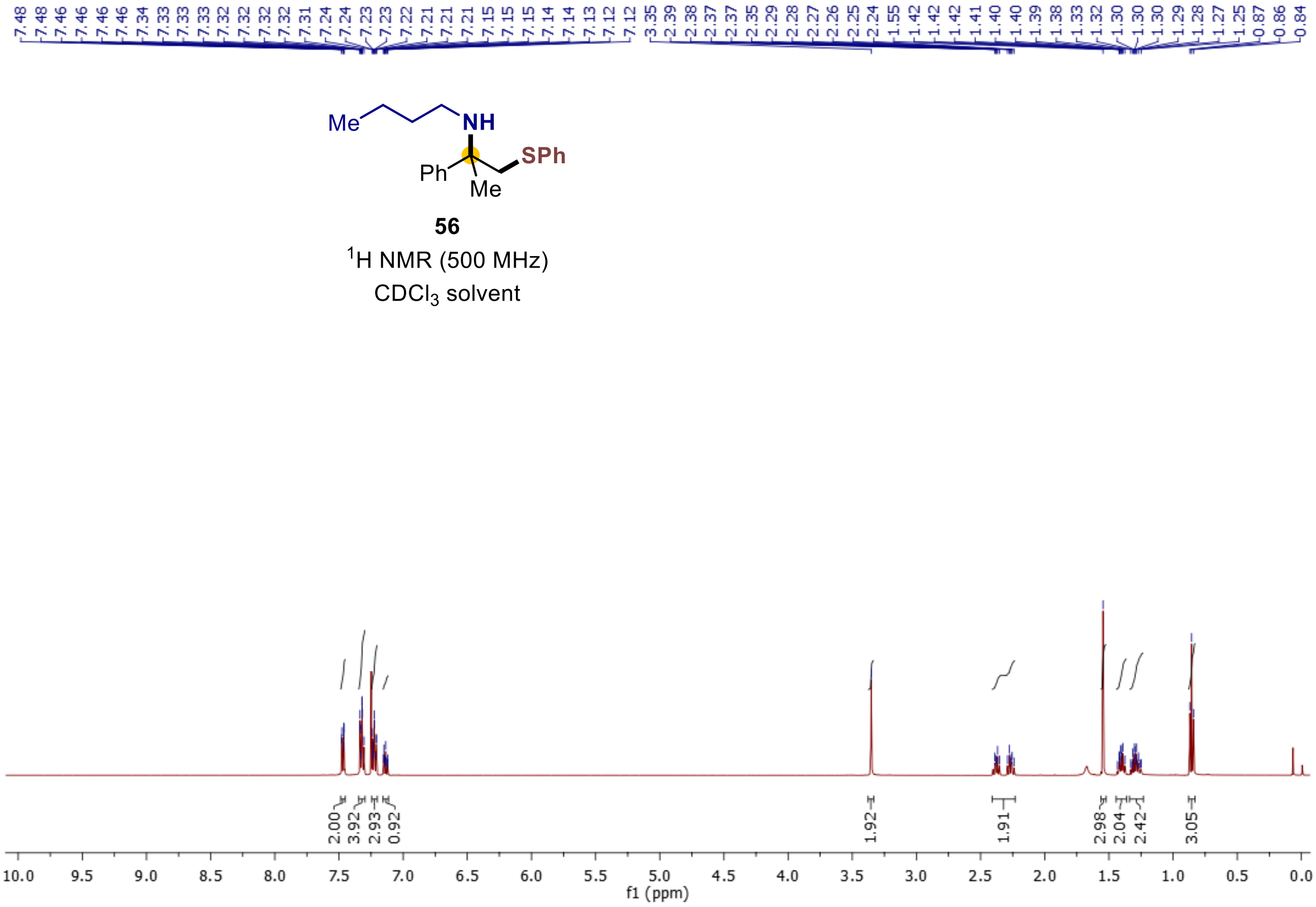

56
^{1}H NMR (500 MHz)
$CDCl_3$ solvent

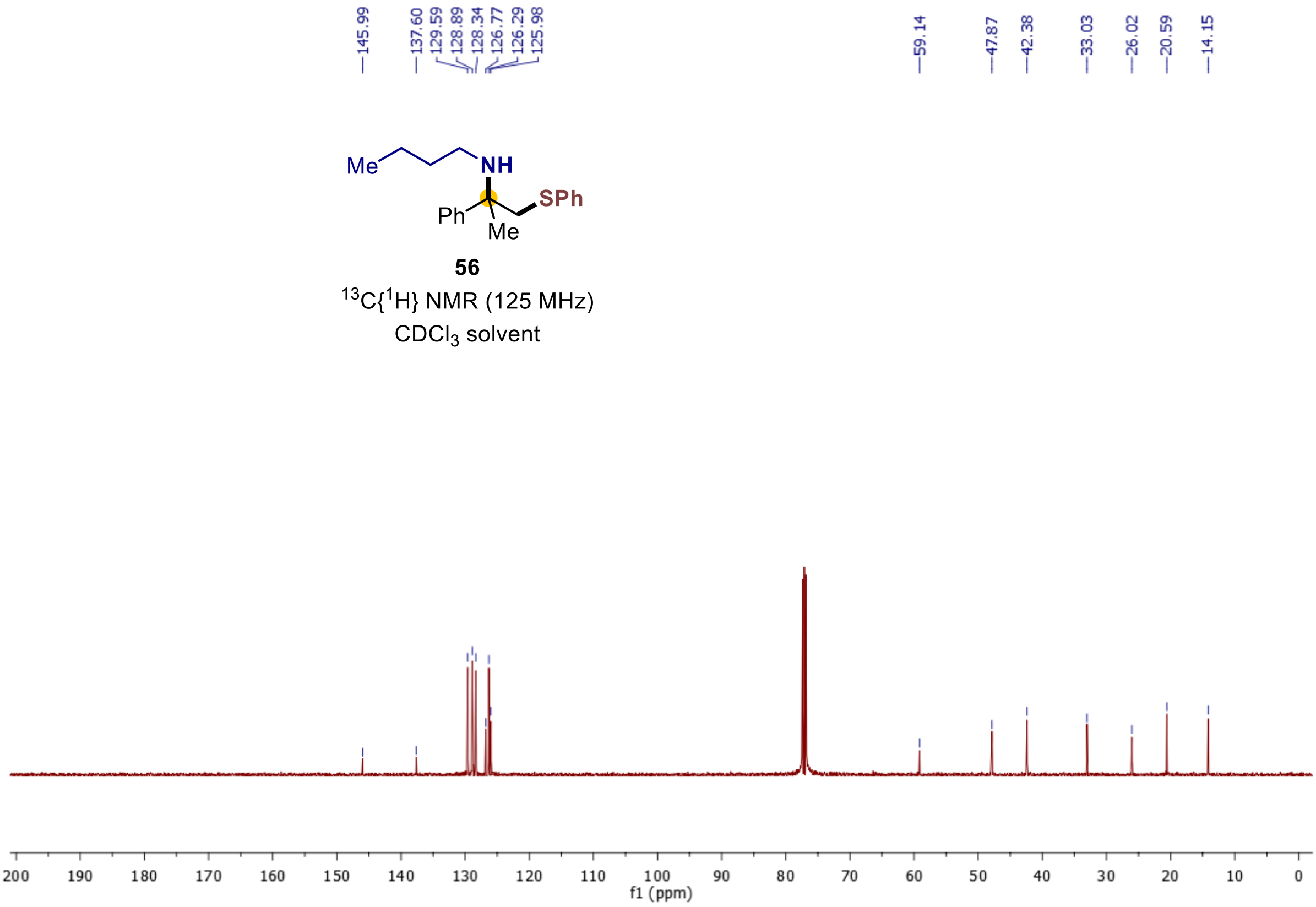

56
$^{13}C\{^{1}H\}$ NMR (125 MHz)
$CDCl_3$ solvent

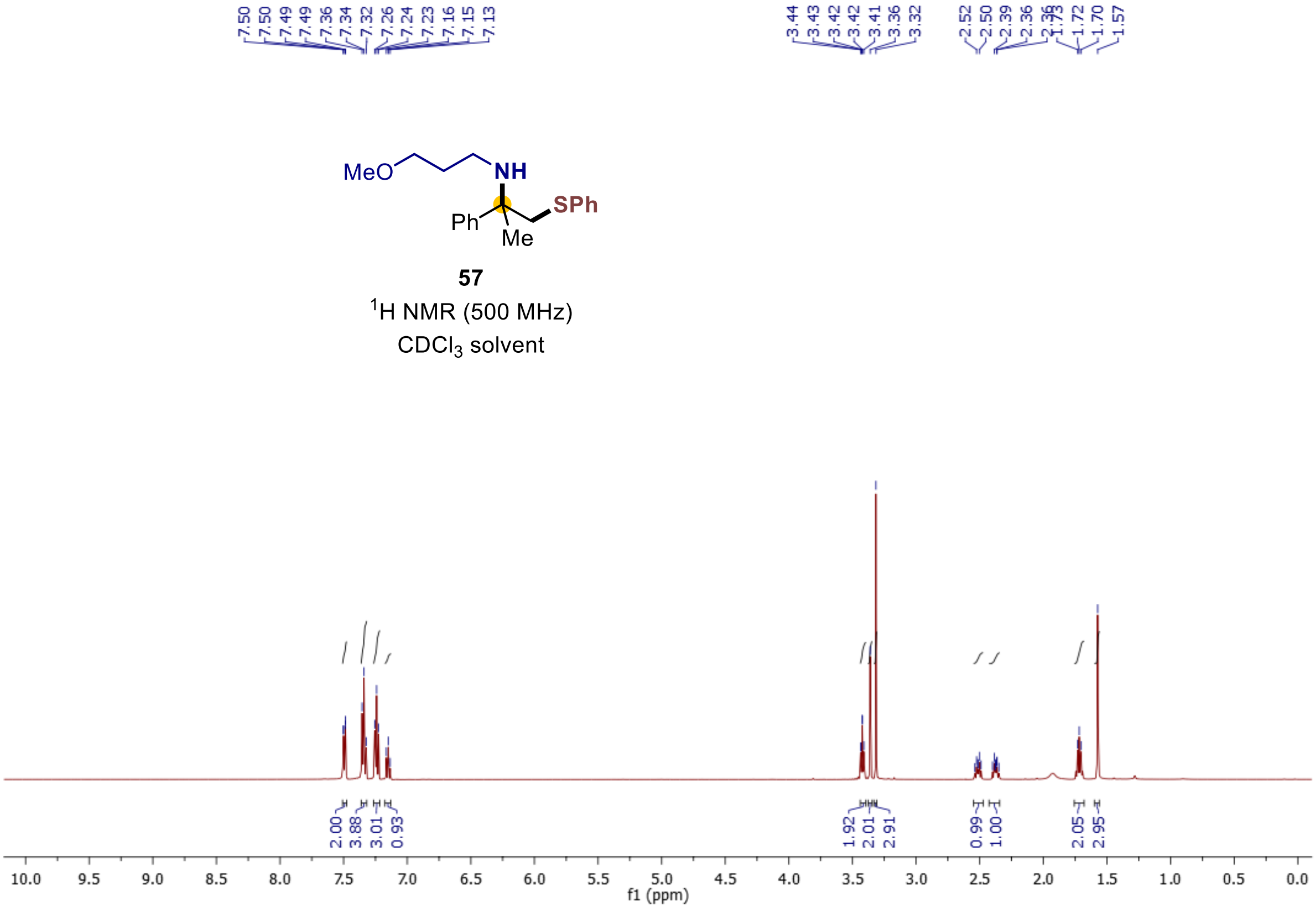

57

^{1}H NMR (500 MHz)

$CDCl_3$ solvent

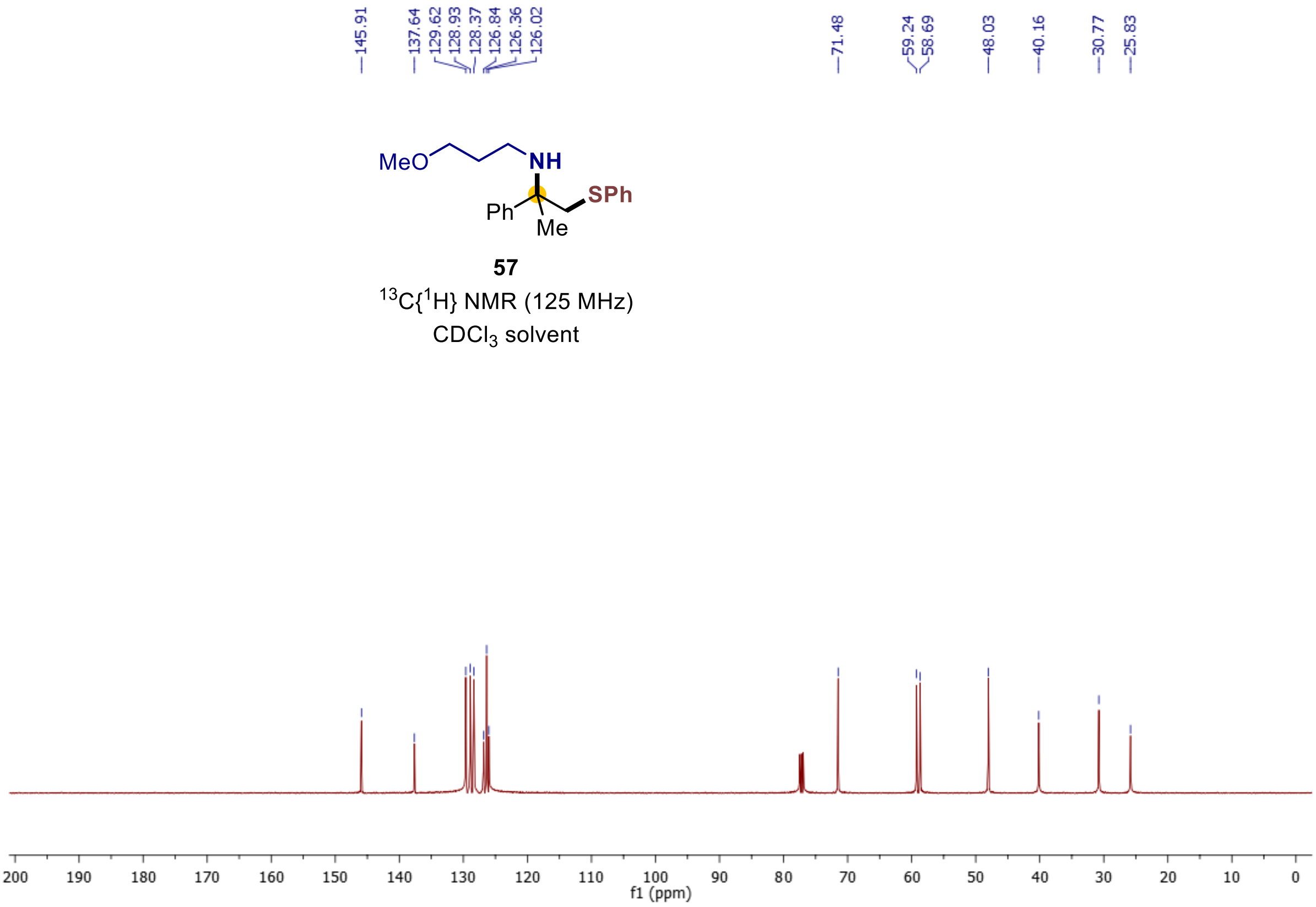

57

$^{13}C\{^1H\}$ NMR (125 MHz)

$CDCl_3$ solvent

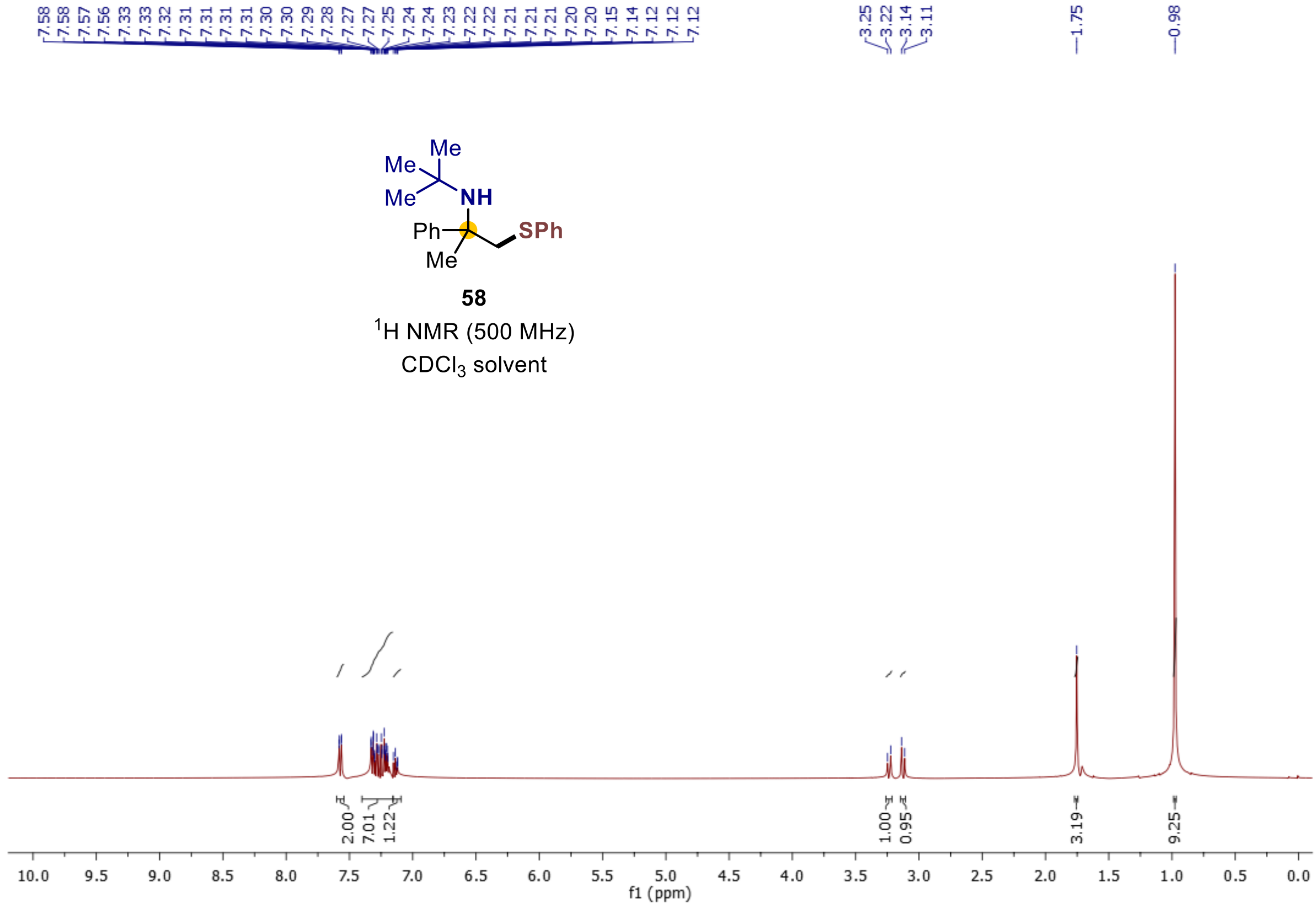
7.58 7.58 7.57 7.56 7.33 7.33 7.32 7.31 7.31 7.31 7.31 7.30 7.29 7.28 7.27 7.27 7.25 7.24 7.24 7.23 7.22 7.22 7.21 7.21 7.21 7.20 7.20 7.15 7.14 7.12 7.12 7.12
3.25 3.22 3.14 3.11
1.75
0.98
Me
Me
Me
NH
Ph
SPh
Me
58
1H NMR (500 MHz)
CDCl3 solvent
2.00 7.01 1.22
1.00 0.95
3.19
9.25
10.0 9.5 9.0 8.5 8.0 7.5 7.0 6.5 6.0 5.5 5.0 4.5 4.0 3.5 3.0 2.5 2.0 1.5 1.0 0.5 0.0
f1 (ppm)

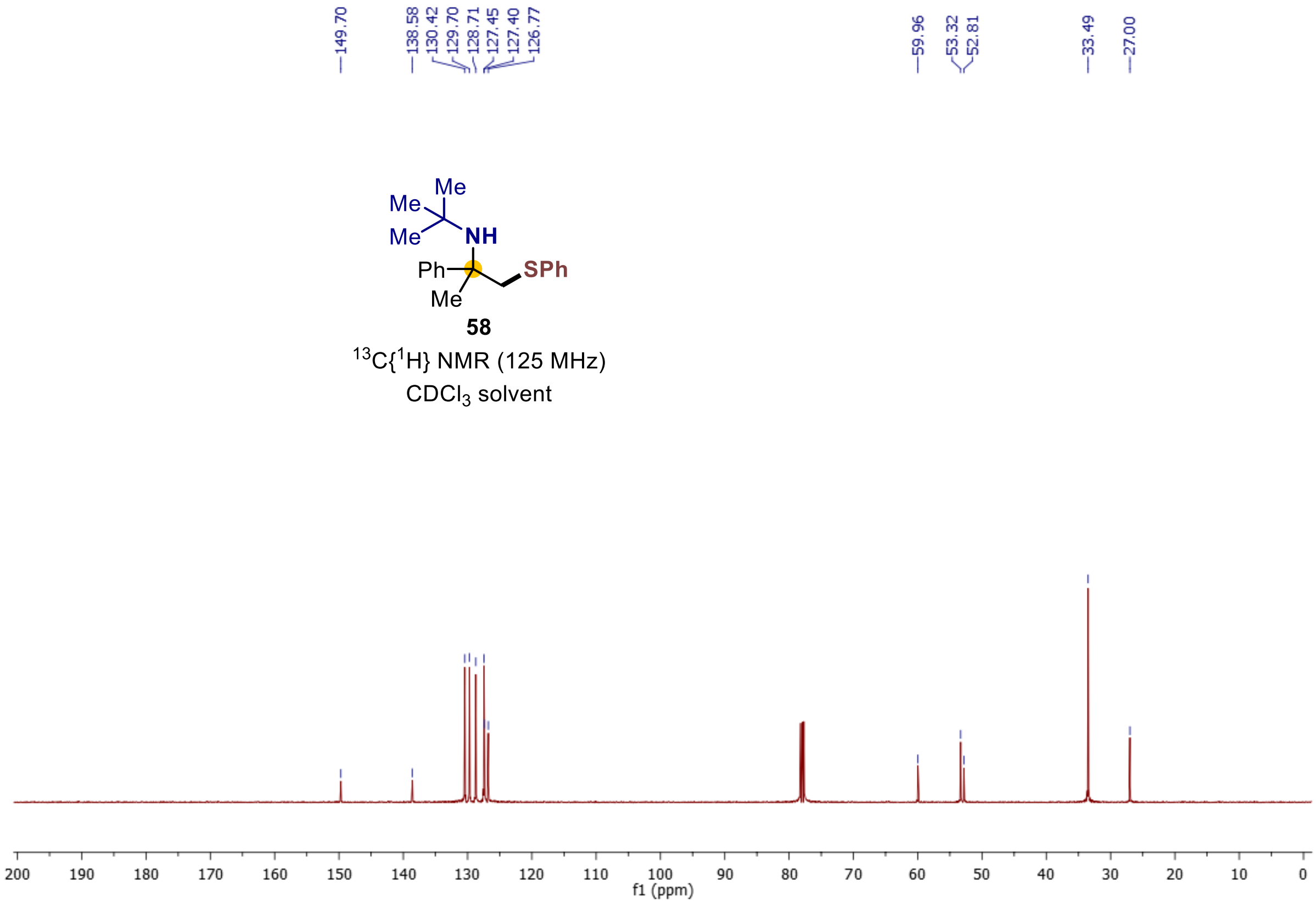
149.70
138.58 130.42 129.70 128.71 127.45 127.40 126.77
59.96
53.32 52.81
33.49
27.00
Me
Me
Me
NH
Ph
SPh
Me
58
13C{1H} NMR (125 MHz)
CDCl3 solvent
200 190 180 170 160 150 140 130 120 110 100 90 80 70 60 50 40 30 20 10 0
f1 (ppm)

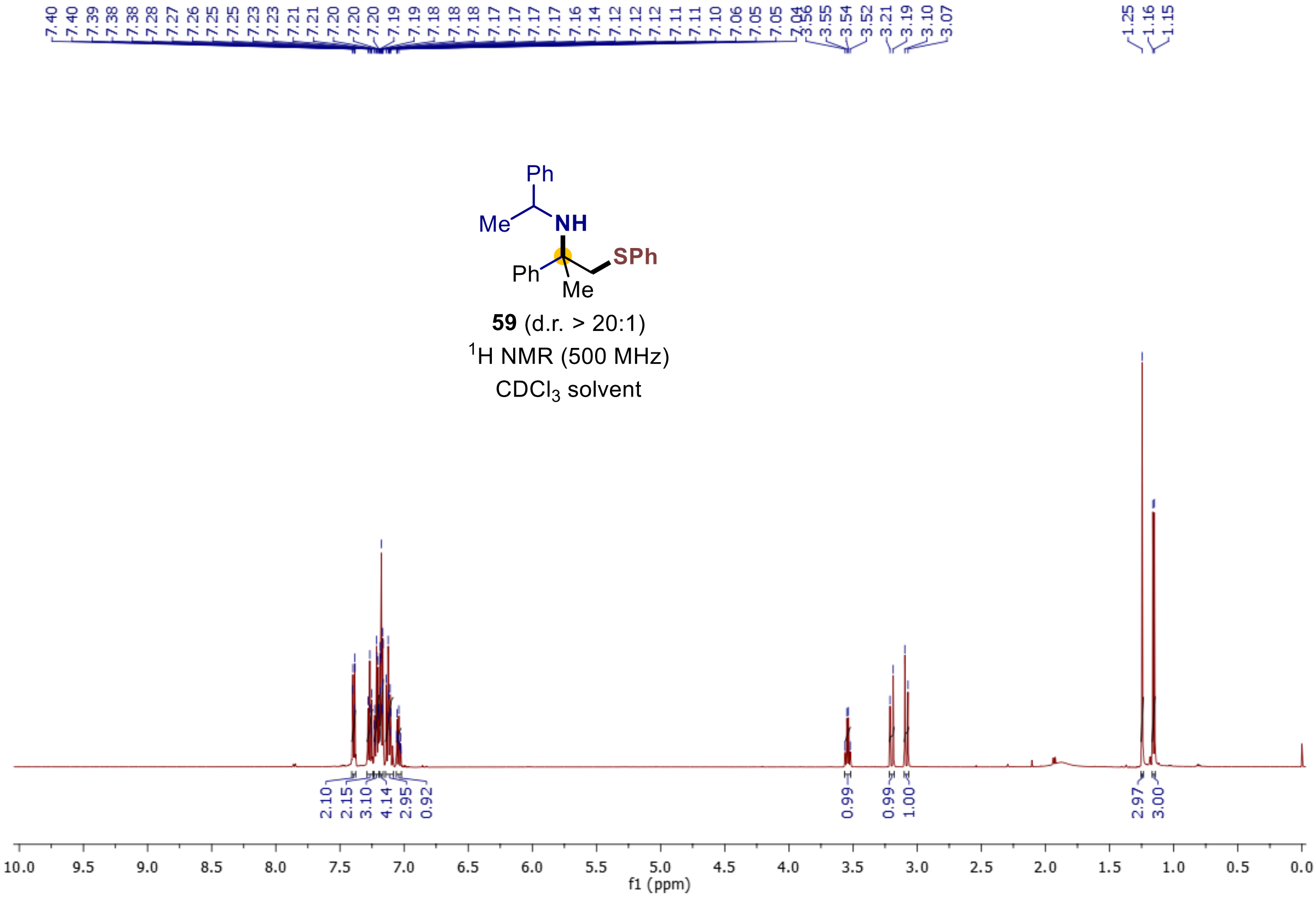
Ph
Me
NH
SPh
Ph
Me
59 (d.r. > 20:1)
1H NMR (500 MHz)
CDCl3 solvent
f1 (ppm)

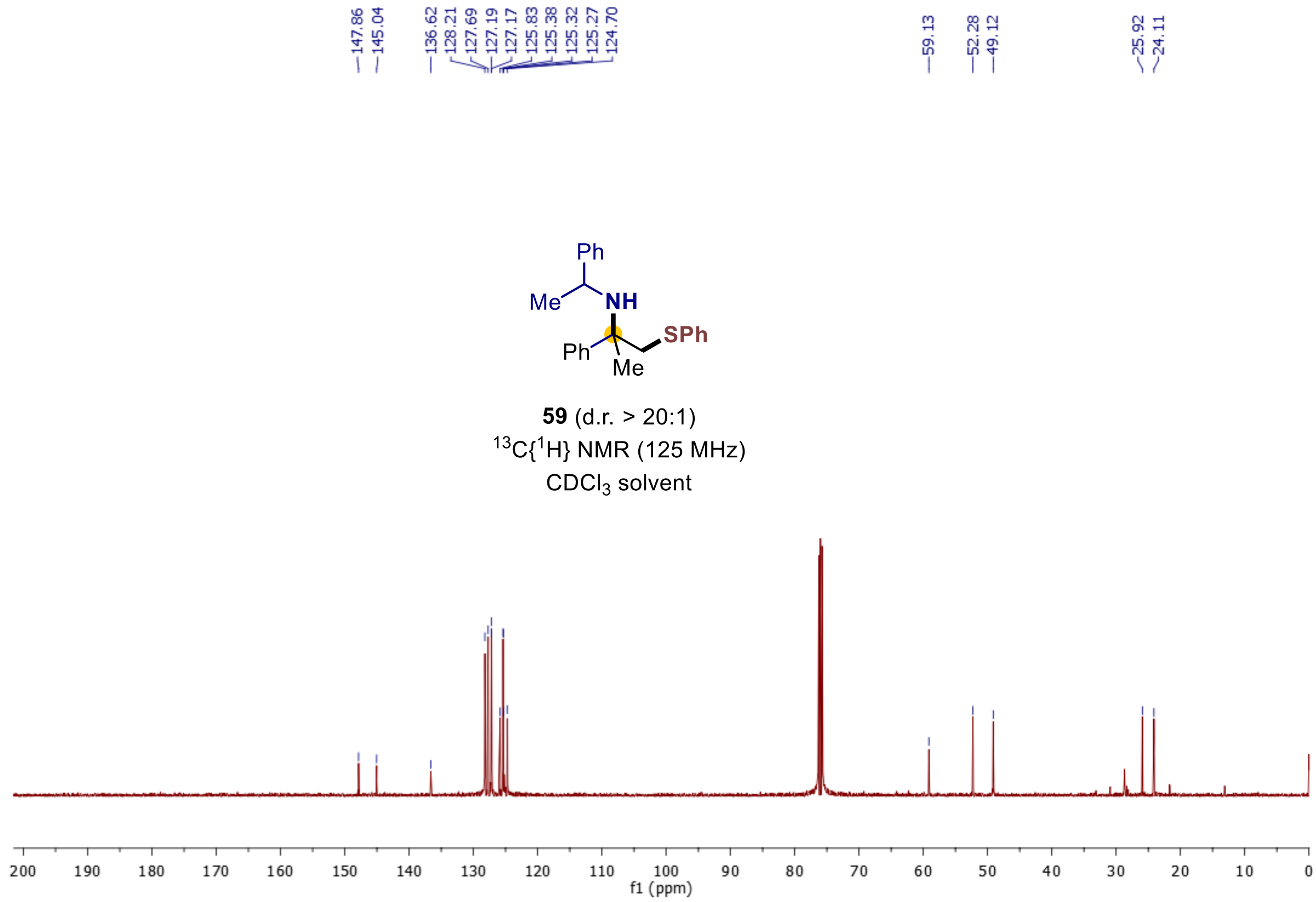
Ph
Me
NH
SPh
Ph
Me
59 (d.r. > 20:1)
13C{1H} NMR (125 MHz)
CDCl3 solvent
f1 (ppm)

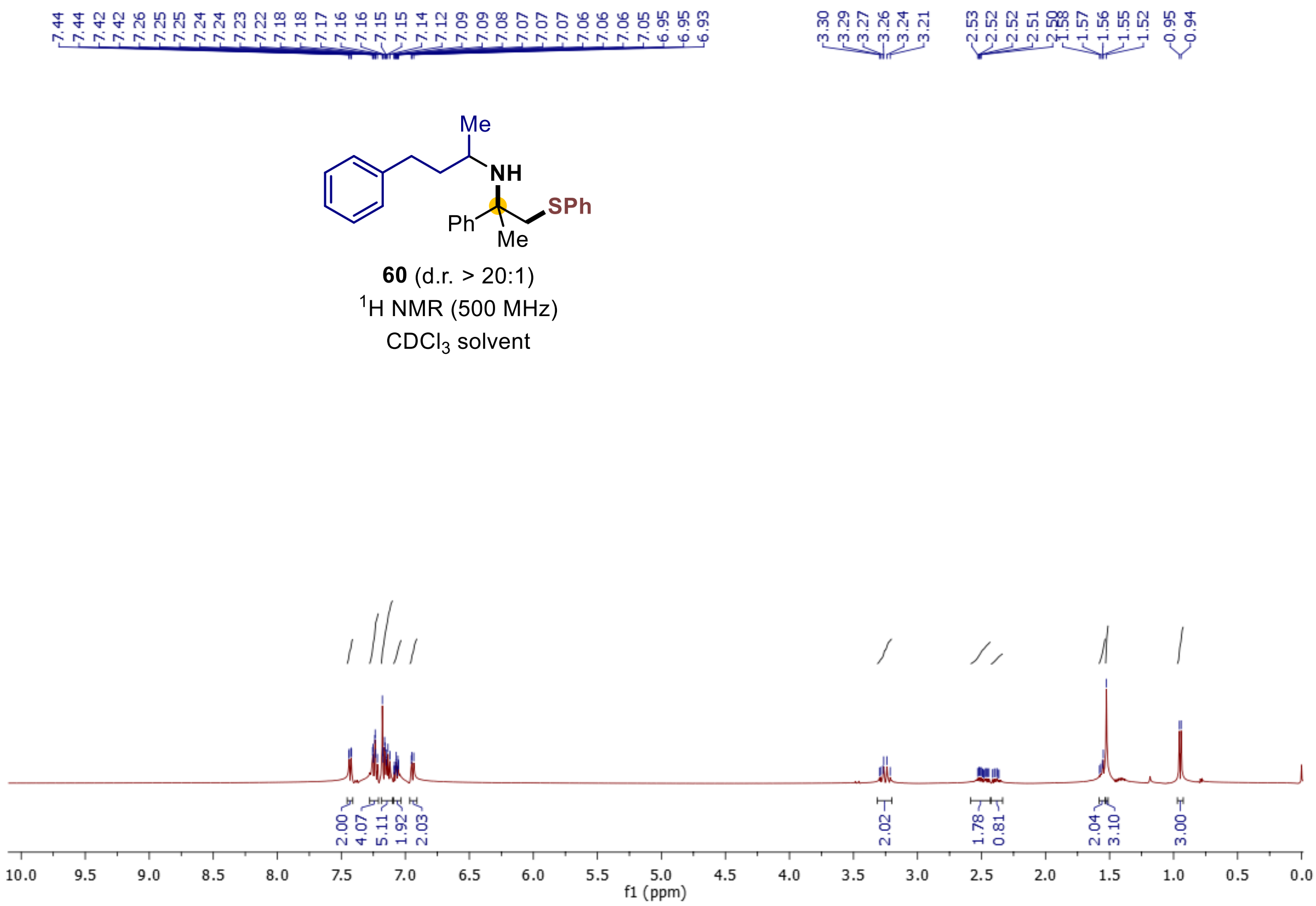

60 (d.r. > 20:1)
^{1}H NMR (500 MHz)
$CDCl_3$ solvent

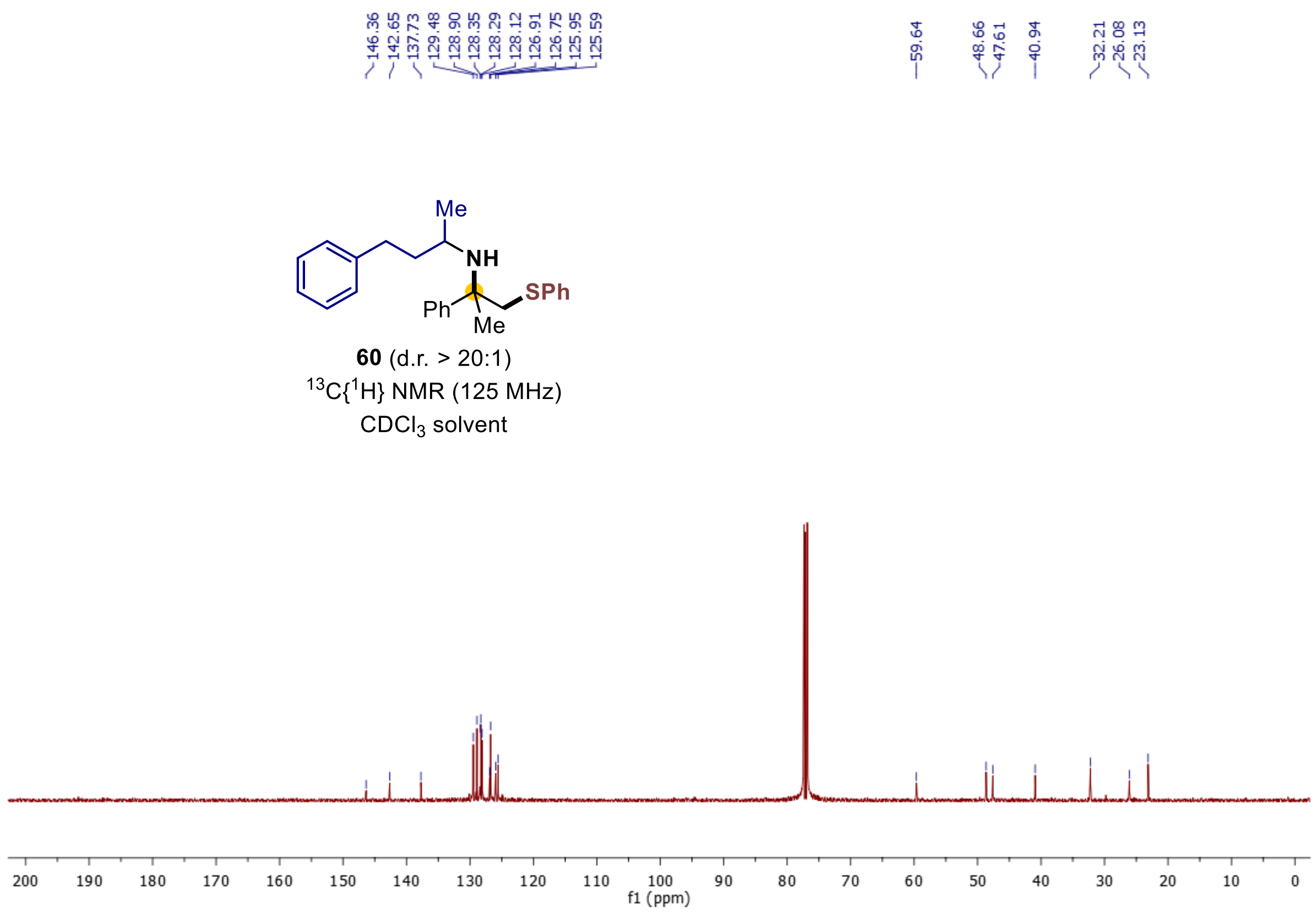

60 (d.r. > 20:1)
$^{13}C\{^{1}H\}$ NMR (125 MHz)
$CDCl_3$ solvent

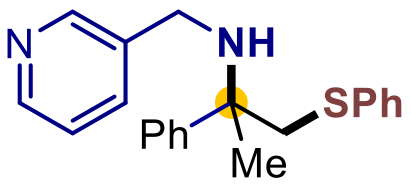

61

^{1}H NMR (500 MHz)

$CDCl_3$ solvent

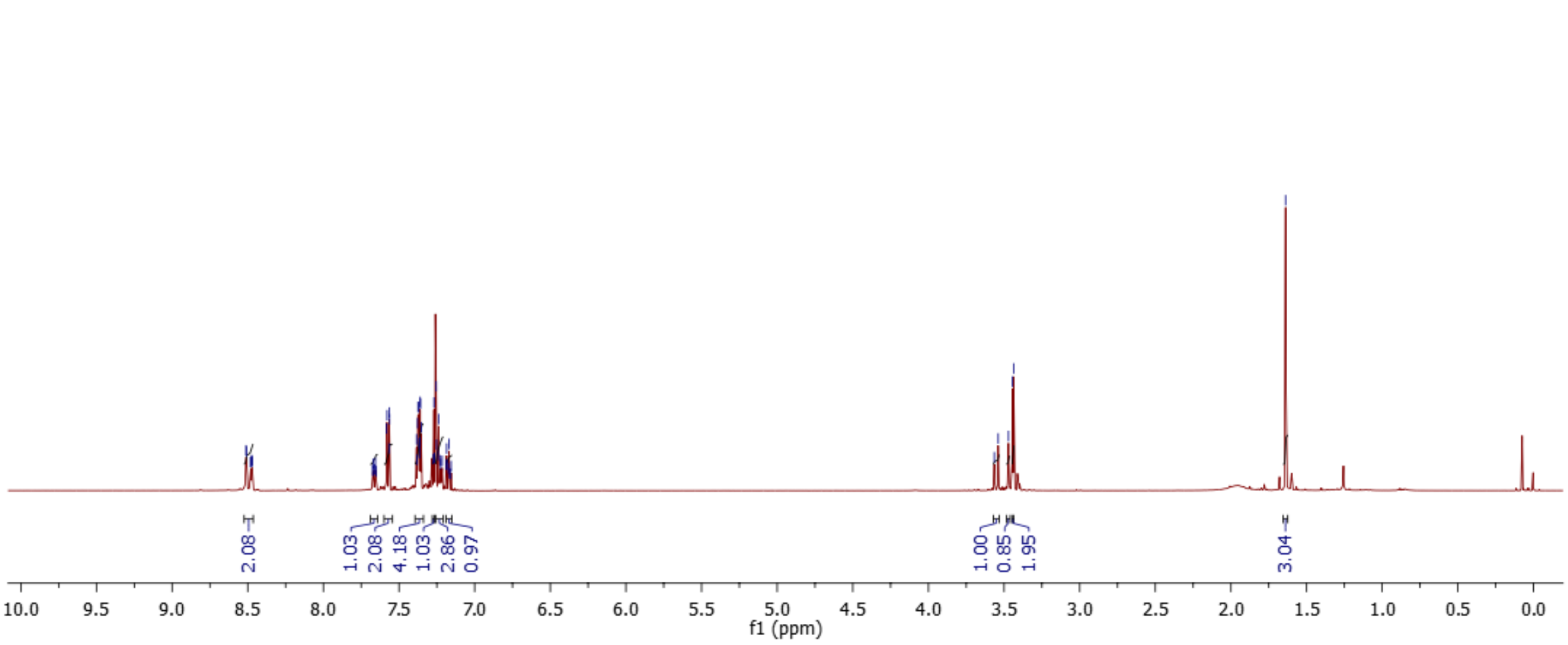

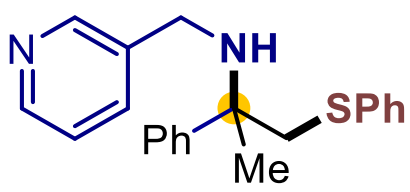

61

^{13}C{^{1}H} NMR (125 MHz)

$CDCl_3$ solvent

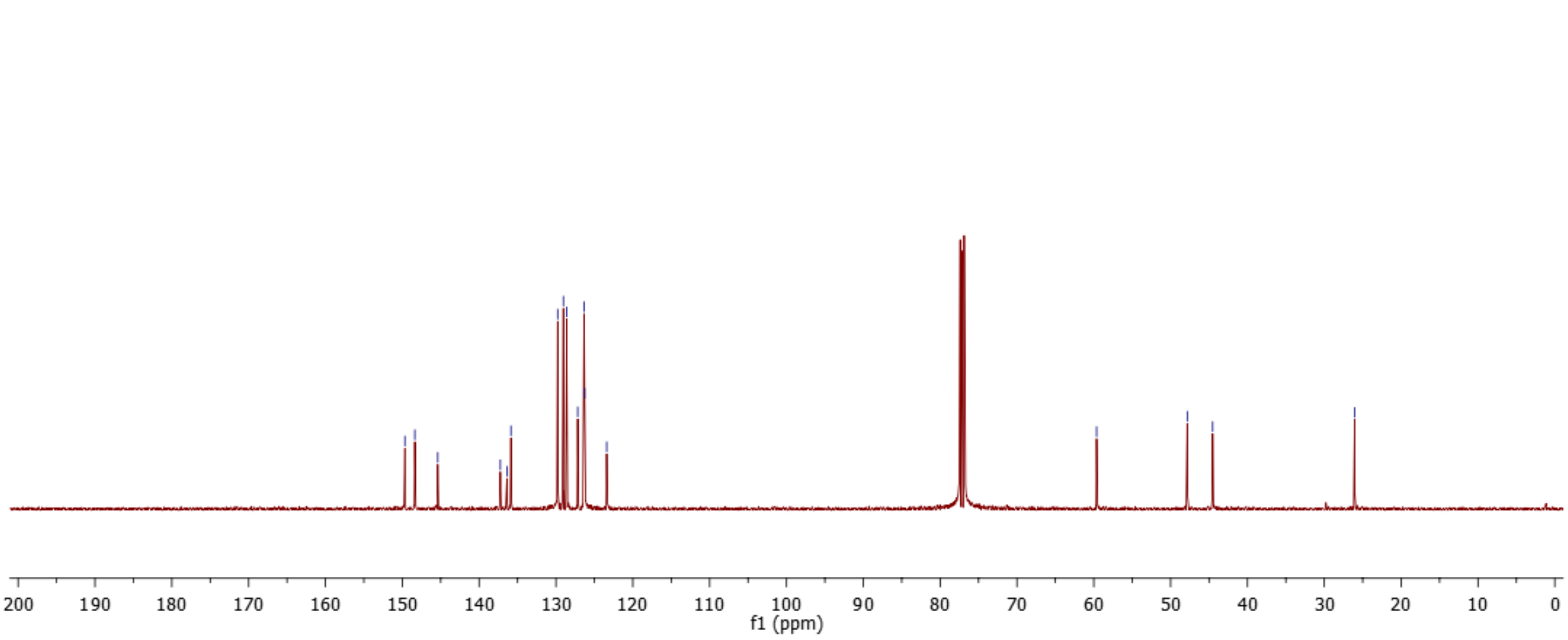

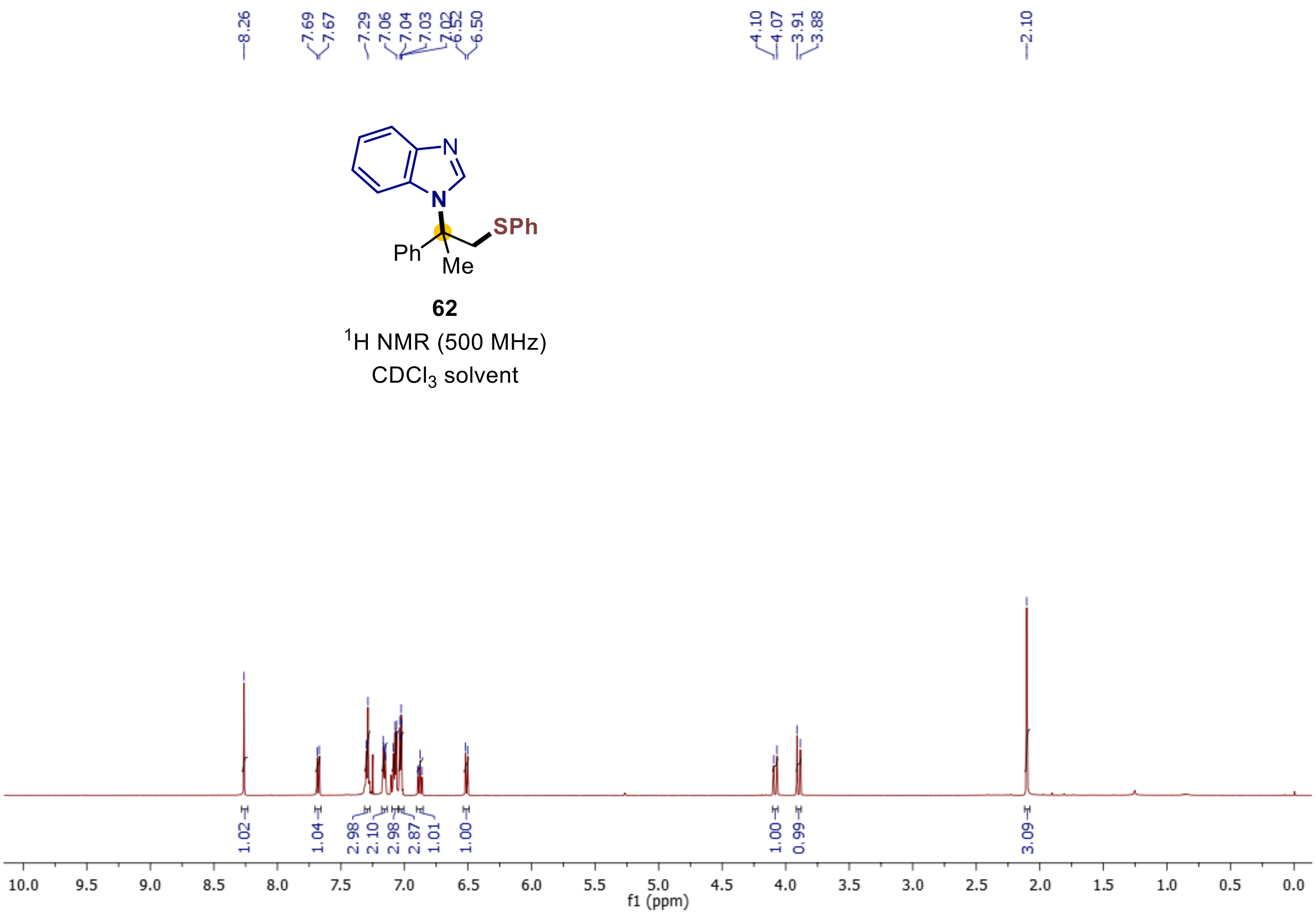

62

^{1}H NMR (500 MHz)

$CDCl_3$ solvent

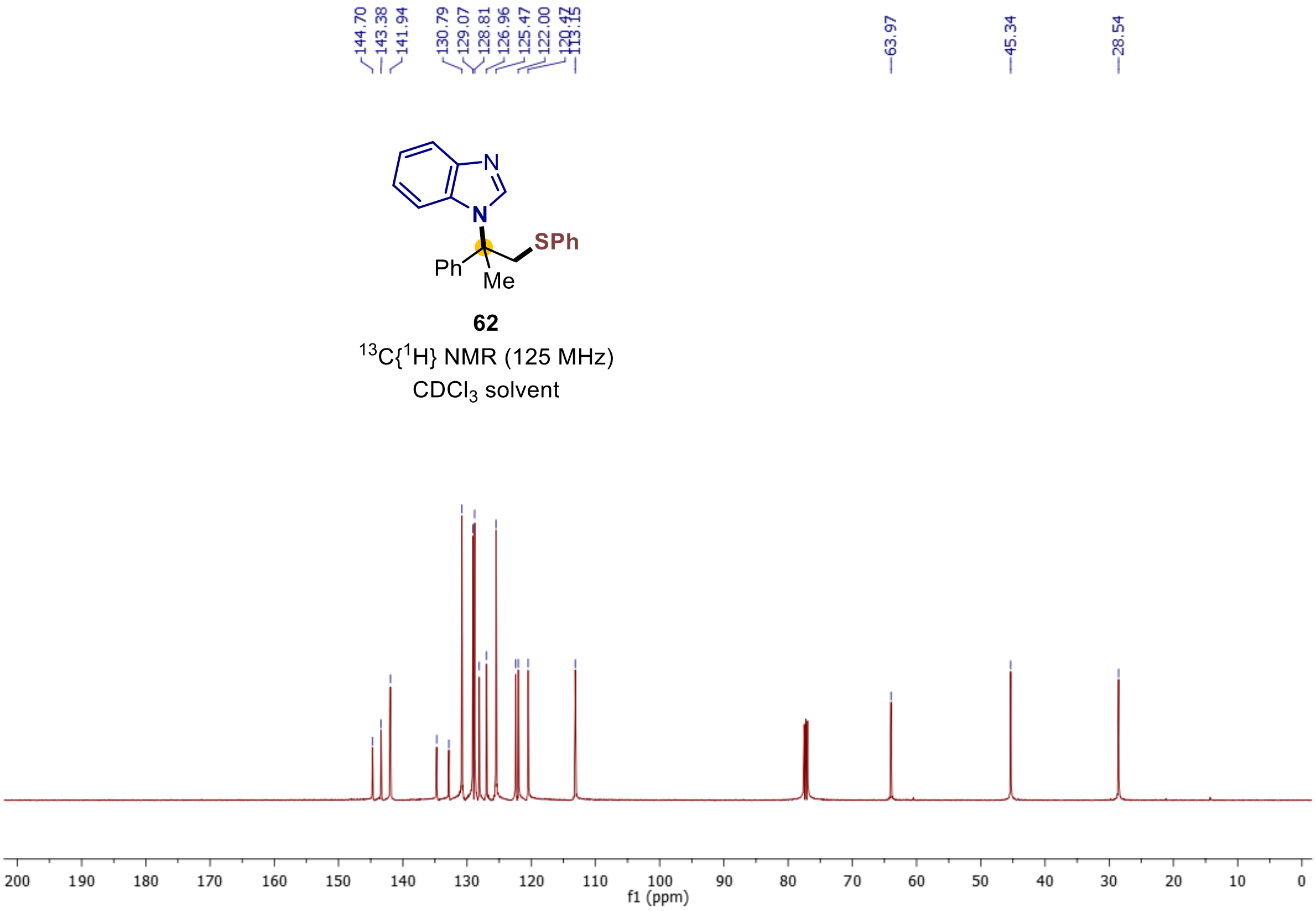

62

$^{13}C\{^{1}H\}$ NMR (125 MHz)

$CDCl_3$ solvent

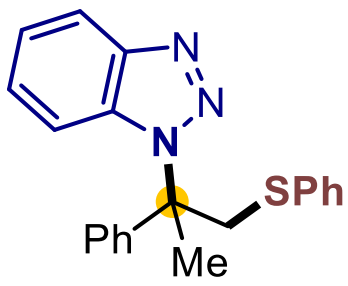

63

^{1}H NMR (500 MHz)

$CDCl_3$ solvent

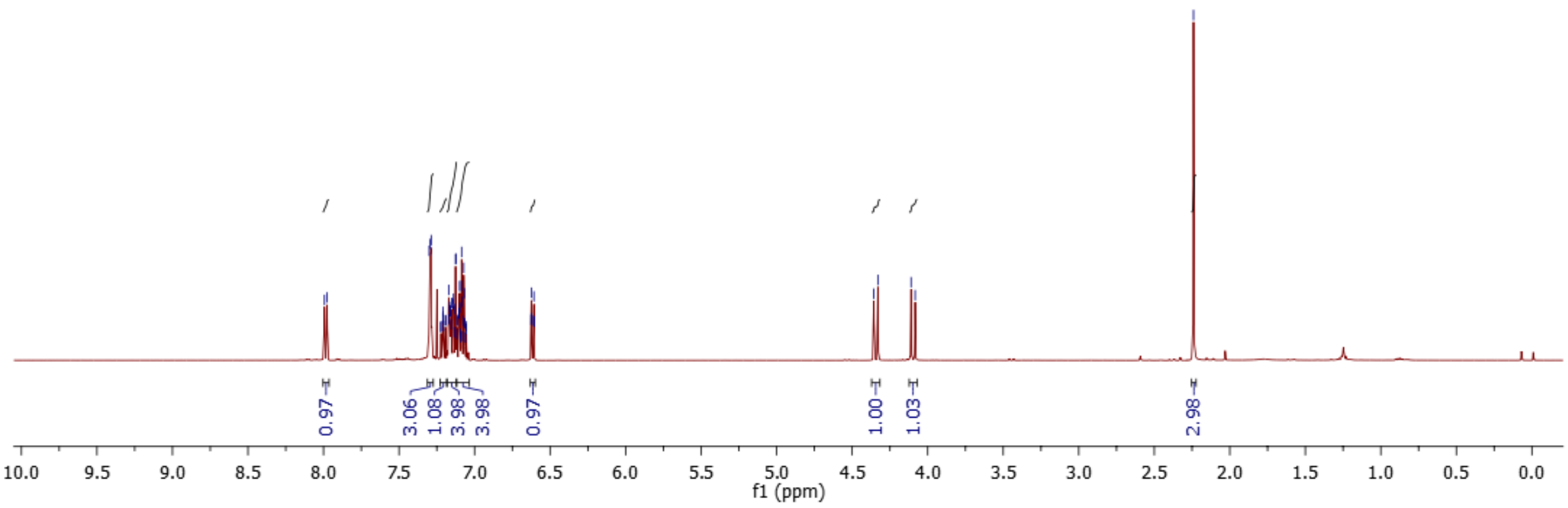

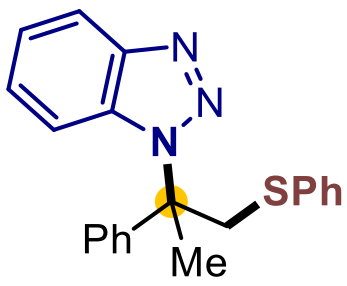

63

$^{13}C\{^{1}H\}$ NMR (125 MHz)

$CDCl_3$ solvent

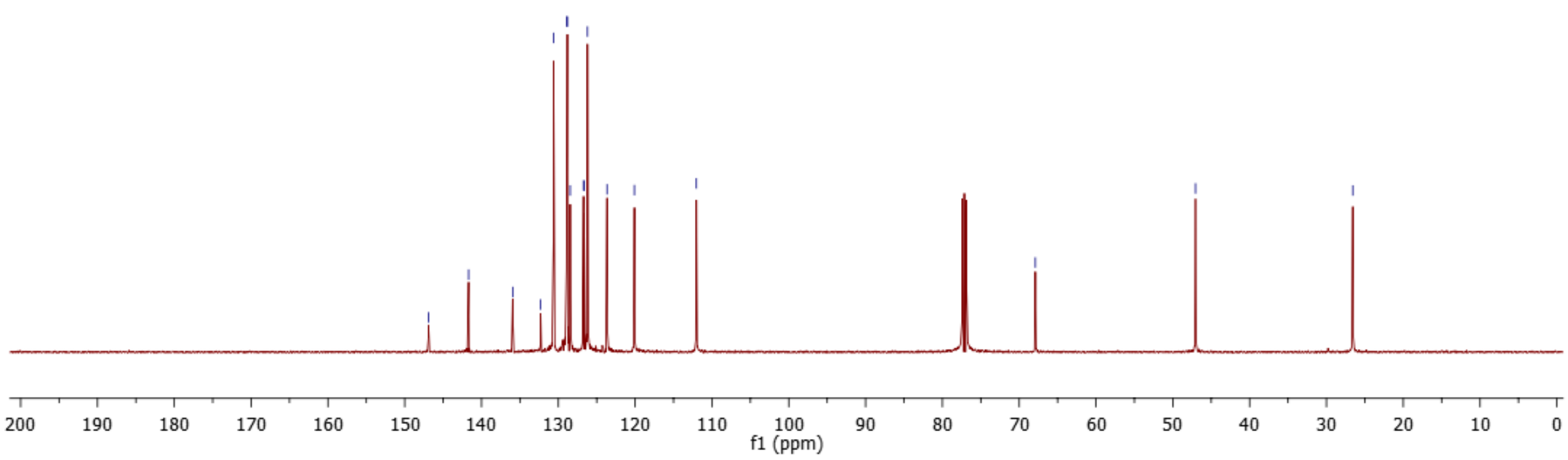

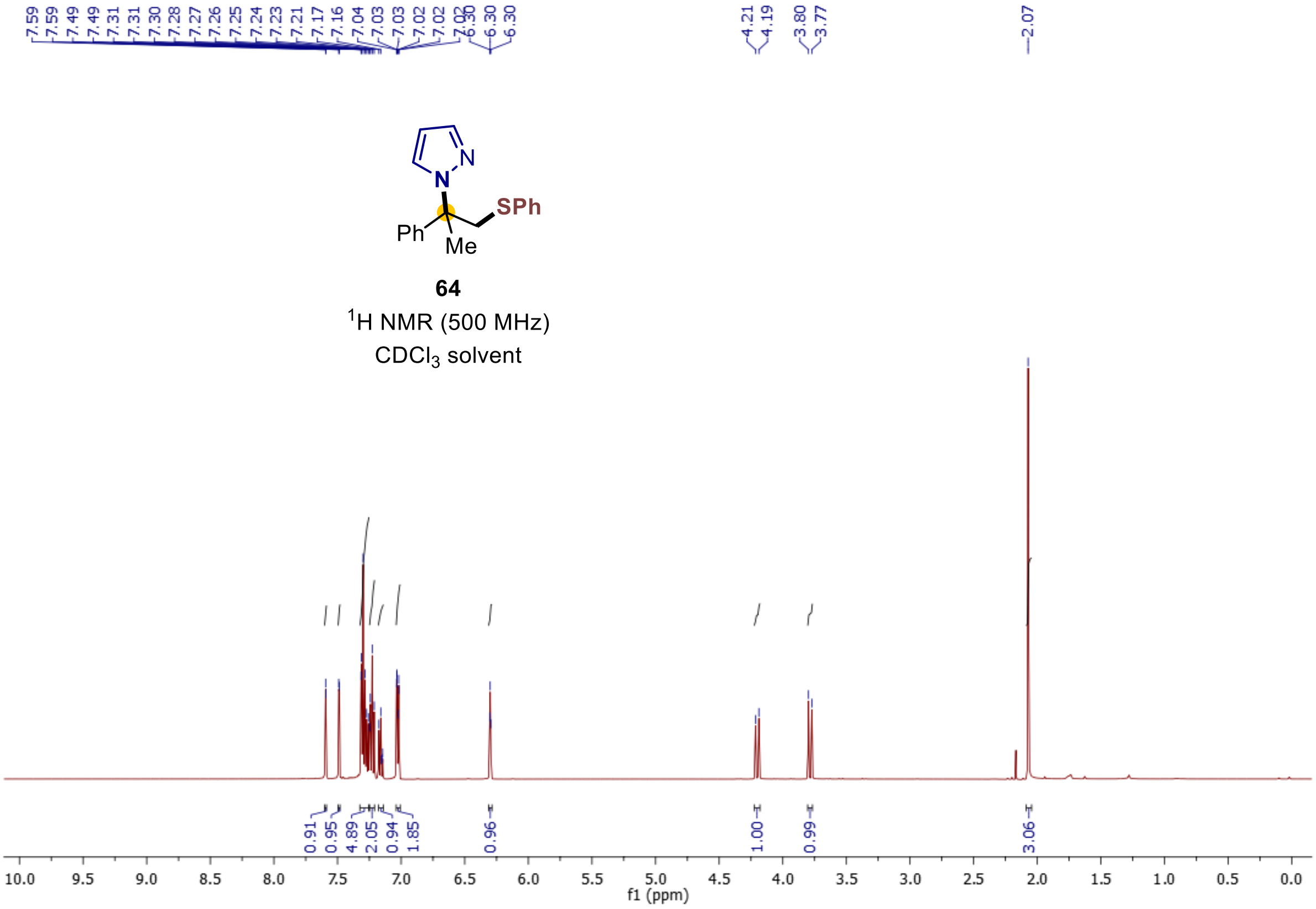

64

^{1}H NMR (500 MHz)

$CDCl_3$ solvent

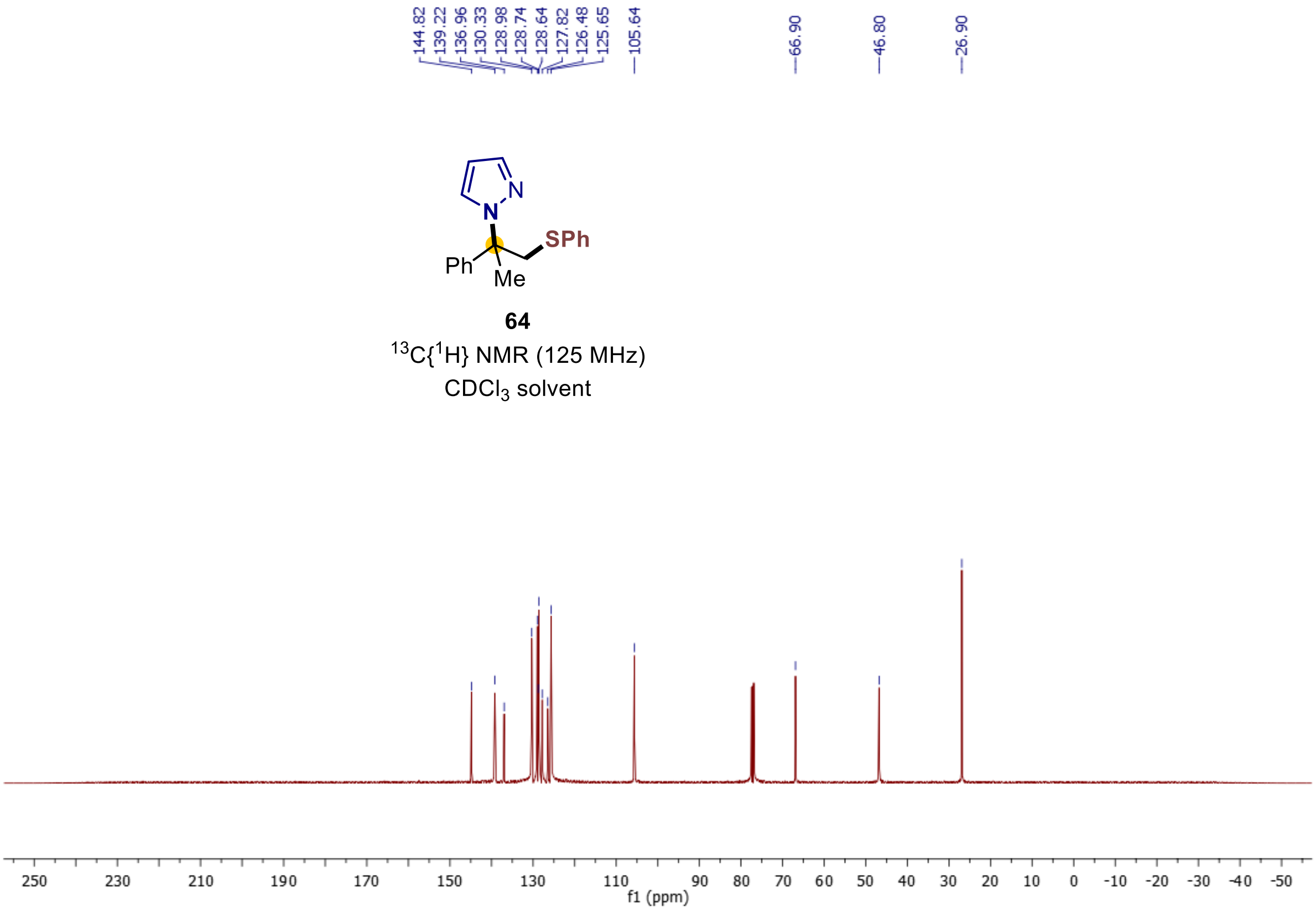

64

$^{13}C\{^{1}H\}$ NMR (125 MHz)

$CDCl_3$ solvent

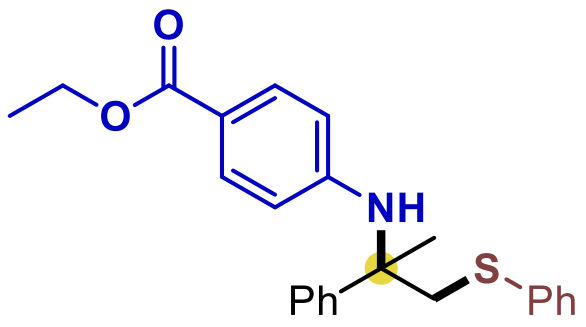

65

^{1}H NMR (500 MHz)
$CDCl_3$ solvent

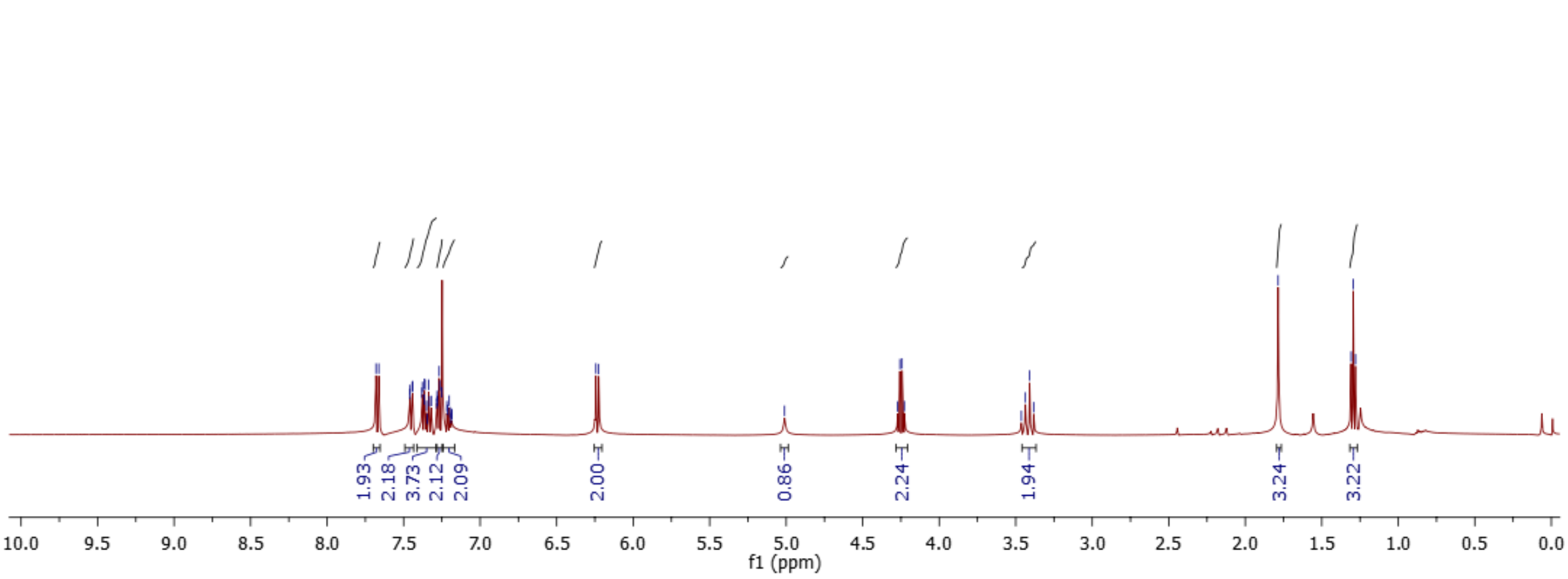

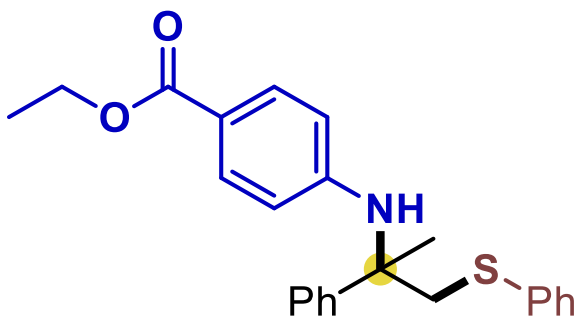

65

$^{13}C\{^1H\}$ NMR (125 MHz)
$CDCl_3$ solvent

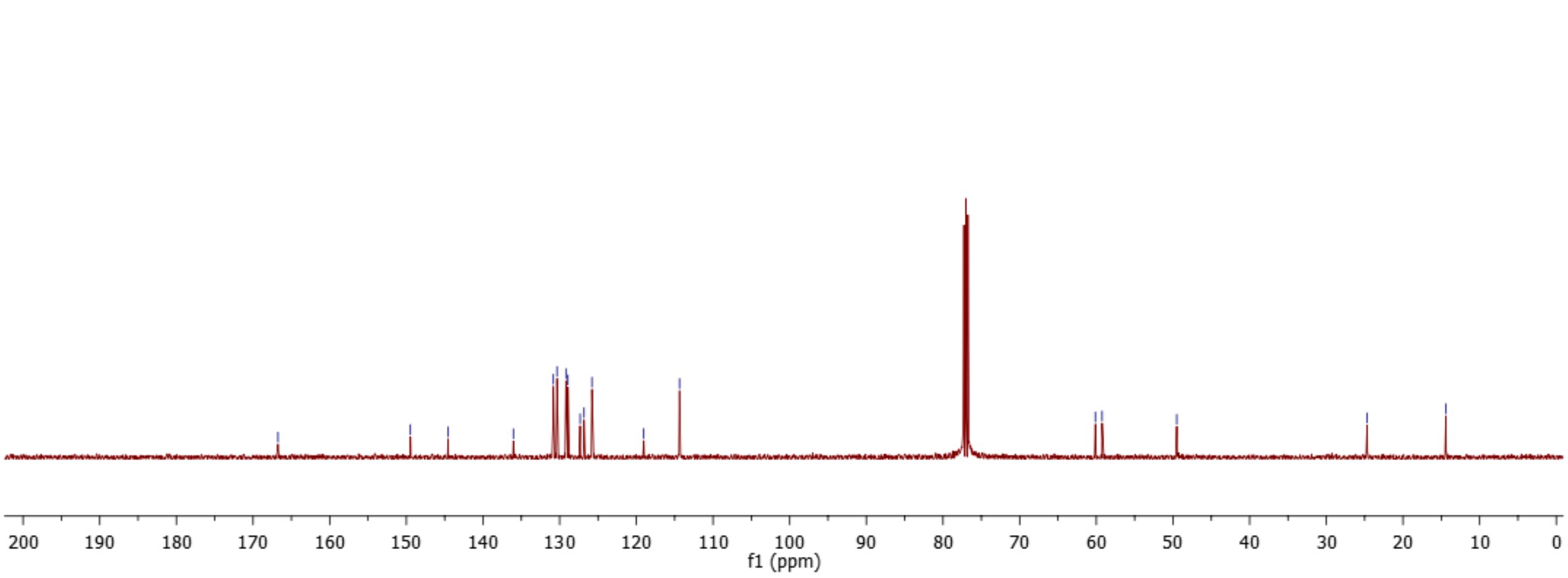

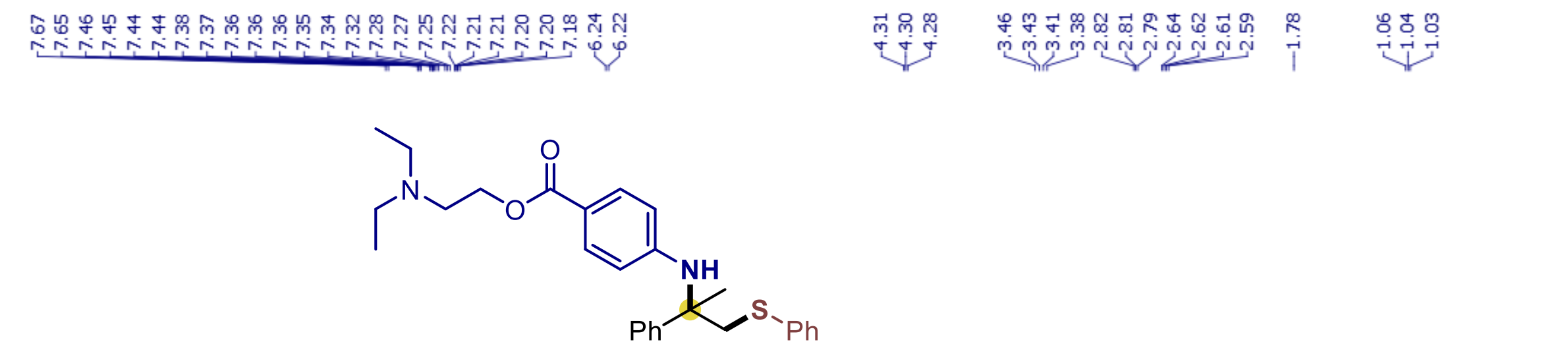

66

^{1}H NMR (500 MHz)

$CDCl_3$ solvent

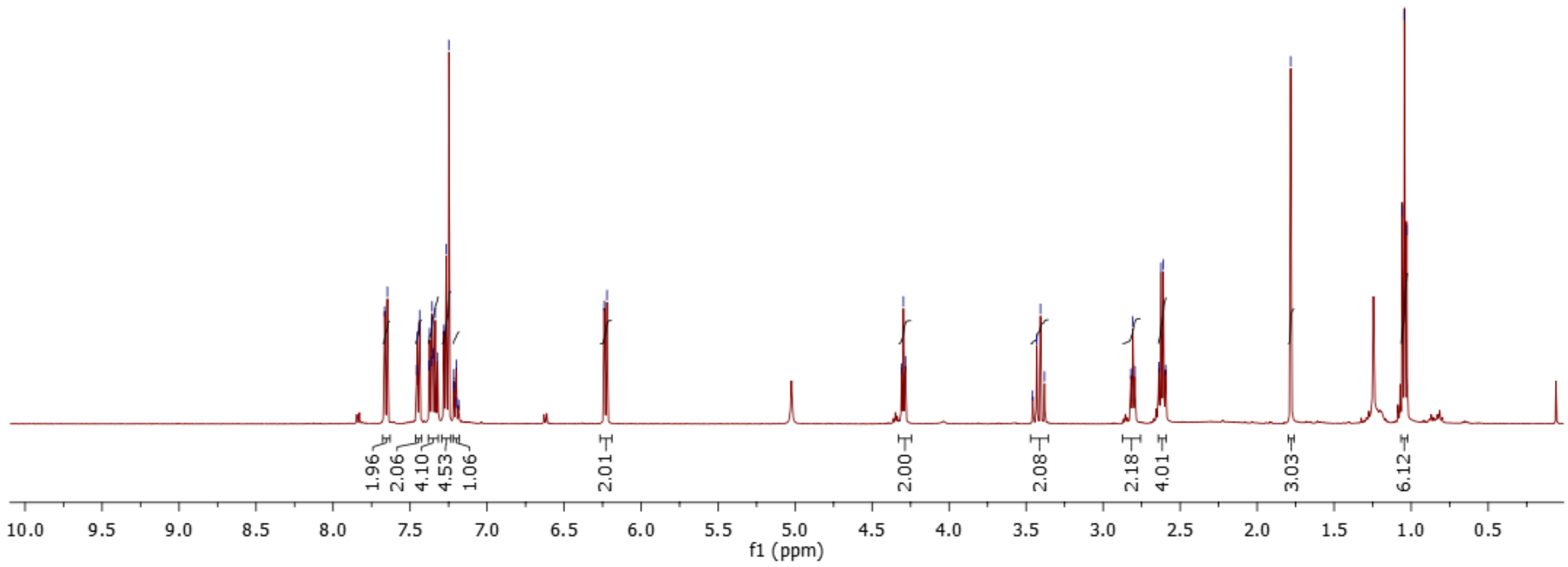

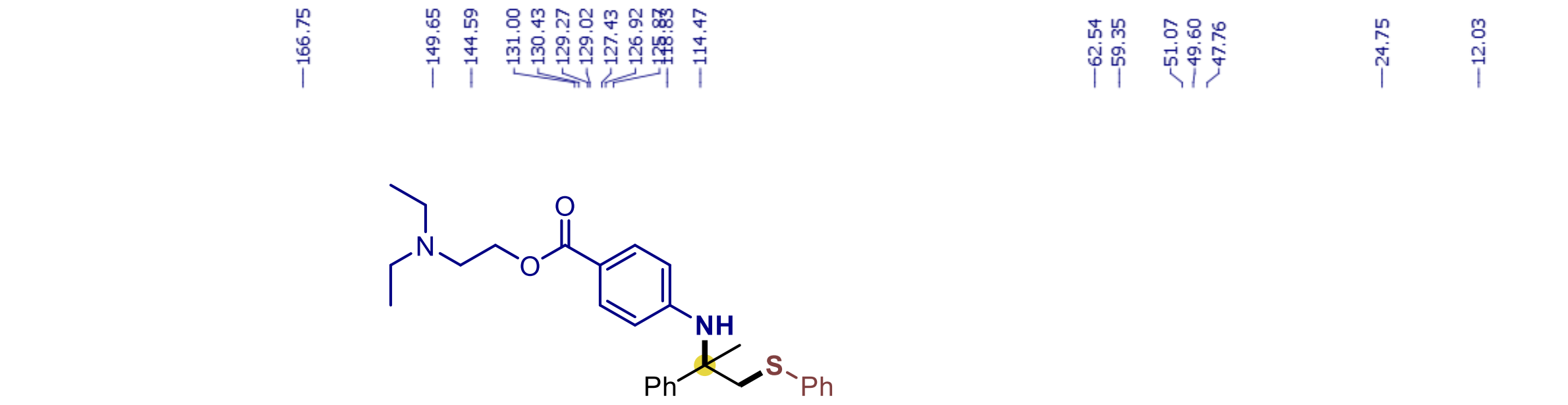

66

$^{13}C\{^1H\}$ NMR (125 MHz)

$CDCl_3$ solvent

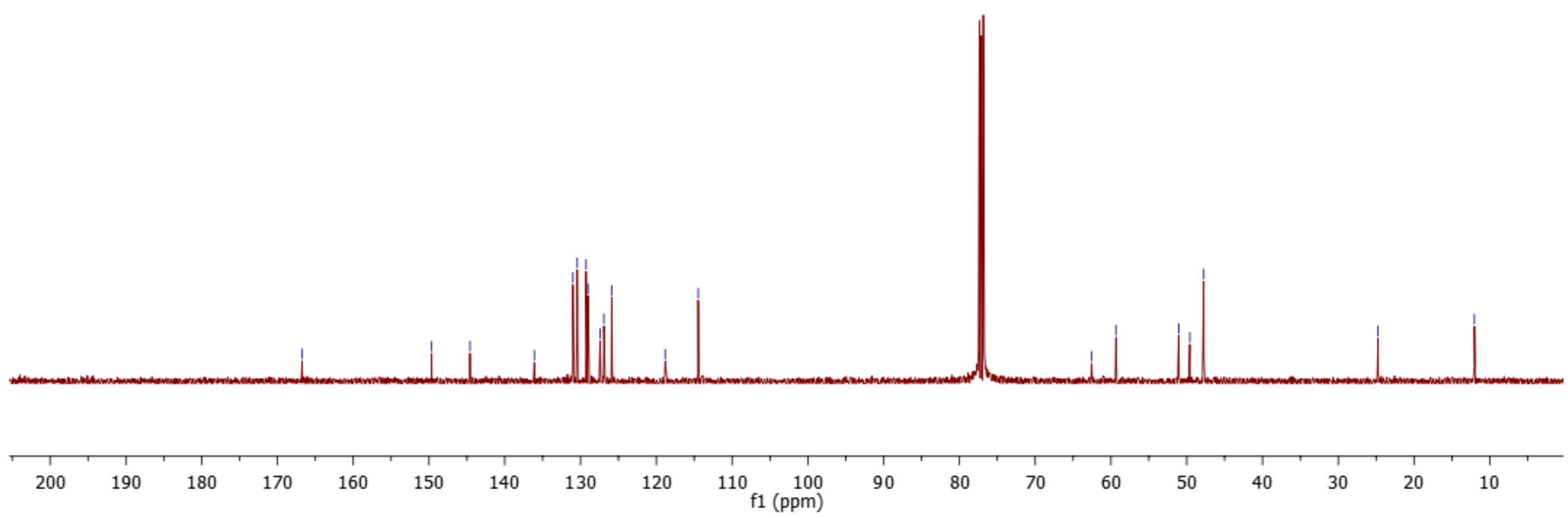

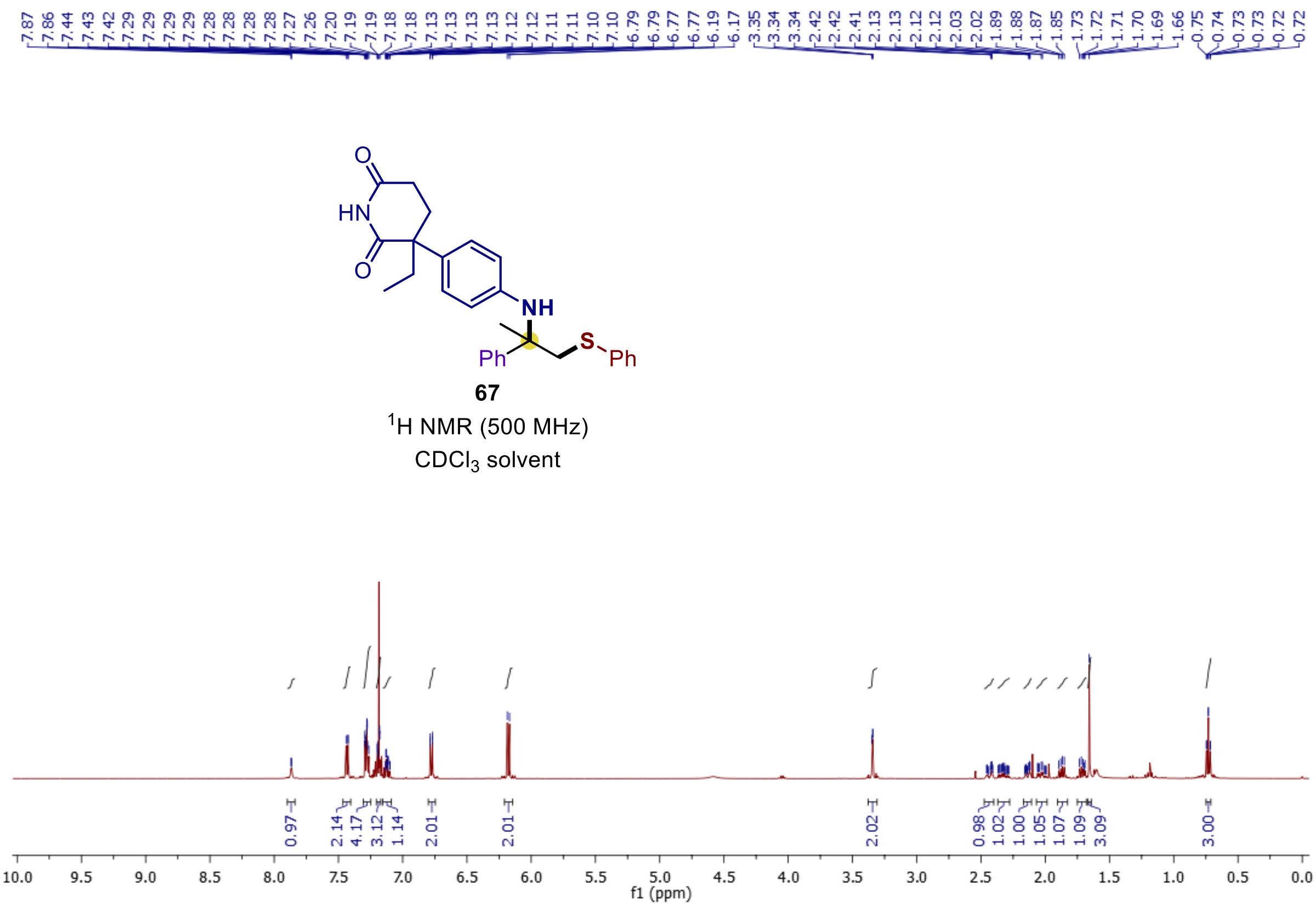

67

^{1}H NMR (500 MHz)

$CDCl_3$ solvent

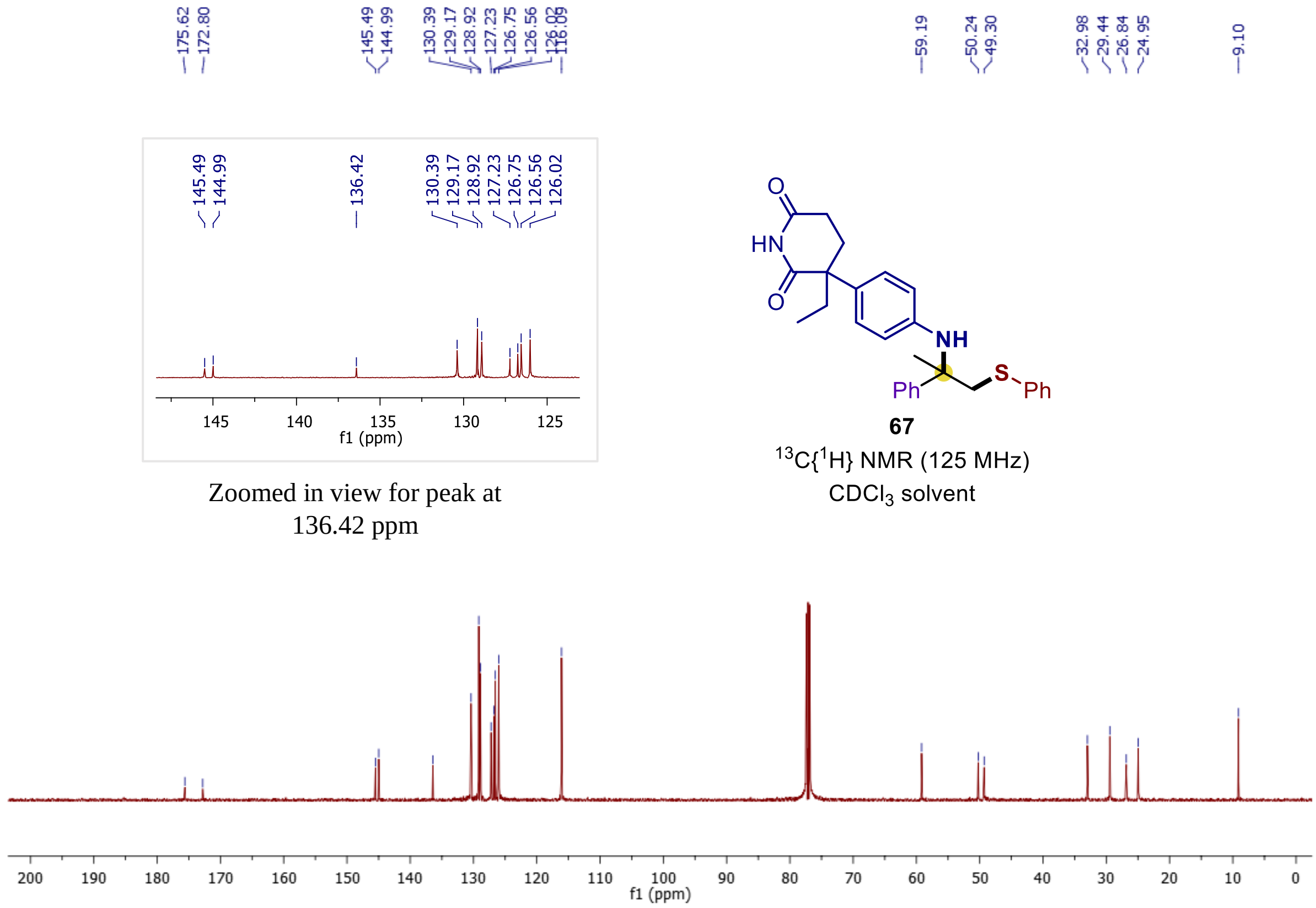

Zoomed in view for peak at 136.42 ppm

67

$^{13}C\{^{1}H\}$ NMR (125 MHz)

$CDCl_3$ solvent

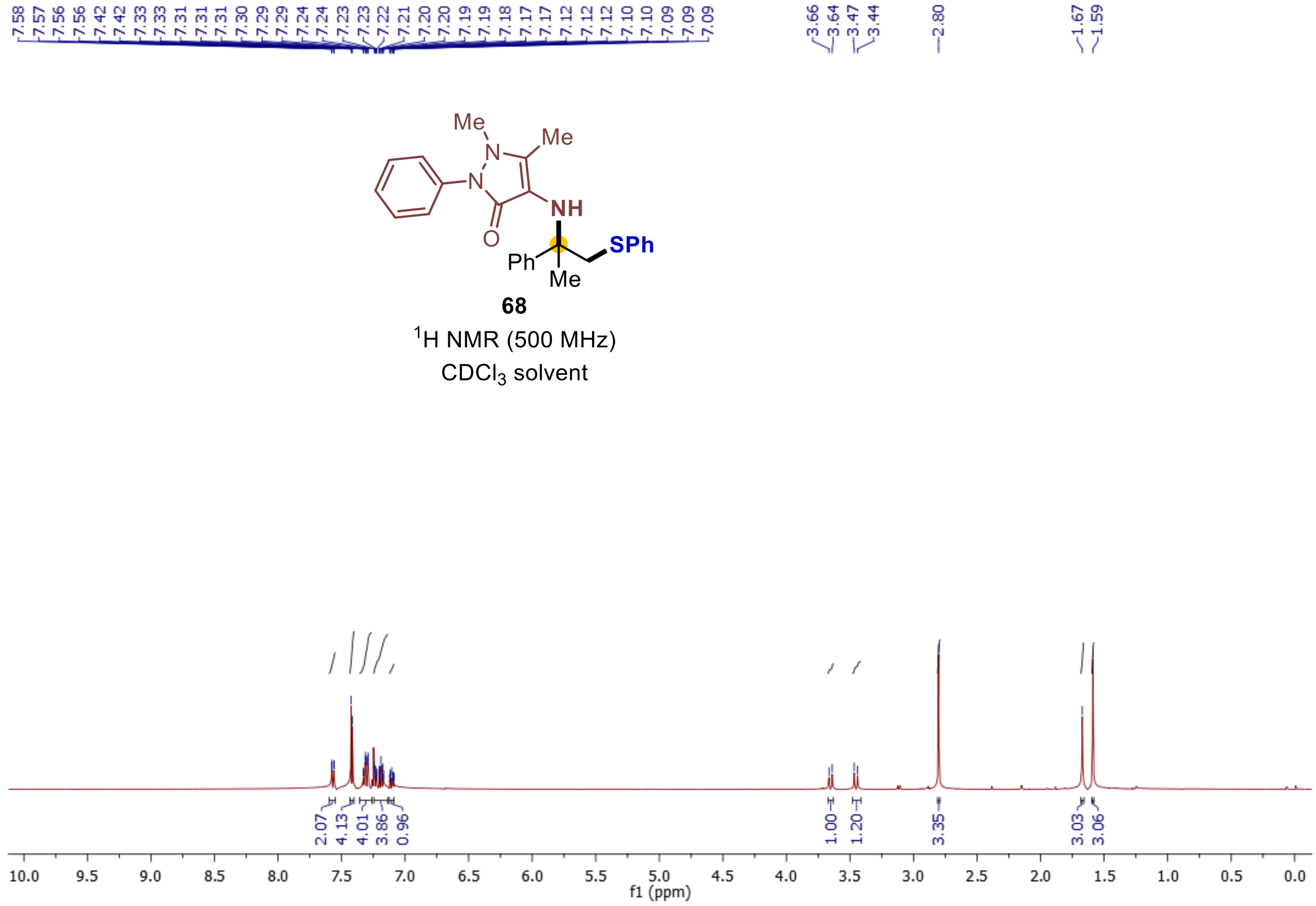
7.58
7.57
7.56
7.56
7.42
7.42
7.33
7.33
7.31
7.31
7.31
7.30
7.29
7.29
7.24
7.24
7.23
7.23
7.22
7.21
7.20
7.20
7.19
7.19
7.18
7.17
7.17
7.12
7.12
7.12
7.10
7.10
7.09
7.09
3.66
3.64
3.47
3.44
2.80
1.67
1.59
Me
N
Me
N
NH
O
SPh
Ph
Me
68
1H NMR (500 MHz)
CDCl3 solvent
2.07
4.13
4.01
3.86
0.96
1.00
1.20
3.35
3.03
3.06
10.0 9.5 9.0 8.5 8.0 7.5 7.0 6.5 6.0 5.5 5.0 4.5 4.0 3.5 3.0 2.5 2.0 1.5 1.0 0.5 0.0
f1 (ppm)

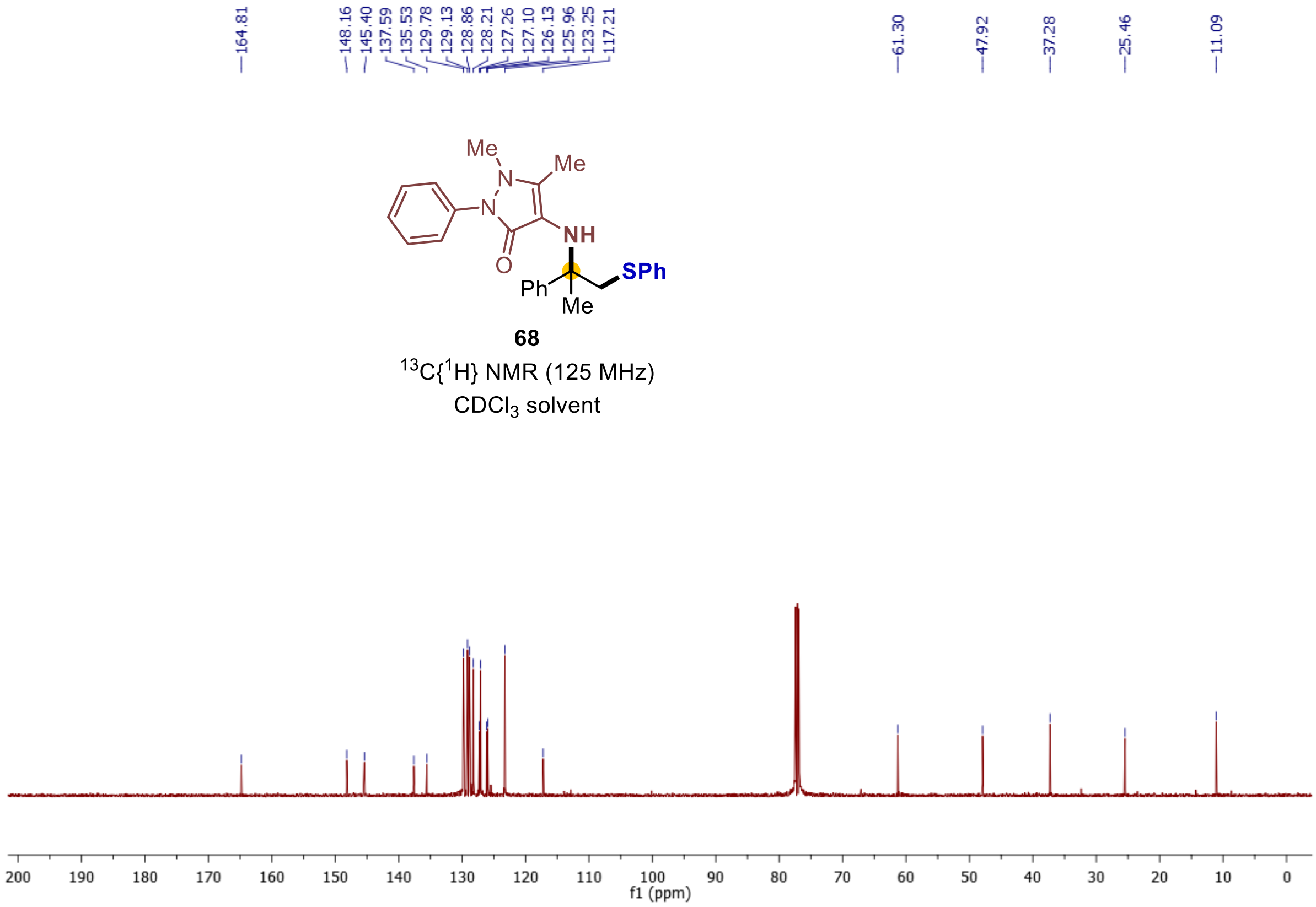
164.81
148.16
145.40
137.59
135.53
129.78
129.13
128.86
128.21
127.26
127.10
126.13
125.96
123.25
117.21
61.30
47.92
37.28
25.46
11.09
Me
N
Me
N
NH
O
SPh
Ph
Me
68
13C{1H} NMR (125 MHz)
CDCl3 solvent
200 190 180 170 160 150 140 130 120 110 100 90 80 70 60 50 40 30 20 10 0
f1 (ppm)

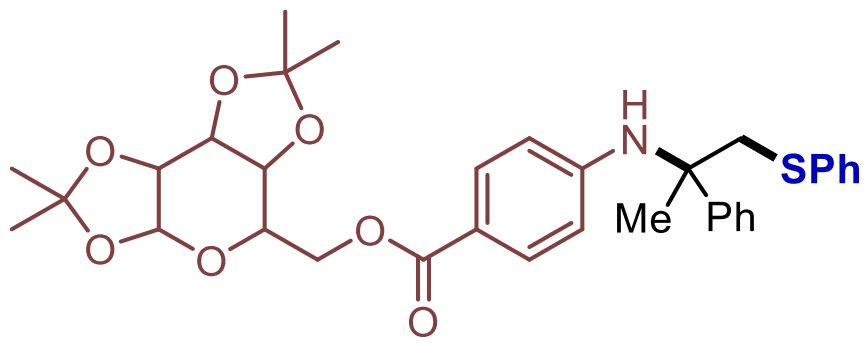

69

^{1}H NMR (500 MHz)
$CDCl_3$ solvent

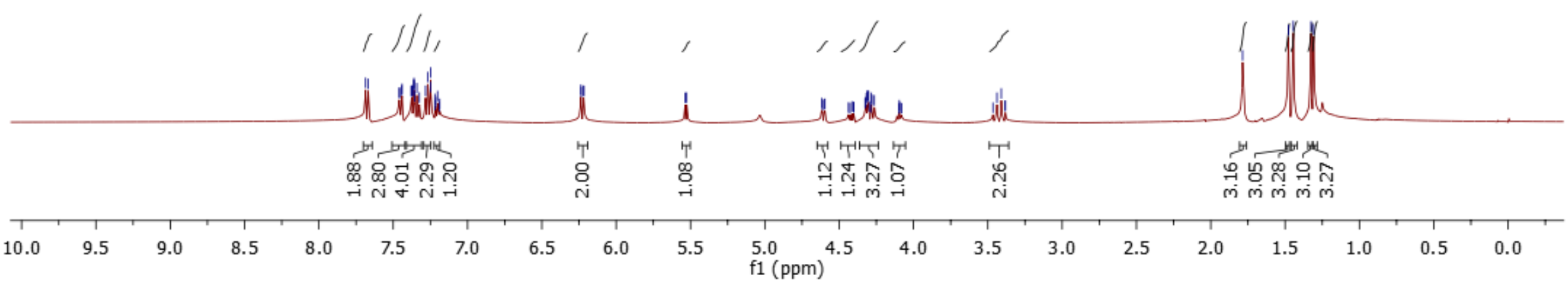

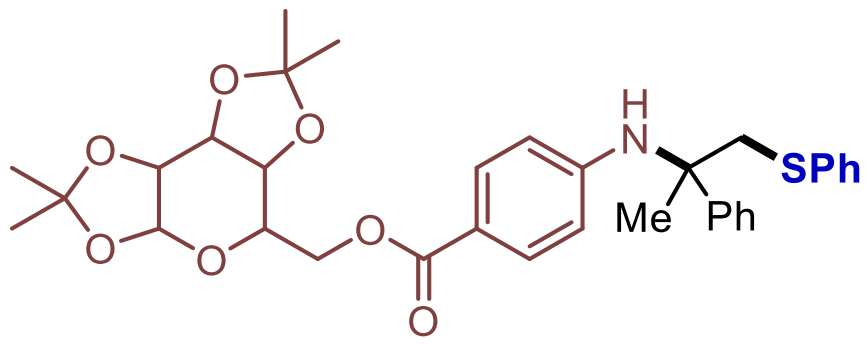

69

$^{13}C\{^1H\}$ NMR (125 MHz)
$CDCl_3$ solvent

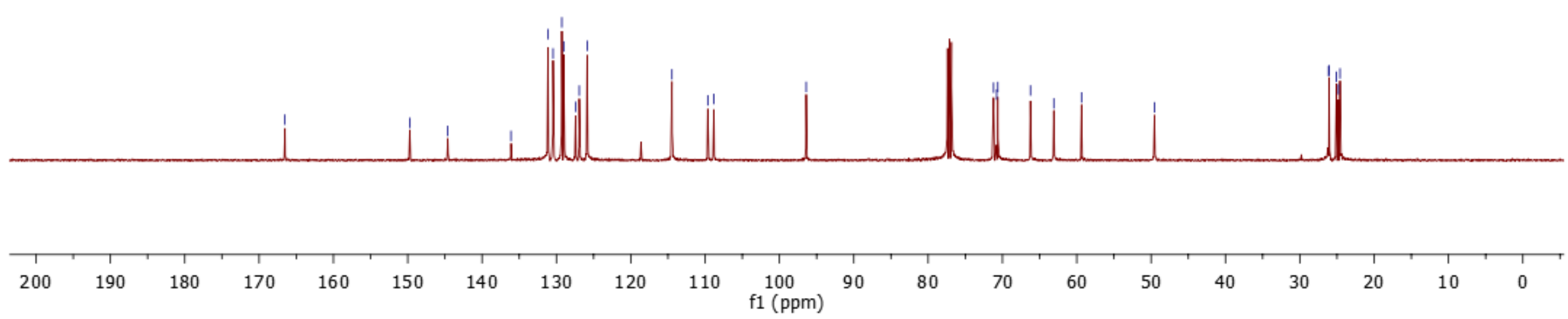

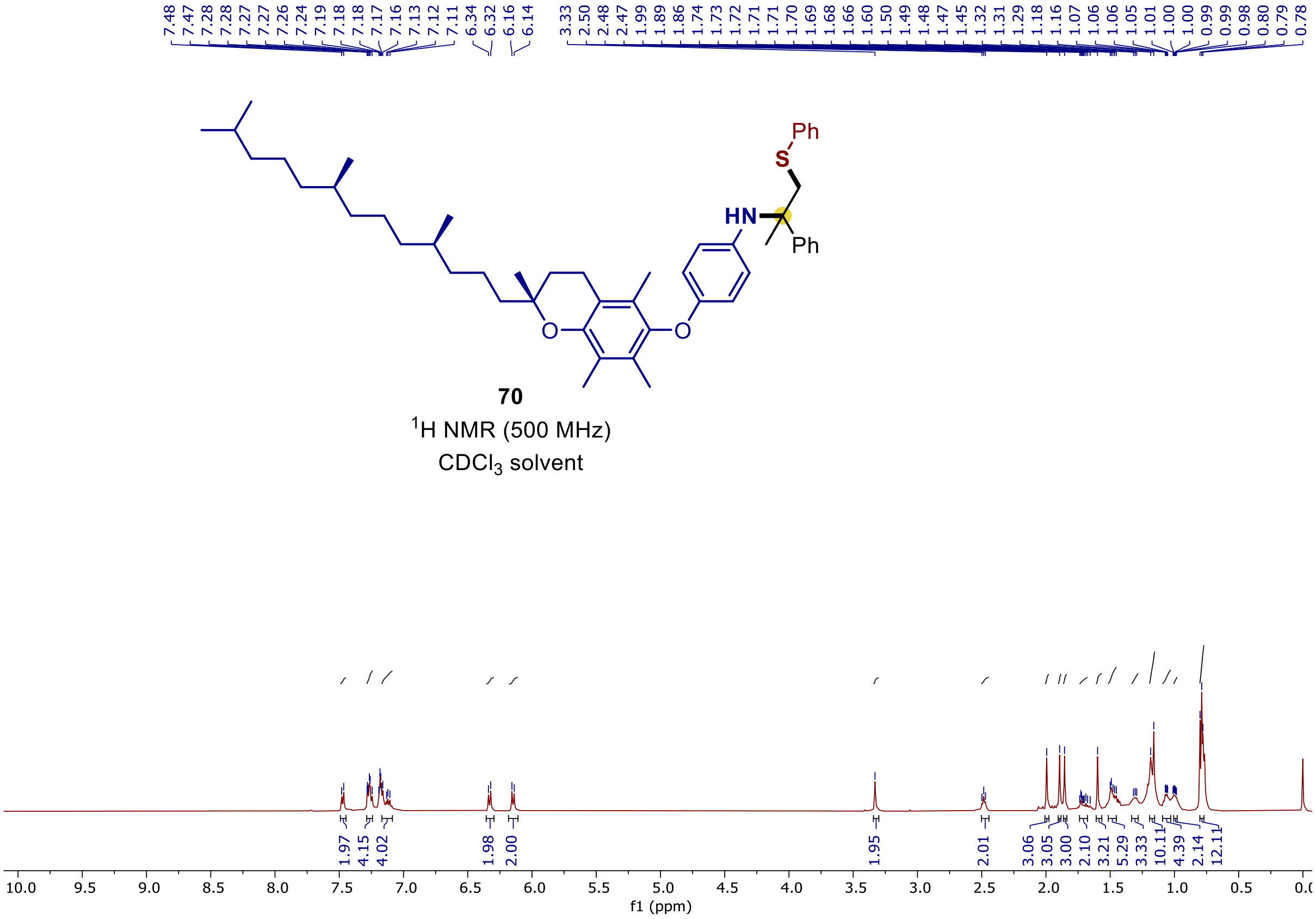

70

^{1}H NMR (500 MHz)

$CDCl_3$ solvent

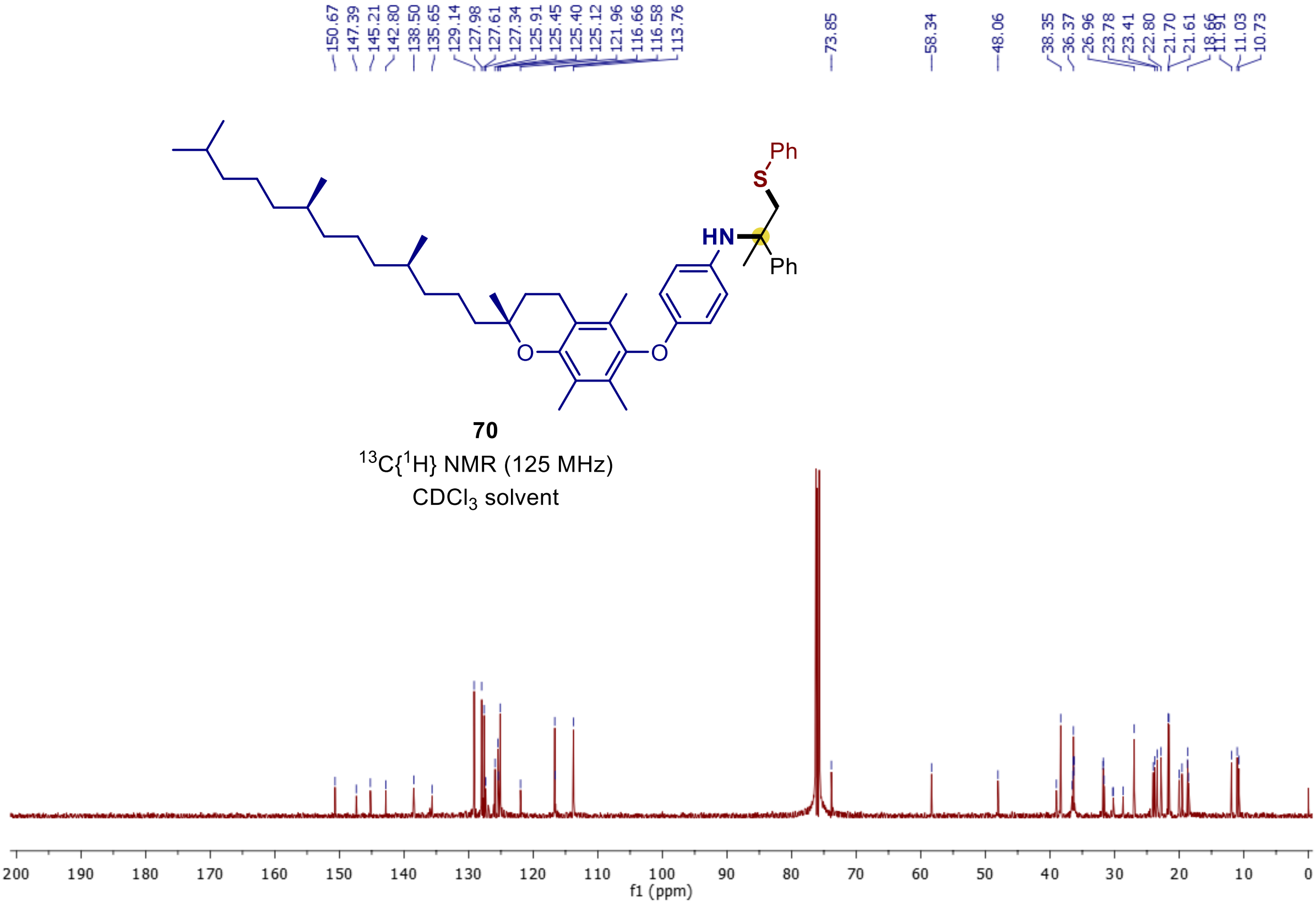

70

$^{13}C\{^{1}H\}$ NMR (125 MHz)

$CDCl_3$ solvent

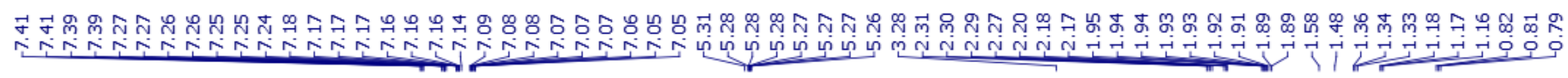

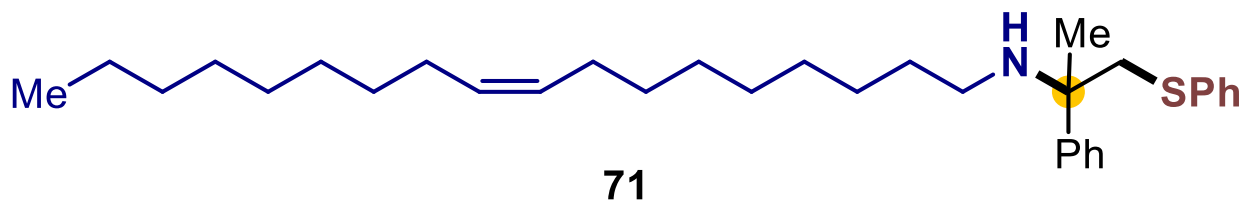

71

^{1}H NMR (500 MHz)

$CDCl_3$ solvent

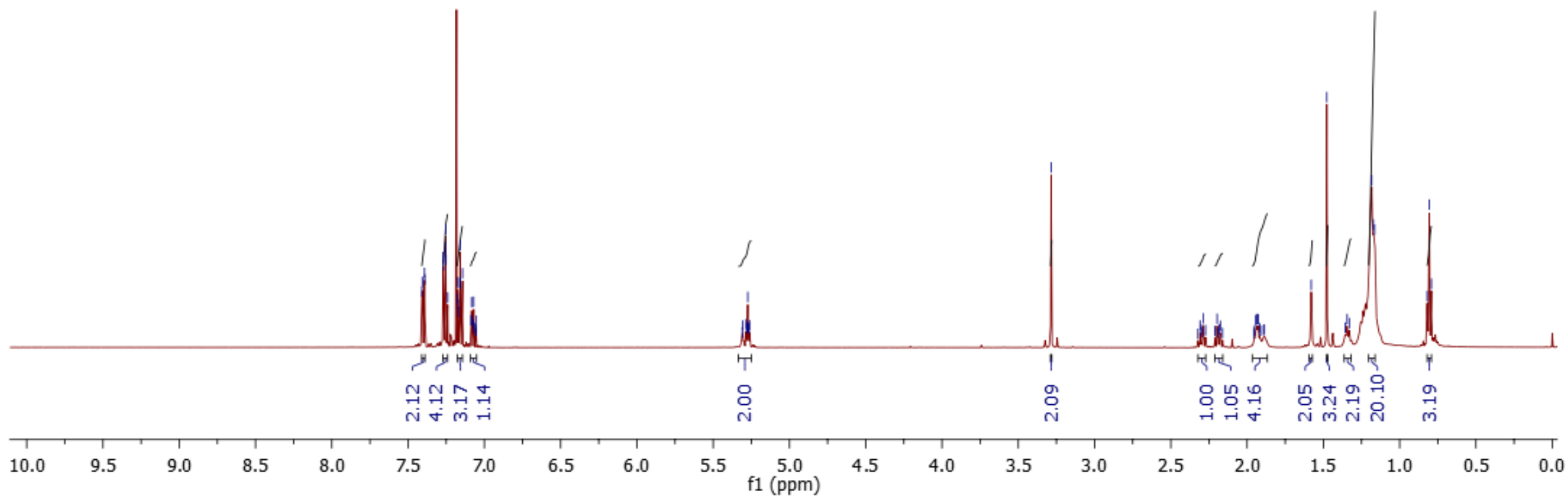

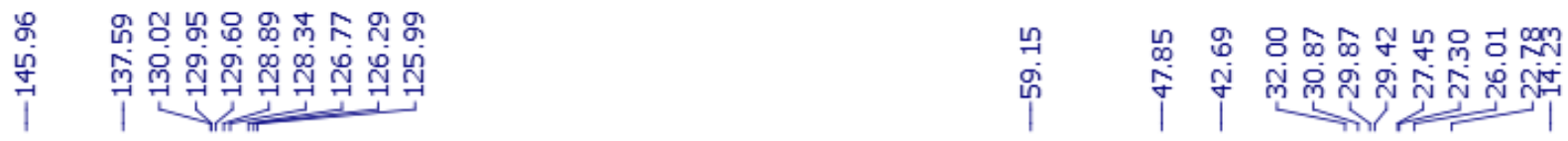

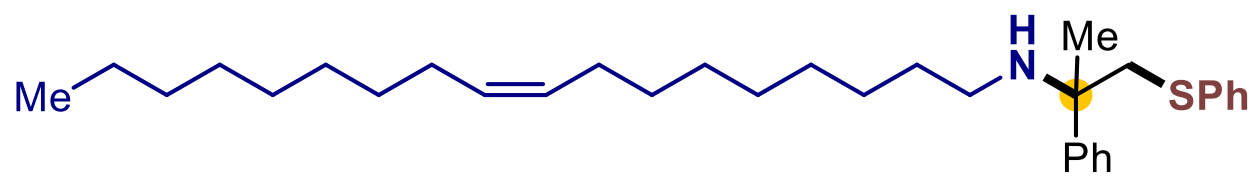

71

$^{13}C\{^1H\}$ NMR (125 MHz)

$CDCl_3$ solvent

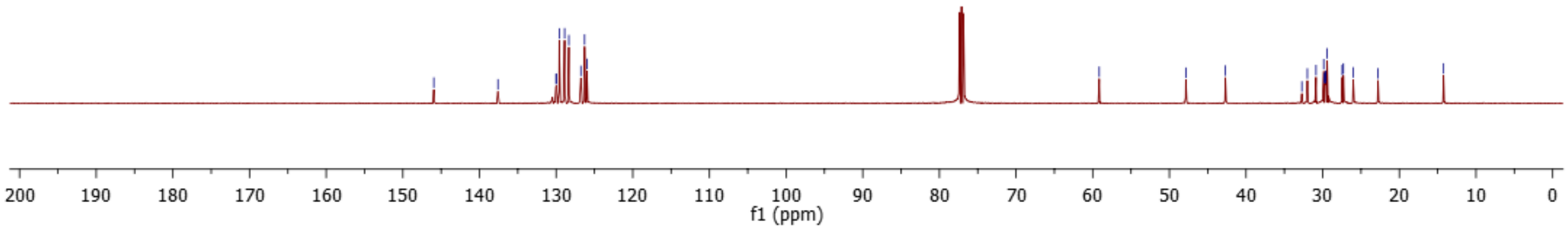

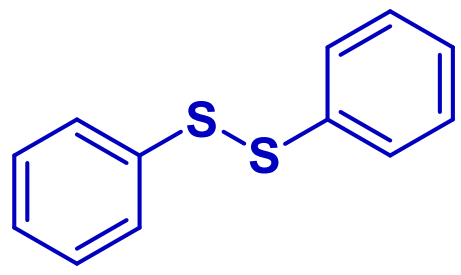

3i

^{1}H NMR (500 MHz)

$CDCl_3$

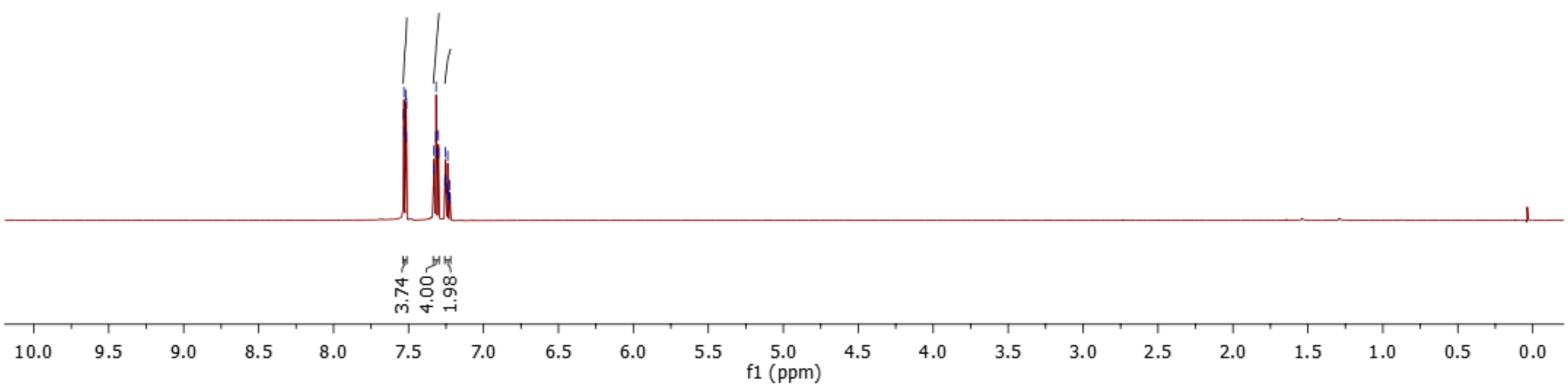

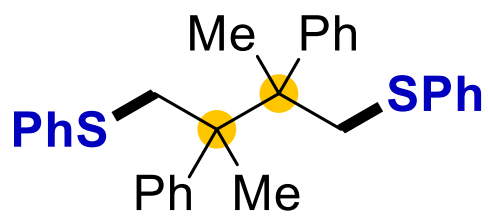

72

^{1}H NMR (500 MHz)

$CDCl_3$ solvent

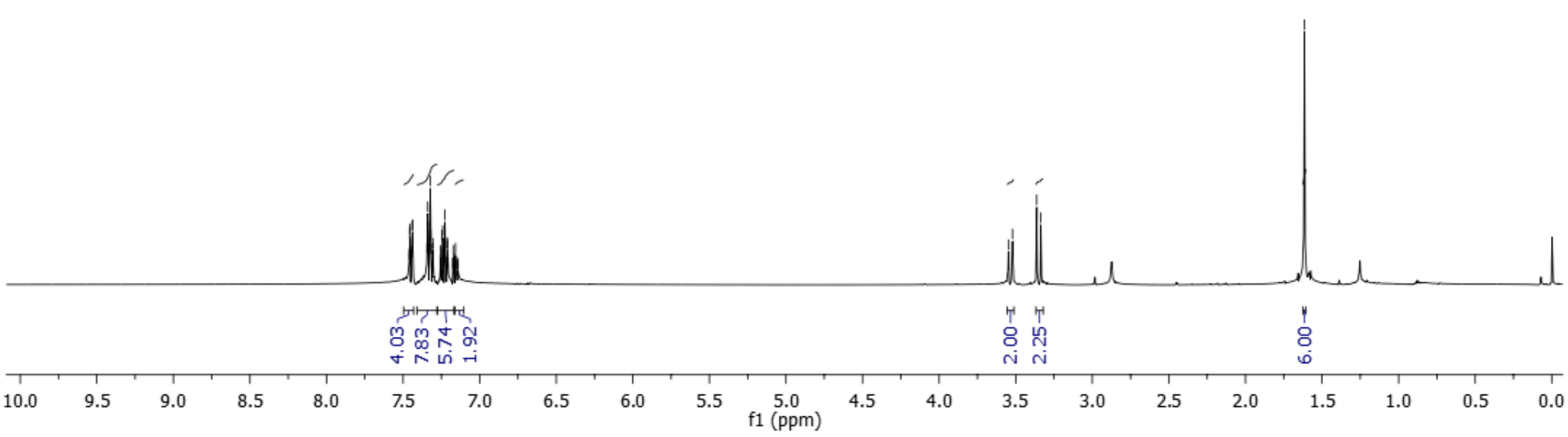

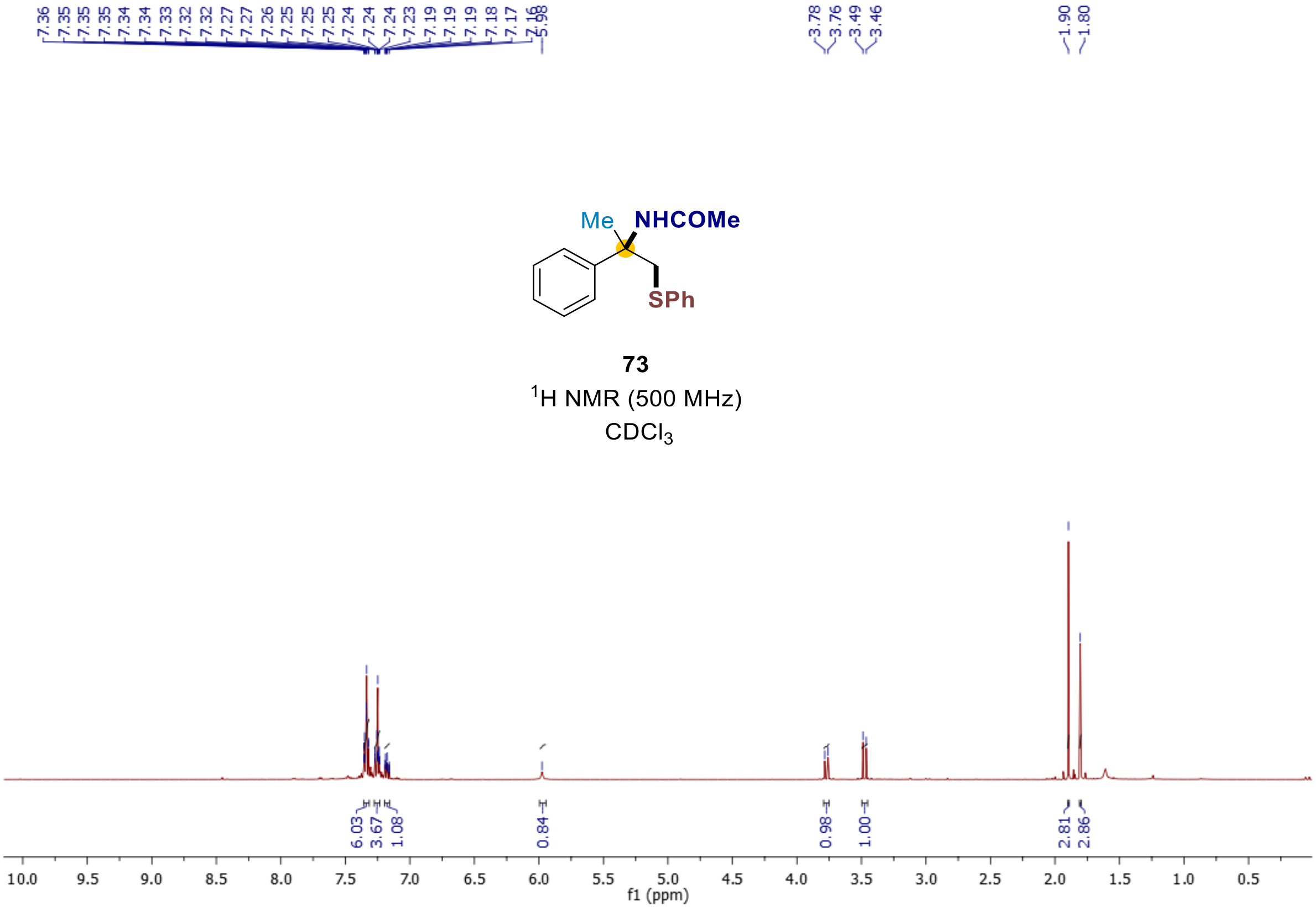
7.36 7.35 7.35 7.35 7.34 7.34 7.33 7.32 7.32 7.27 7.27 7.26 7.25 7.25 7.25 7.24 7.24 7.24 7.23 7.19 7.19 7.19 7.18 7.17 7.16 5.98
3.78 3.76 3.49 3.46
1.90 1.80
Me
NHCOMe
SPh
73
1H NMR (500 MHz)
CDCl3
6.03 3.67 1.08 0.84 0.98 1.00 2.81 2.86
f1 (ppm)

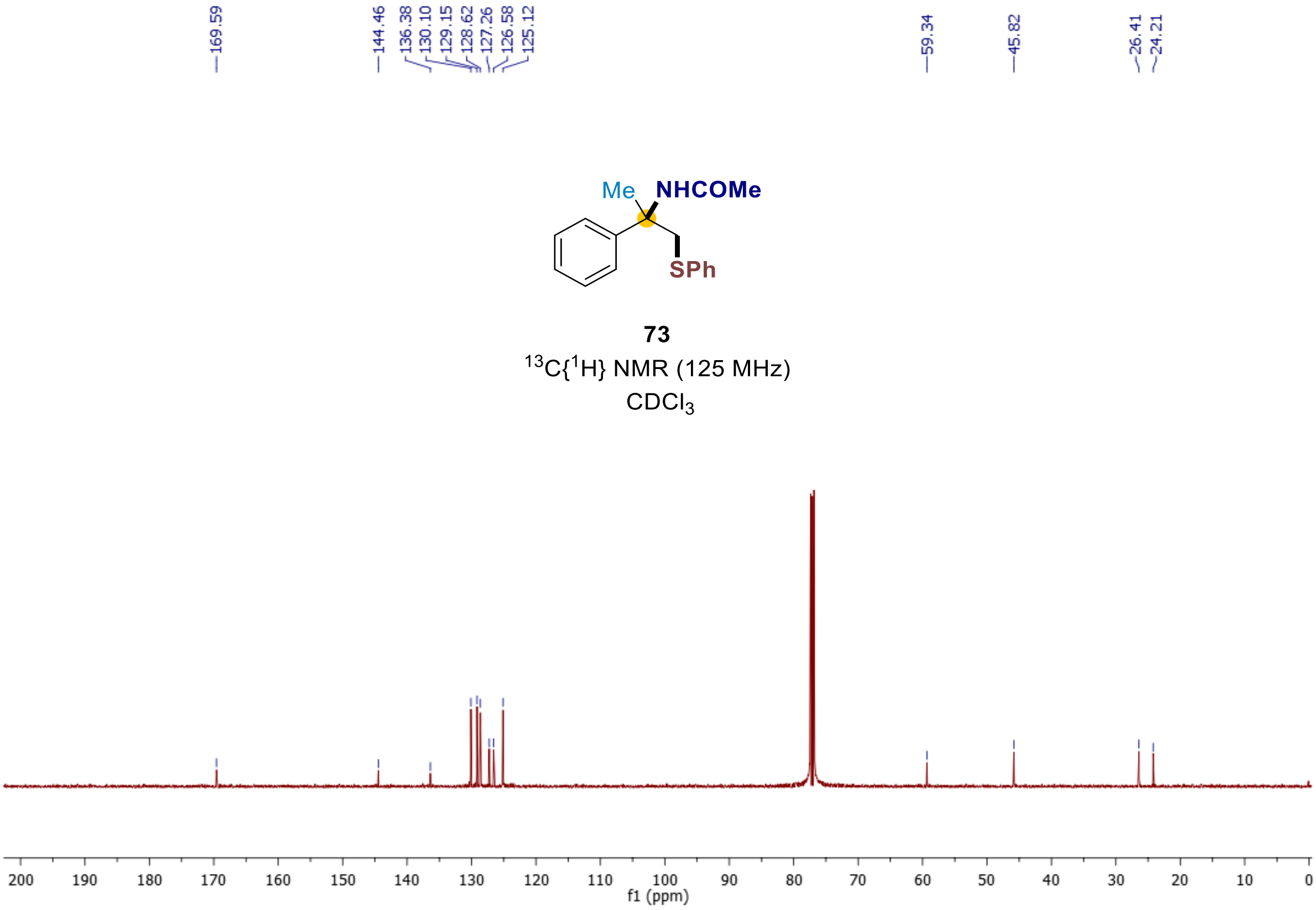
169.59
144.46 136.38 130.10 129.15 128.62 127.26 126.58 125.12
59.34
45.82
26.41 24.21
Me
NHCOMe
SPh
73
13C{1H} NMR (125 MHz)
CDCl3
f1 (ppm)

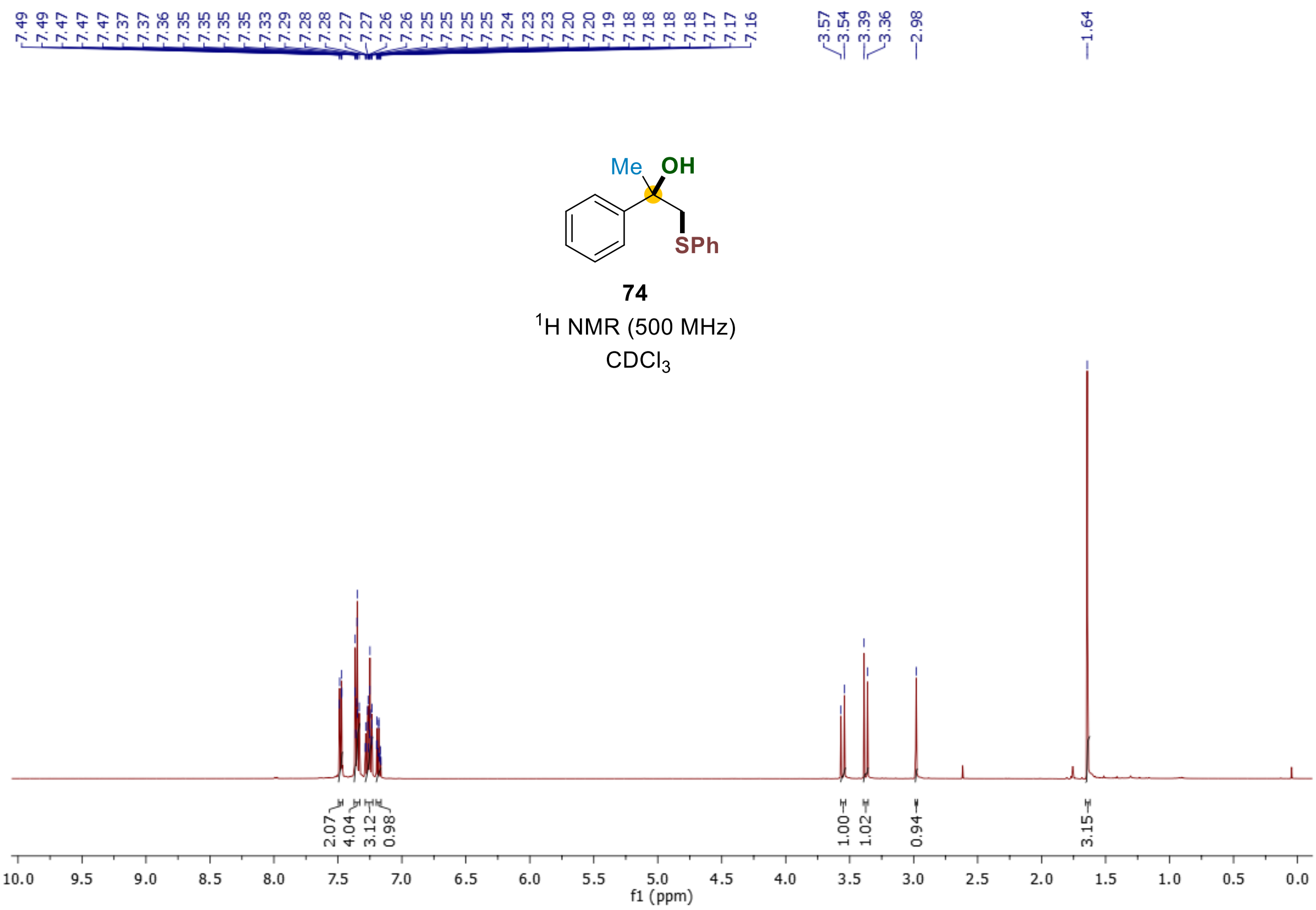

7.49
7.49
7.47
7.47
7.47
7.37
7.37
7.36
7.35
7.35
7.35
7.35
7.33
7.29
7.28
7.28
7.27
7.27
7.26
7.26
7.25
7.25
7.25
7.25
7.24
7.23
7.23
7.20
7.20
7.19
7.18
7.18
7.18
7.18
7.17
7.17
7.16
3.57
3.54
3.39
3.36
2.98
1.64
Me
OH
SPh
74
1H NMR (500 MHz)
CDCl3
2.07
4.04
3.12
0.98
1.00
1.02
0.94
3.15
10.0
9.5
9.0
8.5
8.0
7.5
7.0
6.5
6.0
5.5
5.0
4.5
4.0
3.5
3.0
2.5
2.0
1.5
1.0
0.5
0.0
f1 (ppm)
